# Global burden, health inequalities and improvement gap of head and neck cancers in middle-aged and older adults from 1990 to 2021

**DOI:** 10.1371/journal.pone.0335969

**Published:** 2025-11-06

**Authors:** Minxia Yang, Feng Xuan, Xiaofeng Ma, Zhaoqi Qiu

**Affiliations:** 1 Department of Radiology, Shaoxing People’s Hospital, Shaoxing, Zhejiang Province, China; 2 Department of Radiation Oncology, Zhuji Affiliated Hospital of Wenzhou Medical University, Shaoxing, China; 3 Department of Radiology, Zhuji Affiliated Hospital of Wenzhou Medical University, Shaoxing, China; Faculdade Ciencias Medicas de Minas Gerais, BRAZIL

## Abstract

**Background:**

This study aimed to assess temporal trends, health inequities and potential improvements in the burden of head and neck cancer (HNC) in middle-aged and older adults between 1990 and 2021, focusing on three major subtypes: larynx, nasopharynx, and lip/oral cavity cancers.

**Methods:**

A secondary analysis of the Global Burden of Disease Study (GBD) 2021 was performed, using age-standardised incidence rates (ASIR) and age-standardised disability-adjusted life years (ASDR) to quantify the burden of HNC. The average annual percent change was calculated to analyzed trends. The slope index of inequality (SII) and the concentration index quantified health inequities. Frontier analysis identified regions with potential for improvement.

**Result:**

In 2021, there were approximately 650,205 new cases of overall HNC globally, resulting in 9,621,610 DALYs, with ASIR and ASDR both declining since 1990. ASIR exhibited a decrease for laryngeal and nasopharyngeal cancers, in contrast to an increase for lip and oral cavity cancers. ASDR decreased across all cancer types. The SII showed a notable shift in ASDR from countries with higher socio-demographic indices (SDI) in 1990 to those with lower SDI countries by 2021. Meantime, the concentration index revealed a worsening inequality in lower SDI countries. Frontier analyses across 204 countries and territories indicated that certain high SDI countries could effectively reduce ASDR for HNCs.

**Conclusion:**

The global burden of HNCs shown considerable regional disparities. Health inequalities have persisted, with lower SDI regions bearing a heavier burden, particularly in laryngeal and lip/oral cavity cancers. Developing tailored national cancer control plans and enhancing international medical cooperation are essential to reduce HNC burden and promote equitable health outcomes.

## 1. Introduction

Head and neck cancer (HNC) encompasses a variety of malignant tumors that arise from the tissues of the larynx, nasopharynx, oral cavity, and lips [1,2]. Based on the Global Cancer Statistics 2022, the mortality rates for lip and oral cavity cancer, laryngeal cancer, and nasopharyngeal cancer were ranked 15th, 18th, and 21st, respectively, in 2022 [3]. Most of these cancers, approximately 90%, develop from squamous cells [2]. These organs are responsible for vital processes, such as respiration and swallowing, and play a key role in filtering and humidifying inhaled air. Despite the use of multimodal treatments, including surgery, radiotherapy, and chemotherapy, the prognosis for HNC remains poor [4]. Furthermore, most patients are diagnosed at advanced stages, where pharmacologic interventions show limited efficacy. Additionally, surgical treatments at these stages frequently lead to considerable functional impairment, further significantly increasing the burden on healthcare systems [4].

Age is a significant risk factor for cancer, with most cases diagnosed in those over 40 years old [5]. Due to the growing and aging population, combined with advancements in cancer detection and treatment, the number of cancer survivors worldwide is steadily increasing [3,6]. Unlike younger patients, middle-aged and elderly patients have very different tumor biological characteristics and responses to treatment, and elderly patients often have varying degrees of comorbidities and are less tolerant to treatment [7–9]. While substantial variations in the disease burden of HNC and its subtypes have been documented across all age groups in different regions and countries [10,11], there is a lack of global epidemiological data on HNC in middle-aged and older adults. Founded by the American Society of Clinical Oncology in 2013, the Health Equity Committee works towards cancer health equity by bolstering scientific insights and fostering international alliances [12]. Additionally, in order to achieve ‘health for all’ and reduce health disparities, the World Health Organization’s universal health coverage policy emphasizes the importance of addressing disparities in health across various regions [13]. Nevertheless, in-depth analysis of health inequalities in the burden of HNC and its subtypes at global and regional levels in middle-aged and older adults is lacking.

To close knowledge gaps and advance previous research, we utilized the most recent data from the Global Burden of Disease Study with the following objectives: (1) to update and provide a detailed assessment of the global, regional, and national burden of HNC among middle-aged and older adults, with a focus on three primary subtypes: laryngeal, nasopharyngeal, and lip/oral cavity cancers; 2) to quantify absolute and relative health inequalities across countries and the Global Burden of Disease Study (GBD) regions; and (3) to apply frontier analysis to identify countries and territories with high socio-demographic indices (SDI) where improvements in HNCs burden could potentially be achieved.

## 2. Methods

### 2.1. Data source

In this research, we performed a secondary analysis of the GBD 2021 dataset, developed by the Institute for Health Metrics and Evaluation and accessible through the following website: http://ghdx.healthdata.org/gbd-results-tool. Detailed methodologies and descriptions of the original data are available in the GBD 2021 methods appendices (https://www.healthdata.org/gbd/). For our analysis, we extracted both incidence and disability-adjusted life years (DALYs), along with their respective 95% uncertainty intervals (UIs), for three major HNCs. DALYs are a composite measure of population health that reflects the total burden of disease. They are calculated as the sum of years of life lost due to premature mortality and years lived with disability, thus capturing both fatal and non-fatal health outcomes. The extracted data were categorized by metric (number, rate), cause (larynx cancer, nasopharynx cancer, lip and oral cavity cancer), location (Global, 21 GBD regions, and 204 countries/territories), age (45–49, 50–54, 55–59… 90–94, 95+), sex (both, male, female), and year (1990–2021). Furthermore, the SDI, a key metric closely correlated with health outcomes, was used to evaluate the level of sociodemographic development. The SDI is derived from the geometric mean of three key indicators: the mean years of education for those aged 15 and above, the total fertility rate among individuals under 25 years of age, and the per capita lag-distributed income. An SDI score of 0 denotes the theoretical minimum level of development, whereas a score of 1 reflects the theoretical maximum (S1 and S2 Tables in S1 File) [14]. This research complied with the Guidelines for Accurate and Transparent Health Estimates Reporting (GATHER) [15,16], ensuring accurate and transparent health estimates reporting (S3 Table in S1 File).

Given the use of the open-source Global Burden of Disease (GBD) Study dataset in our study, obtaining ethical approval and consent to participate was not applicable.

### 2.2. Disease definitions

In this study, three major types of HNC were diagnosed based on clinical criteria established by the World Health Organization and aligned with the International Classification of Diseases, 10th Revision (ICD-10).The ICD-10 codes for HNC are larynx cancer (LC) C32-C32.9, D02.0, D14.1, D38.0; nasopharynx cancer (NPC) C11-C11.9, D10.6; lip and oral cavity cancer (LOC) C00-C08.9, D10.0-D10.5, D11-D11.9.

### 2.3. Statistical analysis

The analysis measured the disease burden using numerical values and age-standardized rates (ASR) per 100,000, which allows for valid comparisons across populations and over time by removing the impact of diverse age distributions. Furthermore, we included laryngeal cancer, nasopharyngeal cancer, and oral cavity cancer in our definition of the burden of total HNC. Since the estimates of the total HNC and its UIs have not been published, we derived the estimates and 95% UIs of the total HNC by summing the estimates and 95% UIs of the three HNCs (LC, NPC, and LOC) [17]. Additionally, the ASRs were calculated using the direct method of standardization [18], based on the following formula: $ASR\;=\;\sum_{i=1}^{N}\;\alpha_{i}\;W_{i}\;/\sum_{i=1}^{N}\;W_{i}$. Where *a*_*i*_ represents the age-specific rate in the ith age group, and *W_i_* is the population weight in the same age group of the GBD 2021 world standard population. N is the number of age groups, which is 11 in our study. Consequently, the ASRs were presented per 100,000 individuals, accompanied by 95% confidence intervals (CIs). To further investigate trends of disease burden for HNC and its subtypes in middle-aged and older adults, focusing on age-standardized incidence rate (ASIR) and age-standardized DALYs rate (ASDR) at global, regional, and national levels, this study employed Joinpoint regression analysis [18,19]. Joinpoint software (version 5.1.0.0; National Cancer Institute, Rockville, MD, US) provided a structured approach to evaluate temporal trends and test the statistical significance of variations between joinpoints. A Monte Carlo permutation method using 4,499 random permutations was employed, with Bonferroni correction applied to maintain the overall significance level. Then the average annual percentage change (AAPC) along with their corresponding 95% CIs were calculated. Notably, an increasing trend was identified if both the AAPC estimate and its lower boundary of the 95% CI were above zero; conversely, a decreasing trend was recognized if both the AAPC estimate, and its upper boundary of the 95% CI were below zero. Otherwise, the trend was considered stable.

#### 2.3.1. Health inequality analysis.

To quantify health inequalities in disease burden of ASDR for HNC and its subtypes among middle-aged and older adults globally and regionally, we utilized the Slope Index of Inequality (SII) and Concentration Index (CIX) [20]. SII measures absolute differences in disease burden between socio-demographic groups, highlighting populations at highest risk and informing targeted interventions. CIX assesses relative inequality, indicating whether the disease disproportionately affects advantaged or disadvantaged groups, thereby guiding policymakers in equity-focused resource allocation. The SII was computed by regressing national ASDR on a relative social position scale based on SDI, with the midpoint of the population ranked by SDI defining this scale [20,21]. A robust linear regression model was employed to manage heteroscedasticity [22]. Meanwhile, the CIX was derived by fitting a Lorenz concentration curve to the cumulative distribution of the SDI-ranked population and ASDR. The area under this curve was integrated to calculate the CIX, with values ranging from −1 to 1. Negative values represent a disproportionately higher disease burden in countries with lower SDI scores, whereas positive values denote a concentration of disease burden in countries with higher SDI scores [20,22]. The SII was calculated to assess the disparity in ASDR across socioeconomic contexts. It identifies differences in disease burden between the most and least advantaged regions based on SDI. Meanwhile, the CIX captured the degree of relative inequality, highlighting whether disease burden is concentrated more heavily in disadvantaged or advantaged populations. Together, these two indicators provided a comprehensive understanding of both absolute and relative health inequality in ASDR of HNC and its subtypes among middle-aged and older adults. [20,23]

#### 2.3.2. Frontier analysis.

To assess the potential for reducing ASDR of HNC and its subtypes in middle-aged and older adults for 204 countries and territories, we conducted a frontier analysis based on ASDR and SDI from 1990 to 2021. The frontier line illustrated the optimal performers, or those countries and territories with the lowest ASDR given their SDI. To highlight the unrealized potential of the ASDR in 2021, we calculated the absolute difference between the frontier ASDR and the observed ASDR, referred to as the “effective difference”. This effective difference represents the scope for improvement based on a country or territory’s available sociodemographic resources [24–26].

All analyses were executed using R statistical software (version 4.4.1), with a significance threshold set at a P-value below 0.05. The SII, CIX, and AAPC were all detailed alongside their 95% confidence intervals.

## 3. Results

### 3.1. Burden of overall HNC in middle-aged and older adults

**Fig 1:**
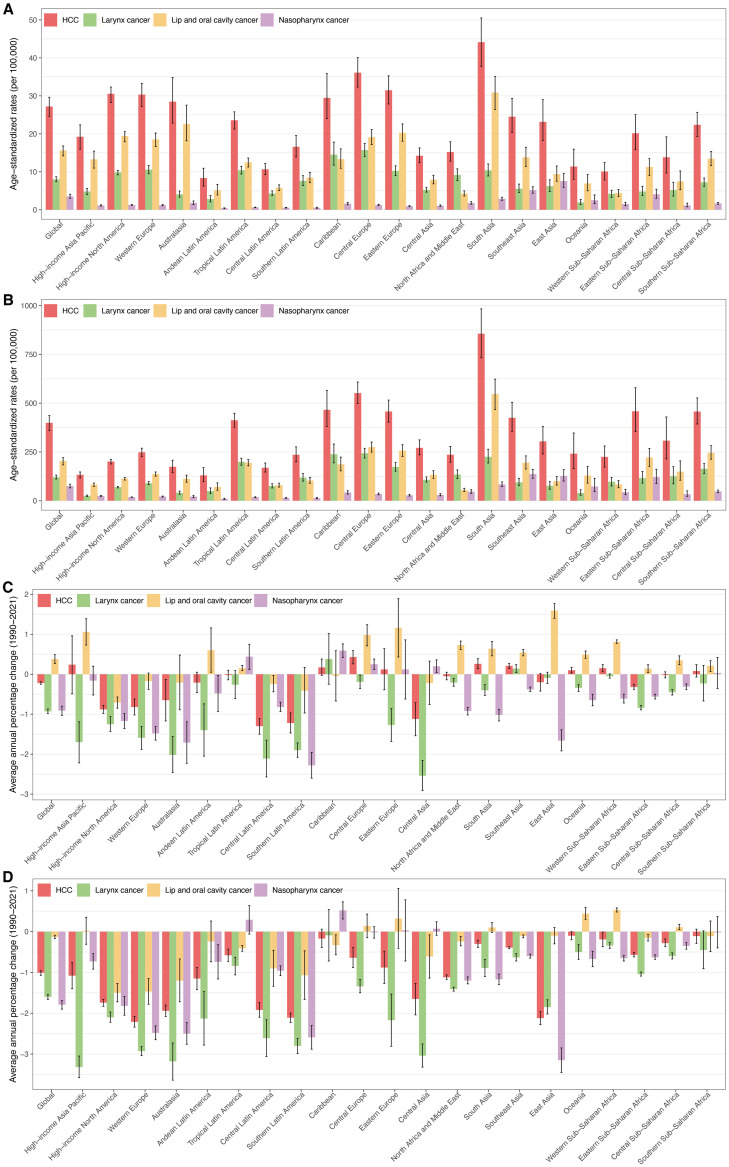
Age-standardised incidence and DALY rates in 2021, and their average annual percentage changes for HNC and its subtypes in middle-aged and older adults at global and 21 GBD level from 1990 to 2021. **(A)** ASIR in 2021. **(B)** ASDR in 2021. **(C)** AAPC of ASIR from 1990 to 2021. **(D)** AAPC of ASDR from 1990 to 2021. ASIR, age-standardised incidence rate. ASDR, age-standardised DALY rate. DALYs, disability-adjusted life years. HNC, head and neck cancer. AAPC, average annual percentage change.

The global incidence of overall HNC among middle-aged and older adults was 320,615 cases in 1990 and increased to 650,205 cases in 2021. The global ASIR for 1990 and 2021 were 29.29 per 100,000 and 27.19 per 100,000, respectively (Table 1). Global DALYs increased from 6,099,884 in 1990 to 9,621,610 in 2021. The global ASDR declined from 543.39 per 100,000 to 398.94 per 100,000 in 2021(Table 2). From 1990 to 2021, the global ASIR and ASDR both decreased, with AAPC values of −0.22, −1.01, respectively (Fig 1).

**Table 1:** Burden (incidence) of HNC in middle-aged and older adults, and their temporal trends from 1990 to 2021, using Joinpoint analysis.

Location	Number of cases, (95% CI) 1990	Age-standardised rate (per 100,000) 1990	Number of cases, (95% CI) 2021	Age-standardised rate (per 100,000) 2021	Annual average percent change (95% CI)	P
Global	320615(299926-341279)	29.29(27.33-31.18)	650205(589726-708525)	27.19(24.63-29.63)	−0.22(−0.25 to −0.19)	<0.0001
Larynx cancer	117909(111050-124774)	10.70(10.06-11.33)	192593(177458-208729)	8.02(7.38-8.69)	−0.93(−0.98 to −0.87)	<0.0001
Nasopharynx cancer	52454(46607-58419)	4.68(4.16-5.21)	85245(73448-99102)	3.53(3.04-4.11)	−0.91(−1.03 to −0.80)	<0.0001
Lip and oral cavity cancer	150252(142269-158086)	13.91(13.12-14.65)	372367(338821-400693)	15.64(14.20-16.83)	0.38(0.27 to 0.49)	<0.0001
High-income Asia Pacific	10291(9160-11459)	18.44(16.38-20.54)	24381(19948-28450)	19.23(15.99-22.37)	0.24(−0.49 to 0.96)	0.5241
High-income North America	36758(34626-38515)	40.12(37.87-42.01)	54089(49857-57314)	30.55(28.26-32.32)	−0.88(−0.98 to −0.78)	<0.0001
Western Europe	57393(52699-62396)	38.63(35.44-42.07)	70713(62972-77784)	30.32(27.23-33.30)	−0.82(−1.02 to −0.62)	<0.0001
Australasia	2165(1810-2587)	34.38(28.68-41.13)	4016(3218-4913)	28.47(22.84-34.84)	−0.65(−1.17 to −0.13)	0.0139
Andean Latin America	494(402-606)	9.22(7.50-11.30)	1332(1000-1751)	8.34(6.27-10.96)	−0.21(−0.46 to 0.05)	0.1112
Tropical Latin America	6030(5556-6529)	23.9(21.94-25.89)	16858(15238-18445)	23.56(21.27-25.79)	−0.02(−0.14 to 0.10)	0.7041
Central Latin America	3200(3006-3386)	14.92(13.97-15.80)	7253(6289-8293)	10.66(9.24-12.17)	−1.30(−1.50 to −1.11)	<0.0001
Southern Latin America	3209(2719-3754)	25.21(21.35-29.50)	3923(3292-4626)	16.59(13.92-19.56)	−1.22(−1.47 to −0.97)	<0.0001
Caribbean	1980(1737-2257)	28.32(24.83-32.28)	4382(3568-5331)	29.47(24-35.83)	0.17(−0.03 to 0.38)	0.1011
Central Europe	13195(12292-14159)	32.01(29.77-34.37)	20412(18276-22616)	36.12(32.32-40.08)	0.43(0.26 to 0.59)	<0.0001
Eastern Europe	24843(23306-26797)	31.66(29.67-34.20)	29451(26095-33004)	31.46(27.86-35.28)	0.12(−0.39 to 0.64)	0.6463
Central Asia	2784(2562-3023)	20.90(19.17-22.75)	3316(2884-3805)	14.23(12.39-16.30)	−1.12(−1.54 to −0.71)	<0.0001
North Africa and Middle East	7095(5804-8615)	15.60(12.72-19.03)	18764(15767-22121)	15.22(12.77-17.92)	−0.05(−0.14 to 0.05)	0.332
South Asia	66443(57259-76579)	41.28(35.40-47.65)	181013(155070-207117)	44.14(37.81-50.50)	0.26(0.14 to 0.39)	<0.0001
Southeast Asia	15654(13270-18297)	22.72(19.25-26.56)	44646(37079-53427)	24.51(20.34-29.31)	0.21(0.14 to 0.27)	<0.0001
East Asia	59625(49452-70133)	24.73(20.55-29.02)	144689(113819-181732)	23.15(18.24-29)	−0.20(−0.42 to 0.02)	0.0769
Oceania	89(62-125)	11.18(7.87-15.47)	236(163-336)	11.37(8.01-15.97)	0.10(0.03 to 0.17)	0.008
Western Sub-Saharan Africa	2298(1810-2836)	9.47(7.50-11.65)	5501(4201-6864)	10.09(7.85-12.45)	0.15(0.06 to 0.24)	0.0008
Eastern Sub-Saharan Africa	4562(3694-5491)	21.91(17.78-26.32)	9475(7363-11905)	20.16(15.85-25.07)	−0.32(−0.38 to −0.25)	<0.0001
Central Sub-Saharan Africa	859(592-1207)	14.00(9.69-19.62)	2104(1473-2909)	13.83(9.67-19.21)	−0.02(−0.09 to 0.06)	0.6485
Southern Sub-Saharan Africa	1647(1256-2096)	22.05(16.81-28.09)	3652(3146-4195)	22.37(19.29-25.66)	0.08(−0.07 to 0.24)	0.2957

**Table 2:** Burden (DALYs) of HNC in middle-aged and older adults, and their temporal trends from 1990 to 2021, using Joinpoint analysis.

location	Number of cases, (95% CI) 1990	Age-standardised rate (per 100,000) 1990	Number of cases, (95% CI) 2021	Age-standardised rate (per 100,000) 2021	Annual average percent change (95% CI)	P
Global	6099884(5643981-6573812)	543.39(502.4-585.57)	9621610(8687183-10533454)	398.94(359.97-436.74)	−1.01(−1.07 to −0.96)	<0.0001
Larynx cancer	2238987(2088364-2393272)	199.51(185.93-213.31)	2909562(2680707-3165879)	120.48(110.95-131.09)	−1.60(−1.66 to −1.54)	<0.0001
Nasopharynx cancer	1480051(1311369-1648731)	130.06(115.26-144.85)	1813608(1592547-2056015)	74.86(65.72-84.86)	−1.79(−1.90 to −1.69)	<0.0001
Lip and oral cavity cancer	2380847(2244248-2531810)	213.82(201.21-227.41)	4898441(4413928-5311561)	203.60(183.29-220.79)	−0.13(−0.17 to −0.09)	<0.0001
High-income Asia Pacific	103385(93073-113688)	183.27(164.74-201.52)	158722(136610-176512)	132.74(116.50-147.23)	−1.08(−1.40 to −0.76)	<0.0001
High-income North America	304259(289723-316730)	340.91(325.20-354.67)	347660(324568-367031)	200.81(188.16-211.69)	−1.74(−1.83 to −1.66)	<0.0001
Western Europe	705927(655013-758263)	487.40(451.88-524.29)	559250(505228-608614)	248.01(225.87-269.37)	−2.21(−2.34 to −2.08)	<0.0001
Australasia	19612(16738-22865)	314.48(268.13-366.79)	24030(19874-28698)	173.79(143.89-207.6)	−1.94(−2.08 to −1.80)	<0.0001
Andean Latin America	10477(8534-12831)	188.56(153.61-230.91)	21028(15764-27496)	129.87(97.42-169.66)	−1.15(−1.42 to −0.88)	<0.0001
Tropical Latin America	130990(121105-141556)	498.50(460.02-538.66)	297903(272309-324005)	412.27(376.50-448.48)	−0.58(−0.73 to −0.43)	<0.0001
Central Latin America	64281(60643-67888)	287.02(270.20-303.21)	117016(101707-134043)	168.82(146.77-193.22)	−1.92(−2.10 to −1.74)	<0.0001
Southern Latin America	60955(51983-71011)	477.20(406.74-556.07)	54932(46584-64459)	235.05(199.36-275.93)	−2.11(−2.23 to −2.00)	<0.0001
Caribbean	35277(31005-40405)	498.89(438.38-571.36)	69440(56742-84227)	465.88(380.79-565.02)	−0.17(−0.39 to 0.06)	0.1457
Central Europe	272530(254701-291471)	661.51(617.59-707.85)	301790(272725-332433)	551.62(498.09-608.49)	−0.64(−0.88 to −0.39)	<0.0001
Eastern Europe	489088(459998-526164)	622.99(585.36-671.51)	421035(371232-474541)	457.08(402.55-515.94)	−0.88(−1.27 to −0.48)	<0.0001
Central Asia	63939(59207-69130)	467.79(432.33-506.74)	66138(57427-76348)	270.78(235.4-311.92)	−1.65(−2.04 to −1.27)	<0.0001
North Africa and Middle East	160995(131507-195456)	333.65(272.05-406.29)	307594(258112-363872)	235.53(197.73-277.88)	−1.12(−1.17 to −1.06)	<0.0001
South Asia	1625372(1399762-1880956)	949.59(815.00-1099.82)	3637241(3110462-4177947)	855.87(732.35-982.85)	−0.30(−0.39 to −0.21)	<0.0001
Southeast Asia	351938(297923-412977)	480.13(406.64-563.01)	815292(681899-968600)	424.85(355.48-504.14)	−0.39(−0.42 to −0.37)	<0.0001
East Asia	1460792(1204983-1725621)	577.43(477.20-680.78)	1924344(1513817-2416777)	303.91(239.34-381)	−2.12(−2.28 to −1.96)	<0.0001
Oceania	2176(1469-3127)	248.95(170.30-354.05)	5500(3695-8014)	241.04(164.65-346.96)	−0.10(−0.20 to 0.00)	0.0475
Western Sub-Saharan Africa	59276(46448-73538)	232.31(182.71-287.33)	132199(99825-166577)	224.63(172.40-280.50)	−0.19(−0.37 to −0.01)	0.0408
Eastern Sub-Saharan Africa	119157(95887-144250)	538.49(434.55-650.90)	231455(176782-295554)	457.94(353.71-579.06)	−0.57(−0.62 to −0.51)	<0.0001
Central Sub-Saharan Africa	22168(15131-31449)	332.17(227.93-469.18)	51337(35629-71659)	307.73(214.06-429.21)	−0.28(−0.37 to −0.18)	<0.0001
Southern Sub-Saharan Africa	37289(28698-47417)	480.88(369.53-612.20)	77706(66758-89806)	456.88(393.21-526.94)	−0.11(−0.29 to 0.06)	0.1982

Throughout the 21 GBD regions, South Asia was identified as having the highest ASIR (44.14 per 100,000) and ASDR (855.87 per 100,000) in 2021. In stark contrast, Andean Latin America demonstrated the lowest rates, with 8.34 and 129.87 per 100,000 for ASIR and ASDR, respectively. From 1990 to 2021, Central Europe experienced the steepest annual growth in ASIR, with an AAPC value of 0.43. Central Latin America exhibited the greatest decrease in ASIR, with an AAPC of −1.30. In addition, ASDR showed a downward trend across all 21 GBD regions from 1990 to 2021, notably with the steepest drop in Western Europe (AAPC = −2.21) (Fig 1; Tables 1 and 2).

**Fig 2:**
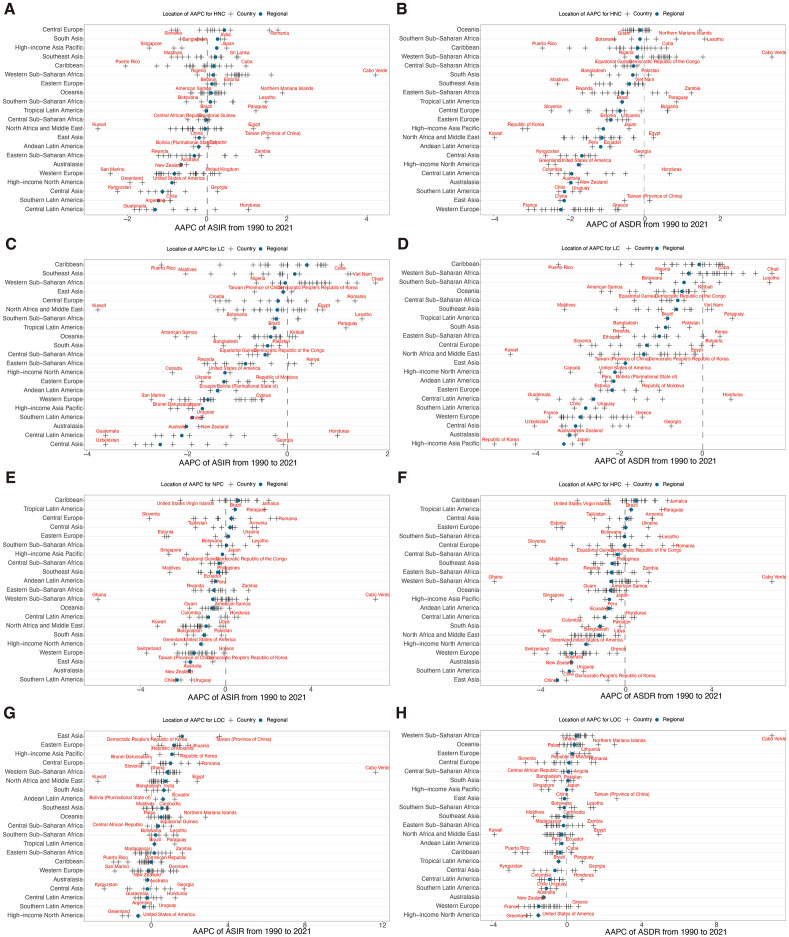
Age-standardised incidence and DALY rates for HNC and its subtypes among middle-aged and older adults at national and GBD regional levels in 2021. **(A)** ASIR of HNC. **(B)** ASDR of HNC. **(C)** ASIR of larynx cancer. **(D)** ASDR of larynx cancer. **(E)** ASIR of nasopharynx cancer. **(F)** ASDR of nasopharynx cancer. **(G)** ASIR of lip and oral cavity cancer. **(H)**, ASDR of lip and oral cavity cancer. ASIR, age-standardised incidence rate. ASDR, age-standardised DALY rate. DALYs, disability-adjusted life years. HNC, head and neck cancer. The black cross represents the ASR for each country, the blue solid dot represents the ASR for GBD regions, and the red text denotes the countries with the maximum and minimum ASR within each GBD region.

Among 204 countries and territories, Pakistan reported the highest ASIR at 83.53 per 100,000 in 2021, while Seychelles had the highest ASDR at 968.08 per 100,000. Notably, the most rapid increases of ASIR and ASDR were both located in Cabo Verde from 1990–2021, with AAPC values of 4.21, and 3.39, respectively. Furthermore, Kuwait exhibited the largest decrease in ASIR (AAPC = −2.37) and ASDR (−3.97) (Fig 2, S4 Table and S1 Fig in S1 File).

### 3.2. Burden of three HNC subtypes in middle-aged and older adults

**Fig 3:**
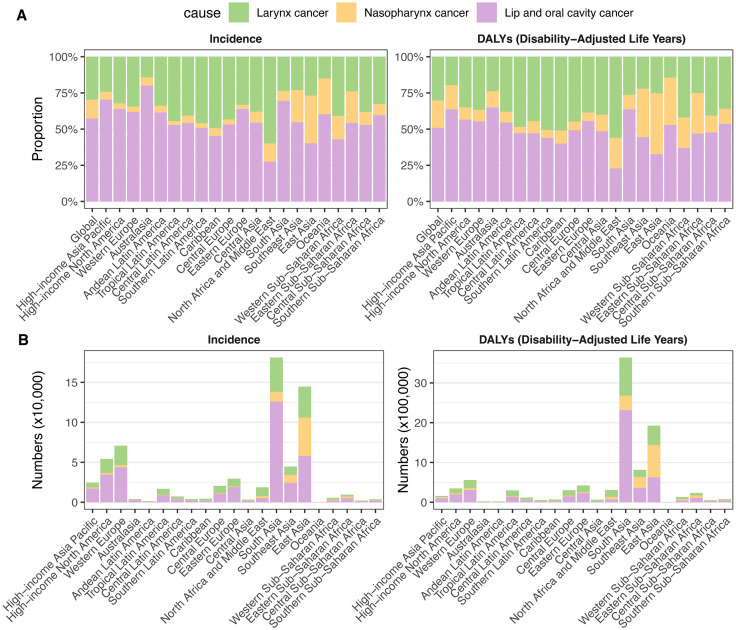
Proportion and numbers of incident cases and DALYs in 2021 by 21 GBD regions, for larynx cancer, nasopharynx cancer, lip and oral cavity cancer in middle-aged and older adults. **(A)**, Proportions of incident cases and DALYs accounted for each cancer. **(B)**, Numbers of incident cases and DALYs for each cancer. DALY, disability-adjusted life-years.

In 2021, the global new case counts for LC, NPC, and LOC in middle-aged and older adults were 192,593, 85,245, and 372,367, respectively. Their corresponding ASIR per 100,000 population were 8.02, 3.53, and 15.64. The global DALYs for LC, NPC, and LOC were 2,909,562, 1,813,608, and 4,898,441, respectively, with ASDR of 120.48, 74.86, and 203.60 per 100,000. LOC ranked first in the global distribution of incidence cases (57.27%) and DALYs (50.91%) for total HNC globally, surpassing LC (19.26% and 30.24%) and NPC (13.11% and 18.85%). Between 1990 and 2021, ASIR significantly increased for LOC (AAPC: 0.38), while LC (AAPC: −0.93) and NPC (AAPC: −0.91) saw declines. Meanwhile, global ASDR decreased across all three subtype cancers in middle-aged and older adults, most notably for LC (AAPC: −1.60) and NPC (AAPC: −1.79). (Tables 1 and 2, Figs 1 and 3).

In 2021, the highest ASIR among middle-aged and older adults were observed in Western Europe for LC (10.58 per 100,000), East Asia for NPC (7.56 per 100,000), and South Asia for LOC (30.87). In terms of ASDR, the highest were seen in Central Europe for LC (243.02 per 100,000), Southeast Asia for NPC (136.06 per 100,000), and Central Europe for LOC (274.20 per 100,000). From 1990 to 2021, there were general declines in ASIR and ASDR for both LC and NPC in middle-aged and older adults, with Central Asia (AAPC for ASIR = −2.54) and Australasia (AAPC for ASDR = −3.18) leading for LC, and Southern Latin America (AAPC for ASIR = −2.28) and East Asia (AAPC for ASDR = −3.15) for NPC. Meanwhile, the largest increases in LOC were seen in East Asia (ASIR: 1.59) and Western Sub-Saharan Africa (ASDR: 0.59), while High-income North America showed the most substantial declines both of ASIR (−0.71) and ASDR (−1.50). (Fig 1, and S5 Table in S1 File).

Nationally, the highest ASIRs among middle-aged and older adults were reported in Monaco for LC (35.85), Malaysia for NPC (15.75), and Palau for LOC (76.27) in 2021. The corresponding highest ASDR were recorded in Montenegro (434.43), Malaysia (415.76), and Pakistan (1204.90) (Fig 2, and S6-8 Tables in S1 File). The most significant increases in ASIR and ASDR occurred in Chad for LC (AAPC: 1.75 and 1.65), Cabo Verde for NPC (AAPC: 7.00 and 6.84) and LOC (AAPC: 11.65 and 11.01). The steepest declines in ASIR were observed in Kuwait for LC (−3.78), Ghana for NPC (−5.98), and Kuwait for LOC (−2.79). Declines in ASDR were largest in Republic of Korea for LC (−4.98), Ghana for NPC (−6.08), and Kuwait for LOC (−3.85) (S6-8 Tables, and S1 Fig in S1 File).

### 3.3. Health inequality and frontier analysis

#### 3.3.1. Overall HNC.

**Fig 4:**
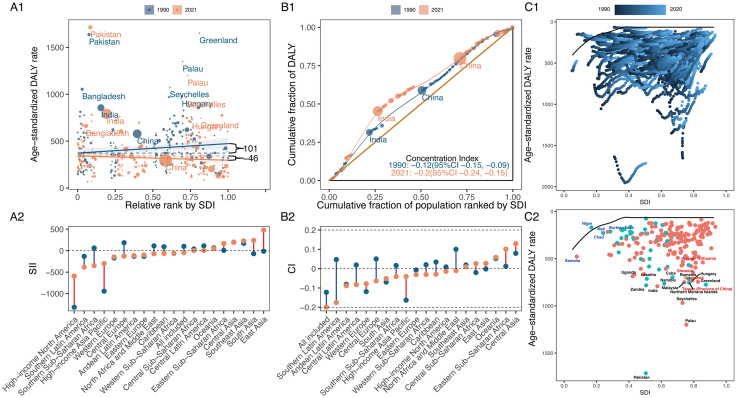
Absolute and relative cross-country inequalities and frontier analysis results for overall HNC of ASDR in middle-aged and older adults from 1990 to 2021. **(A)** Health inequality regression curves (A1) and ranking graphs by values of SII in 2021(A2). **(B)** Concentration curves (B1) and ranking graphs by values of CIX in 2021 (B2). **(C)** Results of frontier analysis from 1990 to 2021 (C1) and in 2021 (C2). HNC, head and neck cancer. ASDR, age-standardised DALY rate. DALYs, disability-adjusted life years. SDI, socio-demographic index. SII, slope index of inequality. CIX, concentration index.

In 1990, higher SDI countries bore the greater global ASDR burden for HNC among middle-aged and older adults. By 2021, this burden had shifted to lower SDI countries, as indicated by a decrease in the SII for ASDR from 100.54 (95% CI: 10.65, 190.43) to −45.97 (95% CI: −113.84, 21.91). Meanwhile, lower SDI countries experienced worsening health inequalities in HNC ASDR, as indicated by the CIX moving from −0.1223 (95% CI: −0.0903, −0.1542) to −0.1991 (95% CI: −0.1543, −0.2438). Even with a notable progress in reducing inequality among lower SDI countries in SII from 1990 to 2021, High-income North America still had the highest negative SII (−1318.20) in the GBD regions by 2021. Furthermore, Southern Latin America showed the greatest deterioration in health inequality, with a CIX of −0.1757 (95% CI: −0.1337, −0.2438) being the lowest in 2021 (Fig 4, and S9 Table in S1 File). Between 1990 and 2021, global SII and CIX trends highlighted a growing concentration of disease burden for HNC ASDR in lower SDI countries, with marked heterogeneity observed across the 21 GBD regions (S2-4 Figs in S1 File). In most GBD regions and years, countries with lower SDI experienced a disproportionately higher disease burden. However, this trend was not uniform across all GBD regions. Specifically, in certain Asian regions, including Central, East, and Southeast Asia, a substantial portion of the disease burden was concentrated in higher SDI countries.

To explore the potential for reducing ASDR of HNC among middle-aged and older adults in relation to national development, we conducted a frontier analysis of ASDR and SDI from 1990 to 2021. Among the top 15 countries and territories (black labels) with the most significant effective difference from the frontier (range: 1649.61–632.59), Pakistan, Palau, Seychelles, India, Zambia, Taiwan (Province of China), Namibia, Northern Mariana Islands, Malaysia, Greenland, Hungary, Romania, Lesotho, Cuba, and Uganda were included (Fig 4, and S10 Table in S1 File). These countries and territories had disproportionately high HNC ASDR for their sociodemographic resources. As shown in Fig 4, Somalia, Niger, Burkina Faso, Mali, and Chad, all with low SDI (<0.4658) and smaller effective differences, were identified by blue labels, while red labels indicated high SDI countries and territories (SDI > 0.8103) that exhibit a high effective difference relative to their development level, such as Taiwan (Province of China), Greenland, Slovakia, Poland, Lithuania.

#### 3.3.2. Larynx cancer.

**Fig 5:**
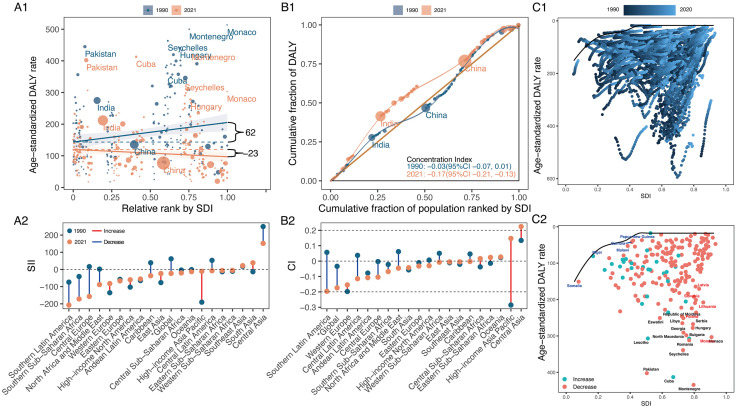
Absolute and relative cross-country inequalities and frontier analysis results for larynx cancer of ASDR in middle-aged and older adults from 1990 to 2021. **(A)** Health inequality regression curves (A1) and ranking graphs by values of SII in 2021(A2). **(B)** Concentration curves (B1) and ranking graphs by values of CIX in 2021 (B2). **(C)** Results of frontier analysis from 1990 to 2021 (C1) and in 2021 (C2). ASDR, age-standardised DALY rate. DALYs, disability-adjusted life years. SDI, socio-demographic index. SII, slope index of inequality. CIX, concentration index.

Over the period from 1990 to 2021, the SII trend revealed that the global ASDR burden of LC in middle-aged and older adults transitioned from higher to lower SDI countries, with SII values declining from 61.85 (95% CI: 14.92, 108.78) in 1990 to −23.12 (95% CI: −49.19, 2.96) in 2021 (Fig 5, and S11 Table in S1 File). At the same time, the CIX for LC ASDR indicated a growing health inequality in lower SDI countries, as values decreased from −0.0338 (95% CI: −0.0737, 0.0062) to −0.1739 (95% CI: −0.2141, −0.1336). Trends in SII across GBD regions highlighted that lower SDI countries in Central Europe, Southern Latin America, and Southern Sub-Saharan Africa experienced the greatest increases in health inequities. Conversely, High-income Asia Pacific experienced the largest improvement. With the most negative SII (−207.16 [95% CI: −230.92, −183.41]) and CIX (−0.1962 [95% CI: −0.2211, −0.1713]) in 2021, Southern Latin America was at the opposite end of the spectrum from Central Asia, which had the most positive SII (151.68 [95% CI: 48.71, 254.65]) and CIX (0.2246 [95% CI: 0.0565, 0.3927]).

Based on ASDR of LC in middle-aged and older adults and SDI, Fig 5 presented a frontier analysis showing the top 15 countries and territories with the greatest effective differences from the frontier (range: 416.90–233.17). These countries and territories, marked in black, included the Montenegro, Cuba, Pakistan, Seychelles, Romania, Bulgaria, Lesotho, Monaco, North Macedonia, Georgia, Hungary, Serbia, Libya, Republic of Moldova, Eswatini. Furthermore, low SDI countries and territories with minor effective differences, like Somalia, Gambia, Niger, Malawi, and Papua New Guinea, were identified by blue labels. Conversely, countries and territories with high SDI and substantial effective differences, such as Monaco, Poland, Lithuania, Slovakia, and Latvia, were marked by red labels (Fig 5, and S12 Table in S1 File).

#### 3.3.3. Nasopharynx cancer.

**Fig 6:**
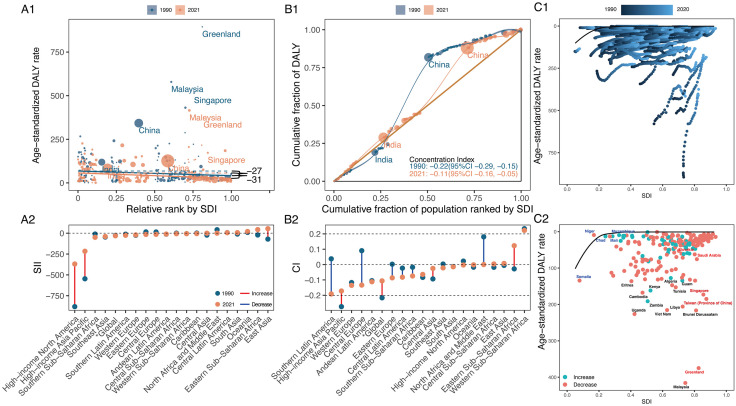
Absolute and relative cross-country inequalities and frontier analysis results for nasopharynx of ASDR in middle-aged and older adults from 1990 to 2021. **(A)** Health inequality regression curves (A1) and ranking graphs by values of SII in 2021(A2). **(B)** Concentration curves (B1) and ranking graphs by values of CIX in 2021 (B2). **(C)** Results of frontier analysis from 1990 to 2021 (C1) and in 2021 (C2). ASDR, age-standardised DALY rate. DALYs, disability-adjusted life years. SDI, socio-demographic index. SII, slope index of inequality. CIX, concentration index.

The global analysis of NPC ASDR among middle-aged and older adults revealed marked SDI-related health inequalities, with lower SDI countries facing a disproportionate share of the burden. Over the 1990−2021 period, the SII for NPC ASDR indicated increasing health inequalities in lower SDI countries, declining from −26.64 (95% CI: −48.99, −4.29) to −30.89 (95% CI: −46.62, −15.16) (Fig 6, and S13 Table in S1 File). During the same period, the CIX improved slightly, rising from –0.22 (95%CI: –0.29, –0.15) to – 0.11 (95%CI: –0.16, –0.05). Regionally, High-income North America and High-income Asia Pacific demonstrated significant improvements in health inequality among countries with lower SDI in SII. High-income North America reported the most negative SII in 2021 (- 369.49 [95%CI: – 819.85, 80.87]), closely followed by High-income Asia Pacific (- 216.59 [95%CI: –388.67, –44.51]). On the contrary, East Asia exhibited the highest positive SII (54.20 [95%CI: –107.21, 215.60]). Furthermore, Southern Latin America’s CIX showed a marked decrease to −0.19 (95% CI: −0.27, −0.12), in contrast to Western Sub-Saharan Africa, which had the highest index at 0.22 (95% CI: 0.06, 0.39) in 2021.

Fig 6 highlighted the frontier analysis results, illustrating the ASDR of NPC and SDI in middle-aged and older adults. The 15 countries and territories with the largest effective difference from the frontier (range: 414.97–133.65) were the Malaysia, Greenland, Uganda, Brunei Darussalam, Viet Nam, Libya, Zambia, Taiwan (Province of China), Singapore, Cambodia, Kenya, Tunisia, Algeria, Eritrea, Guam. The low SDI countries and territories with low effective differences, such as Somalia, Niger, Chad, Mali, Mozambique, were marked in blue. Meanwhile, red labels denoted high SDI countries and territories that exhibited significant performance relative to their development status, including United Greenland, Taiwan (Province of China), Singapore, Saudi Arabia, Slovakia (Fig 6, and S14 Table in S1 File).

#### 3.3.4. Lip and oral cavity cancer.

**Fig 7:**
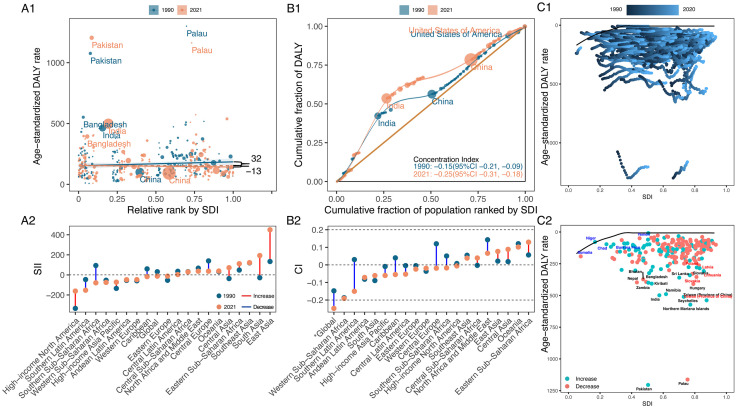
Absolute and relative cross-country inequalities and frontier analysis results for lip and oral cavity cancer of ASDR in middle-aged and older adults from 1990 to 2021. **(A)** Health inequality regression curves (A1) and ranking graphs by values of SII in 2021(A2). **(B)** Concentration curves (B1) and ranking graphs by values of CIX in 2021 (B2). **(C)** Results of frontier analysis from 1990 to 2021 (C1) and in 2021 (C2). ASDR, age-standardised DALY rate. DALYs, disability-adjusted life years. SDI, socio-demographic index. SII, slope index of inequality. CIX, concentration index.

Globally, the ASDR burden of LOC among middle-aged and older adults shifted toward lower SDI countries, as indicated by the change in SII from 31.64 (95%CI: –10.43, 73.71) to –12.77 (95%CI: –47.32, 21.79). Additionally, the CIX for LOC ASDR declined from −0.15 (95% CI: −0.21, −0.09) to −0.25 (95% CI: −0.31, −0.18), indicating worsening inequalities in lower SDI countries (Fig 7, and S15 Table in S1 File). Regionally, High-income North America saw the most substantial improvement in inequality among lower SDI countries, though it still presented the most negative SII (- 161.42 [95%CI: – 342.16, 19.32]). Conversely. East Asia the steepest increase in inequality among countries with higher SDI, with the largest positive SII (450.02 [95%CI: −684.85, 1584.88]). In 2021, Western Sub-Saharan Africa saw the most negative CIX at −0.19 (95% CI: −0.31, −0.08), and Eastern Sub-Saharan Africa had the highest positive index at 0.13 (95% CI: 0.07, 0.19).

Fig 7 displayed frontier analysis results based on ASDR of LOC in middle-aged and older adults by SDI. The top 15 countries and territories with the highest effective differences (range: 1197.46–346.65) were Pakistan, Palau, Northern Mariana Islands, Taiwan (Province of China), Seychelles, India, Namibia, Hungary, Kiribati, Zambia, Nepal, Bangladesh, Bhutan, Sri Lanka, Slovakia. Low SDI countries and territories with minimal effective differences, such as Nepal, Uganda, Guinea, Rwanda, Eritrea, were represented in blue, while those with high SDI and notable effective difference considering their development level, such as Taiwan (Province of China), Slovakia, Lithuania, Latvia, Poland, were marked in red (Fig 7, and S16 Table in S1 File).

## 4. Discussion

This research marks the first effort, to our awareness, to quantify health inequalities in the burden of HNC and its subtypes across countries and GBD regions in middle-aged and older adults through a secondary analysis of GBD 2021 data. At the same time, it identified health improvements that could be realized within the current stage of development for 204 countries and territories, using frontier analysis. Despite a decrease in the ASIR and ASDR, there was a notable rise in global incident cases and DALYs for HNC in middle-aged and older adults from 1990–2021. In 2021, the SII reached its peak negativity in High-income North America, while Southern Latin America experienced the most negative CIX. Of the three subtypes, lower SDI countries experienced worsening inequalities in the SII for NPC and in the CIX for LC and LOC. On the other hand, NPC showed improving health inequalities in the CIX. Additionally, the SII for LC and LOC reflected a shift toward a greater burden in countries with lower SDI between 1990 and 2021. According to the frontier analysis, the Netherlands, Taiwan (Province of China), Greenland, Slovakia, Poland, and Lithuania could reduce HNC-related ASDR by utilizing their socio-demographic strengths.

In accordance with previous studies [10,27], our analysis demonstrated a substantial rise in both global incidence and DALYs of HNC between 1990 and 2021. This upward trend was probably attributable to the combined impact of traditional risk factors, including smoking, alcohol, betel quid consumption, EBV infection, and HPV infection [28,29]. alongside demographic shifts including population growth and accelerated aging. For example, nicotine not only promotes the development of HNC but also activates the CHRNA5 receptor. This activation regulates CES1 expression through the MEK/ERK signaling pathway, thereby contributing to the recurrence and metastasis of head and neck squamous cell carcinoma [30]. In contrast, our analysis identified a notable decline in both ASIR and ASDR in the middle-aged and elderly populations, with AAPC values of −0.22 and −1.01, respectively. However, a previously conducted study reported that the ASIR for all age groups in HNC increased from 12.49 (95% UI: 11.83–13.19) in 1990 to 13.97 (95% UI: 12.71–15.19) in 2021, with an estimated annual percentage change (EAPC) of 0.35 (95% CI: 0.31–0.39) [10]. The discrepancy might arise from their inclusion of thyroid cancer in the head and neck tumor classification by anatomical region. It was well documented that thyroid cancer has experienced a substantial increase in ASIR, rising from 2.01 (95% UI: 1.9–2.12) in 1990 to 2.83 (95% UI: 2.56–3.06) in 2021, with an EAPC of 1.25 (95% CI: 1.12–1.37) [31], largely driven by the expanded application of diagnostic imaging and fine-needle aspiration biopsy [32].

Our findings highlighted notable geographical disparities in the burden of HNC among middle-aged and older adults across the 21 GBD regions and 204 countries or territories. Notably, Central Europe exhibited the most rapid annual increase of ASIR in 21 GBD regions from 1990 to 2021, potentially due to an aging population. Over the same period, Western Europe experienced the most significant reduction in ASDR, possibly attributable to its robust healthcare systems and effective public health strategies for enhancing HNC prevention, early diagnosis, and therapeutics. The observed contrast in ASRs between South Asia and Andean Latin America, suggested that region-specific factors, such as genetic susceptibility and environmental influences, might be contributing to the observed variations. Separate analysis of the three HNC subtypes among middle-aged and older adults revealed that LOC contributed to over half of the cases and DALYs in 2021. Furthermore, from 1990 to 2021, LOC demonstrated increasing age-standardized incidence and DALYs rates across more GBD regions compared to LC and NPC, which generally experienced declines in these rates over the same period. This finding highlights the critical need for reinforcing targeted prevention measures and early screening programs in regions experiencing a rise in ASIR and ASDR of HNCs, especially lip and oral cavity cancer. Effective public health interventions may include the tobacco cessation, and reducing alcohol misuse, along with regular oral health screenings and targeted vaccination programs, such as HPV vaccination. Such efforts could help reduce the disease burden of these cancers.

The SII and the CIX revealed significant disparities in health inequality patterns across cancers, time points, and geographical locations (19 GBD regions and 204 countries or territories). Globally, our findings indicated that lower SDI regions and countries bore a disproportionately higher burden of HNC and its subtypes in middle-aged and older adults. Existing studies indicate that higher SDI regions are equipped with more comprehensive social safety systems and healthcare resources, enabling superior prevention, diagnosis, treatment, and rehabilitation [33–36]. Notably, the CIX for LC and LOC demonstrated that health inequalities worsened from 1990 to 2021, with values nearing or surpassing 0.2, a threshold defined as a moderate-high level of relative inequality. To eliminate these disparities, targeted efforts must be undertaken, including the following: First, developing national cancer control programs aimed at the prevention and mitigation of risk factors, including tobacco control initiatives, widespread HPV vaccination, and the encouragement of healthy diets, physical activity, and obesity management [29,37,38]. Second, ensuring equitable distribution of medical resources by establishing healthcare infrastructure, including oncology clinics, diagnostic labs for biochemistry and pathology, and diagnostic imaging services [36]. Third, enhancing the expertise of healthcare professionals in oncology, strengthening international medical collaborations [39], and introducing advanced medical technologies. Finally, promoting economic development in lower SDI countries, improving cancer care financing mechanisms, and creating sustainable business models for cancer treatment services [40]. Moreover, further research is essential to uncover the underlying drivers of these inequalities, which will provide critical insights for the design of effective and equitable public health strategies [33,41].

To gain deeper insights into the potential improvements in ASDR for HNC and its subtypes among middle-aged and older adults within the context of national development, we employed frontier analysis with data on ASDR and SDI spanning 1990–2021. Remarkably, countries such as Somalia, Niger, Burkina Faso, Mali, and Chad, despite having low SDIs, were estimated to have some of the lowest ASDR for HNC, suggesting that health outcomes in resource-constrained environments could be optimized. Nonetheless, these findings should be interpreted with caution, as they may partly reflect incomplete disease reporting and limitations in health surveillance systems rather than true health advantages. This highlights both the potential for improving outcomes in low-resource settings and the need to account for data limitations when assessing disease burden. Conversely, countries and territories with high SDIs, including Taiwan (Province of China), Greenland, Slovakia, Poland, and Lithuania, displayed a considerable gap from the frontier, underscoring the need for increased investment in HNC health resources to mitigate the persistent burden on HNC among middle-aged and older adults. For LC – related ASDR, the frontier analysis revealed that high SDI regions, such as Monaco, Poland, Lithuania, Slovakia, and Latvia, exhibited considerable gaps from the frontier. Similarly, for NPC, notable gaps were observed in the following five high SDI regions: Greenland, Taiwan (Province of China), Singapore, Saudi Arabia, and Slovakia. In the analysis of LOC, five high SDI regions, including Taiwan (Province of China), Slovakia, Lithuania, Latvia, and Poland, also displayed significant differences from the frontier. Given the substantial social resources available in these regions, several targeted strategies could be implemented to mitigate the cancer burden in these regions. First, high SDI countries and territories have the capacity to implement sophisticated cancer screening initiatives, particularly targeted toward high-risk groups, to enable timely diagnosis and intervention [36]. One recent study has identified integrins ITGA3, ITGA5, and ITGA6 as potential diagnostic and prognostic biomarkers in HNC, with higher expression levels associated with poorer survival. Incorporating such molecular markers into clinical practice might further refine risk stratification and facilitate earlier therapeutic decisions, thereby improving survival outcomes [42]. Additionally, enhancing access to advanced oncology services, such as radiotherapy and immunotherapy, which have been shown to significantly improve survival rates in HNCs [28,43–45]. For example, immune microenvironment modulation, such as the inhibition of CCR5 + T cell accumulation in the tumor microenvironment, can improve the efficacy of anti-TGF-β/PD-L1 bispecific antibody therapy. This provides a potential strategy to reduce the burden of head and neck cancer in high SDI countries [46]. Equally important is the need for robust public health programs to address lifestyle factors, including smoking cessation, alcohol reduction, and HPV vaccination, which are known contributors to the HNC burden. By integrating these efforts, high SDI countries and territories may make substantial progress toward reducing the burden of these cancers and aligning more closely with frontier benchmarks.

### 4.1. Limitations

The major strength of this investigation is that it provides the first systematic estimation of the diseases burden of HNC and its three subtypes in middle-aged and older adults from 1990 to 2021 worldwide. The study was based on a secondary exploration of the GBD 2021 database, the most up-to-date version of the database, known for its robust methodology, extensive sample diversity, and sophisticated statistical techniques. By calculating the SII and CIX, we assessed the absolute and relative health inequalities across countries and GBD regions, and through frontier analysis, we revealed countries and territories where improvement could be possibly achieved. However, we must note that the inherent limitations of this study could potentially influence the interpretation of its findings. First of all, although the GBD methodology of DisMod-MR ensures the consistency of epidemiological parameters and is considered reliable and robust, the accuracy of our study’s conclusions greatly depends on the quality and availability of epidemiological data, frequently absent in low SDI countries with limited medical resources. Consequently, this shortcoming could result in an underestimation of the inequality in the burden of HNCs. In addition, findings based on very small populations should be interpreted with caution, as limited case numbers may lead to disproportionate effects and overstatement of apparent trends. Secondly, our analysis was limited to LC, NPC, and LOC, excluding other HNC subtypes such as oropharyngeal cancer. Although oropharyngeal cancer incidence is rising, particularly in high SDI regions and in association with HPV infection, separate estimates are not available in the current GBD database. We will continue to monitor GBD updates and aim to include oropharyngeal cancer in future analyses as relevant data become available. Thirdly, the CIX, a World Health Organization recommended tool for inequality assessment, is notably sensitive to populous nations like China and India, potentially biasing the outcomes. This is because these two countries have large population bases, which may mask trends in smaller countries. Fourthly, our study spans from 1990 to 2021, and its extended timeframe may be influenced by evolving diagnostic criteria and medical technological advancements, possibly distorting trend interpretations. Finally, health inequality analysis was not conducted because the GBD data for Australasia and Tropical Latin America cover only two countries. These limitations should be considered when evaluating the findings and their implications for public health policy. Future studies should combine original epidemiological survey data with multi-source databases to enhance the representativeness of the results.

## 5. Conclusion

In summary, this research examined global trends in age-standardized incidence and DALY rates of HNC and its three major subtypes among middle-aged and older adults from 1990 to 2021, revealing substantial health inequalities across global and GBD regions. Lower SDI countries and territories bore a disproportionately higher burden, particularly in LC and LOC, while some high SDI regions, including Taiwan (Province of China), Greenland, Slovakia, Poland, and Lithuania, may have opportunities to reduce these burdens by leveraging socio-demographic advantages. These findings underscore persistent global disparities in the management of HNC and its subtypes in middle-aged and older adults, highlighting the urgency for health systems worldwide to prioritize equitable cancer prevention and control strategies.

## Supporting information

S1 File Basic support information. (PDF)
