# Supplementary material for: Global burden, health inequalities and improvement gap of head and neck cancers in middle-aged and older adults from 1990 to 2021
Source: PLoS One. 2025 Nov 6;20(11):e0335969. doi: 10.1371/journal.pone.0335969 (PMC12591431; doi:10.1371/journal.pone.0335969)

Global burden, health inequalities and improvement gap of head and neck cancers in middle-aged and older adults from 1990 to 2021

Minxia Yang [1], Feng Xuan [2], Xiaofeng Ma[3], Zhaoqi Qiu [2, *]

[1] Department of Radiology, Shaoxing People's Hospital, Shaoxing, Zhejiang Province, China.

[2] Department of Radiation Oncology, Zhuji Affiliated Hospital of Wenzhou Medical University, Shaoxing, China.

[3] Department of Radiology, Zhuji Affiliated Hospital of Wenzhou Medical University, Shaoxing, China

*** Correspondence:** Zhaoqi Qiu

Email: qzzdbd@163.com

Table S1: The Socio-demographic Index (SDI) values from the Global Burden of Disease (GBD) data across regions and locations released by the Institute for Health Metrics and Evaluation (IHME) in 2021 4

Table S2: The Socio-demographic Index (SDI) reference values from the Global Burden of Disease (GBD) data released by the Institute for Health Metrics and Evaluation (IHME) in 2021... 15

Table S3: The Guidelines for Accurate and Transparent Health Assessment Reporting (GATHER). 16

Table S4: Incidence and DALYs of overall head and neck cancers in middle-aged and older adults, and their average annual percentage changes in 204 countries/territories from 1990 to 2021. 18

Table S5: Incidence and DALYs of larynx cancer, nasopharynx cancer, and lip and oral cavity cancer in middle-aged and older adults, and their average annual percentage changes from 1990 to 2021 at global and 21 GBD regions........ 41

Table S6: Incidence and DALYs of Larynx cancer in middle-aged and older adults, and their average annual percentage changes in 204 countries/territories from 1990 to 2021........ 57

Table S7: Incidence and DALYs of Nasopharynx cancer in middle-aged and older adults, and their average annual percentage changes in 204 countries/territories from 1990 to 2021........ 80

Table S8: Incidence and DALYs of Lip and oral cavity cancer in middle-aged and older adults, and their average annual percentage changes in 204 countries/territories from 1990 to 2021... 103

Table S9: Absolute and relative cross-country inequality for overall head and neck cancer of age-standardized DALYs rate in middle-aged and older adults from 1990 to 2021........ 124

Table S10: Frontier analysis on the basis of sociodemographic-index and age-standardized DALYs rate of overall head and neck cancer in middle-aged and older adults from 1990 to 2021.. 126

Table S11: Absolute and relative cross-country inequality for larynx cancer of age-standardized DALYs rate in middle-aged and older adults from 1990 to 2021. 136

Table S12: Frontier analysis on the basis of sociodemographic-index and age-standardized DALYs rate of larynx cancer in middle-aged and older adults from 1990 to 2021........ 138

Table S13: Absolute and relative cross-country inequality for nasopharynx cancer of age-standardized DALYs rate in middle-aged and older adults from 1990 to 2021. 148

Table S14: Frontier analysis on the basis of sociodemographic-index and age-standardized DALYs rate of nasopharynx cancer in middle-aged and older adults from 1990 to 2021........ 150

Table S15: Absolute and relative cross-country inequality for lip and oral cavity cancer of age-standardized DALYs rate in middle-aged and older adults from 1990 to 2021.......................... 160

Table S16: Frontier analysis on the basis of sociodemographic-index and age-standardized DALYs rate of lip and oral cavity cancer in middle-aged and older adults from 1990 to 2021. 162

Figure S1. Average annual percentage change of ASIR and ASDR for HNC and its subtypes among middle-aged and older adults at 204 countries and territories from 1990 to 2021. **A**, AAPC of ASIR for HNC. **B**, AAPC of ASDR for HNC. **C**, AAPC of ASIR for larynx cancer. **D**, AAPC of ASDR for larynx cancer. **E**, AAPC of ASIR for nasopharynx cancer. **F**, AAPC of ASDR for nasopharynx cancer. **G**, AAPC of ASIR for lip and oral cavity cancer. **H**, AAPC of ASDR for lip and oral cavity cancer. The black cross represents the ASR for each country, the blue solid dot represents the ASR for GBD regions, and the red text denotes the countries with the maximum and minimum ASR within each GBD region.. 172

Figure S2. Temporal trend of slope index of inequality and concentration index of head and neck cancer and its subtypes among middle-aged and older adults at global level from 1990 to 2021. 174

Figure S3. Temporal trend of slope index of inequality of head and neck cancer and its subtypes among middle-aged and older adults at global and GBD regions from 1990 to 2021............. 175

Figure S4. Temporal trend of concentration index of head and neck cancer and its subtypes among middle-aged and older adults at global and GBD regions from 1990 to 2021..................... 176

S1 Table: The Socio-demographic Index (SDI) values from the Global Burden of Disease (GBD) data across regions and locations released by the Institute for Health Metrics and Evaluation (IHME) in 2021.

	Location Name	SDI value (2021)
**Global and 21 GBD regions**	Global	0.666367819
	East Asia	0.722912119
	Southeast Asia	0.64907177
	Oceania	0.467359461
	Central Asia	0.674963478
	Central Europe	0.795780357
	Eastern Europe	0.803414319
	High-income Asia Pacific	0.877157409
	Australasia	0.845644432
	Western Europe	0.848728514
	Southern Latin America	0.743029817
	High-income North America	0.86421664
	Caribbean	0.6423146
	Andean Latin America	0.654007956
	Central Latin America	0.641931122
	Tropical Latin America	0.648941531
	North Africa and Middle East	0.658716072
	South Asia	0.559642669
	Central Sub-Saharan Africa	0.484517732
	Eastern Sub-Saharan Africa	0.412187942
	Southern Sub-Saharan Africa	0.643347819

	Location Name	SDI value (2021)
	Western Sub-Saharan Africa	0.446420999
**204 countries and territories**	Somalia	0.077688109
	Niger	0.168072774
	Chad	0.240436019
	Mali	0.268579941
	South Sudan	0.278371125
	Burkina Faso	0.285118402
	Burundi	0.289374365
	Central African Republic	0.30916769
	Mozambique	0.326462614
	Guinea	0.336401293
	Afghanistan	0.337199998
	Liberia	0.352442452
	Guinea-Bissau	0.353109621
	Sierra Leone	0.358665881
	Ethiopia	0.358823295
	Benin	0.373486574
	Democratic Republic of the Congo	0.383179849
	Malawi	0.384553634
	Madagascar	0.400246943
	Eritrea	0.403863943
	Senegal	0.408054193

	Location Name	SDI value (2021)
	Togo	0.408533695
	Papua New Guinea	0.417797443
	Uganda	0.423261181
	Coted'Ivoire	0.425941883
	Solomon Islands	0.429360316
	Nepal	0.433174635
	Rwanda	0.435588706
	Timor-Leste	0.444667619
	United Republic of Tanzania	0.446568273
	Haiti	0.448278285
	Yemen	0.450376375
	Angola	0.453721949
	Gambia	0.40971416
	Bhutan	0.473062378
	Vanuatu	0.473100706
	Cambodia	0.473621491
	Zimbabwe	0.473819486
	Comoros	0.475978688
	Cameroon	0.479691223
	Djibouti	0.487958371
	Lao People's Democratic Republic	0.489136091
	Bangladesh	0.492420885

Location Name	SDI value (2021)
Mauritania	0.4989451
Nigeria	0.503390833
Pakistan	0.504028689
Sao Tome and Principe	0.505413747
Zambia	0.505948954
Lesotho	0.510393066
Honduras	0.513037248
Kenya	0.523768077
Nicaragua	0.523958472
Kiribati	0.527186583
Cabo Verde	0.533534539
Myanmar	0.53390084
Guatemala	0.539972424
Tajikistan	0.541511187
Sudan	0.541949735
Morocco	0.562698301
El Salvador	0.563775188
Ghana	0.56493039
Marshall Islands	0.574091128
India	0.575401649
Tuvalu	0.576620529
Congo	0.583075236

	Location Name	SDI value (2021)
	Eswatini	0.585459713
	Micronesia (Federated States of)	0.587534967
	Samoa	0.593392769
	Venezuela (Bolivarian Republic of)	0.596513059
	Bolivia (Plurinational State of)	0.599010799
	Kyrgyzstan	0.603979328
	Egypt	0.606787094
	Belize	0.610229002
	Namibia	0.617564872
	Mongolia	0.617621565
	Dominican Republic	0.619388201
	Syrian Arab Republic	0.623004075
	Nauru	0.625177834
	Tonga	0.626349936
	Viet Nam	0.627933721
	Palestine	0.631011665
	Suriname	0.633665739
	Gabon	0.634691393
	Paraguay	0.635718099
	Saint Vincent and the Grenadines	0.637195963
	Botswana	0.642721629
	Guyana	0.650812335

Location Name	SDI value (2021)
Maldives	0.650886627
Philippines	0.651219329
Brazil	0.653043887
Colombia	0.655442913
Indonesia	0.656868336
Equatorial Guinea	0.657857456
Algeria	0.659500924
Ecuador	0.661017053
Peru	0.662054037
Uzbekistan	0.662621694
Iraq	0.662626231
Mexico	0.664575304
Cuba	0.668729864
Grenada	0.668993028
Saint Lucia	0.672509735
Fiji	0.675051631
South Africa	0.679626598
Turkmenistan	0.682160776
Tunisia	0.682432216
Thailand	0.682547933
Jamaica	0.683263064
Tokelau	0.686425621

	Location Name	SDI value (2021)
	Azerbaijan	0.694851274
	Iran (Islamic Republic of)	0.697207398
	Costa Rica	0.700340477
	Sri Lanka	0.701534935
	Armenia	0.701833194
	Albania	0.706849791
	Panama	0.708864828
	Turkey	0.712692673
	Mauritius	0.718260446
	Uruguay	0.719283445
	China	0.72162976
	Bosnia and Herzegovina	0.723077893
	Argentina	0.723122973
	American Samoa	0.723727533
	Kazakhstan	0.725144495
	Jordan	0.725307227
	Libya	0.725771399
	Niue	0.72622205
	Seychelles	0.730150775
	Republic of Moldova	0.732214875
	Georgia	0.732473604
	Malaysia	0.742523828

	Location Name	SDI value (2021)
	Portugal	0.744151851
	Lebanon	0.744746351
	Barbados	0.746748764
	Dominica	0.746967185
	Antigua and Barbuda	0.749886887
	North Macedonia	0.750629703
	Democratic People's Republic of Korea	0.5698546
	Bahrain	0.753043204
	Palau	0.754046931
	Saint Kitts and Nevis	0.754987055
	Ukraine	0.760773913
	Bulgaria	0.768150939
	Romania	0.768453864
	Trinidad and Tobago	0.768763254
	Spain	0.769283698
	Chile	0.771514716
	Northern Mariana Islands	0.771535213
	Oman	0.773391602
	Cook Islands	0.779109955
	Belarus	0.784484711
	Hungary	0.790754768
	Greece	0.791854408

	Location Name	SDI value (2021)
	Serbia	0.792416294
	Montenegro	0.795800584
	Croatia	0.798341027
	Malta	0.801585034
	Guam	0.803982203
	Bahamas	0.805020668
	Italy	0.805773534
	Russian Federation	0.808536005
	Israel	0.809011652
	Brunei Darussalam	0.810234367
	Slovakia	0.81061053
	Poland	0.812042809
	Saudi Arabia	0.815143493
	Bermuda	0.821365422
	United States Virgin Islands	0.821830853
	Puerto Rico	0.825525847
	Greenland	0.826210336
	Czechia	0.828450433
	Latvia	0.830663516
	Cyprus	0.835630545
	France	0.838364875
	Slovenia	0.842430731

	Location Name	SDI value (2021)
	Australia	0.844252814
	Estonia	0.844917787
	Kuwait	0.846651055
	Qatar	0.846860584
	United Arab Emirates	0.849317734
	New Zealand	0.849442499
	Belgium	0.853654016
	Austria	0.853837004
	Singapore	0.856097766
	Lithuania	0.856484049
	United Kingdom	0.859000182
	Finland	0.859831368
	United States of America	0.862448354
	Andorra	0.869444113
	Japan	0.871241813
	Canada	0.87317068
	Ireland	0.87375385
	Taiwan (Province of China)	0.874747053
	Iceland	0.87636168
	Luxembourg	0.884428955
	Republic of Korea	0.886675267
	Sweden	0.886880299

Location Name	SDI value (2021)
San Marino	0.888005474
Netherlands	0.888464256
Denmark	0.896424204
Germany	0.902957091
Monaco	0.908262831
Norway	0.91613281
Switzerland	0.933059111

S2 Table: The Socio-demographic Index (SDI) reference values from the Global Burden of Disease (GBD) data released by the Institute for Health Metrics and Evaluation (IHME) in 2021.

SDI regions	lower bound	upper bound
Low SDI	0	0.46581580319161997
Low-middle SDI	0.46581580319161997	0.6188294452454329
Middle SDI	0.6188294452454329	0.7119746219361235
High-middle SDI	0.7119746219361235	0.8102959891918925
High SDI	0.8102959891918925	1

S3 Table: The Guidelines for Accurate and Transparent Health Assessment Reporting (GATHER).

**Checklist of information that should be included in new reports of global health estimates**		
Item	Checklist item	Reported on page#
**Objectives and funding**		
**1**	Define the indicator(s), populations (including age, sex, and geographic entities),and time period(s)for which estimates were made.	Pages:4-5
**2**	List the funding sources for the work.	NA
**Data Inputs**		
**For all data inputs from multiple sources that are synthesized as part of the study:**		
**3**	Describe how the data were identified and how the data were accessed.	Pages:4-5,25
**4**	Specify the inclusion and exclusion criteria. Identify all ad-hoc exclusions.	NA
**5**	Provide information on all included data sources and their main characteristics. For each data source used, report reference information or contact name/institution, population represented, data collection method, year(s)of data collection, sex and age range, diagnostic criteria or measurement method, and sample size, as relevant.	Pages:4-7
**6**	Identify and describe any categories of input data that have potentially important biases (e.g., based on characteristics listed in item 5)	Page:23-24
**For data inputs that contribute to the analysis but were not synthesized as part of the study:**		
**7**	Describe and give sources for any other data inputs.	NA
**For all data inputs:**		
**8**	Provide all data inputs in a file format from which data can be efficiently extracted (e.g., a spreadsheet rather than a PDF),including all relevant meta-data listed in item 5.For any data inputs that cannot be shared because of ethical or legal reasons, such as third-party ownership, provide a contact name or the name of the institution that retains the right to the data.	Pages:7-19. Supplementary 1 Tables 4-13
**Data analysis**		

**9**	Provide a conceptual overview of the data analysis method. A diagram may be helpful.	Pages:4-7
**10**	Provide a detailed description of all steps of the analysis, including mathematical formulae. This description should cover, as relevant, data cleaning, data pre-processing, data adjustments and weighting of data sources, and mathematical or statistical model(s).	Pages:4-7
**11**	Describe how candidate models were evaluated and how the final model(s)were selected	Pages:4-7
**12**	Provide the results of an evaluation of model performance, if done, as well as the results of any relevant sensitivity analysis.	Pages:4-7
**13**	Describe methods for calculating uncertainty of the estimates. State which sources of uncertainty were, and were not, accounted for in the uncertainty analysis.	Pages:4-7
**14**	State how analytic or statistical source code used to generate estimates can be accessed	Page:7
**Results and Discussion**		
**15**	Provide published estimates in a file format from which data can be efficiently extracted	Pages:7-24
**16**	Report a quantitative measure of the uncertainty of the estimates (e.g. uncertainty intervals).	Pages:7-24
**17**	Interpret results in light of existing evidence. If updating a previous set of estimates, describe the reasons for changes in estimates.	Pages:7-24
**18**	Discuss limitations of the estimates. Include a discussion of any modelling assumptions or data limitations that affect interpretation of the estimates.	Page:23-24

S4 Table: Incidence and DALYs of overall head and neck cancers in middle-aged and older adults, and their average annual percentage changes in 204 countries/territories from 1990 to 2021.

location	Incidence					DALYs				
	Num in 1990	ASR in 1990	Num in 2021	ASR in 2021	AAPC	Num in 1990	ASR in 1990	Num in 2021	ASR in 2021	AAPC
Afghanistan	334(166-563)	17.17(8.75-28.6)	407(209-672)	15.3(8.16-24.51)	-0.37(-0.42 to -0.32)	8663(4173-14792)	422.23(207.2-714.11)	10398(5175-17374)	344.76(177.52-558.89)	-0.65(-0.71 to -0.59)
Albania	123(88-167)	21.65(15.59-29.33)	252(166-366)	21.1(13.93-30.77)	0(-0.72 to 0.73)	2715(1961-3691)	458.29(331.5-622.35)	3891(2568-5678)	330.24(217.63-482.85)	-1(-1.38 to -0.62)
Algeria	615(412-891)	19.41(13.01-28.14)	1626(1051-2416)	16.81(10.9-24.94)	-0.47(-0.53 to -0.41)	14669(9835-21235)	430.3(288.79-623.43)	31387(20296-46289)	304.64(197.43-448.96)	-1.19(-1.28 to -1.09)
American Samoa	1(0-1)	11.68(7.65-17.28)	1(1-2)	10.48(7.04-15.02)	-0.42(-0.74 to -0.1)	17(11-25)	255.94(167.65-382.04)	33(22-47)	224.67(151.04-322.2)	-0.44(-0.71 to -0.16)
Andorra	4(2-6)	23.95(13.65-39.73)	7(4-12)	16.52(8.95-27.64)	-1.27(-2.22 to -0.31)	42(25-69)	266.9(155.13-434.52)	61(34-100)	142.86(79.2-235.11)	-2.29(-2.89 to -1.7)
Angola	163(99-251)	15.09(9.22-23.14)	499(318-751)	15.32(9.78-23.02)	0.03(-0.05 to 0.11)	4381(2618-6811)	370.09(223.3-572.11)	12091(7729-18311)	337.07(216.32-509.1)	-0.35(-0.55 to -0.15)
Antigua and Barbuda	3(2-3)	19.63(16.5-23.05)	6(5-7)	18.51(15.25-22.21)	-0.52(-1.67 to 0.64)	48(41-56)	354.55(302.73-411.89)	90(76-107)	293.65(246.31-346.85)	-0.91(-2.04 to 0.24)

location	Incidence					DALYs				
	Num in 1990	ASR in 1990	Num in 2021	ASR in 2021	AAPC	Num in 1990	ASR in 1990	Num in 2021	ASR in 2021	AAPC
Argentina	2391(1942-2910)	26.87(21.81-32.71)	2754(2205-3394)	18.24(14.6-22.5)	-1.08(-1.39 to -0.76)	46461(37979-56177)	521.14(425.89-630.29)	40565(32997-49287)	272.18(221.35-330.92)	-1.89(-2.04 to -1.74)
Armenia	180(161-200)	22.47(20.06-25.13)	191(160-227)	15.88(13.25-18.85)	-1.46(-2.03 to -0.89)	4033(3669-4412)	488.74(443.35-536.8)	3301(2796-3850)	276.55(234.29-322.4)	-2.22(-2.8 to -1.64)
Australia	1845(1502-2259)	35.2(28.6-43.14)	3473(2720-4339)	29.41(23.06-36.75)	-0.51(-0.89 to -0.12)	16870(14099-20020)	324.58(271.07-385.37)	20721(16806-25173)	178.78(145.16-217.33)	-1.97(-2.12 to -1.82)
Austria	780(635-951)	26.23(21.28-32.05)	982(774-1218)	21.17(16.73-26.29)	-0.42(-0.71 to -0.13)	12066(9975-14491)	417.66(344.79-502.25)	10405(8426-12587)	231.23(187.47-279.98)	-1.74(-2.1 to -1.37)
Azerbaijan	222(174-282)	15.32(11.88-19.58)	359(246-529)	11.79(8.09-17.33)	-0.55(-1.05 to -0.05)	5485(4321-6902)	364.26(285.58-460.73)	7457(5178-11121)	233.64(162.24-347.13)	-1.43(-1.81 to -1.05)
Bahamas	12(10-14)	28.86(24.49-33.88)	32(24-41)	27.24(20.87-35)	-0.06(-0.46 to 0.35)	249(213-289)	572.76(490.87-664.18)	588(451-759)	486.84(374.94-625.62)	-0.34(-0.53 to -0.14)
Bahrain	11(7-15)	24.97(17.17-35.73)	45(27-72)	19.41(11.96-30.19)	-0.78(-0.99 to -0.58)	215(148-307)	451.77(311.69-641.64)	627(367-1010)	235.55(143.19-370.07)	-2.11(-2.27 to -1.94)
Bangladesh	5671(3592-8429)	44.46(28.14-66.02)	13360(7845-20850)	35.19(20.82-54.68)	-0.67(-0.84 to -0.5)	139810(88607-208805)	1053.85(667.49-1572.55)	258444(151076-407642)	662.01(388.98-1041.33)	-1.4(-1.65 to -1.16)
Barbados	16(14-18)	20.53(17.58-23.87)	29(22-38)	20.6(15.26-26.77)	0.12(-0.01 to 0.24)	260(227-298)	359.2(312.96-411.89)	456(340-591)	322.36(240.09-418.99)	-0.21(-0.33 to -0.08)

location	Incidence					DALYs				
	Num in 1990	ASR in 1990	Num in 2021	ASR in 2021	AAPC	Num in 1990	ASR in 1990	Num in 2021	ASR in 2021	AAPC
Belarus	1392(1115-1719)	38.31(30.67-47.31)	1698(1234-2279)	39.36(28.56-52.9)	0(-0.42 to 0.43)	28074(22504-34594)	773.39(619.49-953.57)	24573(18090-32821)	579.99(426.36-775.67)	-0.91(-1.39 to -0.43)
Belgium	1533(1221-1905)	38.78(30.81-48.28)	1709(1330-2156)	29.45(22.95-37.22)	-1.19(-2.16 to -0.21)	18413(15109-22276)	478.32(391.89-579.52)	15040(12098-18367)	267.65(215.89-327.05)	-1.81(-2.22 to -1.41)
Belize	3(2-3)	11.47(9.85-13.26)	11(9-13)	13.21(10.8-15.98)	0.6(-0.03 to 1.24)	57(49-65)	227.42(197.07-260.14)	213(174-256)	247.33(202.85-296.84)	0.45(-0.2 to 1.1)
Benin	39(26-56)	7.36(4.91-10.58)	122(76-186)	8.75(5.49-13.21)	0.5(0.28 to 0.71)	935(624-1346)	171.04(114.13-246.05)	2806(1721-4337)	187.91(116.35-288.14)	0.24(-0.16 to 0.63)
Bermuda	7(5-8)	38.36(30.65-47.53)	11(8-14)	29.04(21.34-39.08)	-0.99(-1.34 to -0.64)	101(81-125)	589.45(472.67-726.17)	114(84-150)	317.53(235.22-421.27)	-1.98(-2.19 to -1.76)
Bhutan	23(14-36)	34.51(20.6-53.35)	53(33-80)	32.11(20.05-48.83)	-0.25(-0.34 to -0.16)	593(348-927)	810.57(479.36-1265.64)	1017(624-1582)	607.27(374.1-941.39)	-0.92(-1 to -0.84)
Bolivia (Plurinational State of)	101(64-150)	11.97(7.69-17.77)	259(160-396)	10.65(6.63-16.22)	-0.35(-0.41 to -0.29)	2355(1515-3479)	266.45(171.75-393.43)	5054(3120-7736)	199.98(123.97-304.87)	-0.91(-0.95 to -0.87)
Bosnia and Herzegovina	317(255-387)	25.97(20.93-31.78)	451(322-601)	26.86(19.14-35.92)	0.14(-0.16 to 0.44)	6890(5577-8405)	544.44(440.66-664.27)	6951(4960-9154)	422.19(300.13-558.33)	-0.66(-1.08 to -0.23)
Botswana	39(25-59)	25.07(15.89-37.47)	90(53-144)	21.63(13.18-34)	-0.52(-1 to -0.04)	957(593-1450)	578.9(361.26-872.36)	2025(1174-3327)	457.82(271.39-740.18)	-0.79(-1.11 to -0.47)

location	Incidence					DALYs				
	Num in 1990	ASR in 1990	Num in 2021	ASR in 2021	AAPC	Num in 1990	ASR in 1990	Num in 2021	ASR in 2021	AAPC
Brazil	5957(5486-6452)	24.2(22.2-26.22)	16574(14966-18152)	23.7(21.37-25.96)	-0.05(-0.17 to 0.07)	129495(119575-140009)	504.76(465.23-545.7)	292751(267172-318762)	414.59(378.02-451.5)	-0.6(-0.75 to -0.45)
Brunei Darussalam	10(7-14)	36.77(25.22-52.3)	27(19-37)	27.55(19.19-38.52)	-0.83(-1.06 to -0.6)	194(133-276)	690.33(472.44-980.51)	487(338-680)	456.36(317.43-634.81)	-1.2(-1.44 to -0.96)
Bulgaria	911(736-1114)	27.09(21.84-33.19)	1444(1113-1834)	41.08(31.56-52.29)	1.55(1.05 to 2.05)	15970(13089-19245)	473.39(386.9-571.92)	19383(15396-23949)	568.08(450.42-702.76)	0.63(-0.48 to 1.75)
Burkina Faso	91(59-135)	7.68(4.97-11.36)	239(150-363)	9.63(6.08-14.56)	0.7(0.55 to 0.86)	2220(1420-3288)	177.96(114.21-263.2)	5618(3467-8613)	212.39(132.27-323.97)	0.54(0.36 to 0.72)
Burundi	172(110-255)	26.89(17.26-39.74)	263(159-409)	19.22(11.84-29.6)	-1.1(-1.19 to -1.02)	4532(2865-6772)	685.84(434.92-1022.33)	6814(4082-10820)	462.33(280.9-726.53)	-1.32(-1.39 to -1.25)
Cabo Verde	4(3-6)	6.58(4.2-9.84)	30(19-46)	24.28(15.21-36.92)	4.21(3.5 to 4.92)	87(56-129)	141.14(91.11-209.91)	532(329-811)	410.89(254.64-624.88)	3.39(2.7 to 4.08)
Cambodia	273(176-405)	22.42(14.58-33.15)	830(521-1270)	24.54(15.58-37.2)	0.33(0.28 to 0.37)	6931(4430-10359)	535.17(344.2-796.68)	18092(11313-27875)	504.14(318-771.48)	-0.16(-0.2 to -0.12)
Cameroon	105(71-152)	8.5(5.76-12.28)	378(230-591)	10.91(6.75-16.89)	0.8(0.71 to 0.89)	2550(1721-3718)	194.05(131.53-281.74)	8736(5241-13813)	233.9(142.04-366.52)	0.58(0.53 to 0.64)
Canada	3443(2788-4220)	39.55(32-48.53)	4580(3612-5730)	23.72(18.7-29.71)	-1.62(-2.03 to -1.21)	32107(27155-37796)	373.88(315.95-440.25)	33049(27091-39908)	175.56(144.1-211.91)	-2.36(-2.54 to -2.18)

location	Incidence					DALYs				
	Num in 1990	ASR in 1990	Num in 2021	ASR in 2021	AAPC	Num in 1990	ASR in 1990	Num in 2021	ASR in 2021	AAPC
Central African Republic	54(31-86)	16.76(9.98-26.33)	91(52-145)	14.39(8.56-22.48)	-0.48(-0.54 to -0.42)	1474(848-2395)	421.42(247.52-674.73)	2523(1440-4081)	357.26(209.3-566.3)	-0.52(-0.58 to -0.46)
Chad	46(30-68)	6.14(4.01-9.03)	156(97-235)	9.84(6.15-14.81)	1.6(1.52 to 1.68)	1120(734-1647)	144.29(94.43-212.46)	3862(2366-5840)	227.74(140.56-343.13)	1.56(1.46 to 1.66)
Chile	380(309-465)	13.82(11.26-16.92)	718(571-893)	10.16(8.07-12.64)	-0.91(-1.27 to -0.55)	6824(5602-8266)	244.34(200.63-295.84)	8323(6788-10107)	118.38(96.51-143.8)	-2.26(-2.62 to -1.9)
China	56979(46857-67458)	24.6(20.28-29.04)	135780(105178-172748)	22.49(17.46-28.54)	-0.29(-0.51 to -0.06)	1407405(1152066-1670539)	578.65(474.67-685.42)	1808082(1398498-2300132)	295.5(228.86-375.17)	-2.23(-2.39 to -2.06)
Colombia	852(717-998)	18.67(15.69-21.86)	1753(1324-2248)	11.62(8.78-14.89)	-1.69(-2.03 to -1.35)	16982(14373-19848)	356.1(301.27-416.04)	24754(18787-31744)	163.4(124.11-209.44)	-2.48(-3.04 to -1.92)
Comoros	13(8-19)	22.5(14.2-33.9)	27(17-41)	19.54(12.39-29.92)	-0.53(-0.71 to -0.35)	323(197-494)	545.88(336.96-827.88)	636(390-1001)	438.53(271.72-683.58)	-0.77(-0.98 to -0.55)
Congo	52(31-80)	17.86(10.86-27.23)	125(79-187)	16.62(10.81-24.58)	-0.22(-0.33 to -0.1)	1322(771-2075)	424.7(251.73-659.39)	2932(1850-4435)	354.64(228.18-529.49)	-0.56(-0.68 to -0.43)
Cook Islands	0(0-0)	9.21(6.06-13.43)	1(0-1)	10.57(6.91-15.6)	0.43(0.32 to 0.54)	6(4-8)	160.35(105.44-235.15)	10(7-15)	141.19(92.23-208.64)	-0.37(-0.51 to -0.24)
Costa Rica	85(69-103)	18.31(14.91-22.23)	184(142-233)	12.19(9.43-15.48)	-1.34(-1.7 to -0.98)	1447(1186-1742)	307.51(252.13-370.12)	2569(2003-3237)	169.73(132.5-213.69)	-1.97(-2.29 to -1.65)

location	Incidence					DALYs				
	Num in 1990	ASR in 1990	Num in 2021	ASR in 2021	AAPC	Num in 1990	ASR in 1990	Num in 2021	ASR in 2021	AAPC
Coted'Ivoire	138(91-202)	12.31(8.18-17.79)	406(239-651)	12.78(7.74-20.08)	0.18(0.02 to 0.34)	3525(2286-5205)	286.28(187.85-419.08)	9545(5504-15625)	276.47(163.78-445.05)	-0.05(-0.23 to 0.14)
Croatia	859(697-1049)	49.1(39.87-59.96)	864(678-1086)	37.86(29.63-47.63)	-0.74(-0.84 to -0.64)	14757(12107-17826)	827.96(679.27-1000.34)	9537(7638-11798)	436.57(348.79-540.48)	-1.97(-2.17 to -1.77)
Cuba	1070(879-1287)	38.53(31.64-46.33)	2651(2022-3418)	48.91(37.29-63.09)	0.97(0.87 to 1.08)	17348(14423-20738)	625.45(519.97-747.56)	37621(29126-48206)	697.25(539.66-893.8)	0.6(0.48 to 0.71)
Cyprus	40(27-58)	19.63(13.23-28.46)	98(66-142)	17.82(11.88-25.81)	-0.28(-0.49 to -0.07)	554(377-789)	263.42(178.89-377.25)	851(586-1222)	157.12(107.74-226.13)	-1.69(-1.94 to -1.43)
Czechia	1000(827-1197)	27.5(22.7-32.97)	1359(1082-1688)	25.04(19.88-31.18)	-0.19(-0.33 to -0.05)	19922(16610-23693)	559.6(465.68-666.53)	18077(14615-22229)	347.59(280.42-428.09)	-1.46(-1.59 to -1.32)
Democratic People's Republic of Korea	745(465-1123)	15.57(9.81-23.33)	1460(900-2208)	15.36(9.51-23.2)	-0.08(-0.13 to -0.03)	17975(11070-27236)	352.45(218.81-530.99)	29559(18072-44977)	303.89(186.81-461.39)	-0.52(-0.61 to -0.43)
Democratic Republic of the Congo	552(350-825)	13.05(8.27-19.48)	1314(817-1966)	12.95(8.06-19.42)	-0.04(-0.15 to 0.06)	14084(8850-21168)	305.75(192.77-458.4)	32206(19990-48462)	290.07(180.73-435.86)	-0.19(-0.29 to -0.09)
Denmark	522(432-627)	25.8(21.33-30.98)	759(614-923)	24.89(20.14-30.28)	0.09(-0.3 to 0.48)	6397(5443-7442)	325.53(276.98-378.74)	6441(5440-7517)	218.4(185.1-254.65)	-1.13(-1.37 to -0.89)
Djibouti	10(6-15)	24.21(14.93-38.23)	43(25-69)	23(13.68-36.34)	-0.19(-0.27 to -0.1)	254(150-418)	580.74(350.32-939.35)	1050(594-1736)	514.43(298.45-833.63)	-0.43(-0.49 to -0.37)

location	Incidence					DALYs				
	Num in 1990	ASR in 1990	Num in 2021	ASR in 2021	AAPC	Num in 1990	ASR in 1990	Num in 2021	ASR in 2021	AAPC
Dominica	4(3-5)	25.14(18.33-33.58)	6(4-9)	25.57(17.17-36.61)	0.11(0 to 0.22)	75(55-100)	471.72(345.13-629.44)	115(77-164)	478.26(320.33-685.07)	0.08(-0.05 to 0.22)
Dominican Republic	170(120-236)	17.74(12.55-24.61)	532(333-815)	19.57(12.24-29.96)	0.31(0.04 to 0.58)	3576(2539-4943)	354.59(251.65-490.33)	9850(6208-15183)	356.73(224.85-549.24)	0.18(-0.34 to 0.69)
Ecuador	111(92-132)	8.03(6.68-9.61)	334(243-448)	7.59(5.52-10.15)	0.03(-0.24 to 0.31)	2286(1910-2726)	160.28(133.85-191.04)	5304(3874-7158)	118.91(87.01-160.15)	-0.88(-1.14 to -0.62)
Egypt	442(325-611)	6.26(4.58-8.63)	1488(1043-2089)	8.85(6.24-12.37)	1.15(0.92 to 1.39)	10368(7645-14307)	133.39(98.23-183.93)	25996(18150-36325)	142.88(100.52-198.57)	0.25(-0.02 to 0.53)
El Salvador	76(60-94)	9.59(7.62-11.88)	168(123-225)	9.96(7.3-13.35)	0.2(-0.06 to 0.46)	1557(1243-1918)	193.19(154.13-238.05)	2633(1938-3518)	158.26(116.49-211.63)	-0.49(-0.88 to -0.1)
Equatorial Guinea	8(5-13)	15.34(9.13-24.33)	21(12-34)	15.6(9.25-24.68)	0.05(-0.05 to 0.14)	222(129-360)	379.93(222.68-609.6)	442(253-713)	299.05(173.84-476.82)	-0.76(-0.94 to -0.57)
Eritrea	86(54-132)	24.29(15.47-36.6)	172(108-260)	21.28(13.58-31.62)	-0.42(-0.5 to -0.33)	2503(1552-3845)	634.45(400.15-961.32)	4589(2863-6994)	514.74(325.87-774.51)	-0.67(-0.76 to -0.57)
Estonia	177(141-219)	31.31(24.9-38.83)	228(172-293)	34.32(25.92-44.27)	0.58(-0.15 to 1.31)	2922(2348-3589)	520.67(417.9-640.19)	2199(1691-2769)	346.78(265.79-438.15)	-0.99(-1.51 to -0.47)
Eswatini	21(13-31)	25.85(16.29-38.58)	43(25-70)	26.83(15.52-42.89)	0.18(0.1 to 0.27)	520(321-790)	604.67(375.89-912.44)	1046(590-1712)	614.8(350.57-993.83)	0.07(-0.02 to 0.17)

location	Incidence					DALYs				
	Num in 1990	ASR in 1990	Num in 2021	ASR in 2021	AAPC	Num in 1990	ASR in 1990	Num in 2021	ASR in 2021	AAPC
Ethiopia	1131(698-1692)	20.63(12.99-30.65)	1795(1281-2496)	15.61(11.2-21.56)	-0.91(-1.01 to -0.81)	30938(18853-46093)	523.19(323.84-777.01)	41958(29483-59442)	342.77(242.33-481.7)	-1.38(-1.44 to -1.32)
Fiji	13(9-18)	13.09(8.93-18.67)	26(17-39)	12.84(8.33-18.91)	-0.14(-0.41 to 0.12)	269(184-382)	256.49(175.42-365.45)	524(337-777)	237.34(154.08-349.74)	-0.28(-0.49 to -0.07)
Finland	335(275-403)	17.96(14.75-21.69)	552(441-679)	17.85(14.33-21.97)	0.13(0.04 to 0.22)	3843(3290-4472)	210.87(180.39-245.63)	4231(3553-4958)	143.11(121.08-167.52)	-1.18(-1.29 to -1.08)
France	13963(11138-17405)	68.62(54.62-85.76)	15229(11888-19156)	45.68(35.72-57.61)	-1.26(-1.42 to -1.1)	169630(139846-204337)	858.01(706.01-1035.28)	102779(82460-126137)	318.45(256.26-391.26)	-3.06(-3.22 to -2.9)
Gabon	29(19-44)	18.73(11.94-28.26)	53(34-79)	18.23(11.72-26.99)	-0.07(-0.13 to 0)	684(436-1037)	424.21(270.42-642.95)	1143(724-1717)	364.98(233.25-544.46)	-0.45(-0.53 to -0.37)
Gambia	6(4-9)	6.58(4.41-9.47)	21(13-31)	7.85(5.04-11.53)	0.61(-0.01 to 1.25)	146(97-214)	145.4(96.71-211.9)	453(286-672)	161.7(102.84-238.89)	0.39(-0.32 to 1.1)
Georgia	464(396-540)	25.83(22-30.06)	446(373-530)	27.71(23.12-32.9)	0.26(-0.37 to 0.89)	9980(8613-11497)	551.18(475.38-635.3)	8356(7047-9780)	529.95(446.68-620.44)	-0.08(-0.64 to 0.49)
Germany	9652(7861-11825)	29.72(24.17-36.46)	12318(9741-15273)	25.57(20.25-31.73)	-0.35(-0.95 to 0.26)	135071(112230-160726)	423.43(351.24-504.42)	108398(88005-130352)	233.96(190.43-281.53)	-1.88(-2.32 to -1.45)
Ghana	79(52-118)	4.62(3.03-6.87)	266(163-401)	5.9(3.62-8.84)	0.8(0.72 to 0.88)	1973(1278-2951)	107.78(70.24-160.54)	6013(3671-9150)	124.73(76.42-188.72)	0.52(0.4 to 0.64)

location	Incidence					DALYs				
	Num in 1990	ASR in 1990	Num in 2021	ASR in 2021	AAPC	Num in 1990	ASR in 1990	Num in 2021	ASR in 2021	AAPC
Greece	1365(1151-1606)	32.62(27.48-38.4)	1751(1442-2093)	30.13(24.9-36.07)	-0.27(-0.32 to -0.22)	14963(13216-16814)	361.77(319.4-406.75)	16428(14204-18709)	295.98(257.58-336.51)	-0.67(-0.82 to -0.52)
Greenland	8(6-10)	77.07(57.64-101.34)	9(6-12)	41.78(29.28-58.32)	-1.93(-2.06 to -1.8)	180(134-237)	1652.19(1240.07-2171.42)	162(114-226)	744.87(522.12-1044.9)	-2.54(-2.76 to -2.33)
Grenada	4(3-5)	22.06(17.44-27.54)	6(5-8)	19.14(14.65-24.39)	-0.42(-0.88 to 0.04)	80(63-99)	445.33(352.83-556.35)	116(89-148)	343.21(263.44-436.02)	-0.74(-1.65 to 0.18)
Guam	3(3-4)	16.05(12.29-20.44)	8(6-11)	14.65(10.91-19.22)	-0.38(-1.05 to 0.3)	78(61-98)	337.86(263.02-426.58)	164(124-212)	284.83(215.28-368.85)	-0.56(-1 to -0.11)
Guatemala	102(90-115)	12.02(10.63-13.51)	198(161-241)	6.8(5.52-8.24)	-1.82(-2.49 to -1.14)	2337(2081-2617)	251.62(223.92-281.71)	3704(3018-4473)	123.52(100.82-148.9)	-2.28(-3 to -1.56)
Guinea	141(96-197)	15.61(10.64-21.94)	303(195-444)	19.72(12.76-28.74)	0.8(0.73 to 0.86)	3292(2236-4622)	355.36(241.26-499.09)	6983(4440-10337)	432.98(277.2-637.61)	0.67(0.61 to 0.72)
Guinea-Bissau	11(6-17)	9.7(5.74-15.1)	24(15-37)	12.1(7.46-18.19)	0.78(0.7 to 0.87)	277(160-443)	240.3(140.23-381.61)	613(370-935)	281.65(171.87-425.22)	0.58(0.48 to 0.67)
Guyana	13(10-16)	12.44(9.88-15.5)	22(15-30)	11.72(8.21-16.13)	-0.03(-0.36 to 0.31)	292(230-367)	275.29(217.42-345.93)	480(334-669)	249.49(174.45-346.14)	-0.06(-0.43 to 0.32)
Haiti	194(115-308)	22.2(13.31-34.88)	375(218-596)	19.58(11.51-30.95)	-0.35(-0.38 to -0.31)	5016(2955-8010)	538.35(320.06-853.69)	9298(5363-14766)	448.12(260.4-708.45)	-0.54(-0.58 to -0.51)

location	Incidence					DALYs				
	Num in 1990	ASR in 1990	Num in 2021	ASR in 2021	AAPC	Num in 1990	ASR in 1990	Num in 2021	ASR in 2021	AAPC
Honduras	47(33-65)	8.84(6.26-12.19)	203(131-297)	12.13(7.82-17.76)	1.03(0.49 to 1.58)	1063(754-1474)	189.81(134.83-262.69)	4080(2654-5983)	233.74(151.83-342.08)	0.68(0.23 to 1.13)
Hungary	1877(1535-2278)	48.21(39.37-58.57)	2417(1922-2984)	50.75(40.27-62.8)	0.09(-0.17 to 0.36)	37438(30948-45124)	976.48(806.12-1177.95)	33711(27299-40903)	733.05(592.89-890.42)	-1.01(-1.35 to -0.67)
Iceland	17(14-21)	22.98(18.83-27.79)	31(25-39)	20.38(16.15-25.27)	-0.29(-0.62 to 0.04)	175(149-204)	240.87(205.1-280.93)	232(191-276)	155.69(128.84-185.22)	-1.42(-1.58 to -1.27)
India	48465(41022-56538)	36.81(30.96-43.14)	137121(116281-158864)	41.43(35.12-47.98)	0.44(0.29 to 0.59)	1201183(1015022-1404698)	856.78(720.73-1005.03)	2710782(2287442-3155140)	792.4(668.9-921.83)	-0.2(-0.32 to -0.08)
Indonesia	4201(3127-5348)	15.69(11.66-19.97)	11513(8124-15420)	17.53(12.42-23.32)	0.36(0.29 to 0.44)	101695(75874-129578)	352.75(262.84-449.15)	241780(169304-327463)	339.94(239.21-456.92)	-0.15(-0.2 to -0.1)
Iran (Islamic Republic of)	971(800-1146)	13.96(11.37-16.53)	2984(2485-3553)	14.34(11.9-17.07)	0.03(-0.17 to 0.22)	20372(16882-23770)	272.56(224.21-318.94)	40756(35234-46678)	189.45(163.18-217.08)	-1.15(-1.29 to -1.01)
Iraq	354(234-514)	16.58(10.95-23.98)	1177(746-1731)	17.82(11.35-26.06)	0.19(0.06 to 0.32)	7741(5165-11118)	351.78(235.05-504.17)	19258(12196-28128)	273.78(174.66-397.79)	-0.8(-0.91 to -0.69)
Ireland	347(279-428)	32.04(25.73-39.55)	480(373-606)	22.86(17.8-28.91)	-1.17(-1.61 to -0.72)	4193(3445-5036)	396.35(325.29-476.91)	3836(3099-4677)	186.27(150.62-227.29)	-2.38(-3.02 to -1.73)
Israel	200(161-246)	15.74(12.64-19.42)	474(367-598)	14.67(11.39-18.52)	-0.1(-0.75 to 0.55)	2779(2272-3366)	223.21(182.2-270.62)	4594(3668-5636)	145.69(116.62-178.77)	-1.36(-1.92 to -0.79)

location	Incidence					DALYs				
	Num in 1990	ASR in 1990	Num in 2021	ASR in 2021	AAPC	Num in 1990	ASR in 1990	Num in 2021	ASR in 2021	AAPC
Italy	9432(8233-10790)	39.8(34.69-45.58)	9605(8052-11202)	26.21(22.18-30.57)	-1.21(-1.43 to -0.99)	121578(112888-130270)	522.41(484.92-559.98)	85515(76393-93488)	240.86(218.07-262.18)	-2.46(-2.69 to -2.22)
Jamaica	63(50-77)	13.15(10.55-16.13)	121(84-170)	14.29(9.89-20.07)	0.49(0.06 to 0.92)	1106(892-1353)	237.34(191.28-290.31)	2073(1421-2927)	244.94(168-345.66)	0.29(-0.19 to 0.77)
Japan	8230(7377-9116)	17.48(15.64-19.38)	19098(15475-22422)	19.37(16.11-22.54)	0.49(-0.35 to 1.35)	70513(66719-73751)	149.59(141.2-156.6)	119944(104853-130085)	130.67(118.74-139.58)	-0.4(-0.73 to -0.08)
Jordan	55(37-80)	14.89(10.01-21.49)	239(152-364)	11.48(7.36-17.32)	-0.92(-1.06 to -0.79)	1195(804-1731)	296.92(200.55-428.31)	3646(2305-5567)	161.13(102.8-244.23)	-1.91(-2.12 to -1.71)
Kazakhstan	961(835-1100)	26.53(23.03-30.42)	908(745-1097)	17.57(14.42-21.21)	-1.3(-1.69 to -0.9)	21978(19317-24971)	590.05(517.86-671.38)	16842(13979-20035)	314.17(260.86-373.45)	-2.06(-2.56 to -1.56)
Kenya	472(324-645)	20.88(14.38-28.38)	1715(1275-2235)	26.41(19.78-34.19)	0.72(0.6 to 0.84)	11465(7827-15869)	481.65(329.58-664)	40609(29834-53433)	582.83(430.89-762.16)	0.6(0.5 to 0.7)
Kiribati	2(1-3)	19.73(12.33-29.78)	4(3-7)	21.49(12.79-33.95)	0.27(0.23 to 0.31)	52(33-80)	479.21(298.23-729.16)	111(66-180)	500.81(296.3-804.03)	0.14(0.11 to 0.17)
Kuwait	38(30-48)	22.36(17.54-28.23)	64(46-86)	8.18(5.94-10.95)	-2.73(-5.52 to 0.14)	625(496-784)	335.5(266.85-418.61)	741(546-991)	85.11(62.86-113.09)	-3.97(-6.57 to -1.29)
Kyrgyzstan	208(163-262)	24.99(19.59-31.36)	174(128-228)	12.62(9.33-16.5)	-2.24(-2.7 to -1.77)	4814(3755-6049)	566.64(442.37-711.22)	3515(2594-4604)	240.65(178.53-314.38)	-2.61(-3.8 to -1.41)

location	Incidence					DALYs				
	Num in 1990	ASR in 1990	Num in 2021	ASR in 2021	AAPC	Num in 1990	ASR in 1990	Num in 2021	ASR in 2021	AAPC
Lao People's Democratic Republic	124(76-192)	21.85(13.48-33.41)	226(143-340)	18.07(11.54-26.98)	-0.62(-0.7 to -0.54)	3268(1997-5087)	541.85(333.36-838.55)	5207(3268-7903)	387.43(245.5-584.42)	-1.08(-1.13 to -1.03)
Latvia	284(230-348)	28.72(23.21-35.21)	289(224-366)	29.55(22.8-37.45)	0.24(-0.63 to 1.11)	6111(4927-7485)	620.22(499.67-760.26)	4461(3467-5669)	482.48(373.98-615.01)	-0.7(-1.5 to 0.11)
Lebanon	132(81-203)	21.78(13.53-33.2)	343(230-492)	21.18(14.21-30.42)	-0.11(-0.33 to 0.11)	2560(1572-3930)	405.18(251.05-618.7)	4039(2749-5772)	253.42(172.31-362.49)	-1.53(-1.77 to -1.3)
Lesotho	45(29-67)	19.36(12.56-28.54)	90(54-141)	29.37(17.73-45.62)	1.47(1.3 to 1.64)	1082(698-1601)	451.27(291.87-667.36)	2259(1333-3557)	712.84(423.4-1116.92)	1.61(1.44 to 1.78)
Liberia	21(13-33)	7.02(4.4-10.85)	53(31-85)	9.07(5.41-14.41)	0.74(0.53 to 0.96)	516(320-814)	163.9(101.79-257.9)	1228(708-1971)	191.73(112.61-305.05)	0.37(0.13 to 0.62)
Libya	149(94-225)	28.44(18.01-42.98)	484(306-738)	31.57(20.15-47.81)	0.35(0.15 to 0.56)	3345(2097-5088)	611.29(385.64-927.75)	9475(6039-14335)	572.79(367.94-862.37)	-0.27(-0.67 to 0.13)
Lithuania	391(317-477)	31.29(25.38-38.22)	509(395-643)	35.89(27.75-45.45)	0.44(-0.52 to 1.41)	7692(6281-9315)	617.03(503.53-747.58)	7323(5717-9138)	539.39(420.07-673.42)	-0.41(-1.6 to 0.79)
Luxembourg	60(50-71)	41.59(34.86-49.49)	78(63-96)	27.58(22.3-33.81)	-1.14(-1.27 to -1.02)	793(692-908)	559.87(487.79-640.95)	683(577-797)	244.12(206.26-284.93)	-2.71(-3.13 to -2.29)
Madagascar	296(197-423)	20.86(13.93-29.77)	511(323-778)	15.48(9.93-23.26)	-0.97(-1.16 to -0.78)	7579(5001-10896)	509.47(337.42-731.39)	13126(8152-20141)	362.19(228.1-549.61)	-1.1(-1.28 to -0.92)

location	Incidence					DALYs				
	Num in 1990	ASR in 1990	Num in 2021	ASR in 2021	AAPC	Num in 1990	ASR in 1990	Num in 2021	ASR in 2021	AAPC
Malawi	113(77-160)	10.72(7.32-15.11)	247(158-368)	12.07(7.84-17.74)	0.45(0.36 to 0.54)	2779(1870-3954)	248.25(167.99-352.15)	5745(3658-8626)	262.39(169.41-389.85)	0.23(0.15 to 0.31)
Malaysia	1078(746-1515)	41.95(28.99-58.99)	3125(2134-4427)	39.57(27.08-55.91)	-0.26(-0.48 to -0.04)	26159(18005-36972)	969.88(668.12-1369.77)	61064(41530-86587)	748.96(510.39-1060.06)	-0.89(-1.12 to -0.67)
Maldives	4(3-7)	19.6(12.2-29.71)	13(9-19)	15.26(10.26-21.68)	-0.89(-1.51 to -0.27)	93(54-146)	372.72(226.8-576.06)	172(112-249)	190.87(127.3-273.19)	-2.29(-2.57 to -2.02)
Mali	108(76-150)	9.7(6.83-13.47)	241(153-362)	9.83(6.31-14.62)	0.06(-0.02 to 0.14)	2683(1882-3748)	227.25(159.63-316.95)	5648(3558-8621)	215.94(137.33-326.91)	-0.15(-0.23 to -0.06)
Malta	36(28-45)	30.57(24.01-38.55)	57(43-72)	24.12(18.35-30.98)	-0.73(-0.96 to -0.5)	504(404-622)	435.91(348.98-537.47)	547(427-685)	246.02(191.82-308.35)	-1.84(-1.99 to -1.69)
Marshall Islands	1(0-1)	12.62(8.05-19.14)	1(1-2)	13.98(8.47-21.59)	0.29(0.21 to 0.38)	13(8-21)	298.21(186.88-457.37)	33(20-52)	308.19(184.85-477.17)	0.07(-0.01 to 0.15)
Mauritania	21(14-31)	7.85(5.24-11.37)	58(36-90)	9.97(6.14-15.35)	0.77(0.66 to 0.88)	496(332-718)	178.77(119.76-258.34)	1160(704-1802)	189.51(116.02-292.56)	0.19(0.02 to 0.36)
Mauritius	54(46-63)	27.67(23.67-32.08)	128(107-151)	24.84(20.75-29.24)	-0.47(-1.15 to 0.22)	1005(873-1151)	496.01(430.7-567.88)	2051(1743-2369)	392.94(333.96-453.79)	-0.91(-1.61 to -0.22)
Mexico	1466(1401-1527)	13.6(12.96-14.18)	3059(2637-3507)	8.91(7.69-10.2)	-1.52(-1.78 to -1.26)	29392(28204-30558)	259.44(248.41-269.96)	50426(43368-58203)	143.31(123.37-165.2)	-2.1(-2.38 to -1.82)

location	Incidence					DALYs				
	Num in 1990	ASR in 1990	Num in 2021	ASR in 2021	AAPC	Num in 1990	ASR in 1990	Num in 2021	ASR in 2021	AAPC
Micronesia (Federated States of)	2(1-3)	13.89(8.93-20.98)	3(2-4)	14.11(9.05-21.12)	0.02(-0.04 to 0.07)	44(28-67)	330.42(209.14-504.7)	69(43-105)	301.76(191.86-456.13)	-0.29(-0.33 to -0.25)
Monaco	9(6-14)	57.11(36.71-84.26)	11(7-15)	45.23(29.76-66.29)	-0.7(-0.97 to -0.42)	97(63-140)	603.52(393.49-878.09)	89(60-128)	389.42(263.65-567.81)	-1.43(-1.5 to -1.35)
Mongolia	51(34-72)	17.66(11.94-25.05)	82(55-115)	12.28(8.27-17.36)	-1.12(-1.32 to -0.92)	1240(838-1759)	417.95(283.05-592.47)	1833(1235-2578)	252.51(170.44-355.17)	-1.57(-1.77 to -1.38)
Montenegro	75(58-97)	41.76(32.09-54.18)	123(92-165)	45.87(33.97-61.61)	0.59(0.28 to 0.9)	1276(988-1627)	703.56(544.8-897.94)	1725(1291-2309)	646.01(481.84-867.41)	-0.08(-0.29 to 0.14)
Morocco	587(388-851)	15.07(9.95-21.84)	1605(1001-2419)	16.51(10.32-24.8)	0.33(0.25 to 0.41)	14323(9464-20694)	356.43(235.7-514.9)	32572(20299-49394)	323.85(202.44-489.63)	-0.28(-0.33 to -0.23)
Mozambique	210(136-309)	12.84(8.38-18.75)	443(281-668)	14.3(9.2-21.29)	0.44(0.33 to 0.54)	5354(3421-7906)	305.23(196.65-448.57)	11090(7007-16843)	331.8(211.87-498.37)	0.36(0.26 to 0.46)
Myanmar	1264(766-1958)	20.07(12.33-30.8)	2140(1366-3180)	15.99(10.27-23.66)	-0.75(-0.79 to -0.72)	31874(19199-49855)	479.52(291.58-744.52)	45622(28928-68133)	323.96(206.59-481.91)	-1.28(-1.33 to -1.24)
Namibia	58(39-83)	31.7(21.45-45.4)	148(93-218)	37.31(23.87-54.68)	0.65(0.51 to 0.79)	1371(921-1977)	718.05(483.91-1033.2)	3197(2002-4750)	764.33(483.96-1127.9)	0.32(0.17 to 0.46)
Nauru	0(0-0)	16.75(9.82-27.02)	0(0-0)	16.32(9.13-27.02)	-0.11(-0.14 to -0.07)	5(3-9)	382.82(219.53-626.09)	6(3-10)	345.59(192.3-579.21)	-0.33(-0.4 to -0.26)

location	Incidence					DALYs				
	Num in 1990	ASR in 1990	Num in 2021	ASR in 2021	AAPC	Num in 1990	ASR in 1990	Num in 2021	ASR in 2021	AAPC
Nepal	899(557-1375)	34.66(21.45-52.66)	2155(1368-3232)	33.75(21.56-50.39)	-0.12(-0.21 to -0.02)	22593(13909-34623)	813.13(501.3-1239.15)	44337(27938-67492)	668.46(423.49-1013.42)	-0.67(-0.77 to -0.56)
Netherlands	1446(1187-1759)	27.63(22.67-33.66)	2038(1614-2515)	21.67(17.19-26.76)	-0.82(-1.01 to -0.63)	15318(12895-18157)	299.15(251.72-354.88)	16180(13281-19335)	177.16(145.67-211.82)	-1.56(-1.71 to -1.41)
New Zealand	320(254-398)	30.17(23.91-37.53)	543(425-680)	23.72(18.6-29.7)	-0.68(-1.17 to -0.2)	2743(2282-3274)	263.22(218.82-314.4)	3309(2709-3967)	148.28(121.55-177.77)	-1.75(-2.1 to -1.39)
Nicaragua	32(23-44)	8.17(5.82-11.13)	93(63-134)	7.09(4.79-10.2)	-0.5(-0.93 to -0.07)	682(487-928)	164.94(117.64-224.22)	1656(1120-2378)	122.89(83.16-176.19)	-1.01(-1.21 to -0.81)
Niger	48(30-72)	6.37(4.04-9.52)	169(99-268)	7.65(4.54-12.07)	0.68(0.53 to 0.83)	1238(782-1853)	151.89(96.38-226.78)	4029(2323-6479)	168.91(98.62-270.01)	0.42(0.16 to 0.68)
Nigeria	1307(935-1735)	10.62(7.65-14.01)	2608(1787-3596)	10.14(7.13-13.78)	-0.25(-0.44 to -0.05)	35013(24939-46899)	271.64(194.45-362.36)	65253(44168-90227)	233.39(161.26-319.36)	-0.51(-0.71 to -0.31)
Niue	0(0-0)	12.27(8.06-17.88)	0(0-0)	13.65(8.92-20)	0.32(0.27 to 0.36)	1(1-2)	253.95(163.75-373.37)	2(1-2)	245.99(158.83-365.01)	-0.12(-0.15 to -0.09)
North Macedonia	142(110-183)	26.66(20.57-34.35)	268(191-365)	28.83(20.63-39.15)	0.41(0.2 to 0.62)	3109(2428-3996)	567.95(442.88-731.26)	4414(3194-6030)	471.12(341.39-642.83)	-0.52(-0.73 to -0.31)
Northern Mariana Islands	1(1-2)	27.68(18.3-40.95)	7(5-10)	49.27(34.34-67.84)	1.88(1.69 to 2.06)	28(18-42)	494.58(321.86-739.51)	122(85-171)	752.94(525.07-1044)	1.48(1.21 to 1.75)

location	Incidence					DALYs				
	Num in 1990	ASR in 1990	Num in 2021	ASR in 2021	AAPC	Num in 1990	ASR in 1990	Num in 2021	ASR in 2021	AAPC
Norway	353(306-406)	20.29(17.54-23.33)	468(390-549)	17.28(14.46-20.29)	-0.3(-0.7 to 0.1)	3564(3308-3812)	213.97(199.2-228.76)	3297(2961-3576)	124.96(113.15-135.19)	-1.51(-2.04 to -0.97)
Oman	21(13-33)	11.49(7.15-17.52)	58(35-90)	10.23(6.36-15.61)	-0.36(-0.9 to 0.19)	442(268-682)	224.46(137.9-343.84)	854(519-1309)	136.28(84.77-205.65)	-1.6(-1.95 to -1.25)
Pakistan	11385(8637-14839)	74.16(56.12-96.81)	28323(20612-38526)	83.53(60.93-113.19)	0.34(0.24 to 0.44)	261194(198446-339084)	1636.85(1241.14-2127.46)	622660(453427-849159)	1714.33(1252.39-2330.31)	0.12(0.01 to 0.24)
Palau	2(1-3)	81.88(55.38-118.93)	5(3-7)	78.36(52.68-113.09)	-0.17(-0.24 to -0.11)	36(24-51)	1350.31(912.49-1944.88)	82(54-122)	1199.79(798.87-1756.5)	-0.38(-0.46 to -0.31)
Palestine	22(14-33)	9.73(6.37-14.42)	58(40-84)	8.74(6.01-12.47)	-0.35(-0.59 to -0.11)	433(282-646)	184.65(120.53-274.44)	936(641-1347)	130.52(89.93-186.66)	-1.13(-1.34 to -0.92)
Panama	62(53-71)	15.71(13.49-18.13)	142(106-181)	11.78(8.77-14.96)	-0.85(-1.18 to -0.51)	1099(959-1248)	274.85(239.51-312.07)	2058(1532-2585)	170.07(126.58-213.55)	-1.51(-1.77 to -1.26)
Papua New Guinea	47(25-77)	9.55(5.27-15.46)	142(78-231)	10.07(5.66-16.19)	0.21(0.13 to 0.3)	1209(635-2012)	222.99(119.62-367.95)	3482(1869-5793)	223.23(122.71-366.31)	0.08(0.02 to 0.14)
Paraguay	73(51-102)	12.15(8.44-16.9)	284(180-429)	17.68(11.23-26.64)	1.22(0.94 to 1.5)	1495(1035-2091)	243.14(168.47-339.4)	5151(3239-7787)	313.99(197.87-473.78)	0.86(0.67 to 1.05)
Peru	283(205-380)	9.03(6.54-12.11)	739(488-1078)	8.13(5.37-11.84)	-0.22(-0.64 to 0.2)	5836(4261-7814)	180.11(131.54-241.15)	10670(7032-15582)	116.26(76.68-169.58)	-1.37(-1.86 to -0.88)

location	Incidence					DALYs				
	Num in 1990	ASR in 1990	Num in 2021	ASR in 2021	AAPC	Num in 1990	ASR in 1990	Num in 2021	ASR in 2021	AAPC
Philippines	1408(1124-1717)	17.95(14.33-21.91)	3902(3181-4700)	17.14(14.04-20.57)	-0.14(-0.23 to -0.05)	31617(25583-38254)	374.1(302.39-452.95)	83922(68440-101348)	348.28(285.33-419.05)	-0.25(-0.35 to -0.16)
Poland	3517(3286-3759)	29.9(27.9-31.97)	6026(5251-6824)	32.19(28.06-36.47)	0.3(-0.04 to 0.64)	80253(76306-84375)	687.82(653.5-723.59)	98024(87799-108066)	543(486.54-598.79)	-0.72(-1.07 to -0.37)
Portugal	1268(1034-1550)	33.9(27.6-41.47)	1971(1540-2490)	33.3(26.05-42.18)	-0.08(-0.79 to 0.63)	22333(18344-27064)	607.21(497.8-736.52)	20848(16634-25771)	375.18(299.59-464.4)	-1.56(-1.71 to -1.4)
Puerto Rico	291(235-357)	29.61(23.83-36.27)	301(222-394)	16.46(12.11-21.72)	-2.04(-2.52 to -1.57)	4602(3723-5591)	470.52(380.46-571.85)	3616(2706-4700)	209.36(156.04-273.53)	-2.7(-3.17 to -2.23)
Qatar	5(3-8)	19.77(12.93-29.61)	45(26-73)	19.35(11.66-30.4)	-0.49(-1.24 to 0.27)	98(63-150)	331.3(215.95-498.18)	532(306-861)	192.91(116.33-303.11)	-2.11(-2.83 to -1.39)
Republic of Korea	1802(1238-2421)	21.94(15.09-29.39)	4691(3225-6470)	17.9(12.3-24.7)	-0.63(-0.86 to -0.4)	27676(19331-36847)	322.64(225.36-428.46)	31770(22326-42782)	121.81(85.55-164.11)	-3.22(-3.39 to -3.04)
Republic of Moldova	393(341-449)	31.11(27.03-35.59)	514(426-616)	31.52(26.09-37.76)	0.2(-0.42 to 0.83)	8892(7806-10096)	694.25(609.13-788.56)	9041(7609-10669)	561.81(472.71-662.97)	-0.47(-1.29 to 0.36)
Romania	2016(1647-2440)	25.53(20.83-30.92)	4069(3149-5148)	44.6(34.46-56.48)	1.77(1.4 to 2.14)	44672(36511-53782)	563.1(459.59-678.68)	63420(50099-79092)	713.12(563.04-889.89)	0.62(0.3 to 0.95)
Russian Federation	15887(15236-16543)	31.26(29.94-32.59)	19745(17595-21717)	30.73(27.39-33.79)	0.18(-0.43 to 0.8)	316720(303868-330049)	622.02(596.14-648.88)	275403(245589-303574)	433.2(386.47-477.27)	-0.98(-1.56 to -0.39)

location	Incidence					DALYs				
	Num in 1990	ASR in 1990	Num in 2021	ASR in 2021	AAPC	Num in 1990	ASR in 1990	Num in 2021	ASR in 2021	AAPC
Rwanda	242(159-352)	30.3(20.04-44)	385(238-599)	21.72(13.62-33.42)	-1.22(-1.42 to -1.02)	6532(4267-9549)	777.37(509.65-1133.92)	9266(5628-14592)	489.12(300.66-763.02)	-1.63(-1.84 to -1.41)
Saint Kitts and Nevis	2(2-2)	20.14(17.29-23.26)	4(3-5)	20.38(15.81-25.62)	0.2(-0.64 to 1.05)	40(34-46)	421.86(364.14-486.9)	77(59-97)	365.07(283.87-457.02)	-0.34(-1.01 to 0.34)
Saint Lucia	7(6-8)	29.1(25.16-33.47)	17(13-22)	25.35(19.44-32.09)	-0.36(-0.52 to -0.21)	131(114-150)	566.66(491.41-647.63)	304(235-384)	446.13(345.38-562.81)	-0.63(-0.79 to -0.47)
Saint Vincent and the Grenadines	6(5-7)	30.61(26.17-35.66)	13(10-15)	31.59(25.89-38.2)	0.15(-0.49 to 0.8)	111(96-129)	586.8(504.66-678.63)	236(196-284)	586.92(487.3-704.67)	0.01(-0.54 to 0.57)
Samoa	3(2-4)	12.59(8.33-18.59)	5(3-8)	12.28(7.94-18.29)	-0.08(-0.11 to -0.05)	74(48-111)	297.15(194.46-443.29)	117(75-176)	269.59(173.09-404.19)	-0.29(-0.34 to -0.25)
San Marino	4(2-5)	39.38(26.04-57.39)	4(2-6)	20.38(10.82-33.74)	-2.42(-3.03 to -1.8)	37(25-52)	408.43(275.75-583.82)	32(18-53)	180.52(96.95-298.33)	-2.91(-3.23 to -2.58)
Sao Tome and Principe	1(0-1)	2.95(1.89-4.32)	1(1-2)	3.75(2.39-5.65)	0.8(0.57 to 1.04)	12(8-18)	65.92(42.43-96.93)	23(15-35)	74.86(47.96-111.88)	0.47(0.14 to 0.8)
Saudi Arabia	233(150-345)	14.6(9.45-21.41)	841(555-1245)	14.34(9.66-20.78)	-0.06(-0.15 to 0.04)	5583(3548-8365)	322.46(206.73-478.54)	14778(9714-21945)	219.66(147.35-319.25)	-1.28(-1.46 to -1.11)
Senegal	66(43-97)	7.57(4.96-11.04)	214(130-328)	10.11(6.2-15.43)	0.95(0.78 to 1.13)	1604(1044-2354)	175.05(114.21-256.3)	4747(2868-7341)	212.56(129.17-327)	0.63(0.29 to 0.96)

location	Incidence					DALYs				
	Num in 1990	ASR in 1990	Num in 2021	ASR in 2021	AAPC	Num in 1990	ASR in 1990	Num in 2021	ASR in 2021	AAPC
Serbia	1108(760-1578)	34.2(23.47-48.73)	1561(1058-2195)	36.72(24.78-51.74)	0.18(0.01 to 0.36)	22044(15145-31405)	658.35(452.34-938.63)	20911(14184-29074)	504.26(340.78-703.19)	-0.84(-1.16 to -0.53)
Seychelles	8(5-12)	53.45(36.04-76.34)	19(13-28)	58.61(38.91-84.45)	0.18(-0.41 to 0.78)	162(109-233)	1075.71(721.15-1541.5)	332(219-480)	968.08(642.13-1394.26)	-0.44(-1 to 0.13)
Sierra Leone	39(25-58)	7.14(4.55-10.61)	93(58-142)	9.11(5.77-13.81)	0.87(0.74 to 1.01)	935(590-1405)	167.11(105.69-251.01)	2170(1353-3344)	199.89(125.97-306.45)	0.68(0.53 to 0.82)
Singapore	250(202-306)	39.94(32.19-49.02)	565(430-723)	23.83(18.14-30.52)	-1.45(-1.81 to -1.09)	5002(4126-6032)	756.46(622.54-913.71)	6521(5005-8284)	272.93(209.38-346.85)	-3.09(-3.25 to -2.92)
Slovakia	800(588-1068)	50.21(36.87-67.05)	972(645-1405)	38.7(25.64-56.02)	-0.89(-1.08 to -0.69)	15321(11236-20554)	978.01(716.4-1314.29)	14497(9531-20860)	594.43(389.64-856.73)	-1.5(-1.74 to -1.26)
Slovenia	239(192-294)	35.16(28.18-43.21)	309(233-399)	27.65(20.82-35.77)	-0.8(-0.93 to -0.68)	3804(3128-4581)	560.42(460.35-675.47)	2858(2244-3588)	263.24(206.31-331.47)	-2.46(-2.84 to -2.08)
Solomon Islands	5(2-7)	12.05(6.41-19.27)	12(8-19)	12.8(7.86-19.78)	0.19(0.06 to 0.33)	119(59-196)	289.5(148.78-472.24)	312(189-488)	291.43(177.52-453.48)	0.04(-0.13 to 0.2)
Somalia	160(95-252)	22.96(13.93-35.64)	322(192-510)	18.68(11.34-29.06)	-0.63(-0.68 to -0.58)	4560(2683-7228)	592.34(355.4-927.29)	8970(5295-14454)	477.19(287.36-756.47)	-0.68(-0.73 to -0.62)
South Africa	1287(939-1704)	22.58(16.45-29.94)	2856(2449-3296)	21.83(18.72-25.17)	-0.15(-0.68 to 0.38)	28751(21215-38029)	487.87(359.05-646.67)	58474(49957-67634)	431.67(369.14-498.65)	-0.33(-0.54 to -0.12)

location	Incidence					DALYs				
	**Num in 1990**	**ASR in 1990**	**Num in 2021**	**ASR in 2021**	**AAPC**	**Num in 1990**	**ASR in 1990**	**Num in 2021**	**ASR in 2021**	**AAPC**
South Sudan	158(96-248)	22.43(13.77-35.16)	218(131-345)	19.51(11.84-30.5)	-0.45(-0.55 to -0.36)	3970(2394-6317)	549.01(332.25-871.29)	5595(3303-9045)	457.02(272.84-730.66)	-0.6(-0.7 to -0.5)
Spain	8493(6776-10501)	59.2(47.11-73.31)	10229(7795-13156)	41.52(31.68-53.56)	-1.09(-1.28 to -0.91)	97734(80383-117137)	697.39(572.43-836.7)	73223(58049-90612)	306.93(243.95-380.04)	-2.62(-2.79 to -2.44)
Sri Lanka	810(580-1115)	29.48(21.18-40.44)	2718(1535-4283)	36.17(20.61-56.78)	0.77(0.48 to 1.05)	15065(10647-20881)	515.88(366-712.34)	37343(21004-59568)	491.5(277.92-782.07)	-0.04(-0.29 to 0.22)
Sudan	321(188-538)	12.93(7.56-21.86)	594(359-920)	11.46(6.99-17.64)	-0.42(-0.48 to -0.36)	7768(4536-12775)	299.98(175-496.29)	12083(7231-18978)	218.16(131.97-340.3)	-1.06(-1.13 to -0.99)
Suriname	8(6-11)	11.99(8.83-15.97)	20(13-30)	11.17(7.2-16.68)	-0.08(-0.58 to 0.42)	185(134-247)	253.76(185.57-338.54)	425(272-636)	231.01(147.88-345.46)	-0.17(-0.66 to 0.32)
Sweden	661(557-776)	16.87(14.26-19.84)	938(756-1135)	16.45(13.33-19.92)	0.18(-0.36 to 0.72)	6110(5356-6904)	162.88(143.11-183.93)	6631(5512-7787)	121.37(101.5-142.56)	-0.7(-1.08 to -0.32)
Switzerland	693(556-855)	26.27(21.04-32.41)	893(686-1132)	19.25(14.84-24.42)	-0.96(-1.33 to -0.59)	8207(6778-9823)	318.83(263.16-381.8)	7321(5839-9011)	161.81(129.71-199.28)	-2.17(-2.44 to -1.89)
Syrian Arab Republic	123(87-171)	8.9(6.26-12.37)	352(235-503)	9.72(6.58-13.69)	0.24(-0.2 to 0.68)	2606(1843-3605)	177.31(125.53-245.51)	5242(3574-7470)	136.24(93.99-191.99)	-0.87(-1.07 to -0.68)
Taiwan (Province of China)	1902(1581-2266)	42.09(34.99-50.16)	7448(5690-9604)	66.22(50.58-85.42)	1.52(1.29 to 1.75)	35412(30126-41336)	767.14(652.28-895.83)	86703(68563-107566)	776.31(614.83-961.79)	0.15(0.02 to 0.28)

location	Incidence					DALYs				
	Num in 1990	ASR in 1990	Num in 2021	ASR in 2021	AAPC	Num in 1990	ASR in 1990	Num in 2021	ASR in 2021	AAPC
Tajikistan	89(60-128)	11.87(7.86-17.13)	120(78-174)	7.3(4.75-10.6)	-1.66(-2.07 to -1.25)	2171(1461-3090)	278.68(186.63-398.22)	2887(1876-4247)	162.09(105.06-237.37)	-1.82(-2.21 to -1.43)
Thailand	3241(2336-4386)	33.9(24.47-45.78)	9742(6490-13938)	32.01(21.35-45.76)	-0.17(-0.47 to 0.14)	63862(45943-86445)	625.15(450.62-844.59)	137080(91480-196492)	448.07(299.27-641.73)	-1.18(-1.37 to -0.99)
Timor-Leste	11(7-18)	14.99(9.26-23.2)	36(23-55)	15.56(9.87-23.57)	0.1(0.01 to 0.2)	292(174-468)	348.9(212.07-549.02)	787(493-1211)	328.86(206.53-504.62)	-0.22(-0.31 to -0.13)
Togo	27(18-39)	7.93(5.25-11.62)	118(72-181)	10.9(6.75-16.63)	1.02(0.87 to 1.16)	652(426-971)	181.7(119.48-269.56)	2777(1681-4274)	235.49(143.94-359.51)	0.85(0.79 to 0.9)
Tokelau	0(0-0)	12.68(8.01-19.08)	0(0-0)	12.94(8.19-19.43)	0.06(0.02 to 0.09)	1(1-2)	276.55(171.1-421.78)	1(1-1)	239.78(149.36-364.1)	-0.5(-0.59 to -0.41)
Tonga	2(1-3)	10.76(6.56-16.6)	3(2-4)	12.09(7.53-18.5)	0.36(0.12 to 0.6)	35(21-55)	220.66(133.75-344.43)	51(31-79)	225.71(138.35-347.11)	0.11(-0.12 to 0.33)
Trinidad and Tobago	36(31-42)	16.17(14.04-18.6)	74(54-98)	13.67(9.95-18.11)	-0.45(-0.74 to -0.16)	717(627-817)	315.44(275.78-359.23)	1325(967-1755)	246.23(179.57-326.45)	-0.66(-1 to -0.32)
Tunisia	378(250-551)	27.37(18.16-39.82)	1035(624-1616)	27.83(16.81-43.31)	0(-0.09 to 0.09)	8068(5322-11787)	561.35(370.78-819.03)	16407(9957-25358)	432.49(262.99-667.1)	-0.89(-0.97 to -0.82)
Turkey	2081(1342-3130)	22.01(14.24-33.07)	4626(3076-6684)	17.82(11.88-25.69)	-0.62(-1 to -0.24)	46400(29810-69824)	466.72(301.13-701.29)	63437(42467-90943)	240.46(161.26-344.16)	-2.13(-2.38 to -1.88)

location	Incidence					DALYs				
	Num in 1990	ASR in 1990	Num in 2021	ASR in 2021	AAPC	Num in 1990	ASR in 1990	Num in 2021	ASR in 2021	AAPC
Turkmenistan	118(103-133)	21.93(19.11-24.88)	191(142-254)	16.45(12.33-21.76)	-0.92(-1.45 to -0.4)	2785(2443-3124)	499.51(437.54-561.11)	4058(3055-5450)	330.53(250.11-441.24)	-1.3(-1.82 to -0.78)
Tuvalu	0(0-0)	11.98(7.85-17.62)	0(0-1)	12.91(8.65-18.8)	0.24(0.21 to 0.27)	6(4-8)	283.85(183.09-421.33)	8(5-12)	268.03(177.39-394.53)	-0.15(-0.2 to -0.11)
Uganda	555(365-806)	30.83(20.39-44.57)	1239(783-1878)	29.69(19.02-44.61)	-0.22(-0.35 to -0.1)	14443(9449-21115)	763.71(501.54-1112.4)	30847(19128-47322)	686.57(431.34-1044.4)	-0.44(-0.56 to -0.32)
Ukraine	6320(5039-8005)	31.67(25.23-40.21)	6468(4196-9354)	31.74(20.48-46.04)	0.18(-0.57 to 0.94)	118677(95085-150081)	597.78(478.4-758.36)	98036(63264-143402)	495.13(318.06-725.94)	-0.57(-1.2 to 0.06)
United Arab Emirates	22(13-37)	19.62(11.5-31.58)	160(102-241)	17(11.32-24.71)	-0.61(-1.53 to 0.31)	506(281-857)	382.24(220.76-624.88)	2946(1868-4433)	237.55(158.13-346.71)	-1.52(-2.11 to -0.93)
United Kingdom	6133(5797-6438)	26.2(24.79-27.51)	9970(9144-10634)	29.87(27.61-31.79)	0.3(0.03 to 0.57)	60946(58436-63320)	268.23(257.45-278.65)	71095(66487-74897)	218.77(206.01-229.92)	-0.75(-1.02 to -0.47)
United Republic of Tanzania	751(479-1118)	24.63(15.84-36.46)	1409(851-2219)	19.59(12.05-30.48)	-0.76(-0.82 to -0.7)	18845(11853-28474)	589.54(373.19-886.48)	33680(20024-54191)	440.08(265.7-699.81)	-0.96(-1.03 to -0.88)
United States of America	33307(31388-34819)	40.19(37.97-41.98)	49499(45604-52434)	31.37(29.01-33.19)	-0.82(-0.93 to -0.71)	271965(259134-282554)	337.33(322.14-350.14)	314443(293600-331319)	203.75(190.93-214.4)	-1.69(-1.77 to -1.6)
United States Virgin Islands	5(3-7)	19.31(13.12-27.67)	6(4-9)	11.93(7.32-18.45)	-1.26(-1.99 to -0.53)	89(60-128)	360.29(243.68-517.68)	94(58-145)	199.33(122.41-308.75)	-1.56(-2.31 to -0.8)

location	Incidence					DALYs				
	Num in 1990	ASR in 1990	Num in 2021	ASR in 2021	AAPC	Num in 1990	ASR in 1990	Num in 2021	ASR in 2021	AAPC
Uruguay	438(353-541)	41.57(33.43-51.36)	451(354-562)	31.52(24.72-39.36)	-0.93(-1.02 to -0.83)	7667(6230-9373)	738.41(599.14-903.8)	6041(4867-7393)	436.88(351.82-535.37)	-1.7(-1.79 to -1.61)
Uzbekistan	491(379-621)	15.35(11.79-19.48)	845(638-1103)	11.35(8.59-14.75)	-0.98(-1.22 to -0.75)	11454(8984-14359)	349.07(273.1-438.29)	17889(13548-23465)	224.88(170.86-293.88)	-1.48(-1.68 to -1.27)
Vanuatu	2(1-3)	10.71(6.59-16.67)	5(3-8)	10.92(7.17-16.26)	0.07(-0.13 to 0.27)	45(27-72)	247.56(149.17-392.9)	127(82-192)	242.31(157.51-364.37)	-0.04(-0.23 to 0.15)
Venezuela (Bolivarian Republic of)	478(421-538)	18.92(16.64-21.31)	1453(1058-1948)	17.57(12.84-23.47)	-0.3(-0.71 to 0.1)	9722(8642-10848)	371.34(329.56-414.48)	25136(18311-33615)	297.1(216.98-396.4)	-0.83(-1.15 to -0.5)
Viet Nam	3155(2139-4484)	28.8(19.56-40.89)	10192(6497-15208)	35.52(22.86-52.64)	0.73(0.64 to 0.83)	69406(46668-99510)	623.29(419.89-893.29)	180703(113936-273365)	607.13(386.03-912.51)	-0.03(-0.12 to 0.07)
Yemen	195(112-312)	14.49(8.36-23.13)	512(294-802)	13.78(7.99-21.57)	-0.17(-0.26 to -0.07)	4926(2811-7913)	344.05(197.28-551.34)	11197(6358-17513)	281.92(161.52-439.16)	-0.64(-0.76 to -0.52)
Zambia	191(127-274)	23.84(16.01-34.17)	679(284-1318)	33.18(14.72-62.54)	1.35(1.05 to 1.66)	4993(3318-7223)	585.35(390.73-844.86)	17278(6826-34239)	782.26(325.89-1516.65)	1.25(0.93 to 1.57)
Zimbabwe	197(134-278)	17.55(12.01-24.74)	424(281-617)	21.4(14.33-30.81)	0.63(0.33 to 0.94)	4609(3123-6564)	391.17(265.91-555.33)	10705(7040-15625)	503.4(334.71-728.45)	0.85(0.64 to 1.06)

S5 Table: Incidence and DALYs of larynx cancer, nasopharynx cancer, and lip and oral cavity cancer in middle-aged and older adults, and their average annual percentage changes from 1990 to 2021 at global and 21 GBD regions.

cause	location	Incidence					DALYs				
		Num in 1990	ASR in 1990	Num in 2021	ASR in 2021	AAPC	Num in 1990	ASR in 1990	Num in 2021	ASR in 2021	AAPC
Larynx cancer	Global	117909(111050-124774)	10.7(10.06-11.33)	192593(177458-208729)	8.02(7.38-8.69)	-0.93(-0.98 to -0.87)	2238987(2088364-2393272)	199.51(185.93-213.31)	2909562(2680707-3165879)	120.48(110.95-131.09)	-1.6(-1.66 to -1.54)
Larynx cancer	High SDI	37577(35569-39597)	12.75(12.07-13.45)	45565(42067-48452)	8.37(7.76-8.89)	-1.38(-1.52 to -1.24)	444436(421873-467528)	153.14(145.32-161.15)	364270(338277-386593)	68.15(63.57-72.21)	-2.6(-2.66 to -2.53)
Larynx cancer	High-middle SDI	37295(35058-39619)	13.28(12.46-14.13)	49091(43448-55109)	8.84(7.82-9.93)	-1.32(-1.42 to -1.21)	731212(688770-776381)	258.19(242.88-274.4)	639456(572768-709279)	115.46(103.38-128.09)	-2.54(-2.74 to -2.34)
Larynx cancer	Middle SDI	21786(19608-23803)	7.72(6.94-8.43)	53566(47165-60788)	7.07(6.22-8.01)	-0.28(-0.39 to -0.17)	507940(457153-555272)	171.55(154.26-187.41)	891369(793820-1001056)	115.18(102.5-129.32)	-1.28(-1.37 to -1.18)
Larynx cancer	Low-middle SDI	15959(13380-18966)	9.43(7.89-11.21)	34526(30288-39741)	8.64(7.58-9.95)	-0.26(-0.47 to -0.05)	414918(346973-494769)	233.34(194.93-278.25)	776665(677687-899142)	188.37(164.47-217.96)	-0.67(-0.85 to -0.5)

cause	location	Incidence					DALYs				
		Num in 1990	ASR in 1990	Num in 2021	ASR in 2021	AAPC	Num in 1990	ASR in 1990	Num in 2021	ASR in 2021	AAPC
Larynx cancer	Low SDI	5118(3991-6403)	8.13(6.34-10.17)	9593(7997-11360)	6.95(5.8-8.21)	-0.47(-0.56 to -0.38)	136938(106881-171471)	206.21(161.15-258.09)	233951(194583-277196)	160.34(133.7-189.57)	-0.78(-0.85 to -0.7)
Larynx cancer	High-income Asia Pacific	4422(3846-5013)	7.87(6.84-8.91)	5897(4904-6881)	4.8(4-5.63)	-1.7(-2.22 to -1.19)	40350(34236-46411)	71.58(60.79-82.22)	31154(26525-35759)	25.08(21.4-29.01)	-3.32(-3.58 to -3.05)
Larynx cancer	High-income North America	13111(12444-13694)	14.38(13.67-15.02)	17419(16222-18383)	9.84(9.19-10.38)	-1.25(-1.43 to -1.06)	117073(111737-121930)	130.48(124.66-135.83)	121532(113904-128191)	69.65(65.43-73.4)	-2.1(-2.22 to -1.97)
Larynx cancer	Western Europe	25783(23825-27860)	17.27(15.95-18.69)	24383(21789-26906)	10.58(9.5-11.68)	-1.59(-1.88 to -1.31)	333742(310793-357358)	227.91(212.08-244.36)	205399(185260-224145)	90.1(81.71-98.2)	-2.93(-3.04 to -2.82)
Larynx cancer	Australasia	467(401-547)	7.38(6.33-8.67)	570(465-690)	4.07(3.31-4.95)	-2.02(-2.46 to -1.56)	6843(5781-8093)	108.87(91.82-128.86)	5718(4673-6924)	40.74(33.31-49.4)	-3.18(-3.64 to -2.73)

cause	location	Incidence					DALYs				
		Num in 1990	ASR in 1990	Num in 2021	ASR in 2021	AAPC	Num in 1990	ASR in 1990	Num in 2021	ASR in 2021	AAPC
Larynx cancer	Andean Latin America	235(189-289)	4.41(3.55-5.42)	451(332-597)	2.83(2.09-3.74)	-1.4(-2.05 to -0.74)	5317(4301-6558)	96.6(78.17-119.08)	7997(5895-10583)	49.69(36.66-65.68)	-2.13(-2.78 to -1.47)
Larynx cancer	Tropical Latin America	2915(2692-3151)	11.42(10.52-12.34)	7498(6784-8222)	10.44(9.44-11.45)	-0.26(-0.61 to 0.09)	68794(63645-74238)	260.8(241-281.41)	144307(131715-157331)	199.52(181.99-217.57)	-0.84(-1.06 to -0.63)
Larynx cancer	Central Latin America	1750(1648-1850)	8.14(7.64-8.61)	2943(2542-3394)	4.32(3.73-4.97)	-2.11(-2.57 to -1.65)	37585(35454-39703)	168.16(158.36-177.64)	52137(45146-60182)	75.42(65.34-86.98)	-2.61(-3.06 to -2.16)
Larynx cancer	Southern Latin America	1839(1554-2150)	14.37(12.14-16.81)	1802(1512-2128)	7.62(6.39-8.99)	-1.9(-2.08 to -1.72)	37735(31983-44102)	294.43(249.42-344.24)	27760(23492-32697)	118.32(100.17-139.39)	-2.8(-2.99 to -2.62)
Larynx cancer	Caribbean	922(806-1059)	13.13(11.47-15.08)	2159(1747-2654)	14.51(11.75-17.83)	0.38(-0.25 to 1.02)	17752(15531-20445)	250.43(219.05-288.42)	35412(28915-43292)	237.66(194.11-290.47)	-0.09(-0.72 to 0.54)
Larynx cancer	Central Europe	6985(6513-7498)	16.7(15.55-17.94)	8853(7927-9842)	15.7(14.05-17.48)	-0.19(-0.36 to -0.02)	152619(142746-163375)	366.46(342.37-392.55)	135345(122212-149371)	243.02(219.12-268.6)	-1.34(-1.5 to -1.17)

cause	location	Incidence					DALYs				
		Num in 1990	ASR in 1990	Num in 2021	ASR in 2021	AAPC	Num in 1990	ASR in 1990	Num in 2021	ASR in 2021	AAPC
Larynx cancer	Eastern Europe	12381(11655-13219)	15.62(14.68-16.71)	9794(8605-11051)	10.25(8.99-11.58)	-1.27(-1.68 to -0.86)	274769(258984-292520)	348.05(327.7-371.31)	162323(142243-184096)	173.03(151.41-196.57)	-2.17(-2.81 to -1.53)
Larynx cancer	Central Asia	1554(1456-1656)	11.46(10.73-12.21)	1257(1111-1416)	5.25(4.65-5.91)	-2.54(-2.91 to -2.16)	37759(35487-40124)	273.54(256.86-290.88)	26464(23405-29947)	107.67(95.29-121.66)	-3.04(-3.32 to -2.75)
Larynx cancer	North Africa and Middle East	4463(3620-5477)	9.81(7.93-12.1)	11246(9454-13255)	9.15(7.69-10.78)	-0.21(-0.3 to -0.11)	99544(80465-121831)	207.72(167.74-255.25)	172317(144880-203444)	134.24(112.9-158.11)	-1.41(-1.46 to -1.36)
Larynx cancer	South Asia	19220(15908-22949)	11.93(9.83-14.27)	42763(36743-49811)	10.37(8.91-12.07)	-0.4(-0.53 to -0.26)	507403(420818-607358)	298.32(246.63-357.56)	956040(818830-1118633)	225.42(193.12-263.61)	-0.89(-1.1 to -0.68)
Larynx cancer	Southeast Asia	3665(3078-4322)	5.31(4.45-6.26)	10235(8410-12521)	5.54(4.56-6.78)	0.14(0.04 to 0.24)	83126(69816-97892)	114.65(96.19-135.06)	179701(148709-218539)	94.19(78.03-114.47)	-0.63(-0.72 to -0.54)

cause	location	Incidence					DALYs				
		Num in 1990	ASR in 1990	Num in 2021	ASR in 2021	AAPC	Num in 1990	ASR in 1990	Num in 2021	ASR in 2021	AAPC
Larynx cancer	East Asia	14893(12033-17681)	6.31(5.11-7.47)	38785(30025-49393)	6.21(4.82-7.89)	-0.09(-0.23 to 0.05)	331379(266064-394990)	134.19(108.01-159.55)	483179(371483-616155)	76.65(59.06-97.52)	-1.85(-2.02 to -1.67)
Larynx cancer	Oceania	16(11-22)	2.18(1.57-3.06)	36(25-50)	1.94(1.38-2.75)	-0.34(-0.43 to -0.26)	364(255-515)	46.3(32.75-65.3)	790(557-1128)	39.49(27.96-56.27)	-0.5(-0.68 to -0.32)
Larynx cancer	Western Sub-Saharan Africa	1037(806-1308)	4.28(3.35-5.37)	2243(1718-2767)	4.21(3.28-5.15)	-0.05(-0.1 to 0.01)	27305(21094-34659)	107.95(83.84-136.46)	55312(42265-68745)	97.03(75.18-119.58)	-0.34(-0.41 to -0.27)
Larynx cancer	Eastern Sub-Saharan Africa	1300(1007-1610)	6.24(4.84-7.71)	2261(1761-2936)	4.8(3.76-6.17)	-0.84(-0.89 to -0.78)	35126(27209-43621)	159.95(124.2-198.25)	57877(44655-75618)	115.82(89.92-149.84)	-1.04(-1.09 to -0.99)
Larynx cancer	Central Sub-Saharan Africa	372(240-531)	6.01(3.94-8.54)	802(551-1113)	5.21(3.61-7.23)	-0.45(-0.52 to -0.38)	10094(6454-14527)	151.44(98.13-216.81)	20892(14297-29084)	125.69(86.63-174.51)	-0.6(-0.67 to -0.52)

cause	location	Incidence					DALYs				
		Num in 1990	ASR in 1990	Num in 2021	ASR in 2021	AAPC	Num in 1990	ASR in 1990	Num in 2021	ASR in 2021	AAPC
Larynx cancer	Southern Sub-Saharan Africa	577(461-767)	7.66(6.13-10.18)	1196(1026-1386)	7.23(6.21-8.36)	-0.23(-0.67 to 0.22)	14306(11475-18897)	183.98(147.58-243.35)	27906(23784-32603)	163.45(139.58-190.49)	-0.45(-0.91 to 0.02)
Nasopharynx cancer	Global	52454(46607-58419)	4.68(4.16-5.21)	85245(73448-99102)	3.53(3.04-4.11)	-0.91(-1.03 to -0.8)	1480051(1311369-1648731)	130.06(115.26-144.85)	1813608(1592547-2056015)	74.86(65.72-84.86)	-1.79(-1.9 to -1.69)
Nasopharynx cancer	High SDI	6450(6075-6831)	2.27(2.14-2.4)	8691(7801-9594)	1.74(1.57-1.92)	-0.88(-1.04 to -0.71)	146541(138140-155468)	52.16(49.17-55.34)	151791(137110-167262)	30.67(27.78-33.8)	-1.73(-1.84 to -1.63)
Nasopharynx cancer	High-middle SDI	16514(13570-20090)	5.95(4.89-7.24)	28653(22178-37162)	5.29(4.09-6.86)	-0.39(-0.57 to -0.21)	455241(375560-553535)	162.5(134.09-197.64)	489681(393579-615963)	89.86(72.2-113.06)	-1.92(-2.11 to -1.74)
Nasopharynx cancer	Middle SDI	20827(17949-23891)	6.96(6-7.97)	32379(27271-38376)	4.14(3.48-4.9)	-1.66(-1.82 to -1.51)	606826(521884-696767)	194.83(167.69-223.42)	706598(606317-821422)	88.84(76.21-103.25)	-2.54(-2.68 to -2.4)
Nasopharynx cancer	Low-middle SDI	6234(5216-7395)	3.46(2.89-4.11)	11471(9987-13194)	2.76(2.41-3.17)	-0.71(-0.82 to -0.6)	194726(163141-231246)	103.52(86.72-122.94)	340931(295592-393444)	79.49(69.02-91.65)	-0.84(-0.93 to -0.74)

cause	location	Incidence					DALYs				
		Num in 1990	ASR in 1990	Num in 2021	ASR in 2021	AAPC	Num in 1990	ASR in 1990	Num in 2021	ASR in 2021	AAPC
Nasopharynx cancer	Low SDI	2407(1913-2937)	3.56(2.84-4.35)	4009(3185-5030)	2.69(2.14-3.36)	-0.9(-1.05 to -0.74)	76073(60429-92977)	107.75(85.69-131.69)	123514(97488-155972)	78.43(62.15-98.64)	-1.03(-1.16 to -0.9)
Nasopharynx cancer	High-income Asia Pacific	666(611-730)	1.18(1.08-1.29)	1316(1144-1469)	1.15(1.01-1.29)	-0.16(-0.52 to 0.2)	17686(16186-19449)	30.88(28.23-33.96)	26654(23598-29518)	24.77(22.1-27.48)	-0.73(-0.92 to -0.54)
Nasopharynx cancer	High-income North America	1580(1491-1664)	1.83(1.73-1.92)	2068(1913-2206)	1.28(1.19-1.36)	-1.17(-1.36 to -0.98)	27358(26037-28628)	32.07(30.57-33.55)	29480(27581-31246)	18.24(17.11-19.31)	-1.82(-2.05 to -1.59)
Nasopharynx cancer	Western Europe	2734(2501-2985)	1.91(1.74-2.09)	2586(2252-2978)	1.21(1.06-1.4)	-1.48(-1.65 to -1.31)	63170(58134-68497)	44.95(41.33-48.79)	44356(39281-50399)	21.06(18.74-23.92)	-2.48(-2.65 to -2.31)
Nasopharynx cancer	Australasia	185(150-225)	3.01(2.43-3.67)	234(176-302)	1.81(1.36-2.33)	-1.71(-2.23 to -1.19)	2758(2294-3259)	45.19(37.58-53.39)	2718(2042-3491)	21.17(15.94-27.16)	-2.5(-2.76 to -2.23)

cause	location	Incidence					DALYs				
		Num in 1990	ASR in 1990	Num in 2021	ASR in 2021	AAPC	Num in 1990	ASR in 1990	Num in 2021	ASR in 2021	AAPC
Nasopharynx cancer	Andean Latin America	25(20-31)	0.46(0.37-0.56)	62(46-82)	0.39(0.29-0.51)	-0.48(-0.93 to -0.03)	698(567-850)	12.21(9.92-14.87)	1563(1158-2072)	9.56(7.08-12.65)	-0.74(-1.16 to -0.32)
Nasopharynx cancer	Tropical Latin America	147(133-162)	0.55(0.5-0.61)	456(407-506)	0.63(0.56-0.7)	0.44(0.12 to 0.75)	4575(4153-5048)	16.65(15.09-18.38)	13118(11751-14532)	18(16.11-19.94)	0.29(-0.06 to 0.64)
Nasopharynx cancer	Central Latin America	153(142-165)	0.69(0.63-0.74)	377(322-437)	0.54(0.47-0.63)	-0.82(-0.93 to -0.7)	4266(3957-4592)	18.22(16.88-19.62)	9818(8395-11417)	13.89(11.88-16.14)	-0.96(-1.08 to -0.84)
Nasopharynx cancer	Southern Latin America	138(114-165)	1.1(0.91-1.32)	124(95-161)	0.53(0.4-0.69)	-2.28(-2.6 to -1.96)	3803(3161-4552)	30.02(24.94-35.95)	3016(2304-3917)	13.11(10.01-17.04)	-2.59(-2.88 to -2.3)
Nasopharynx cancer	Caribbean	96(82-111)	1.38(1.19-1.61)	240(195-293)	1.61(1.31-1.96)	0.59(0.42 to 0.76)	2582(2214-3004)	36.52(31.32-42.51)	6226(5056-7635)	41.69(33.87-51.11)	0.52(0.31 to 0.73)
Nasopharynx cancer	Central Europe	480(442-520)	1.18(1.08-1.28)	694(607-788)	1.28(1.12-1.45)	0.25(0.12 to 0.38)	13994(12906-15164)	34.31(31.6-37.22)	18032(15783-20575)	34.39(30.06-39.3)	-0.02(-0.16 to 0.12)

cause	location	Incidence					DALYs				
		Num in 1990	ASR in 1990	Num in 2021	ASR in 2021	AAPC	Num in 1990	ASR in 1990	Num in 2021	ASR in 2021	AAPC
Nasopharynx cancer	Eastern Europe	735(666-859)	0.95(0.86-1.11)	855(743-991)	0.93(0.81-1.08)	0.12(-0.62 to 0.86)	22420(20314-26367)	29.04(26.3-34.26)	24746(21462-28890)	27.64(23.95-32.33)	0.03(-0.72 to 0.78)
Nasopharynx cancer	Central Asia	134(116-159)	1.02(0.88-1.23)	257(215-308)	1.08(0.91-1.29)	0.2(0.04 to 0.36)	3980(3471-4646)	29.38(25.55-34.6)	7547(6298-9155)	30.23(25.26-36.58)	0.07(-0.09 to 0.24)
Nasopharynx cancer	North Africa and Middle East	1136(937-1364)	2.38(1.95-2.87)	2368(1948-2852)	1.78(1.47-2.15)	-0.92(-1.02 to -0.83)	33878(28044-40685)	67.07(55.39-80.76)	64934(53270-78523)	46.39(38.07-56)	-1.2(-1.28 to -1.11)
Nasopharynx cancer	South Asia	6930(5774-8203)	4(3.32-4.74)	12333(10664-14172)	2.89(2.5-3.32)	-1.02(-1.17 to -0.88)	219479(183555-259804)	120.88(100.85-143.14)	366815(316207-422546)	83.6(72.12-96.24)	-1.17(-1.3 to -1.04)
Nasopharynx cancer	Southeast Asia	4250(3584-5004)	5.79(4.88-6.82)	9981(8407-11758)	5.15(4.34-6.05)	-0.38(-0.43 to -0.32)	126476(106854-149333)	164.3(138.8-193.97)	273394(230597-322200)	136.06(114.92-160.1)	-0.6(-0.65 to -0.55)

cause	location	Incidence					DALYs				
		Num in 1990	ASR in 1990	Num in 2021	ASR in 2021	AAPC	Num in 1990	ASR in 1990	Num in 2021	ASR in 2021	AAPC
Nasopharynx cancer	East Asia	31296(25991-36890)	12.63(10.51-14.86)	47798(37310-60656)	7.56(5.9-9.57)	-1.66(-1.92 to -1.39)	877591(726089-1036604)	340.62(282.29-401.77)	812205(641978-1019268)	127.25(100.6-159.59)	-3.15(-3.45 to -2.85)
Nasopharynx cancer	Oceania	27(18-41)	3.07(2.05-4.58)	58(37-92)	2.5(1.62-3.92)	-0.65(-0.79 to -0.5)	850(552-1293)	89.93(58.95-135.99)	1803(1140-2873)	72.6(46.34-114.9)	-0.67(-0.86 to -0.48)
Nasopharynx cancer	Western Sub-Saharan Africa	455(352-569)	1.79(1.39-2.23)	894(626-1186)	1.49(1.06-1.96)	-0.61(-0.72 to -0.5)	14172(10947-17779)	53.45(41.39-66.9)	28068(19492-37358)	43.91(30.91-58.16)	-0.65(-0.72 to -0.58)
Nasopharynx cancer	Eastern Sub-Saharan Africa	1079(841-1330)	4.86(3.8-5.99)	2074(1516-2770)	4.09(3.01-5.42)	-0.56(-0.62 to -0.5)	34018(26443-41978)	146.44(114.06-180.64)	65033(47005-87514)	120.68(87.89-161.38)	-0.63(-0.68 to -0.57)
Nasopharynx cancer	Central Sub-Saharan Africa	84(59-119)	1.27(0.89-1.82)	190(129-286)	1.15(0.78-1.74)	-0.31(-0.38 to -0.24)	2629(1855-3728)	37.09(26.06-52.78)	5961(4013-9051)	33.22(22.43-50.48)	-0.35(-0.43 to -0.27)

cause	location	Incidence					DALYs				
		**Num in 1990**	**ASR in 1990**	**Num in 2021**	**ASR in 2021**	**AAPC**	**Num in 1990**	**ASR in 1990**	**Num in 2021**	**ASR in 2021**	**AAPC**
Nasopharynx cancer	Southern Sub-Saharan Africa	125(101-153)	1.67(1.35-2.05)	278(241-320)	1.69(1.47-1.94)	0.03(-0.36 to 0.42)	3668(2982-4510)	47.11(38.23-58.02)	8121(6998-9397)	47.38(40.93-54.65)	-0.02(-0.4 to 0.37)
Lip and oral cavity cancer	Global	150252(142269-158086)	13.91(13.12-14.65)	372367(338821-400693)	15.64(14.2-16.83)	0.38(0.27 to 0.49)	2380847(2244248-2531810)	213.82(201.21-227.41)	4898441(4413928-5311561)	203.6(183.29-220.79)	-0.13(-0.17 to -0.09)
Lip and oral cavity cancer	High SDI	52348(49133-55209)	17.77(16.67-18.76)	98417(89222-105266)	17.79(16.3-18.97)	-0.01(-0.17 to 0.16)	500180(473298-525676)	173.52(164.13-182.5)	687322(633489-729253)	129.74(120.6-137.27)	-0.94(-1.11 to -0.77)
Lip and oral cavity cancer	High-middle SDI	32524(30658-34399)	11.88(11.16-12.58)	69021(61778-76193)	12.57(11.24-13.87)	0.25(0.09 to 0.42)	493201(467665-519107)	176.82(167.3-186.25)	751231(677418-827758)	136.94(123.45-150.9)	-0.8(-1.05 to -0.56)
Lip and oral cavity cancer	Middle SDI	28205(26001-30580)	10.14(9.32-11)	100018(88145-112122)	13.31(11.71-14.92)	0.89(0.82 to 0.96)	558111(513789-606335)	188.88(173.6-205.19)	1452004(1284048-1620755)	187.54(165.72-209.26)	-0.02(-0.08 to 0.04)
Lip and oral cavity cancer	Low-middle SDI	29137(25424-33331)	17.39(15.11-19.91)	84368(72770-95983)	21.35(18.41-24.27)	0.67(0.52 to 0.81)	647574(566651-743043)	365.02(318.44-418.99)	1594484(1367444-1819831)	387.28(332.45-441.62)	0.2(0.11 to 0.28)

cause	location	Incidence					DALYs				
		Num in 1990	ASR in 1990	Num in 2021	ASR in 2021	AAPC	Num in 1990	ASR in 1990	Num in 2021	ASR in 2021	AAPC
Lip and oral cavity cancer	Low SDI	7873(6628-9158)	12.75(10.73-14.83)	20228(16789-23882)	14.85(12.35-17.5)	0.51(0.35 to 0.67)	179003(151820-208474)	271.74(230.34-316.41)	409225(339846-483606)	281.06(234.05-331.41)	0.13(-0.02 to 0.28)
Lip and oral cavity cancer	High-income Asia Pacific	5202(4704-5716)	9.4(8.47-10.34)	17167(13899-20100)	13.28(10.98-15.45)	1.06(0.73 to 1.39)	45349(42652-47828)	80.82(75.73-85.34)	100914(86488-111235)	82.89(73.01-90.73)	0.02(-0.31 to 0.35)
Lip and oral cavity cancer	High-income North America	22068(20691-23156)	23.91(22.48-25.07)	34602(31723-36724)	19.43(17.89-20.58)	-0.71(-0.85 to -0.57)	159828(151949-166172)	178.36(169.98-185.3)	196648(183083-207594)	112.92(105.62-118.98)	-1.5(-1.72 to -1.27)
Lip and oral cavity cancer	Western Europe	28876(26374-31552)	19.45(17.75-21.29)	43743(38931-47899)	18.53(16.67-20.23)	-0.17(-0.38 to 0.03)	309014(286086-332408)	214.54(198.48-231.14)	309494(280688-334071)	136.84(125.42-147.25)	-1.47(-1.78 to -1.15)
Lip and oral cavity cancer	Australasia	1514(1259-1814)	23.99(19.92-28.79)	3212(2577-3921)	22.59(18.16-27.57)	-0.21(-0.89 to 0.48)	10011(8662-11513)	160.43(138.73-184.54)	15595(13159-18283)	111.88(94.64-131.03)	-1.2(-1.72 to -0.67)

cause	location	Incidence					DALYs				
		Num in 1990	ASR in 1990	Num in 2021	ASR in 2021	AAPC	Num in 1990	ASR in 1990	Num in 2021	ASR in 2021	AAPC
Lip and oral cavity cancer	Andean Latin America	234(193-286)	4.35(3.58-5.32)	819(622-1072)	5.12(3.89-6.7)	0.6(0.05 to 1.16)	4462(3665-5423)	79.75(65.52-96.96)	11467(8711-14841)	70.62(53.68-91.32)	-0.24(-0.74 to 0.26)
Lip and oral cavity cancer	Tropical Latin America	2968(2731-3217)	11.94(10.93-12.94)	8904(8047-9717)	12.49(11.27-13.64)	0.15(0.09 to 0.22)	57621(53308-62270)	221.05(203.93-238.88)	140477(128842-152143)	194.76(178.39-210.97)	-0.41(-0.48 to -0.34)
Lip and oral cavity cancer	Central Latin America	1297(1217-1371)	6.1(5.7-6.45)	3933(3425-4461)	5.8(5.04-6.57)	-0.24(-0.44 to -0.03)	22431(21232-23592)	100.64(94.96-105.95)	55062(48166-62445)	79.51(69.55-90.09)	-0.9(-1.34 to -0.46)
Lip and oral cavity cancer	Southern Latin America	1232(1051-1439)	9.74(8.3-11.37)	1997(1685-2337)	8.44(7.12-9.88)	-0.41(-0.97 to 0.17)	19416(16839-22358)	152.74(132.38-175.88)	24156(20789-27845)	103.61(89.18-119.49)	-1.07(-1.66 to -0.47)
Lip and oral cavity cancer	Caribbean	962(849-1087)	13.81(12.18-15.6)	1983(1626-2384)	13.34(10.94-16.04)	-0.04(-0.67 to 0.59)	14943(13259-16955)	211.94(188.01-240.42)	27802(22772-33300)	186.53(152.81-223.44)	-0.33(-0.57 to -0.08)
Lip and oral cavity cancer	Central Europe	5730(5338-6141)	14.13(13.14-15.15)	10865(9742-11985)	19.14(17.16-21.14)	0.98(0.71 to 1.24)	105917(99049-112933)	260.74(243.62-278.08)	148413(134731-162486)	274.2(248.91-300.59)	0.14(-0.15 to 0.43)

cause	location	Incidence					DALYs				
		Num in 1990	ASR in 1990	Num in 2021	ASR in 2021	AAPC	Num in 1990	ASR in 1990	Num in 2021	ASR in 2021	AAPC
Lip and oral cavity cancer	Eastern Europe	11726(10984-12719)	15.09(14.12-16.38)	18802(16747-20962)	20.29(18.06-22.61)	1.16(0.43 to 1.89)	191899(180700-207277)	245.89(231.36-265.94)	233967(207528-261555)	256.41(227.19-287.04)	0.32(-0.41 to 1.06)
Lip and oral cavity cancer	Central Asia	1096(990-1208)	8.42(7.57-9.31)	1802(1558-2081)	7.9(6.83-9.09)	-0.22(-0.76 to 0.33)	22199(20248-24360)	164.87(149.93-181.25)	32127(27724-37246)	132.88(114.85-153.68)	-0.61(-1.14 to -0.08)
Lip and oral cavity cancer	North Africa and Middle East	1496(1246-1774)	3.42(2.83-4.07)	5150(4365-6014)	4.28(3.62-4.99)	0.73(0.62 to 0.83)	27572(22998-32940)	58.87(48.92-70.27)	70342(59962-81905)	54.9(46.76-63.76)	-0.24(-0.35 to -0.12)
Lip and oral cavity cancer	South Asia	40293(35578-45427)	25.35(22.25-28.64)	125917(107663-143133)	30.87(26.4-35.11)	0.64(0.46 to 0.82)	898490(795389-1013793)	530.39(467.52-599.12)	2314386(1975425-2636768)	546.84(467.11-623)	0.1(-0.02 to 0.22)
Lip and oral cavity cancer	Southeast Asia	7738(6608-8971)	11.62(9.93-13.48)	24431(20262-29147)	13.82(11.44-16.48)	0.54(0.46 to 0.62)	142337(121254-165752)	201.18(171.65-233.98)	362197(302593-427861)	194.6(162.53-229.57)	-0.11(-0.15 to -0.08)

cause	location	Incidence					DALYs				
		Num in 1990	ASR in 1990	Num in 2021	ASR in 2021	AAPC	Num in 1990	ASR in 1990	Num in 2021	ASR in 2021	AAPC
Lip and oral cavity cancer	East Asia	13436(11428-15562)	5.79(4.93-6.68)	58105(46485-71683)	9.38(7.52-11.54)	1.59(1.4 to 1.77)	251822(212831-294027)	102.61(86.89-119.46)	628960(500356-781354)	100.01(79.68-123.89)	-0.1(-0.3 to 0.1)
Lip and oral cavity cancer	Oceania	46(33-62)	5.92(4.25-7.83)	142(101-194)	6.93(5.01-9.3)	0.49(0.4 to 0.58)	962(661-1318)	112.72(78.6-152.76)	2907(1998-4012)	128.95(90.35-175.79)	0.44(0.3 to 0.59)
Lip and oral cavity cancer	Western Sub-Saharan Africa	807(653-959)	3.4(2.76-4.04)	2364(1858-2911)	4.38(3.51-5.34)	0.81(0.77 to 0.86)	17800(14406-21099)	70.9(57.47-83.98)	48819(38068-60474)	83.7(66.31-102.76)	0.53(0.48 to 0.58)
Lip and oral cavity cancer	Eastern Sub-Saharan Africa	2183(1846-2551)	10.81(9.14-12.62)	5140(4086-6199)	11.27(9.08-13.48)	0.14(0.04 to 0.24)	50012(42234-58651)	232.1(196.28-272.01)	108544(85121-132422)	221.43(175.89-267.84)	-0.15(-0.23 to -0.07)
Lip and oral cavity cancer	Central Sub-Saharan Africa	402(293-557)	6.71(4.86-9.27)	1111(792-1511)	7.47(5.28-10.24)	0.35(0.24 to 0.46)	9445(6822-13194)	143.64(103.73-199.59)	24484(17319-33524)	148.82(105-204.22)	0.11(0.05 to 0.18)

cause	location	Incidence					DALYs				
		Num in 1990	ASR in 1990	Num in 2021	ASR in 2021	AAPC	Num in 1990	ASR in 1990	Num in 2021	ASR in 2021	AAPC
Lip and oral cavity cancer	Southern Sub-Saharan Africa	945(694-1176)	12.73(9.33-15.86)	2177(1879-2490)	13.44(11.61-15.36)	0.21(0.07 to 0.34)	19316(14242-24009)	249.79(183.72-310.84)	41678(35976-47806)	246.05(212.7-281.8)	-0.11(-0.49 to 0.26)

S6 Table: Incidence and DALYs of Larynx cancer in middle-aged and older adults, and their average annual percentage changes in 204 countries/territories from 1990 to 2021.

location	Incidence					DALYs				
	Num in 1990	ASR in 1990	Num in 2021	ASR in 2021	AAPC	Num in 1990	ASR in 1990	Num in 2021	ASR in 2021	AAPC
Afghanistan	225(110-377)	11.62(5.81-19.24)	268(136-442)	10.23(5.39-16.45)	-0.4(-0.47 to -0.34)	5819(2755-9879)	285.13(137.56-479.64)	6851(3412-11446)	232.36(118.96-377.83)	-0.65(-0.72 to -0.59)
Albania	74(53-100)	12.89(9.32-17.4)	134(89-195)	11.13(7.42-16.2)	-0.54(-1.07 to -0.01)	1702(1237-2303)	287.13(208.85-388.27)	2172(1455-3166)	181.95(121.82-266)	-1.53(-2.01 to -1.06)
Algeria	242(162-357)	7.79(5.2-11.49)	625(409-929)	6.59(4.31-9.78)	-0.55(-0.67 to -0.42)	5170(3455-7669)	155.53(103.99-230.64)	9628(6389-14030)	97.05(64.41-141.39)	-1.52(-1.64 to -1.39)
American Samoa	0(0-0)	4.49(2.93-6.66)	0(0-0)	2.28(1.53-3.28)	-2.23(-2.76 to -1.69)	5(3-7)	84.83(55.73-126.24)	5(4-8)	40.28(27.04-57.62)	-2.42(-2.94 to -1.91)
Andorra	2(1-3)	11.84(6.84-19.64)	3(2-5)	7.59(4.15-12.36)	-1.48(-1.94 to -1.03)	21(12-33)	129.38(76.63-209.23)	26(14-42)	61.11(33.92-99.14)	-2.55(-3.04 to -2.07)
Angola	75(43-118)	6.93(4.05-10.79)	188(120-282)	5.72(3.68-8.59)	-0.6(-0.82 to -0.38)	2110(1193-3343)	179.3(103.04-281.92)	4851(3125-7328)	136.43(88.26-205.71)	-0.86(-1.08 to -0.64)
Antigua and Barbuda	1(1-1)	7.73(6.48-9.12)	2(2-3)	7.78(6.37-9.42)	-0.45(-1.95 to 1.08)	21(18-25)	154.75(130.78-181.39)	41(34-49)	133.13(110.55-158.9)	-0.92(-2.38 to 0.57)
Argentina	1393(1126-1695)	15.58(12.59-18.96)	1311(1047-1621)	8.67(6.92-10.73)	-1.72(-1.98 to -1.46)	29177(23676-35400)	326.17(264.59-395.92)	21076(17019-25732)	140.75(113.67-171.89)	-2.56(-2.81 to -2.31)

location	Incidence					DALYs				
	Num in 1990	ASR in 1990	Num in 2021	ASR in 2021	AAPC	Num in 1990	ASR in 1990	Num in 2021	ASR in 2021	AAPC
Armenia	139(127-151)	17.06(15.55-18.65)	104(89-121)	8.55(7.31-9.97)	-2.29(-3.08 to -1.5)	3205(2976-3427)	385.57(356.87-414.15)	1897(1646-2184)	157.91(137.24-181.5)	-2.92(-3.66 to -2.17)
Australia	363(302-440)	6.92(5.74-8.38)	446(352-555)	3.83(3.02-4.78)	-2.04(-2.65 to -1.43)	5989(4937-7223)	114.37(94.19-137.99)	4997(4003-6137)	42.52(34.13-52.27)	-3.21(-3.61 to -2.8)
Austria	345(280-423)	11.54(9.32-14.16)	314(246-393)	6.83(5.35-8.55)	-1.59(-2.68 to -0.48)	5291(4353-6399)	180.86(148.41-218.96)	3289(2642-4007)	72.32(58.1-88.1)	-2.9(-3.75 to -2.05)
Azerbaijan	170(139-204)	11.49(9.37-13.92)	242(175-350)	7.77(5.61-11.16)	-1.23(-1.45 to -1.01)	4330(3557-5199)	284.97(233.29-343.56)	5244(3832-7684)	162.51(118.5-237.37)	-1.78(-1.98 to -1.57)
Bahamas	5(4-6)	11.08(9.37-13.02)	13(10-16)	10.78(8.28-13.93)	0.2(0.04 to 0.35)	105(90-122)	243.49(207.2-282.51)	254(194-328)	209.63(161.35-269.73)	-0.27(-0.44 to -0.1)
Bahrain	6(4-8)	13.58(9.35-19.62)	21(13-34)	8.96(5.66-14.02)	-1.28(-1.76 to -0.81)	116(80-166)	248.43(172.96-355.1)	271(170-435)	105.51(67.38-165.05)	-2.71(-3.12 to -2.31)
Bangladesh	1789(1189-2610)	14.08(9.36-20.56)	3493(2178-5256)	9.17(5.72-13.79)	-1.28(-1.54 to -1.02)	46890(31030-68358)	356.44(235.98-519.73)	74500(46645-114262)	191.1(119.75-292.85)	-1.9(-2.14 to -1.66)
Barbados	5(4-6)	6.37(5.47-7.39)	11(8-14)	7.37(5.43-9.6)	0.65(-0.13 to 1.43)	91(79-105)	124.47(108.34-143.93)	176(132-229)	123.59(91.9-160.51)	0.16(-0.57 to 0.89)
Belarus	596(477-739)	16.23(12.96-20.15)	625(444-851)	14.21(10.09-19.38)	-0.46(-1.23 to 0.32)	13026(10483-16001)	356.73(286.31-439.12)	9700(7042-13086)	224.13(162.4-303.13)	-1.6(-2.26 to -0.93)

location	Incidence					DALYs				
	Num in 1990	ASR in 1990	Num in 2021	ASR in 2021	AAPC	Num in 1990	ASR in 1990	Num in 2021	ASR in 2021	AAPC
Belgium	836(663-1041)	20.98(16.63-26.19)	596(459-755)	10.26(7.92-13.01)	-2.32(-2.66 to -1.98)	10063(8212-12271)	258.35(210.48-315.66)	5096(4062-6236)	89.21(71.33-109.11)	-3.38(-3.67 to -3.1)
Belize	1(1-1)	5.09(4.35-5.88)	5(4-6)	6.42(5.24-7.79)	0.72(-0.84 to 2.3)	28(24-32)	111.36(95.67-127.75)	109(89-132)	128.05(104.7-154.77)	0.4(-0.49 to 1.29)
Benin	19(12-27)	3.51(2.3-5.09)	53(33-79)	3.82(2.42-5.71)	0.28(0.16 to 0.4)	469(309-679)	86.31(56.73-124.84)	1291(811-1970)	88.59(55.95-134.48)	0.11(-0.11 to 0.32)
Bermuda	2(2-3)	11.88(9.38-14.83)	4(3-5)	10.33(7.52-13.92)	-0.69(-1.7 to 0.33)	36(28-44)	206.55(163.42-256.81)	42(31-56)	117.34(87.38-156)	-1.97(-3.06 to -0.87)
Bhutan	6(4-10)	8.99(5.36-14.24)	13(8-19)	7.69(4.72-11.81)	-0.49(-0.57 to -0.42)	163(96-259)	226.33(134.2-360.08)	268(165-415)	160.92(99.44-248.68)	-1.08(-1.18 to -0.98)
Bolivia (Plurinational State of)	48(31-69)	5.7(3.68-8.2)	101(64-152)	4.13(2.62-6.22)	-1.03(-1.14 to -0.91)	1183(768-1696)	134.55(87.44-193.03)	2141(1344-3296)	84.99(53.5-130.29)	-1.47(-1.54 to -1.41)
Bosnia and Herzegovina	212(171-258)	17.16(13.82-20.93)	253(177-336)	14.96(10.49-19.97)	-0.37(-0.87 to 0.13)	4820(3895-5876)	379.52(306.65-462.84)	4104(2865-5375)	246.06(171.25-323.03)	-1.3(-1.69 to -0.91)
Botswana	15(10-22)	9.5(6.06-13.77)	29(17-47)	6.84(4.24-11.15)	-1.05(-1.21 to -0.89)	393(245-577)	236.67(148.56-346.01)	695(415-1186)	158.08(95.95-265.02)	-1.26(-1.48 to -1.04)
Brazil	2883(2660-3115)	11.57(10.65-12.5)	7373(6661-8091)	10.5(9.48-11.52)	-0.29(-0.64 to 0.07)	68060(62870-73479)	264.24(243.81-285.25)	141855(129296-154689)	200.7(182.81-218.89)	-0.87(-1.09 to -0.65)

location	Incidence					DALYs				
	Num in 1990	ASR in 1990	Num in 2021	ASR in 2021	AAPC	Num in 1990	ASR in 1990	Num in 2021	ASR in 2021	AAPC
Brunei Darussalam	3(2-4)	10.4(7.19-14.79)	5(3-7)	5.06(3.48-7.17)	-2.33(-2.74 to -1.92)	43(30-60)	164.92(114.8-231.96)	59(41-83)	62.41(43.33-87.22)	-3.12(-3.39 to -2.85)
Bulgaria	507(412-618)	14.77(11.94-18.07)	696(537-881)	19.63(15.12-24.88)	1(0.06 to 1.94)	10189(8341-12285)	297.26(242.31-359.84)	10746(8561-13250)	307.3(244.74-379.09)	0.23(-0.74 to 1.2)
Burkina Faso	42(27-63)	3.54(2.28-5.28)	107(66-163)	4.32(2.69-6.57)	0.67(0.52 to 0.82)	1093(698-1627)	87.67(56.16-130.33)	2667(1632-4122)	102.3(63.02-157.17)	0.51(0.37 to 0.66)
Burundi	51(31-78)	8.02(4.79-12.19)	69(43-109)	5.1(3.22-7.92)	-1.44(-1.57 to -1.32)	1398(826-2146)	212.25(125.76-325.37)	1853(1141-2936)	127.69(79.65-200.51)	-1.63(-1.74 to -1.51)
Cabo Verde	4(2-6)	5.92(3.75-8.9)	8(5-12)	6.45(3.8-9.75)	0.24(-0.21 to 0.7)	79(51-118)	127.94(82.16-190.97)	154(90-235)	121.67(71.01-185.07)	-0.09(-1.03 to 0.85)
Cambodia	81(52-119)	6.65(4.34-9.72)	215(132-347)	6.31(3.91-10.13)	-0.14(-0.2 to -0.07)	2056(1318-3039)	160.55(103.58-236.33)	4585(2836-7413)	128.49(80.04-206.99)	-0.68(-0.83 to -0.53)
Cameroon	47(30-70)	3.8(2.49-5.64)	163(96-264)	4.76(2.83-7.61)	0.74(0.64 to 0.83)	1210(793-1815)	93.04(61.24-138.83)	4040(2308-6585)	110.64(63.99-178.76)	0.57(0.49 to 0.65)
Canada	1211(979-1493)	13.92(11.24-17.18)	1279(1004-1609)	6.62(5.19-8.34)	-2.3(-2.53 to -2.08)	12101(10144-14366)	140.01(117.26-166.31)	9640(7827-11744)	50.36(40.87-61.32)	-3.16(-3.28 to -3.04)
Central African Republic	25(13-40)	7.7(4.19-11.95)	39(21-63)	6.07(3.37-9.56)	-0.78(-0.87 to -0.7)	718(376-1146)	204.42(110.07-322.68)	1139(595-1855)	160.72(87.04-256.44)	-0.79(-0.9 to -0.67)

location	Incidence					DALYs				
	Num in 1990	ASR in 1990	Num in 2021	ASR in 2021	AAPC	Num in 1990	ASR in 1990	Num in 2021	ASR in 2021	AAPC
Chad	22(14-32)	2.9(1.87-4.29)	76(45-117)	4.89(2.96-7.53)	1.75(1.59 to 1.92)	558(362-824)	72.07(46.77-106.64)	1950(1158-3000)	118.56(70.87-182)	1.65(1.47 to 1.83)
Chile	189(154-232)	6.84(5.56-8.39)	283(223-355)	4(3.14-5.02)	-1.73(-2.31 to -1.15)	3732(3050-4541)	133.27(108.97-162.12)	3617(2912-4426)	51.36(41.32-62.87)	-3.07(-3.71 to -2.43)
China	14363(11514-17151)	6.34(5.09-7.54)	37684(28928-48332)	6.24(4.81-7.99)	-0.09(-0.23 to 0.06)	322216(257232-386030)	135.66(108.6-162.1)	469713(358088-602971)	77.13(58.92-98.76)	-1.86(-2.04 to -1.68)
Colombia	450(377-527)	9.77(8.19-11.46)	655(489-853)	4.35(3.24-5.65)	-2.52(-3.69 to -1.33)	9705(8179-11370)	203(171.01-237.85)	10239(7690-13265)	67.77(50.94-87.76)	-3.43(-4.47 to -2.39)
Comoros	4(2-5)	6.55(4.09-9.7)	7(4-10)	4.88(3.15-7.35)	-0.97(-1.16 to -0.78)	97(59-145)	166.31(101.3-245.92)	166(107-254)	116.02(74.72-176.15)	-1.17(-1.44 to -0.9)
Congo	23(13-34)	7.58(4.32-11.47)	45(29-68)	6.06(3.93-9.07)	-0.7(-0.88 to -0.51)	605(330-936)	192.92(107.27-296.3)	1145(726-1755)	140.75(90.76-213.29)	-0.99(-1.19 to -0.78)
Cook Islands	0(0-0)	2.43(1.62-3.42)	0(0-0)	1.96(1.28-3.02)	-0.68(-1.1 to -0.26)	1(1-2)	42.3(28.45-59.23)	2(1-3)	25.68(16.97-38.97)	-1.6(-1.99 to -1.21)
Costa Rica	37(30-45)	8.01(6.5-9.78)	58(45-75)	3.86(2.95-4.99)	-2.27(-2.61 to -1.92)	672(547-813)	143.4(116.77-173.58)	867(669-1105)	57.27(44.23-73.01)	-2.91(-3.24 to -2.58)
Coted'Ivoire	53(34-79)	4.75(3.1-6.98)	137(81-231)	4.34(2.61-7.16)	-0.3(-0.43 to -0.16)	1444(914-2169)	118.95(76.26-177.38)	3469(2017-5939)	102.54(60.71-172.53)	-0.48(-0.62 to -0.33)

location	Incidence					DALYs				
	Num in 1990	ASR in 1990	Num in 2021	ASR in 2021	AAPC	Num in 1990	ASR in 1990	Num in 2021	ASR in 2021	AAPC
Croatia	406(330-497)	22.8(18.51-27.97)	320(246-408)	14.29(10.97-18.25)	-1.44(-2.38 to -0.5)	7758(6365-9388)	433.05(354.83-525.04)	4272(3364-5367)	192.32(151-241.72)	-2.56(-3.69 to -1.41)
Cuba	564(464-681)	20.32(16.7-24.53)	1500(1142-1937)	27.75(21.13-35.85)	1.07(0.08 to 2.07)	10008(8307-12048)	360.47(299.25-433.84)	22261(17269-28655)	412.98(320.3-531.74)	0.47(0.16 to 0.78)
Cyprus	18(12-27)	8.79(5.86-12.89)	42(28-62)	7.6(5.06-11.12)	-0.51(-0.83 to -0.18)	267(181-386)	125.04(84.41-181.7)	362(250-521)	65.42(45.09-94.5)	-2.07(-2.34 to -1.8)
Czechia	459(376-556)	12.58(10.29-15.24)	470(368-593)	8.75(6.84-11.07)	-1.17(-1.99 to -0.33)	9237(7643-11070)	257.8(212.88-309.42)	6013(4795-7491)	113.88(90.68-141.92)	-2.65(-3.44 to -1.86)
Democratic People's Republic of Korea	198(122-294)	4.25(2.65-6.26)	409(252-612)	4.35(2.69-6.47)	0.08(0.02 to 0.14)	4395(2679-6529)	89.33(55.07-131.81)	7329(4503-11042)	76.41(47.3-114.68)	-0.51(-0.58 to -0.43)
Democratic Republic of the Congo	234(140-355)	5.5(3.34-8.33)	503(309-759)	4.89(3.04-7.35)	-0.39(-0.54 to -0.25)	6259(3728-9547)	136.41(82.23-207.19)	13151(8070-19801)	118.66(73.48-177.84)	-0.45(-0.57 to -0.34)
Denmark	295(241-360)	14.55(11.86-17.76)	274(215-343)	8.99(7.07-11.23)	-1.54(-1.95 to -1.14)	3647(3023-4327)	183.74(152.13-218.11)	2329(1898-2815)	77.64(63.43-93.78)	-2.69(-3.18 to -2.19)
Djibouti	3(2-5)	7.3(4.49-11.7)	12(7-19)	6.57(3.96-10.24)	-0.33(-0.47 to -0.2)	79(47-130)	184.44(112.26-298.8)	312(185-493)	155.27(93.43-242.32)	-0.59(-0.69 to -0.49)
Dominica	1(1-2)	8.84(6.43-11.84)	2(2-3)	9.94(6.68-14.22)	0.39(0.3 to 0.47)	30(22-40)	187.46(136.16-251.79)	50(33-71)	205.42(137.09-294.27)	0.32(0.24 to 0.41)

location	Incidence					DALYs				
	Num in 1990	ASR in 1990	Num in 2021	ASR in 2021	AAPC	Num in 1990	ASR in 1990	Num in 2021	ASR in 2021	AAPC
Dominican Republic	60(43-82)	6.19(4.45-8.48)	179(115-264)	6.55(4.21-9.66)	0.23(-0.12 to 0.6)	1404(1012-1911)	138.02(99.48-188.18)	3708(2395-5511)	134.25(86.79-199.38)	-0.06(-0.92 to 0.81)
Ecuador	53(44-63)	3.85(3.18-4.6)	105(75-143)	2.37(1.7-3.22)	-1.54(-1.94 to -1.14)	1169(970-1396)	82.54(68.42-98.53)	1885(1361-2577)	42.21(30.52-57.61)	-2.19(-2.6 to -1.78)
Egypt	317(233-442)	4.4(3.22-6.14)	930(643-1322)	5.4(3.76-7.65)	0.7(0.32 to 1.08)	7647(5660-10643)	97.5(71.98-135.7)	16783(11615-23764)	91.39(63.61-128.81)	-0.17(-0.53 to 0.19)
El Salvador	28(22-35)	3.58(2.8-4.49)	52(38-72)	3.13(2.25-4.29)	-0.25(-0.82 to 0.33)	637(501-799)	79.28(62.31-99.51)	924(668-1261)	55.77(40.29-76.16)	-0.96(-1.42 to -0.5)
Equatorial Guinea	4(2-6)	6.88(3.81-11.11)	7(4-11)	5.04(2.81-8.27)	-0.98(-1.2 to -0.77)	106(57-173)	180.25(98.24-291.93)	157(84-262)	107.55(58.85-178.22)	-1.64(-1.85 to -1.42)
Eritrea	26(16-40)	7.39(4.6-11.12)	46(29-68)	5.59(3.61-8.29)	-0.91(-1.02 to -0.8)	791(473-1203)	202.57(123.43-304.75)	1265(794-1912)	143.13(91.43-213.59)	-1.13(-1.26 to -1.01)
Estonia	85(67-105)	14.97(11.89-18.55)	76(56-100)	11.69(8.68-15.47)	-0.8(-2.13 to 0.54)	1419(1146-1727)	251.77(203.13-306.99)	734(558-935)	115.53(87.73-147.67)	-2.44(-3.47 to -1.4)
Eswatini	9(5-13)	10.75(6.37-16.29)	16(9-26)	10.05(5.59-15.53)	-0.21(-0.46 to 0.04)	232(134-360)	270.44(157.36-415.73)	430(234-687)	250.7(137.65-396.3)	-0.17(-0.45 to 0.1)
Ethiopia	313(172-468)	5.57(3.14-8.28)	394(272-561)	3.39(2.34-4.81)	-1.6(-1.73 to -1.47)	8764(4735-13190)	147.29(81.25-220.53)	9633(6618-13700)	78.9(54.27-111.94)	-2.01(-2.15 to -1.88)

location	Incidence					DALYs				
	**Num in 1990**	**ASR in 1990**	**Num in 2021**	**ASR in 2021**	**AAPC**	**Num in 1990**	**ASR in 1990**	**Num in 2021**	**ASR in 2021**	**AAPC**
Fiji	3(2-4)	2.8(1.91-4.07)	6(4-8)	2.72(1.74-4.08)	-0.14(-0.65 to 0.37)	57(40-82)	58.91(40.43-85.31)	115(75-170)	53.3(34.67-78.83)	-0.34(-0.74 to 0.06)
Finland	134(108-166)	7.19(5.77-8.93)	169(130-217)	5.56(4.29-7.15)	-0.65(-1.41 to 0.11)	1399(1147-1696)	76.08(62.27-92.42)	1140(914-1414)	38.17(30.6-47.35)	-2.19(-2.78 to -1.6)
France	6151(4922-7646)	30.19(24.1-37.61)	5540(4226-7107)	16.93(12.91-21.78)	-1.74(-2.15 to -1.34)	77908(64340-93723)	390.94(322.1-471.14)	40217(31845-49786)	123.92(98.28-153.42)	-3.66(-4.07 to -3.25)
Gabon	12(7-18)	7.43(4.66-11.31)	19(12-29)	6.46(4.09-9.6)	-0.4(-0.47 to -0.33)	296(186-451)	182.26(114.1-277.58)	449(281-675)	143.13(90.43-213.78)	-0.76(-0.98 to -0.54)
Gambia	2(1-3)	1.85(1.22-2.71)	5(3-7)	1.79(1.15-2.66)	-0.14(-0.68 to 0.4)	47(30-70)	46.13(30.14-68.73)	117(74-175)	42(26.8-62.51)	-0.3(-1.13 to 0.54)
Georgia	314(276-356)	17.33(15.2-19.64)	232(197-272)	14.36(12.18-16.82)	-0.09(-0.55 to 0.37)	7018(6234-7874)	385.48(342.18-432.73)	4621(3974-5328)	290.54(249.93-334.9)	-0.76(-1.4 to -0.13)
Germany	3411(2757-4178)	10.44(8.43-12.8)	3819(2979-4803)	7.95(6.21-9.99)	-0.93(-1.36 to -0.49)	51540(42543-61941)	160.25(132.11-192.78)	35796(28784-43751)	75.93(61.23-92.76)	-2.44(-2.77 to -2.11)
Ghana	50(33-76)	2.98(1.94-4.48)	195(118-293)	4.28(2.6-6.4)	1.19(1.04 to 1.34)	1297(842-1951)	72.07(46.97-108.14)	4644(2805-7104)	96.17(58.3-146.09)	0.96(0.81 to 1.11)
Greece	902(761-1062)	21.41(18.05-25.2)	906(745-1089)	15.75(12.98-18.97)	-0.97(-1.13 to -0.8)	9640(8521-10814)	230.84(203.98-259.09)	8281(7124-9465)	147.02(127.13-167.84)	-1.45(-1.64 to -1.26)

location	Incidence					DALYs				
	Num in 1990	ASR in 1990	Num in 2021	ASR in 2021	AAPC	Num in 1990	ASR in 1990	Num in 2021	ASR in 2021	AAPC
Greenland	1(1-2)	11.53(8.02-16.2)	1(1-2)	6.64(4.45-9.76)	-1.57(-1.92 to -1.23)	26(18-36)	240.07(167.27-333.53)	23(16-34)	107.37(72.93-156.41)	-2.45(-2.77 to -2.13)
Grenada	1(1-1)	6.12(4.9-7.62)	2(1-2)	5.14(3.93-6.53)	-0.41(-1.58 to 0.77)	25(20-31)	137.13(109.14-171.35)	35(27-44)	101.71(78.14-129.16)	-0.73(-2.08 to 0.64)
Guam	0(0-1)	2.62(1.92-3.42)	1(1-1)	1.25(0.94-1.65)	-2.01(-2.7 to -1.31)	8(6-11)	41.82(31.13-54.07)	12(9-15)	19.74(15.2-25.62)	-2.21(-2.88 to -1.54)
Guatemala	49(44-55)	5.77(5.13-6.45)	57(46-69)	1.94(1.58-2.36)	-3.63(-4.52 to -2.73)	1181(1056-1318)	127.85(114.31-142.59)	1171(947-1424)	39.11(31.68-47.49)	-3.96(-4.9 to -3.01)
Guinea	27(17-40)	3.01(1.93-4.42)	69(42-106)	4.57(2.83-7.01)	1.38(1.25 to 1.52)	690(445-999)	74.84(48.18-108.32)	1719(1052-2664)	109.33(67.24-168.61)	1.26(1.13 to 1.4)
Guinea-Bissau	5(3-9)	5.04(2.83-7.83)	11(7-17)	5.61(3.34-8.4)	0.36(0.27 to 0.45)	149(82-238)	130.51(72.47-206.78)	299(174-449)	139.74(81.97-208.8)	0.24(0.16 to 0.32)
Guyana	5(4-6)	4.51(3.54-5.74)	8(5-11)	4.24(2.91-5.9)	-0.13(-0.97 to 0.72)	116(91-149)	109.64(86-140.61)	190(131-266)	98.48(68.01-137.23)	-0.26(-1.11 to 0.59)
Haiti	89(52-135)	10.01(5.89-15.2)	160(93-252)	8.32(4.86-13.05)	-0.54(-0.58 to -0.49)	2404(1407-3687)	256.4(151.01-392.22)	4149(2387-6576)	200.73(116.32-316.92)	-0.74(-0.79 to -0.69)
Honduras	23(16-31)	4.24(2.98-5.86)	94(61-136)	5.63(3.67-8.1)	0.99(0.72 to 1.26)	543(384-752)	97.14(68.78-134.48)	2038(1337-2955)	117.16(76.69-169.34)	0.67(0.43 to 0.91)

location	Incidence					DALYs				
	Num in 1990	ASR in 1990	Num in 2021	ASR in 2021	AAPC	Num in 1990	ASR in 1990	Num in 2021	ASR in 2021	AAPC
Hungary	803(651-987)	20.41(16.53-25.14)	876(678-1113)	18.73(14.43-23.87)	-0.21(-0.74 to 0.32)	17125(14018-20955)	441.01(360.26-540.43)	12869(10070-16065)	277.99(216.98-347.45)	-1.5(-2.1 to -0.89)
Iceland	5(4-7)	7.18(5.72-9.01)	7(5-8)	4.4(3.39-5.63)	-1.51(-1.97 to -1.05)	55(45-66)	74.29(60.73-90.4)	49(39-60)	32.55(25.87-40.42)	-2.55(-2.86 to -2.24)
India	14361(11597-17495)	10.91(8.75-13.33)	32890(27757-38842)	9.86(8.32-11.63)	-0.32(-0.65 to 0.01)	382398(309933-465140)	274.78(221.57-335.12)	724819(610833-857391)	211.89(178.62-250.49)	-0.82(-1.09 to -0.56)
Indonesia	1097(796-1382)	4.13(2.97-5.23)	2950(2007-3987)	4.45(3-6)	0.24(0.17 to 0.31)	26640(19462-33485)	94.23(68.37-118.86)	60830(41565-82511)	86.48(58.73-116.82)	-0.28(-0.34 to -0.22)
Iran (Islamic Republic of)	807(664-948)	11.4(9.27-13.43)	2308(1928-2741)	10.99(9.15-13.04)	-0.19(-0.39 to 0.01)	17110(14128-19928)	227.01(185.91-265.06)	31735(27493-36255)	146.91(126.81-167.86)	-1.43(-1.55 to -1.31)
Iraq	224(148-324)	10.55(6.97-15.22)	742(467-1097)	11.34(7.2-16.68)	0.27(0.09 to 0.45)	4944(3307-7080)	226.66(152.01-323.73)	11903(7533-17425)	173.02(110.41-252.04)	-0.85(-1.01 to -0.68)
Ireland	139(112-170)	12.87(10.38-15.77)	157(122-200)	7.5(5.83-9.57)	-1.65(-2.49 to -0.8)	1750(1436-2095)	164.32(134.7-197.03)	1232(992-1520)	59.3(47.82-73.19)	-3.11(-3.77 to -2.43)
Israel	97(77-120)	7.58(6.06-9.43)	202(155-257)	6.28(4.85-8)	-0.52(-1.46 to 0.44)	1348(1101-1639)	107.35(87.5-130.71)	1921(1530-2374)	60.57(48.3-74.85)	-1.89(-2.54 to -1.23)
Italy	5028(4411-5753)	21.05(18.44-24.11)	3854(3272-4478)	10.74(9.16-12.5)	-2.15(-2.72 to -1.58)	64087(59495-68729)	271.72(252.13-291.55)	34186(30648-37529)	94.98(85.94-103.9)	-3.23(-3.42 to -3.05)

location	Incidence					DALYs				
	Num in 1990	ASR in 1990	Num in 2021	ASR in 2021	AAPC	Num in 1990	ASR in 1990	Num in 2021	ASR in 2021	AAPC
Jamaica	25(20-31)	5.18(4.1-6.43)	51(34-72)	6.06(4.1-8.58)	0.76(0.24 to 1.28)	482(384-595)	103.05(82.09-127.18)	927(625-1318)	110(74.2-156.34)	0.43(-0.12 to 0.97)
Japan	3166(2809-3538)	6.67(5.92-7.46)	3730(3184-4205)	3.83(3.33-4.24)	-1.9(-2.64 to -1.15)	22647(21389-23808)	47.89(45.15-50.38)	19457(17387-20998)	19.63(17.88-21.08)	-2.88(-3.24 to -2.52)
Jordan	25(16-36)	6.73(4.46-9.84)	91(57-141)	4.34(2.76-6.65)	-1.39(-1.84 to -0.94)	522(350-763)	132.87(89.17-192.97)	1270(805-1935)	57.23(36.57-86.68)	-2.66(-3.25 to -2.08)
Kazakhstan	485(430-544)	13.14(11.64-14.77)	303(255-355)	5.61(4.73-6.57)	-2.71(-3.29 to -2.14)	11930(10676-13335)	317.37(283.51-355.48)	6063(5172-6988)	110.86(94.6-127.77)	-3.36(-3.78 to -2.94)
Kenya	97(69-133)	4.31(3.09-5.9)	328(241-428)	5.03(3.7-6.53)	0.48(0.4 to 0.57)	2438(1744-3351)	104.02(74.39-142.71)	8104(5927-10683)	117.93(86.31-154.42)	0.41(0.26 to 0.56)
Kiribati	0(0-0)	0.73(0.48-1.06)	0(0-0)	0.77(0.48-1.2)	0.16(0.08 to 0.24)	2(1-3)	17.52(11.68-25.33)	4(2-6)	17.64(10.99-27.47)	0.03(-0.06 to 0.11)
Kuwait	16(12-20)	9.22(7.16-11.71)	25(18-34)	3.22(2.3-4.4)	-3.78(-7.26 to -0.18)	250(198-314)	138.16(109.67-172.72)	256(189-343)	31.05(22.82-41.21)	-4.6(-7.88 to -1.22)
Kyrgyzstan	67(53-83)	7.8(6.17-9.73)	47(35-61)	3.28(2.43-4.26)	-2.83(-4.19 to -1.44)	1688(1335-2098)	195.29(154.27-243.17)	1024(757-1318)	69.11(51.18-88.96)	-3.28(-4.46 to -2.07)
Lao People's Democratic Republic	36(23-56)	6.37(3.97-9.71)	56(34-89)	4.57(2.8-7.13)	-1.06(-1.13 to -0.99)	949(592-1463)	158.48(99.35-243.13)	1279(773-2027)	97.61(59.56-153.91)	-1.54(-1.59 to -1.49)

location	Incidence					DALYs				
	Num in 1990	ASR in 1990	Num in 2021	ASR in 2021	AAPC	Num in 1990	ASR in 1990	Num in 2021	ASR in 2021	AAPC
Latvia	142(115-173)	14.29(11.59-17.48)	96(73-123)	10.08(7.66-13)	-0.98(-1.8 to -0.15)	3044(2483-3699)	308(251-374.51)	1579(1215-2017)	170.51(130.69-218.56)	-1.88(-2.71 to -1.04)
Lebanon	83(51-125)	13.69(8.4-20.5)	209(141-299)	13(8.76-18.6)	-0.12(-0.44 to 0.2)	1638(1001-2456)	259.66(160.11-387.89)	2414(1662-3425)	151.62(104.34-215.1)	-1.63(-1.9 to -1.37)
Lesotho	18(12-26)	7.62(4.95-11.13)	37(22-55)	11.77(7.17-17.65)	1.48(1.27 to 1.68)	458(296-670)	190.57(123.59-278.56)	981(585-1488)	306.8(183.74-463.75)	1.59(1.42 to 1.77)
Liberia	10(6-16)	3.32(2.07-5.32)	22(13-36)	3.98(2.4-6.35)	0.59(0.34 to 0.83)	256(159-418)	81.39(50.56-132.71)	543(316-876)	90.3(53.42-144.55)	0.35(0.09 to 0.6)
Libya	74(47-112)	14.33(9.14-21.83)	248(156-383)	16.73(10.6-25.7)	0.59(0.04 to 1.13)	1512(963-2323)	283.3(181.52-434.03)	4169(2661-6415)	264.96(170.29-405.25)	-0.21(-0.46 to 0.03)
Lithuania	196(159-239)	15.67(12.72-19.1)	170(129-217)	12.02(9.11-15.35)	-0.77(-2.05 to 0.52)	4210(3448-5090)	337.11(275.79-407.92)	2863(2206-3613)	207.85(159.85-262.42)	-1.59(-2.85 to -0.32)
Luxembourg	27(23-32)	18.49(15.59-21.86)	29(23-35)	10.17(8.24-12.49)	-2.03(-2.87 to -1.19)	357(312-409)	249.59(217.54-285.5)	239(202-279)	85.49(72.1-99.75)	-3.32(-3.52 to -3.11)
Madagascar	81(53-116)	5.75(3.75-8.23)	126(80-189)	3.89(2.5-5.79)	-1.26(-1.33 to -1.18)	2159(1393-3095)	146.73(95.01-210.12)	3364(2146-5086)	95.39(61.41-142.9)	-1.4(-1.47 to -1.32)
Malawi	22(15-32)	2.07(1.41-2.98)	45(29-69)	2.15(1.43-3.28)	0.14(-0.03 to 0.3)	591(399-858)	52.88(35.8-76.55)	1155(753-1799)	52.78(34.74-81.45)	0.02(-0.14 to 0.18)

location	Incidence					DALYs				
	Num in 1990	ASR in 1990	Num in 2021	ASR in 2021	AAPC	Num in 1990	ASR in 1990	Num in 2021	ASR in 2021	AAPC
Malaysia	164(108-236)	6.71(4.41-9.68)	521(344-748)	6.66(4.4-9.56)	-0.16(-0.39 to 0.07)	3469(2292-5005)	136.76(90.34-197.51)	8407(5581-12047)	105.42(70.08-150.8)	-0.99(-1.22 to -0.76)
Maldives	1(1-2)	4.86(2.99-7.32)	2(1-3)	2.71(1.75-3.99)	-2.03(-2.49 to -1.56)	26(15-38)	102.54(61.72-154.39)	34(22-50)	37.43(24.36-55.08)	-3.3(-3.61 to -3)
Mali	43(30-59)	3.84(2.71-5.36)	88(56-133)	3.61(2.33-5.44)	-0.15(-0.33 to 0.03)	1135(797-1587)	96.79(68.09-135.28)	2226(1423-3419)	86.19(55.41-131.51)	-0.34(-0.5 to -0.17)
Malta	16(13-20)	13.7(10.72-17.31)	19(14-24)	8.01(6.08-10.4)	-1.68(-2.05 to -1.32)	210(168-257)	180.55(144.48-220.59)	166(130-208)	72.95(57.32-91.58)	-2.87(-3.06 to -2.68)
Marshall Islands	0(0-0)	2.81(1.7-4.36)	0(0-0)	2.81(1.63-4.46)	0.03(-0.16 to 0.23)	3(2-4)	62.27(37.9-96.06)	5(3-8)	57.9(33.62-90.61)	-0.19(-0.42 to 0.03)
Mauritania	9(6-13)	3.38(2.21-4.96)	23(14-36)	3.91(2.37-6.18)	0.5(0.32 to 0.68)	230(153-336)	83.52(55.39-122.12)	492(298-769)	81.79(49.68-127.47)	-0.05(-0.34 to 0.23)
Mauritius	19(16-23)	9.59(8.17-11.25)	42(36-50)	8.03(6.75-9.44)	-0.72(-2.65 to 1.26)	379(328-439)	184.17(159.23-213.35)	674(575-775)	127.54(108.8-146.81)	-1.3(-3.2 to 0.64)
Mexico	843(806-878)	7.82(7.45-8.14)	1210(1025-1413)	3.53(2.99-4.11)	-2.63(-3.5 to -1.75)	17933(17198-18655)	158.81(152.02-165.31)	21814(18488-25580)	62.35(52.9-73.02)	-3.08(-3.81 to -2.34)
Micronesia (Federated States of)	0(0-1)	2.9(1.83-4.49)	1(0-1)	2.56(1.59-3.94)	-0.41(-0.49 to -0.34)	8(5-13)	65.2(41.64-100.99)	11(7-17)	52.81(32.86-81.15)	-0.68(-0.75 to -0.61)

location	Incidence					DALYs				
	Num in 1990	ASR in 1990	Num in 2021	ASR in 2021	AAPC	Num in 1990	ASR in 1990	Num in 2021	ASR in 2021	AAPC
Monaco	8(5-12)	49.12(31.47-72.75)	8(5-12)	35.85(23.44-52.67)	-1.03(-1.15 to -0.9)	82(54-120)	513.79(332.64-751.04)	69(47-100)	303.13(204.82-443.8)	-1.71(-1.81 to -1.61)
Mongolia	15(10-21)	5.25(3.58-7.21)	22(15-32)	3.21(2.21-4.55)	-1.6(-2.1 to -1.09)	409(277-558)	137.17(93.21-187.45)	543(369-775)	73.62(50.26-105.05)	-1.98(-2.45 to -1.51)
Montenegro	52(40-67)	28.72(22.21-37.18)	82(61-110)	30.15(22.61-40.56)	0.37(-0.1 to 0.85)	907(704-1155)	500.01(388.32-637.23)	1172(887-1578)	434.43(328.06-586.1)	-0.28(-0.5 to -0.06)
Morocco	304(199-440)	7.87(5.17-11.39)	844(520-1291)	8.74(5.39-13.33)	0.34(0.23 to 0.45)	7027(4614-10074)	177.11(116.5-253.95)	15428(9600-23512)	155.56(96.95-236.42)	-0.41(-0.52 to -0.29)
Mozambique	111(70-164)	6.62(4.23-9.76)	211(136-313)	6.62(4.3-9.75)	0.02(-0.08 to 0.12)	3015(1915-4479)	169.76(108.35-251.39)	5693(3644-8474)	167.71(108.3-247.66)	-0.02(-0.11 to 0.07)
Myanmar	382(222-586)	6.04(3.57-9.19)	503(314-766)	3.76(2.36-5.7)	-1.53(-1.58 to -1.47)	9636(5524-14812)	145.55(84.61-222.46)	10634(6581-16103)	76.34(47.57-115.13)	-2.07(-2.14 to -2)
Namibia	17(12-24)	9.34(6.44-13.22)	39(25-59)	9.94(6.43-14.68)	0.22(0 to 0.43)	444(304-631)	232(159.35-329.03)	946(596-1409)	227.94(145.43-337.29)	0(-0.2 to 0.2)
Nauru	0(0-0)	3.93(2.35-5.95)	0(0-0)	2.93(1.74-4.55)	-0.96(-1.04 to -0.87)	1(1-1)	83.2(48.84-125.83)	1(1-1)	59.85(34.97-92.06)	-1.06(-1.16 to -0.96)
Nepal	276(167-428)	10.65(6.49-16.41)	560(348-849)	8.71(5.42-13.21)	-0.62(-0.73 to -0.51)	7350(4446-11432)	267.01(162.32-413.35)	12557(7873-19265)	189.58(119.06-290.41)	-1.08(-1.19 to -0.98)

location	Incidence					DALYs				
	Num in 1990	ASR in 1990	Num in 2021	ASR in 2021	AAPC	Num in 1990	ASR in 1990	Num in 2021	ASR in 2021	AAPC
Netherlands	680(552-840)	13.01(10.56-16.07)	712(558-894)	7.58(5.95-9.51)	-1.67(-1.83 to -1.51)	6567(5473-7856)	127.31(106.03-152.38)	4996(4055-6061)	54.05(43.91-65.58)	-2.7(-3.02 to -2.39)
New Zealand	103(81-130)	9.66(7.59-12.19)	124(96-158)	5.38(4.14-6.85)	-1.77(-2.16 to -1.37)	854(706-1026)	80.89(66.86-97.24)	721(587-879)	31.58(25.76-38.52)	-3.05(-3.4 to -2.7)
Nicaragua	18(13-25)	4.67(3.35-6.36)	44(30-65)	3.38(2.31-4.94)	-1.13(-1.35 to -0.9)	407(292-554)	99.11(71.02-134.68)	833(569-1214)	62.17(42.48-90.35)	-1.63(-1.85 to -1.4)
Niger	24(15-36)	3.15(1.95-4.75)	75(43-119)	3.46(2.03-5.44)	0.33(0.12 to 0.54)	638(395-965)	79.25(49.39-119.3)	1886(1085-3005)	80.87(47-128.34)	0.09(-0.16 to 0.35)
Nigeria	615(414-852)	5(3.41-6.89)	1026(683-1433)	4.15(2.83-5.73)	-0.62(-0.71 to -0.52)	16343(10898-22935)	127.87(86.03-178.44)	25250(16550-35424)	94.45(63.38-131.2)	-0.99(-1.09 to -0.88)
Niue	0(0-0)	2.15(1.37-3.24)	0(0-0)	2.22(1.42-3.37)	0.09(0.04 to 0.14)	0(0-0)	42.74(27.26-64.04)	0(0-0)	38.24(24.7-57.21)	-0.35(-0.42 to -0.27)
North Macedonia	94(73-122)	17.53(13.52-22.67)	166(118-226)	17.55(12.53-23.84)	0.01(-0.29 to 0.31)	2141(1671-2766)	390.22(304.3-505.08)	2831(2052-3869)	298.15(216.39-406.93)	-0.81(-1.05 to -0.56)
Northern Mariana Islands	0(0-0)	5.15(3.23-7.85)	0(0-1)	3.23(2.14-4.67)	-1.46(-1.87 to -1.05)	4(2-5)	85.31(53.61-129.31)	7(5-10)	48.93(32.61-70.07)	-1.75(-2.19 to -1.3)
Norway	111(96-128)	6.44(5.56-7.42)	128(107-151)	4.76(3.97-5.62)	-0.69(-1.1 to -0.28)	1043(956-1135)	61.13(56.11-66.43)	803(710-890)	29.9(26.55-33.08)	-2.14(-3.47 to -0.8)

location	Incidence					DALYs				
	**Num in 1990**	**ASR in 1990**	**Num in 2021**	**ASR in 2021**	**AAPC**	**Num in 1990**	**ASR in 1990**	**Num in 2021**	**ASR in 2021**	**AAPC**
Oman	7(4-11)	3.77(2.35-5.74)	17(11-26)	3.07(1.92-4.72)	-0.67(-1.24 to -0.09)	144(88-222)	75.26(46.59-114.79)	228(146-348)	38.5(24.7-57.98)	-2.12(-2.48 to -1.77)
Pakistan	2788(2104-3675)	18.19(13.67-24.03)	5808(4077-8148)	17.28(12.15-24.1)	-0.16(-0.26 to -0.07)	70602(53464-92316)	445.07(336.04-583.12)	143896(100935-201725)	402.24(282.56-561.21)	-0.32(-0.4 to -0.24)
Palau	0(0-0)	2.35(1.49-3.52)	0(0-0)	1.95(1.2-3.01)	-0.66(-0.78 to -0.54)	1(1-2)	45.25(28.6-67.73)	2(1-3)	34.02(21.16-52.85)	-0.93(-1.05 to -0.81)
Palestine	14(9-20)	5.98(3.89-8.96)	34(23-50)	5.03(3.41-7.25)	-0.56(-0.84 to -0.28)	271(176-406)	115.37(74.98-172.25)	535(364-776)	74.64(50.94-107.56)	-1.4(-1.62 to -1.19)
Panama	26(23-30)	6.68(5.76-7.69)	43(32-54)	3.55(2.62-4.51)	-1.96(-2.32 to -1.61)	511(444-581)	128.21(111.4-145.89)	685(505-864)	56.86(41.9-71.69)	-2.65(-3.48 to -1.81)
Papua New Guinea	9(5-14)	1.99(1.17-3.2)	22(13-35)	1.83(1.11-2.97)	-0.22(-0.43 to -0.02)	205(118-328)	42.9(24.94-68.92)	502(304-819)	38.08(23.05-61.8)	-0.32(-0.41 to -0.23)
Paraguay	33(23-45)	5.4(3.74-7.52)	124(79-187)	7.71(4.93-11.56)	1.16(0.69 to 1.64)	734(508-1036)	119.42(82.81-168.26)	2452(1543-3710)	149.57(94.32-226.03)	0.7(0.19 to 1.22)
Peru	134(96-180)	4.31(3.08-5.79)	246(159-366)	2.72(1.76-4.04)	-1.37(-2.39 to -0.35)	2965(2144-3984)	92.53(66.92-124.34)	3971(2551-5930)	43.69(28.1-65.13)	-2.32(-3.33 to -1.29)
Philippines	297(241-378)	3.79(3.08-4.8)	833(661-1023)	3.65(2.91-4.47)	-0.1(-0.2 to -0.01)	6717(5471-8460)	80.57(65.78-101.29)	17306(13771-21269)	72.87(58.21-89.21)	-0.31(-0.45 to -0.17)

location	Incidence					DALYs				
	Num in 1990	ASR in 1990	Num in 2021	ASR in 2021	AAPC	Num in 1990	ASR in 1990	Num in 2021	ASR in 2021	AAPC
Poland	2103(1971-2244)	17.68(16.55-18.88)	2620(2285-2965)	13.89(12.11-15.74)	-0.86(-1.29 to -0.43)	49434(46970-52021)	419.66(398.55-441.9)	43674(38949-48380)	235.56(210.05-261)	-1.97(-2.47 to -1.47)
Portugal	536(440-651)	14.24(11.66-17.3)	504(397-630)	8.6(6.8-10.77)	-1.66(-1.83 to -1.48)	11967(9809-14573)	322.72(263.86-393.43)	8333(6596-10347)	147.84(117.11-183.5)	-2.54(-2.72 to -2.36)
Puerto Rico	109(87-134)	11.07(8.85-13.56)	100(74-133)	5.64(4.11-7.52)	-2.52(-3.17 to -1.87)	1915(1538-2329)	194.65(156.3-236.76)	1297(965-1710)	75.08(55.67-99.46)	-3.45(-4.11 to -2.79)
Qatar	3(2-5)	13.23(8.67-20.02)	26(15-42)	10.63(6.42-16.95)	-0.67(-1.34 to 0)	64(41-99)	223.96(146.81-339.74)	281(168-449)	103.24(63.63-162.33)	-2.46(-3.24 to -1.68)
Republic of Korea	1181(762-1616)	14.3(9.24-19.5)	2024(1370-2852)	7.64(5.17-10.78)	-1.97(-2.46 to -1.49)	16845(11100-22642)	199(131.18-266.7)	10868(7588-14885)	41.21(28.74-56.5)	-4.98(-5.25 to -4.71)
Republic of Moldova	190(167-216)	14.9(13.07-16.94)	221(183-264)	13.41(11.13-16)	-0.29(-1.46 to 0.89)	4489(3970-5058)	349.09(308.55-393.62)	4084(3454-4798)	251.09(212.3-294.94)	-1.04(-2.28 to 0.22)
Romania	1113(902-1352)	13.89(11.24-16.92)	1848(1418-2355)	20.48(15.69-26.1)	1.34(0.9 to 1.78)	25603(20758-30993)	319.83(258.61-388.09)	28048(22119-35000)	311.95(245.89-389.18)	-0.04(-0.82 to 0.74)
Russian Federation	7893(7582-8194)	15.3(14.68-15.9)	6616(5828-7316)	10(8.81-11.06)	-1.22(-2.01 to -0.42)	177656(170554-184503)	345.82(331.7-359.52)	106607(94143-118045)	164.28(145.15-181.84)	-2.27(-3.2 to -1.33)
Rwanda	74(47-111)	9.32(5.94-13.86)	98(60-154)	5.52(3.44-8.67)	-1.66(-1.78 to -1.54)	2062(1312-3076)	246.74(157.3-367.87)	2450(1506-3933)	130.9(81.07-208.67)	-2.01(-2.14 to -1.88)

location	Incidence					DALYs				
	Num in 1990	ASR in 1990	Num in 2021	ASR in 2021	AAPC	Num in 1990	ASR in 1990	Num in 2021	ASR in 2021	AAPC
Saint Kitts and Nevis	1(1-1)	6.13(5.21-7.19)	1(1-2)	7.2(5.6-9.11)	0.68(-0.42 to 1.79)	14(12-16)	143.11(122.01-166.77)	30(23-38)	139.33(108.73-176.52)	0.04(-1.03 to 1.13)
Saint Lucia	2(2-3)	9.64(8.32-11.07)	6(5-8)	9.34(7.14-11.79)	-0.01(-0.75 to 0.73)	49(42-56)	209.22(181.35-239.55)	120(93-152)	176.86(136.82-222.66)	-0.41(-1.18 to 0.36)
Saint Vincent and the Grenadines	2(2-2)	9.44(8.06-10.97)	5(4-6)	11.57(9.55-13.94)	0.6(-0.21 to 1.41)	39(34-46)	205.59(175.61-238.76)	95(79-114)	235.45(195.22-282.43)	0.38(-0.28 to 1.05)
Samoa	0(0-0)	1.15(0.79-1.67)	0(0-1)	1.09(0.73-1.64)	-0.16(-0.29 to -0.02)	6(4-9)	25.13(17.24-36.17)	9(6-14)	21.65(14.59-32.26)	-0.47(-0.61 to -0.32)
San Marino	2(1-3)	20.58(13.48-30.29)	2(1-3)	9.27(4.8-15.51)	-2.72(-2.93 to -2.51)	18(12-26)	202.14(135.2-289.4)	14(7-22)	75.79(40.28-124.84)	-3.33(-3.49 to -3.17)
Sao Tome and Principe	0(0-1)	2.5(1.59-3.68)	1(1-1)	3.17(2.02-4.75)	0.75(0.46 to 1.05)	10(7-15)	57.35(36.7-84.67)	20(13-30)	65.03(41.74-96.92)	0.4(0.15 to 0.64)
Saudi Arabia	58(37-86)	3.75(2.41-5.52)	218(142-326)	3.9(2.57-5.76)	0.12(-0.02 to 0.27)	1306(833-1932)	79.75(51.24-117.31)	3250(2122-4823)	52.85(35.12-77.27)	-1.34(-1.47 to -1.21)
Senegal	33(21-47)	3.73(2.45-5.39)	92(56-141)	4.4(2.67-6.72)	0.57(0.23 to 0.92)	836(548-1223)	91.67(60.17-133.81)	2202(1329-3350)	100.16(60.49-152.23)	0.33(-0.04 to 0.71)
Serbia	642(442-919)	19.3(13.29-27.66)	823(559-1156)	19.48(13.2-27.37)	0.08(-0.53 to 0.69)	13099(9059-18800)	386.67(267.21-555.92)	11226(7596-15671)	267.43(180.35-374.12)	-1.09(-1.43 to -0.75)

location	Incidence					DALYs				
	Num in 1990	ASR in 1990	Num in 2021	ASR in 2021	AAPC	Num in 1990	ASR in 1990	Num in 2021	ASR in 2021	AAPC
Seychelles	3(2-5)	22.57(15.29-31.78)	7(4-10)	20.39(13.62-29.06)	-0.39(-0.72 to -0.06)	71(48-99)	464.41(315.48-653.47)	116(77-165)	338.95(227.66-482.8)	-1.06(-1.35 to -0.76)
Sierra Leone	20(13-29)	3.6(2.32-5.37)	41(26-65)	4.1(2.55-6.35)	0.43(0.35 to 0.51)	496(318-750)	88.86(57.03-134.36)	1027(638-1613)	96.59(60.38-150.88)	0.28(0.18 to 0.38)
Singapore	73(58-92)	12.18(9.64-15.3)	139(105-180)	5.82(4.4-7.51)	-2.2(-3.09 to -1.29)	815(663-994)	134.2(109.22-163.42)	770(607-961)	32.42(25.52-40.48)	-4.48(-5.23 to -3.72)
Slovakia	305(215-423)	19.04(13.34-26.36)	315(211-459)	12.47(8.31-18.2)	-1.35(-1.42 to -1.28)	6358(4483-8754)	401.47(282.59-554.52)	5003(3356-7190)	200.95(134.16-289.33)	-2.2(-2.53 to -1.87)
Slovenia	102(83-125)	14.95(12.08-18.27)	122(92-157)	10.99(8.31-14.2)	-1.23(-2.12 to -0.34)	1805(1482-2175)	265.17(217.49-320.01)	1244(976-1561)	113.93(89.16-143.11)	-2.92(-3.65 to -2.17)
Solomon Islands	1(1-2)	2.92(1.7-4.63)	2(1-3)	2.53(1.56-3.91)	-0.47(-0.61 to -0.34)	24(13-39)	65.24(36.74-104.66)	51(32-79)	54.52(33.73-83.6)	-0.59(-0.73 to -0.44)
Somalia	52(30-83)	7.61(4.42-12.02)	98(56-155)	5.7(3.33-8.87)	-0.91(-1.01 to -0.82)	1521(862-2450)	202.94(117.14-323.16)	2803(1596-4491)	150.86(87.62-238.93)	-0.92(-1.04 to -0.81)
South Africa	427(330-605)	7.44(5.74-10.54)	908(779-1049)	6.85(5.88-7.91)	-0.29(-1.02 to 0.45)	10527(8159-14799)	178.01(137.94-250.97)	20341(17423-23665)	149.58(128.18-173.78)	-0.65(-1.41 to 0.12)
South Sudan	51(31-80)	7.26(4.43-11.22)	63(37-101)	5.73(3.36-9.02)	-0.74(-0.93 to -0.56)	1339(810-2062)	185.18(112.11-285.01)	1682(972-2706)	140.35(81.93-223.55)	-0.87(-1.05 to -0.69)

location	Incidence					DALYs				
	Num in 1990	ASR in 1990	Num in 2021	ASR in 2021	AAPC	Num in 1990	ASR in 1990	Num in 2021	ASR in 2021	AAPC
Spain	3934(3159-4812)	27.35(21.91-33.51)	3573(2709-4629)	14.99(11.36-19.44)	-1.97(-2.56 to -1.37)	56536(46241-67812)	399.71(326.26-480.07)	32648(25485-40742)	137.22(107.29-171.01)	-3.37(-3.56 to -3.18)
Sri Lanka	110(77-158)	3.88(2.7-5.55)	428(232-701)	5.58(3.05-9.12)	1.2(0.74 to 1.66)	2289(1602-3258)	76.71(53.69-109.16)	6094(3324-9970)	79.2(43.41-129.44)	0.11(-0.35 to 0.58)
Sudan	220(125-380)	8.89(5.03-15.52)	414(244-646)	8.01(4.76-12.44)	-0.34(-0.4 to -0.28)	5338(3048-8913)	207(117.9-348.66)	8385(4914-13313)	152.77(90.41-240.8)	-0.98(-1.03 to -0.92)
Suriname	2(2-3)	3.43(2.47-4.65)	6(4-9)	3.26(2.03-4.93)	-0.08(-0.76 to 0.62)	57(41-78)	78.64(56.59-106.63)	131(81-197)	71.06(43.85-106.61)	-0.24(-0.89 to 0.41)
Sweden	186(152-228)	4.76(3.88-5.84)	200(154-253)	3.52(2.71-4.47)	-0.85(-1.98 to 0.29)	1592(1344-1873)	41.17(34.81-48.41)	1254(998-1538)	22.13(17.61-27.15)	-1.91(-2.93 to -0.87)
Switzerland	324(257-403)	12.32(9.76-15.36)	295(224-380)	6.44(4.9-8.32)	-2.05(-2.4 to -1.7)	3429(2828-4126)	132.36(109.15-159.31)	2149(1705-2655)	47.27(37.6-58.32)	-3.39(-3.99 to -2.8)
Syrian Arab Republic	85(60-119)	6.12(4.32-8.58)	243(161-349)	6.6(4.42-9.37)	0.2(-0.14 to 0.55)	1852(1316-2576)	125.85(89.59-175.53)	3624(2452-5196)	93.77(64.13-133.08)	-1.02(-1.34 to -0.69)
Taiwan (Province of China)	331(268-408)	7.34(5.92-9.04)	692(523-899)	5.92(4.47-7.69)	-0.66(-1.2 to -0.12)	4769(3932-5744)	104.37(85.98-125.87)	6137(4836-7704)	53.13(41.84-66.68)	-2.04(-2.36 to -1.73)
Tajikistan	46(33-61)	5.9(4.31-7.88)	56(37-81)	3.3(2.13-4.76)	-1.91(-2.3 to -1.52)	1175(851-1566)	148.15(107.59-197.28)	1389(912-2021)	76.94(50.07-111.42)	-2.15(-2.54 to -1.77)

location	Incidence					DALYs				
	Num in 1990	ASR in 1990	Num in 2021	ASR in 2021	AAPC	Num in 1990	ASR in 1990	Num in 2021	ASR in 2021	AAPC
Thailand	869(612-1221)	9(6.35-12.61)	2375(1512-3582)	7.72(4.92-11.65)	-0.49(-0.78 to -0.2)	17674(12461-24769)	174.17(123.06-243.43)	32747(21147-48968)	106.55(68.79-159.49)	-1.6(-2.01 to -1.19)
Timor-Leste	3(2-4)	3.88(2.43-5.99)	9(5-13)	3.75(2.33-5.76)	-0.11(-0.24 to 0.02)	72(45-113)	90.82(56.92-140.53)	188(116-291)	79.06(48.65-122.19)	-0.43(-0.66 to -0.2)
Togo	12(8-19)	3.77(2.44-5.6)	52(31-80)	4.81(2.94-7.34)	0.83(0.75 to 0.91)	324(208-485)	92.01(59.36-137.51)	1318(781-2036)	113.59(67.89-174.01)	0.69(0.52 to 0.86)
Tokelau	0(0-0)	2.38(1.46-3.75)	0(0-0)	1.98(1.23-3.13)	-0.58(-0.64 to -0.52)	0(0-0)	49.06(29.84-77.68)	0(0-0)	34.78(21.5-55.04)	-1.11(-1.17 to -1.05)
Tonga	0(0-1)	2.26(1.4-3.58)	0(0-1)	2.17(1.32-3.4)	-0.15(-0.5 to 0.19)	7(4-10)	43.67(27.57-68.69)	8(5-13)	38.5(23.65-59.87)	-0.4(-0.7 to -0.09)
Trinidad and Tobago	13(11-15)	5.85(5.07-6.74)	29(21-38)	5.3(3.85-7.07)	-0.38(-0.54 to -0.23)	290(253-333)	126.87(110.49-145.59)	555(403-736)	102.28(74.37-135.69)	-0.62(-1.53 to 0.3)
Tunisia	169(112-247)	12.32(8.12-17.93)	493(293-779)	13.2(7.86-20.81)	0.23(0.11 to 0.35)	3292(2151-4787)	231.77(151.71-336.59)	6596(3960-10286)	174.63(105.04-271.72)	-0.92(-1.03 to -0.81)
Turkey	1437(915-2194)	15.14(9.68-23.09)	3057(2009-4480)	11.68(7.69-17.09)	-0.88(-1.18 to -0.58)	31799(20210-48600)	320.68(204.89-489.03)	39534(26163-57367)	149.52(99.05-216.74)	-2.43(-2.65 to -2.21)
Turkmenistan	51(46-57)	9.38(8.31-10.5)	51(38-67)	4.25(3.19-5.56)	-3.03(-3.42 to -2.64)	1316(1171-1460)	233.3(207.21-259.33)	1173(890-1555)	94.71(72.13-125.06)	-3.37(-3.76 to -2.98)

location	Incidence					DALYs				
	Num in 1990	ASR in 1990	Num in 2021	ASR in 2021	AAPC	Num in 1990	ASR in 1990	Num in 2021	ASR in 2021	AAPC
Tuvalu	0(0-0)	2.44(1.6-3.56)	0(0-0)	2.26(1.46-3.35)	-0.25(-0.3 to -0.19)	1(1-2)	55.31(36.42-80.93)	1(1-2)	44.86(29.42-66.39)	-0.67(-0.74 to -0.61)
Uganda	145(96-207)	8.13(5.44-11.59)	271(170-421)	6.6(4.2-10.13)	-0.69(-0.84 to -0.55)	3779(2485-5433)	203.29(134.36-290.86)	6835(4187-10713)	156.61(97.28-242.68)	-0.86(-1 to -0.71)
Ukraine	3279(2652-4033)	16.45(13.28-20.29)	1991(1244-2970)	9.79(6.09-14.64)	-1.71(-2.05 to -1.37)	70924(57828-86789)	357.62(291.02-438.9)	36756(23135-54548)	183.4(114.96-272.5)	-2.14(-2.44 to -1.83)
United Arab Emirates	8(5-13)	7.38(4.19-11.7)	56(35-85)	5.56(3.63-8.14)	-1.01(-1.73 to -0.29)	192(105-313)	150.68(85.17-240.94)	969(619-1445)	80.35(53.22-116.57)	-2.02(-2.69 to -1.35)
United Kingdom	2573(2449-2690)	10.95(10.42-11.45)	3011(2787-3199)	8.94(8.32-9.48)	-0.73(-1.04 to -0.42)	24650(23708-25586)	106.72(102.69-110.81)	20623(19312-21773)	62(58.34-65.36)	-1.81(-2.25 to -1.36)
United Republic of Tanzania	210(133-317)	6.87(4.4-10.33)	336(206-557)	4.71(2.91-7.68)	-1.22(-1.34 to -1.11)	5470(3446-8343)	172.28(109.18-261.58)	8417(5081-14289)	111.75(68.14-187.14)	-1.4(-1.5 to -1.29)
United States of America	11898(11310-12398)	14.44(13.75-15.04)	16139(15042-17030)	10.23(9.56-10.78)	-1.15(-1.35 to -0.96)	104943(100232-109018)	129.49(123.87-134.43)	111867(104886-117991)	71.96(67.63-75.82)	-1.97(-2.1 to -1.84)
United States Virgin Islands	2(1-3)	7.44(5.13-10.65)	2(2-4)	4.99(3.07-7.57)	-1.35(-1.95 to -0.74)	37(25-54)	149.78(102.67-217.63)	42(26-64)	88.36(54.42-133.87)	-1.7(-2.31 to -1.08)
Uruguay	257(206-317)	24.29(19.51-30.04)	208(162-262)	14.78(11.46-18.61)	-1.7(-1.84 to -1.57)	4824(3904-5913)	462.94(374.06-568.02)	3066(2444-3791)	222.16(177.03-274.93)	-2.35(-2.58 to -2.13)

location	Incidence					DALYs				
	Num in 1990	ASR in 1990	Num in 2021	ASR in 2021	AAPC	Num in 1990	ASR in 1990	Num in 2021	ASR in 2021	AAPC
Uzbekistan	268(219-323)	8.23(6.73-9.9)	200(150-262)	2.63(1.99-3.43)	-3.63(-4.52 to -2.73)	6690(5475-8059)	201.9(165.53-242.69)	4512(3393-5899)	56.99(43.04-74.06)	-4.02(-4.89 to -3.15)
Vanuatu	0(0-1)	2.14(1.26-3.41)	1(0-1)	1.76(1.09-2.72)	-0.64(-0.84 to -0.44)	7(4-12)	45.78(26.86-73.09)	17(11-26)	36.79(22.94-56.61)	-0.7(-0.89 to -0.52)
Venezuela (Bolivarian Republic of)	275(244-309)	10.86(9.59-12.17)	729(530-987)	8.72(6.36-11.77)	-0.86(-1.37 to -0.34)	5997(5326-6692)	229.08(203.14-255.67)	13566(9854-18279)	159.7(116.28-214.74)	-1.33(-1.77 to -0.89)
Viet Nam	596(391-866)	5.4(3.55-7.85)	2279(1403-3517)	7.91(4.92-12.13)	1.25(1.13 to 1.36)	13028(8602-19126)	116.62(77.1-171.22)	36558(22526-56621)	124.06(77.12-190.87)	0.21(0.11 to 0.31)
Yemen	137(78-218)	10.13(5.8-16.12)	367(206-583)	9.87(5.57-15.63)	-0.08(-0.21 to 0.04)	3474(1967-5543)	242.94(138.28-387.44)	8044(4436-12714)	203.38(113.26-320.05)	-0.55(-0.73 to -0.36)
Zambia	59(38-87)	7.53(4.88-11.01)	156(71-378)	7.8(3.69-18.17)	0.14(-0.02 to 0.31)	1596(1026-2325)	191.19(123.7-277.99)	4095(1811-10057)	191.46(87.99-455.86)	0.02(-0.14 to 0.19)
Zimbabwe	91(61-130)	7.98(5.4-11.38)	167(108-249)	8.34(5.48-12.27)	0.2(0.04 to 0.36)	2252(1514-3222)	190.51(128.4-271.78)	4514(2930-6724)	211.93(139.19-312.4)	0.39(0.1 to 0.69)

S7 Table: Incidence and DALYs of Nasopharynx cancer in middle-aged and older adults, and their average annual percentage changes in 204 countries/territories from 1990 to 2021.

**location**	**Incidence**					**DALYs**				
	**Num in 1990**	**ASR in 1990**	**Num in 2021**	**ASR in 2021**	**AAPC**	**Num in 1990**	**ASR in 1990**	**Num in 2021**	**ASR in 2021**	**AAPC**
Afghanistan	49(23-79)	2.4(1.13-3.85)	51(24-84)	1.69(0.8-2.7)	-1.12(-1.17 to -1.07)	1508(680-2469)	70.67(32.29-115.18)	1663(740-2749)	48.34(22.03-78.06)	-1.21(-1.27 to -1.16)
Albania	7(5-10)	1.27(0.88-1.81)	14(9-21)	1.17(0.73-1.77)	-0.38(-0.68 to -0.08)	214(147-304)	35.26(24.28-50.16)	350(218-530)	30.5(18.89-46.17)	-0.58(-0.9 to -0.27)
Algeria	238(160-339)	7.15(4.78-10.21)	576(370-850)	5.63(3.61-8.34)	-0.77(-0.89 to -0.65)	7034(4728-10011)	199.32(133.81-284.2)	15801(10068-23392)	147.1(93.78-218.15)	-0.99(-1.11 to -0.87)
American Samoa	0(0-0)	4.57(2.94-6.91)	1(0-1)	4.81(3.18-6.97)	0.17(-0.3 to 0.64)	9(6-14)	131.67(84.63-200.78)	21(13-30)	134.4(88.59-195.61)	0.06(-0.42 to 0.55)
Andorra	0(0-0)	1.05(0.57-1.79)	0(0-1)	0.68(0.34-1.19)	-1.5(-1.88 to -1.13)	4(2-6)	23.68(13.18-40.13)	5(3-9)	12.07(6.23-20.95)	-2.31(-2.61 to -2)
Angola	16(10-24)	1.34(0.83-2.06)	42(26-66)	1.19(0.73-1.86)	-0.37(-0.59 to -0.15)	503(308-771)	39.66(24.28-60.79)	1325(806-2118)	34.15(20.89-54.4)	-0.46(-0.7 to -0.22)
Antigua and Barbuda	0(0-0)	0.98(0.84-1.14)	0(0-0)	1.2(0.99-1.41)	0.73(-0.08 to 1.55)	3(3-4)	25.87(22.09-30.02)	9(8-11)	29.63(24.53-35.21)	0.53(-0.29 to 1.36)
Argentina	103(82-128)	1.18(0.93-1.47)	89(66-119)	0.6(0.44-0.8)	-2.1(-2.48 to -1.72)	2866(2274-3564)	32.48(25.76-40.4)	2222(1630-2965)	15.17(11.11-20.28)	-2.42(-2.75 to -2.1)

location	Incidence					DALYs				
	Num in 1990	ASR in 1990	Num in 2021	ASR in 2021	AAPC	Num in 1990	ASR in 1990	Num in 2021	ASR in 2021	AAPC
Armenia	4(3-5)	0.54(0.45-0.65)	10(8-12)	0.83(0.68-1.01)	1.44(1.12 to 1.75)	123(103-149)	15.23(12.67-18.39)	259(209-314)	22.33(18-27.07)	1.29(0.96 to 1.62)
Australia	166(131-205)	3.23(2.55-3.99)	210(155-277)	1.94(1.44-2.54)	-1.67(-2.28 to -1.05)	2439(1992-2923)	47.83(39.07-57.34)	2418(1792-3155)	22.48(16.7-29.3)	-2.49(-2.77 to -2.21)
Austria	37(29-46)	1.27(1.01-1.58)	36(26-48)	0.82(0.59-1.11)	-1.45(-1.64 to -1.26)	891(708-1104)	31.25(24.89-38.64)	664(485-880)	15.36(11.24-20.36)	-2.29(-2.47 to -2.11)
Azerbaijan	7(4-11)	0.49(0.3-0.77)	12(7-20)	0.41(0.25-0.66)	-0.58(-0.81 to -0.35)	211(132-326)	14.17(8.77-21.89)	364(215-581)	11.33(6.68-18.06)	-0.72(-0.96 to -0.49)
Bahamas	1(1-1)	1.53(1.29-1.8)	2(2-3)	1.77(1.35-2.3)	0.5(0.21 to 0.79)	19(16-22)	43.07(36.01-50.86)	58(44-77)	47.27(35.57-61.98)	0.31(0 to 0.62)
Bahrain	1(1-2)	2.74(1.82-3.97)	4(2-7)	1.42(0.84-2.34)	-2.04(-2.61 to -1.47)	38(25-55)	71.69(47.35-103.48)	106(59-180)	33.03(18.91-55.18)	-2.48(-2.86 to -2.09)
Bangladesh	647(407-977)	4.84(3.05-7.32)	1065(599-1778)	2.72(1.54-4.53)	-1.8(-1.96 to -1.64)	20140(12645-30198)	145.74(91.62-218.85)	30946(17280-51789)	77.21(43.25-128.99)	-2(-2.22 to -1.78)
Barbados	1(1-1)	1.44(1.22-1.69)	3(2-3)	1.87(1.37-2.45)	1.04(0.37 to 1.71)	26(22-31)	37.58(31.85-44.23)	64(46-85)	46.23(33.46-61.52)	0.91(0.24 to 1.58)
Belarus	40(29-54)	1.11(0.8-1.5)	40(28-58)	0.93(0.64-1.34)	-0.49(-1.06 to 0.08)	1214(880-1645)	34.13(24.79-46.14)	1114(763-1607)	26.24(17.99-37.83)	-0.78(-1.36 to -0.21)

location	Incidence					DALYs				
	Num in 1990	ASR in 1990	Num in 2021	ASR in 2021	AAPC	Num in 1990	ASR in 1990	Num in 2021	ASR in 2021	AAPC
Belgium	62(48-80)	1.63(1.25-2.1)	69(50-94)	1.29(0.93-1.76)	-0.88(-1.2 to -0.55)	1444(1120-1842)	38.61(29.98-49.17)	1288(948-1713)	24.23(17.89-32.16)	-1.64(-1.97 to -1.3)
Belize	0(0-0)	0.64(0.55-0.74)	1(1-1)	0.98(0.8-1.2)	1.44(1 to 1.88)	4(4-5)	17.51(14.99-20.31)	24(19-29)	26.47(21.51-32.41)	1.37(0.95 to 1.8)
Benin	2(1-3)	0.33(0.22-0.5)	5(3-9)	0.32(0.18-0.53)	-0.15(-0.4 to 0.1)	61(39-91)	10.62(6.82-15.88)	179(97-302)	10.28(5.65-17.24)	-0.11(-0.37 to 0.15)
Bermuda	0(0-0)	1.84(1.39-2.4)	1(0-1)	1.72(1.17-2.48)	-0.31(-0.58 to -0.05)	8(6-11)	47.25(35.47-61.59)	13(9-18)	37.08(25.53-53.22)	-0.77(-0.95 to -0.58)
Bhutan	3(2-5)	4.18(2.43-6.67)	4(3-7)	2.6(1.51-4.43)	-1.5(-1.61 to -1.4)	98(57-158)	125.92(73.4-202.43)	126(72-218)	73.53(41.87-126.37)	-1.71(-1.82 to -1.6)
Bolivia (Plurinational State of)	6(4-10)	0.75(0.48-1.11)	15(9-23)	0.62(0.39-0.94)	-0.63(-0.7 to -0.56)	188(120-279)	20.6(13.19-30.56)	413(257-636)	16.01(10-24.56)	-0.82(-0.89 to -0.75)
Bosnia and Herzegovina	5(4-5)	0.37(0.3-0.44)	8(6-11)	0.5(0.37-0.66)	1.14(0.82 to 1.46)	138(113-166)	10.73(8.81-12.91)	216(160-285)	13.59(9.98-18.01)	0.79(0.44 to 1.14)
Botswana	3(2-5)	2.19(1.35-3.43)	7(4-12)	1.76(1.01-2.85)	-0.73(-1.15 to -0.3)	104(64-165)	62.17(38.18-98.47)	222(120-371)	49.31(27.38-81.59)	-0.73(-1.07 to -0.39)
Brazil	145(131-160)	0.56(0.5-0.62)	448(399-498)	0.63(0.56-0.7)	0.41(0.1 to 0.73)	4521(4097-4992)	16.84(15.25-18.61)	12876(11528-14301)	18.08(16.18-20.09)	0.27(-0.08 to 0.62)

location	Incidence					DALYs				
	Num in 1990	ASR in 1990	Num in 2021	ASR in 2021	AAPC	Num in 1990	ASR in 1990	Num in 2021	ASR in 2021	AAPC
Brunei Darussalam	3(2-4)	10.45(6.87-15.08)	8(6-12)	7.98(5.42-11.34)	-0.86(-1.02 to -0.7)	86(56-123)	287.92(189.58-414.7)	246(168-348)	216.46(147.58-305.77)	-0.93(-1.06 to -0.8)
Bulgaria	26(20-34)	0.8(0.6-1.03)	49(35-67)	1.47(1.05-2.02)	2.09(1.46 to 2.72)	714(542-919)	21.46(16.21-27.75)	1170(855-1595)	35.85(26.13-48.97)	1.76(1.15 to 2.38)
Burkina Faso	5(3-7)	0.38(0.23-0.58)	11(7-18)	0.39(0.23-0.62)	0.12(-0.01 to 0.25)	159(97-246)	11.93(7.33-18.48)	376(213-602)	12.56(7.15-20)	0.19(0.06 to 0.31)
Burundi	38(24-58)	5.8(3.62-8.8)	60(34-98)	4.07(2.36-6.62)	-1.16(-1.29 to -1.03)	1196(735-1819)	176.7(108.81-268.37)	1891(1082-3121)	121.05(69.87-198.28)	-1.24(-1.37 to -1.11)
Cabo Verde	0(0-0)	0.06(0.04-0.09)	1(0-1)	0.47(0.28-0.75)	7(6.67 to 7.32)	1(1-2)	1.97(1.27-3.01)	21(12-33)	14.91(8.78-23.45)	6.84(6.5 to 7.17)
Cambodia	83(54-121)	6.43(4.18-9.41)	217(135-332)	6.09(3.81-9.26)	-0.16(-0.24 to -0.07)	2512(1616-3674)	185.58(119.64-271.07)	6265(3912-9665)	167.58(104.99-257.2)	-0.31(-0.43 to -0.19)
Cameroon	5(3-8)	0.38(0.25-0.57)	17(10-27)	0.4(0.24-0.64)	0.1(0.02 to 0.18)	178(114-267)	12.24(7.88-18.3)	571(327-931)	13.01(7.53-21.08)	0.17(0.09 to 0.24)
Canada	159(129-194)	1.9(1.54-2.32)	185(137-245)	1.08(0.79-1.42)	-1.85(-2.14 to -1.56)	3377(2792-4023)	40.56(33.57-48.23)	3146(2371-4068)	18.18(13.77-23.45)	-2.54(-2.77 to -2.31)
Central African Republic	6(4-8)	1.65(1.04-2.45)	10(6-15)	1.38(0.85-2.16)	-0.53(-0.59 to -0.47)	181(114-273)	48.93(31-73.45)	314(187-493)	40.94(24.77-63.93)	-0.55(-0.61 to -0.48)

location	Incidence					DALYs				
	Num in 1990	ASR in 1990	Num in 2021	ASR in 2021	AAPC	Num in 1990	ASR in 1990	Num in 2021	ASR in 2021	AAPC
Chad	2(1-4)	0.29(0.18-0.45)	7(4-12)	0.39(0.23-0.62)	1(0.77 to 1.23)	75(47-118)	9.23(5.79-14.5)	255(145-414)	12.84(7.35-20.68)	1.1(0.86 to 1.35)
Chile	16(12-20)	0.58(0.45-0.75)	19(14-26)	0.27(0.2-0.37)	-2.36(-2.6 to -2.11)	442(343-564)	15.67(12.16-20.03)	434(324-575)	6.23(4.66-8.25)	-2.92(-3.49 to -2.36)
China	30210(24913-35781)	12.69(10.49-15)	46272(35836-59117)	7.57(5.86-9.65)	-1.67(-1.94 to -1.4)	848528(696761-1006079)	342.63(281.88-405.63)	778626(609000-985288)	126.19(98.74-159.58)	-3.19(-3.5 to -2.88)
Colombia	43(35-53)	0.89(0.72-1.09)	81(57-112)	0.53(0.37-0.74)	-1.71(-1.95 to -1.46)	1223(987-1492)	24.27(19.57-29.63)	2004(1405-2780)	13.17(9.23-18.27)	-2.09(-2.34 to -1.84)
Comoros	2(1-4)	4.15(2.42-6.76)	5(3-9)	3.54(1.98-5.97)	-0.52(-0.7 to -0.34)	78(44-128)	125.3(71.56-204.93)	159(86-274)	104.16(56.92-177.98)	-0.6(-0.81 to -0.39)
Congo	5(3-7)	1.61(1.06-2.36)	11(6-16)	1.28(0.8-1.94)	-0.73(-0.96 to -0.5)	154(100-228)	47.46(30.93-69.77)	333(201-516)	36.77(22.53-56.4)	-0.81(-1.04 to -0.59)
Cook Islands	0(0-0)	0.72(0.46-1.08)	0(0-0)	0.62(0.39-0.94)	-0.51(-0.85 to -0.17)	1(0-1)	19.83(12.68-29.84)	1(1-2)	15.14(9.56-23.08)	-0.86(-1.16 to -0.55)
Costa Rica	7(6-9)	1.58(1.24-1.97)	17(12-23)	1.14(0.83-1.53)	-1.11(-1.42 to -0.79)	201(158-250)	41.94(32.92-52.28)	423(307-570)	27.87(20.28-37.47)	-1.41(-1.73 to -1.09)
Coted'Ivoire	13(8-19)	0.98(0.61-1.49)	33(18-55)	0.91(0.52-1.5)	-0.23(-0.39 to -0.07)	409(252-631)	29.97(18.57-46)	1065(584-1800)	27.81(15.52-46.48)	-0.25(-0.46 to -0.03)

location	Incidence					DALYs				
	Num in 1990	ASR in 1990	Num in 2021	ASR in 2021	AAPC	Num in 1990	ASR in 1990	Num in 2021	ASR in 2021	AAPC
Croatia	21(16-26)	1.19(0.92-1.51)	20(15-26)	0.92(0.69-1.2)	-0.96(-2.07 to 0.17)	621(477-786)	34.73(26.64-44.01)	503(381-660)	24.3(18.33-31.95)	-1.27(-2.42 to -0.1)
Cuba	35(27-43)	1.26(0.97-1.58)	101(72-138)	1.86(1.34-2.53)	1.47(1.3 to 1.65)	874(679-1091)	31.72(24.68-39.59)	2474(1782-3377)	45.93(33.14-62.68)	1.43(1.24 to 1.62)
Cyprus	2(1-3)	0.89(0.59-1.31)	3(2-5)	0.66(0.42-0.99)	-0.97(-1.24 to -0.7)	46(30-67)	21.46(14.14-31.33)	64(42-96)	12.15(7.98-18.23)	-1.85(-2.16 to -1.55)
Czechia	55(43-70)	1.55(1.22-1.95)	48(33-68)	0.94(0.64-1.32)	-1.52(-1.64 to -1.4)	1507(1188-1901)	42.97(33.93-54.14)	998(701-1373)	19.9(13.97-27.38)	-2.46(-2.71 to -2.2)
Democratic People's Republic of Korea	294(183-450)	5.83(3.66-8.87)	469(289-712)	4.83(2.99-7.32)	-0.61(-0.65 to -0.57)	8918(5466-13695)	168.12(103.74-256.9)	13018(7908-19784)	131.54(80.37-199.45)	-0.79(-0.82 to -0.76)
Democratic Republic of the Congo	55(34-85)	1.19(0.74-1.88)	123(73-199)	1.12(0.67-1.83)	-0.21(-0.29 to -0.13)	1690(1047-2652)	34.52(21.31-54.32)	3831(2246-6286)	32.2(19.04-52.98)	-0.26(-0.37 to -0.14)
Denmark	18(14-23)	0.92(0.72-1.16)	20(14-28)	0.74(0.51-1.03)	-0.64(-1.1 to -0.18)	380(302-469)	20.09(16.01-24.79)	304(217-414)	11.03(7.9-14.94)	-1.87(-2.24 to -1.51)
Djibouti	2(1-3)	4.22(2.32-7.29)	8(4-15)	3.92(2.05-7.04)	-0.24(-0.33 to -0.16)	60(32-107)	126.88(68.22-223.38)	256(128-479)	115.22(58.97-212.38)	-0.31(-0.41 to -0.22)
Dominica	0(0-0)	1.26(0.92-1.7)	0(0-1)	1.59(1.04-2.34)	0.73(0.51 to 0.95)	5(4-7)	33.17(24.11-44.78)	10(7-15)	42.01(27.43-62.32)	0.76(0.53 to 0.99)

location	Incidence					DALYs				
	Num in 1990	ASR in 1990	Num in 2021	ASR in 2021	AAPC	Num in 1990	ASR in 1990	Num in 2021	ASR in 2021	AAPC
Dominican Republic	12(9-17)	1.3(0.91-1.81)	33(21-51)	1.21(0.76-1.86)	-0.08(-0.55 to 0.4)	350(245-488)	34.07(23.9-47.56)	895(561-1369)	31.98(20.04-48.85)	-0.12(-0.7 to 0.46)
Ecuador	6(5-7)	0.42(0.33-0.53)	15(10-22)	0.35(0.24-0.5)	-0.66(-1.12 to -0.2)	159(124-199)	10.86(8.5-13.62)	383(256-549)	8.55(5.73-12.25)	-0.86(-1.33 to -0.4)
Egypt	24(17-32)	0.32(0.23-0.44)	40(26-58)	0.22(0.15-0.32)	-1.19(-1.48 to -0.91)	703(501-965)	8.59(6.11-11.82)	1131(748-1654)	5.75(3.83-8.38)	-1.3(-1.57 to -1.03)
El Salvador	5(4-6)	0.6(0.49-0.74)	10(8-14)	0.61(0.45-0.81)	0.08(-0.6 to 0.77)	137(112-166)	16.6(13.56-20.18)	265(194-352)	16.07(11.75-21.35)	-0.07(-0.79 to 0.65)
Equatorial Guinea	1(1-1)	1.45(0.91-2.23)	1(1-2)	1(0.57-1.63)	-1.13(-1.35 to -0.91)	26(17-41)	42.89(26.97-66.2)	45(25-75)	27.98(15.66-46.55)	-1.31(-1.54 to -1.09)
Eritrea	21(14-30)	5.25(3.52-7.69)	40(25-63)	4.51(2.81-7.02)	-0.51(-0.65 to -0.37)	693(458-1026)	163.18(108.48-239.85)	1310(803-2065)	136.07(84.25-212.96)	-0.61(-0.76 to -0.46)
Estonia	11(8-15)	2.02(1.48-2.73)	5(4-7)	0.84(0.57-1.16)	-2.88(-3.91 to -1.84)	336(246-453)	60.73(44.4-81.9)	136(94-189)	22.52(15.46-31.25)	-3.18(-4.02 to -2.33)
Eswatini	2(1-3)	2.26(1.44-3.33)	4(2-6)	2.47(1.48-3.85)	0.3(0.11 to 0.48)	56(35-83)	64.44(40.85-95.45)	123(72-195)	71.45(42.02-112.63)	0.31(0.08 to 0.53)
Ethiopia	347(223-472)	5.76(3.72-7.84)	465(295-730)	3.71(2.37-5.8)	-1.41(-1.47 to -1.35)	11120(7096-15065)	175.03(112.23-237.25)	14254(8969-22706)	107.91(68.16-171.02)	-1.56(-1.62 to -1.5)

location	Incidence					DALYs				
	Num in 1990	ASR in 1990	Num in 2021	ASR in 2021	AAPC	Num in 1990	ASR in 1990	Num in 2021	ASR in 2021	AAPC
Fiji	1(1-2)	1.21(0.82-1.77)	2(1-4)	1(0.65-1.52)	-0.62(-0.85 to -0.39)	41(28-61)	35.26(23.86-51.61)	69(44-105)	28.42(18.21-43.2)	-0.7(-0.93 to -0.47)
Finland	11(9-14)	0.63(0.49-0.79)	10(7-14)	0.38(0.26-0.52)	-1.62(-1.79 to -1.45)	275(216-345)	15.46(12.19-19.37)	186(133-250)	6.93(4.99-9.33)	-2.57(-2.76 to -2.37)
France	770(607-965)	3.97(3.11-4.99)	617(430-855)	2.02(1.4-2.82)	-2.19(-2.28 to -2.09)	17903(14209-22043)	93.92(74.31-115.81)	9134(6546-12384)	30.18(21.61-40.99)	-3.63(-3.86 to -3.4)
Gabon	2(2-4)	1.53(1.01-2.25)	4(2-6)	1.22(0.75-1.86)	-0.73(-0.88 to -0.57)	73(48-107)	44.39(29.33-65.44)	114(70-177)	34.33(21.26-52.94)	-0.81(-0.94 to -0.68)
Gambia	0(0-1)	0.45(0.29-0.66)	1(1-2)	0.49(0.31-0.73)	0.29(-0.69 to 1.27)	14(9-21)	13.45(8.83-20.02)	44(27-66)	14.71(9.23-22.07)	0.26(-0.77 to 1.3)
Georgia	16(11-22)	0.91(0.63-1.27)	21(15-28)	1.33(0.93-1.8)	1.25(0.18 to 2.33)	471(329-659)	26.37(18.42-36.92)	571(397-772)	37.63(26.21-50.97)	1.26(0.5 to 2.03)
Germany	459(373-564)	1.44(1.16-1.77)	383(276-515)	0.86(0.62-1.16)	-1.74(-2.19 to -1.29)	10756(8832-12920)	34.25(28.06-41.22)	6796(5006-8912)	15.57(11.49-20.41)	-2.73(-3.07 to -2.39)
Ghana	4(2-6)	0.18(0.1-0.3)	1(1-2)	0.03(0.01-0.04)	-5.98(-6.62 to -5.34)	137(75-227)	6.3(3.44-10.42)	52(29-88)	0.87(0.48-1.46)	-6.08(-6.73 to -5.42)
Greece	71(60-84)	1.77(1.49-2.08)	93(76-112)	1.75(1.43-2.13)	-0.04(-0.22 to 0.13)	1575(1358-1824)	39.17(33.78-45.4)	1782(1500-2096)	34.82(29.45-40.93)	-0.37(-0.54 to -0.21)

location	Incidence					DALYs				
	Num in 1990	ASR in 1990	Num in 2021	ASR in 2021	AAPC	Num in 1990	ASR in 1990	Num in 2021	ASR in 2021	AAPC
Greenland	3(2-4)	30.98(22.93-41.18)	3(2-4)	14.22(9.56-20.57)	-2.39(-2.71 to -2.08)	104(76-138)	894.58(660.94-1186.89)	83(56-119)	374.89(251.9-543.58)	-2.69(-3.01 to -2.38)
Grenada	0(0-0)	1.61(1.21-2.12)	1(0-1)	1.86(1.37-2.5)	0.53(0.23 to 0.83)	8(6-10)	44.14(33.03-58.08)	16(12-22)	48.18(35.5-65.08)	0.4(-0.08 to 0.89)
Guam	2(1-2)	8.14(6.37-10.23)	3(2-4)	4.9(3.73-6.34)	-1.7(-2.57 to -0.82)	54(42-67)	221.46(174.56-277.34)	78(60-100)	134.45(102.71-172.41)	-1.69(-2.05 to -1.33)
Guatemala	8(7-9)	0.83(0.72-0.95)	18(14-22)	0.59(0.47-0.73)	-1.26(-1.84 to -0.69)	218(188-251)	21.92(18.98-25.24)	483(382-595)	15.71(12.43-19.34)	-1.26(-1.89 to -0.63)
Guinea	7(5-11)	0.79(0.52-1.16)	14(8-21)	0.83(0.51-1.29)	0.19(0.11 to 0.27)	225(148-332)	23.85(15.71-35.2)	429(258-673)	25.17(15.19-39.36)	0.18(0.06 to 0.29)
Guinea-Bissau	1(0-1)	0.49(0.29-0.78)	1(1-2)	0.5(0.31-0.76)	0.1(0 to 0.19)	20(12-32)	15.79(9.37-25.49)	42(26-65)	16.3(10-25)	0.14(0.08 to 0.19)
Guyana	1(1-1)	0.7(0.53-0.91)	2(1-3)	0.92(0.59-1.38)	0.93(0.28 to 1.59)	21(16-28)	19.59(14.65-25.76)	51(33-77)	25.93(16.69-39.25)	0.96(0.43 to 1.5)
Haiti	19(11-29)	2.17(1.23-3.26)	42(23-66)	2.11(1.18-3.32)	-0.03(-0.1 to 0.04)	583(325-881)	61.06(34.27-92.09)	1261(697-1976)	58.14(32.37-91.05)	-0.1(-0.17 to -0.03)
Honduras	3(2-5)	0.63(0.42-0.89)	13(8-19)	0.74(0.47-1.11)	0.57(0.29 to 0.84)	100(68-143)	17.21(11.62-24.5)	353(224-530)	19.52(12.37-29.32)	0.44(0.16 to 0.72)

location	Incidence					DALYs				
	Num in 1990	ASR in 1990	Num in 2021	ASR in 2021	AAPC	Num in 1990	ASR in 1990	Num in 2021	ASR in 2021	AAPC
Hungary	57(45-71)	1.48(1.17-1.84)	76(56-101)	1.65(1.23-2.18)	0.37(-0.18 to 0.92)	1697(1356-2099)	44.8(35.81-55.4)	2026(1522-2666)	45.65(34.35-59.99)	0.05(-0.53 to 0.64)
Iceland	1(1-1)	1.22(0.95-1.56)	1(1-2)	0.88(0.62-1.22)	-1.09(-1.45 to -0.73)	19(15-23)	26.42(20.82-33.35)	20(15-27)	14.26(10.48-18.87)	-1.99(-2.32 to -1.66)
India	5546(4571-6582)	3.92(3.22-4.66)	9732(8320-11326)	2.83(2.42-3.29)	-1.03(-1.2 to -0.86)	176690(145935-209446)	119.08(98.04-141.33)	288321(245760-336241)	81.79(69.77-95.3)	-1.19(-1.35 to -1.03)
Indonesia	1236(939-1600)	4.31(3.28-5.56)	2786(1966-3844)	3.93(2.82-5.35)	-0.29(-0.34 to -0.24)	37649(28465-48849)	123.38(93.58-159.65)	80264(55900-111880)	105.85(74.66-145.87)	-0.49(-0.54 to -0.44)
Iran (Islamic Republic of)	44(38-51)	0.63(0.54-0.73)	91(79-106)	0.43(0.37-0.5)	-1.2(-1.39 to -1.02)	1264(1079-1455)	16.53(14.09-19.11)	2257(1985-2594)	10.17(8.91-11.7)	-1.54(-1.73 to -1.35)
Iraq	33(22-49)	1.47(0.98-2.17)	74(45-112)	0.98(0.61-1.47)	-1.3(-1.56 to -1.03)	1006(668-1491)	43.74(29.04-64.66)	2113(1306-3187)	26.55(16.48-39.77)	-1.62(-1.86 to -1.39)
Ireland	15(12-19)	1.42(1.09-1.82)	17(12-23)	0.84(0.6-1.15)	-1.72(-2.1 to -1.33)	350(272-447)	34.04(26.39-43.51)	287(212-383)	14.41(10.65-19.26)	-2.76(-3.02 to -2.5)
Israel	16(12-21)	1.33(1.01-1.71)	28(20-38)	0.91(0.66-1.23)	-1.3(-1.69 to -0.91)	402(307-518)	33.11(25.3-42.63)	553(411-734)	18.32(13.62-24.33)	-1.99(-2.38 to -1.61)
Italy	390(348-432)	1.72(1.53-1.91)	350(292-416)	1.08(0.9-1.28)	-1.57(-1.81 to -1.32)	9661(8847-10500)	43.24(39.6-47.01)	6913(6064-7837)	21.66(19.13-24.48)	-2.29(-2.54 to -2.04)

location	Incidence					DALYs				
	Num in 1990	ASR in 1990	Num in 2021	ASR in 2021	AAPC	Num in 1990	ASR in 1990	Num in 2021	ASR in 2021	AAPC
Jamaica	4(3-6)	0.94(0.69-1.25)	13(8-19)	1.51(0.97-2.25)	2.01(1.14 to 2.9)	111(82-148)	24.24(17.95-32.14)	330(212-492)	38.73(24.86-57.79)	1.99(1.07 to 2.92)
Japan	416(389-444)	0.89(0.82-0.94)	904(790-999)	1(0.89-1.11)	0.34(-0.01 to 0.69)	10305(9774-10836)	21.75(20.61-22.88)	17421(15748-18746)	21.02(19.39-22.44)	-0.18(-0.51 to 0.15)
Jordan	11(7-16)	2.68(1.78-3.9)	33(20-51)	1.43(0.88-2.22)	-1.99(-2.23 to -1.74)	328(218-480)	75.66(50.46-110.58)	881(542-1384)	35.78(22.15-56.16)	-2.35(-2.59 to -2.1)
Kazakhstan	39(33-48)	1.1(0.92-1.35)	61(48-76)	1.17(0.93-1.46)	0.2(-0.1 to 0.49)	1176(985-1432)	31.87(26.68-38.99)	1761(1395-2218)	32.59(25.84-41)	0.08(-0.25 to 0.4)
Kenya	117(81-169)	4.93(3.41-7.12)	383(278-514)	5.49(3.99-7.34)	0.34(0.25 to 0.43)	3597(2488-5240)	145.54(100.62-211.63)	11932(8585-16104)	161.7(116.8-217.36)	0.33(0.24 to 0.42)
Kiribati	0(0-0)	2.69(1.61-4.22)	1(0-1)	2.5(1.44-4.04)	-0.23(-0.28 to -0.18)	9(6-15)	82.5(49.59-129.83)	18(10-30)	76.28(43.82-124.52)	-0.25(-0.29 to -0.2)
Kuwait	4(3-6)	2.27(1.68-2.98)	7(4-10)	0.7(0.48-1)	-3.19(-4.97 to -1.37)	122(90-162)	59.54(44.05-78.57)	155(104-221)	15.25(10.35-21.66)	-3.79(-5.59 to -1.96)
Kyrgyzstan	9(6-12)	1.08(0.77-1.48)	17(12-25)	1.23(0.82-1.74)	0.43(0.1 to 0.76)	281(199-387)	33.2(23.53-45.65)	521(352-735)	34.61(23.39-48.81)	0.16(-0.21 to 0.53)
Lao People's Democratic Republic	41(24-62)	6.83(4.1-10.31)	63(39-95)	4.7(2.94-7.07)	-1.2(-1.24 to -1.16)	1253(754-1926)	200.11(120.73-306.63)	1864(1158-2855)	130.83(81.87-199.23)	-1.37(-1.41 to -1.33)

location	Incidence					DALYs				
	Num in 1990	ASR in 1990	Num in 2021	ASR in 2021	AAPC	Num in 1990	ASR in 1990	Num in 2021	ASR in 2021	AAPC
Latvia	21(16-27)	2.13(1.6-2.78)	8(6-12)	0.91(0.63-1.27)	-2.77(-3.92 to -1.6)	630(474-820)	64.67(48.58-84.37)	231(162-321)	26.02(18.3-36.14)	-2.98(-4.2 to -1.75)
Lebanon	10(6-16)	1.56(0.94-2.46)	16(10-24)	1.02(0.66-1.51)	-1.32(-1.51 to -1.13)	288(170-463)	43.84(25.98-70.22)	385(248-568)	24.64(15.89-36.39)	-1.82(-2.01 to -1.63)
Lesotho	4(3-7)	1.79(1.12-2.82)	9(5-13)	2.83(1.74-4.36)	1.54(1.27 to 1.82)	121(75-194)	50.26(31.18-80.24)	260(158-402)	81.75(49.97-125.94)	1.63(1.35 to 1.91)
Liberia	1(1-2)	0.35(0.21-0.54)	3(1-4)	0.35(0.2-0.59)	0(-0.39 to 0.39)	35(22-56)	10.98(6.68-17.25)	90(49-156)	11.31(6.25-19.39)	0.04(-0.35 to 0.43)
Libya	43(27-65)	7.84(4.93-11.86)	127(80-189)	7.54(4.8-11.21)	-0.13(-0.42 to 0.17)	1283(790-1943)	225.49(139.68-341.4)	3672(2311-5449)	205.89(130.18-304.87)	-0.29(-0.6 to 0.03)
Lithuania	24(19-30)	1.94(1.52-2.44)	12(9-15)	0.84(0.62-1.1)	-2.7(-3.66 to -1.73)	730(567-917)	59.16(45.89-74.33)	315(233-413)	23.95(17.67-31.34)	-2.91(-3.96 to -1.85)
Luxembourg	3(3-4)	2.36(1.98-2.81)	4(3-5)	1.35(1.07-1.71)	-1.89(-2.33 to -1.46)	82(69-96)	59(49.84-69.22)	69(57-84)	24.94(20.59-30.64)	-2.84(-3.22 to -2.45)
Madagascar	58(36-87)	3.9(2.42-5.87)	110(63-180)	3.03(1.76-4.95)	-0.79(-0.87 to -0.71)	1803(1120-2707)	117.37(72.96-176.48)	3530(1980-5806)	90.41(51.35-147.88)	-0.83(-0.91 to -0.76)
Malawi	11(7-16)	0.97(0.63-1.44)	20(13-31)	0.91(0.58-1.38)	-0.15(-0.29 to -0.02)	346(222-517)	29.31(18.89-43.64)	646(401-1004)	27.49(17.26-42.37)	-0.16(-0.33 to 0.02)

location	Incidence					DALYs				
	Num in 1990	ASR in 1990	Num in 2021	ASR in 2021	AAPC	Num in 1990	ASR in 1990	Num in 2021	ASR in 2021	AAPC
Malaysia	539(368-770)	19.95(13.61-28.54)	1288(860-1872)	15.75(10.52-22.87)	-0.72(-0.99 to -0.46)	16203(11023-23250)	578.98(394.09-830.63)	34687(23083-49997)	415.76(276.88-599.07)	-1.03(-1.29 to -0.76)
Maldives	0(0-1)	1.53(0.91-2.29)	1(0-1)	0.69(0.45-1.01)	-2.62(-2.9 to -2.34)	12(7-18)	42.19(24.56-63.13)	16(10-24)	15.89(10.38-23.7)	-3.17(-3.45 to -2.9)
Mali	6(4-8)	0.49(0.33-0.7)	12(7-18)	0.43(0.26-0.68)	-0.39(-0.47 to -0.3)	187(127-270)	14.87(10.09-21.51)	367(219-596)	13.04(7.83-21.03)	-0.42(-0.5 to -0.34)
Malta	5(4-7)	4.64(3.53-6.05)	7(5-9)	3.26(2.27-4.47)	-1.14(-1.47 to -0.81)	130(100-169)	113.26(86.76-146.8)	130(92-175)	62.33(44.02-83.87)	-1.94(-2.27 to -1.6)
Marshall Islands	0(0-0)	3.93(2.42-6.02)	0(0-1)	3.42(2.01-5.32)	-0.45(-0.5 to -0.4)	5(3-8)	117.16(71.76-179.28)	12(7-18)	100.24(59.2-155.19)	-0.51(-0.56 to -0.46)
Mauritania	1(1-2)	0.34(0.21-0.53)	2(1-4)	0.29(0.14-0.53)	-0.55(-0.85 to -0.24)	31(19-50)	10.57(6.51-16.82)	60(28-117)	8.89(4.24-17.3)	-0.57(-0.88 to -0.27)
Mauritius	5(4-6)	2.44(2.08-2.84)	12(10-14)	2.33(1.91-2.77)	-0.17(-0.88 to 0.54)	140(120-163)	67.05(57.21-77.93)	315(259-372)	60.23(49.57-71.32)	-0.34(-1.06 to 0.38)
Mexico	61(58-65)	0.55(0.52-0.58)	148(124-171)	0.42(0.35-0.49)	-0.91(-1.26 to -0.56)	1689(1600-1782)	14.23(13.46-15.03)	3904(3292-4545)	10.79(9.11-12.55)	-0.91(-1.06 to -0.75)
Micronesia (Federated States of)	1(0-1)	4.36(2.62-6.7)	1(0-1)	3.25(2.04-4.97)	-0.95(-1.04 to -0.87)	18(11-27)	130.04(77.85-200.02)	23(15-36)	94.63(59.24-144.23)	-1.02(-1.09 to -0.95)

location	Incidence					DALYs				
	Num in 1990	ASR in 1990	Num in 2021	ASR in 2021	AAPC	Num in 1990	ASR in 1990	Num in 2021	ASR in 2021	AAPC
Monaco	0(0-0)	0.99(0.65-1.46)	0(0-0)	0.97(0.62-1.45)	-0.09(-0.18 to 0.01)	3(2-5)	21.31(13.93-31.09)	4(2-5)	17.18(11.31-25.29)	-0.72(-0.79 to -0.65)
Mongolia	4(2-5)	1.2(0.76-1.84)	8(5-12)	1.14(0.72-1.67)	-0.2(-0.52 to 0.13)	106(68-163)	35.17(22.56-53.92)	253(162-366)	32.83(21.04-47.5)	-0.25(-0.54 to 0.05)
Montenegro	1(0-1)	0.33(0.26-0.42)	1(1-1)	0.35(0.26-0.45)	0.26(-0.1 to 0.62)	17(14-22)	9.45(7.56-11.88)	24(18-31)	9.22(6.95-12.06)	-0.03(-0.38 to 0.33)
Morocco	165(106-246)	4.1(2.65-6.14)	378(227-586)	3.76(2.27-5.82)	-0.27(-0.38 to -0.17)	4986(3248-7435)	121.01(78.72-180.53)	10944(6555-17057)	105.78(63.54-164.62)	-0.44(-0.54 to -0.34)
Mozambique	5(3-8)	0.29(0.18-0.43)	11(7-16)	0.32(0.2-0.49)	0.39(0.31 to 0.47)	154(96-234)	8.34(5.25-12.64)	336(208-521)	9.48(5.92-14.55)	0.43(0.35 to 0.5)
Myanmar	378(236-568)	5.75(3.61-8.61)	544(347-808)	3.88(2.49-5.75)	-1.27(-1.35 to -1.2)	11441(7158-17202)	166.46(104.41-249.83)	15546(9837-23163)	106.17(67.5-157.75)	-1.46(-1.54 to -1.38)
Namibia	3(2-5)	1.77(1.14-2.64)	7(4-10)	1.57(0.99-2.34)	-0.33(-0.46 to -0.2)	106(68-159)	53.45(34.23-80.32)	206(127-309)	46.82(29.07-69.84)	-0.39(-0.61 to -0.17)
Nauru	0(0-0)	4.88(2.75-8.01)	0(0-0)	3.78(2.02-6.34)	-0.83(-0.89 to -0.78)	2(1-4)	144.33(79.76-238.09)	2(1-3)	110.15(58.82-185.6)	-0.88(-0.94 to -0.82)
Nepal	108(68-164)	3.85(2.41-5.87)	190(120-294)	2.86(1.81-4.41)	-0.94(-1.04 to -0.85)	3395(2136-5127)	114.94(72.31-173.73)	5617(3524-8720)	82.03(51.54-127.08)	-1.08(-1.17 to -0.98)

location	Incidence					DALYs				
	Num in 1990	ASR in 1990	Num in 2021	ASR in 2021	AAPC	Num in 1990	ASR in 1990	Num in 2021	ASR in 2021	AAPC
Netherlands	88(71-108)	1.74(1.4-2.14)	121(89-159)	1.41(1.04-1.86)	-0.67(-0.9 to -0.45)	1774(1451-2143)	35.65(29.17-43.01)	1757(1338-2256)	20.48(15.66-26.27)	-1.8(-1.98 to -1.62)
New Zealand	19(14-26)	1.9(1.42-2.56)	24(17-31)	1.13(0.84-1.48)	-1.67(-2.09 to -1.24)	319(248-409)	31.89(24.73-40.79)	300(224-387)	14.49(10.83-18.67)	-2.53(-3.06 to -2.01)
Nicaragua	2(2-3)	0.58(0.4-0.81)	8(5-11)	0.57(0.38-0.84)	-0.04(-0.32 to 0.23)	67(47-93)	15.53(10.83-21.46)	209(138-307)	15.01(9.92-22.07)	-0.16(-0.42 to 0.11)
Niger	3(2-4)	0.31(0.18-0.5)	8(4-14)	0.29(0.15-0.53)	-0.21(-0.33 to -0.08)	89(51-145)	9.81(5.7-15.87)	252(122-490)	9.16(4.49-17.56)	-0.17(-0.31 to -0.04)
Nigeria	399(301-510)	3.12(2.37-3.98)	760(519-1043)	2.71(1.89-3.68)	-0.48(-0.57 to -0.38)	12340(9298-15878)	93.04(70.32-119.36)	23667(15933-32575)	78.75(53.93-107.62)	-0.56(-0.64 to -0.48)
Niue	0(0-0)	3.24(2.02-4.94)	0(0-0)	2.61(1.61-4.02)	-0.7(-0.8 to -0.61)	1(0-1)	93.17(57.14-141.96)	0(0-1)	71.47(44.07-109.73)	-0.86(-0.92 to -0.79)
North Macedonia	5(4-6)	0.9(0.71-1.14)	9(6-12)	0.98(0.72-1.34)	0.28(-0.14 to 0.69)	145(114-185)	26.16(20.63-33.3)	245(177-337)	26.5(19.14-36.48)	0.21(-0.08 to 0.5)
Northern Mariana Islands	0(0-0)	5.56(3.6-8.39)	1(1-1)	4.97(3.28-7.32)	-0.35(-0.8 to 0.1)	10(6-15)	152.57(97.92-230.1)	24(16-35)	131.85(86.79-191.53)	-0.41(-0.9 to 0.07)
Norway	12(10-13)	0.72(0.63-0.82)	10(8-12)	0.38(0.31-0.47)	-2.04(-2.49 to -1.58)	258(233-286)	16.73(15.09-18.5)	164(140-192)	6.66(5.7-7.81)	-3(-3.4 to -2.61)

location	Incidence					DALYs				
	Num in 1990	ASR in 1990	Num in 2021	ASR in 2021	AAPC	Num in 1990	ASR in 1990	Num in 2021	ASR in 2021	AAPC
Oman	3(2-5)	1.63(1.02-2.49)	6(4-9)	0.92(0.56-1.41)	-1.86(-2.21 to -1.51)	98(61-152)	46.74(29.1-71.82)	160(96-249)	23.01(14.11-35.17)	-2.29(-2.67 to -1.9)
Pakistan	627(472-831)	3.9(2.93-5.18)	1342(964-1862)	3.66(2.63-5.05)	-0.2(-0.25 to -0.15)	19155(14482-25454)	115.4(87.02-153.43)	41805(29989-58124)	107.2(77.12-148.58)	-0.23(-0.28 to -0.18)
Palau	0(0-0)	0.18(0.1-0.32)	0(0-0)	0.13(0.08-0.21)	-1.11(-1.31 to -0.9)	0(0-0)	4.9(2.69-8.64)	0(0-0)	3.24(1.9-5.23)	-1.36(-1.61 to -1.1)
Palestine	2(2-4)	1.03(0.66-1.52)	5(3-7)	0.7(0.48-1.01)	-1.24(-1.35 to -1.13)	65(42-98)	27.07(17.39-40.46)	135(91-194)	17.51(11.8-25.05)	-1.43(-1.64 to -1.22)
Panama	4(3-4)	0.93(0.79-1.08)	10(8-13)	0.86(0.63-1.1)	-0.23(-0.5 to 0.04)	98(83-113)	23.92(20.39-27.74)	257(189-333)	21.16(15.56-27.4)	-0.37(-0.65 to -0.08)
Papua New Guinea	17(9-29)	3.02(1.6-5.2)	40(20-71)	2.5(1.3-4.46)	-0.56(-0.62 to -0.51)	523(269-911)	89.37(46.55-155.06)	1242(625-2242)	72.81(37.25-130.49)	-0.62(-0.68 to -0.57)
Paraguay	2(1-3)	0.29(0.2-0.41)	8(5-13)	0.5(0.31-0.77)	1.82(1.56 to 2.08)	54(37-78)	8.55(5.88-12.18)	242(148-368)	14.39(8.82-21.84)	1.7(1.45 to 1.96)
Peru	13(9-18)	0.4(0.29-0.55)	32(20-48)	0.35(0.22-0.52)	-0.54(-1.84 to 0.77)	351(251-481)	10.54(7.53-14.41)	767(490-1162)	8.27(5.29-12.5)	-0.64(-1.84 to 0.58)
Philippines	382(320-461)	4.59(3.84-5.58)	1109(906-1337)	4.61(3.79-5.54)	0.03(-0.03 to 0.08)	11281(9489-13598)	126.79(106.33-153.45)	32095(26068-38963)	127.46(103.9-154.25)	0.03(-0.03 to 0.08)

location	Incidence					DALYs				
	Num in 1990	ASR in 1990	Num in 2021	ASR in 2021	AAPC	Num in 1990	ASR in 1990	Num in 2021	ASR in 2021	AAPC
Poland	132(123-141)	1.13(1.05-1.21)	187(165-209)	1.04(0.92-1.16)	-0.37(-0.87 to 0.12)	3838(3590-4083)	33.23(31.07-35.37)	4821(4276-5355)	27.96(24.79-31.07)	-0.66(-1.19 to -0.12)
Portugal	69(55-88)	1.89(1.48-2.39)	79(57-108)	1.46(1.05-2)	-0.93(-1.33 to -0.52)	1876(1477-2355)	51.85(40.76-65.26)	1716(1258-2325)	32.51(23.85-44.02)	-1.62(-2.14 to -1.1)
Puerto Rico	13(10-17)	1.37(1.05-1.77)	20(14-28)	1.16(0.8-1.6)	-0.63(-1.01 to -0.26)	338(258-439)	35.14(26.81-45.59)	448(314-617)	27.4(19.13-37.81)	-0.89(-1.28 to -0.49)
Qatar	0(0-1)	1.54(0.99-2.29)	3(2-5)	0.88(0.51-1.47)	-1.9(-2.59 to -1.2)	14(9-21)	38.59(24.72-57.25)	70(39-123)	19(10.93-32.58)	-2.33(-3.02 to -1.63)
Republic of Korea	127(89-178)	1.48(1.04-2.08)	219(152-303)	0.85(0.59-1.18)	-1.83(-2.05 to -1.62)	3774(2629-5295)	41.16(28.72-57.8)	4851(3438-6677)	18.78(13.31-25.82)	-2.51(-2.67 to -2.35)
Republic of Moldova	25(21-30)	2.03(1.71-2.38)	34(27-41)	2.11(1.69-2.58)	0.07(-0.54 to 0.69)	764(644-896)	59.99(50.5-70.43)	966(777-1198)	61.47(49.45-76.18)	0.02(-0.64 to 0.68)
Romania	80(62-99)	1.03(0.81-1.29)	187(137-249)	2.1(1.53-2.79)	2.36(2.03 to 2.69)	2371(1863-2961)	30.31(23.79-37.88)	5202(3809-6907)	60.31(44.11-80.15)	2.27(1.91 to 2.63)
Russian Federation	452(424-502)	0.91(0.85-1.01)	529(470-601)	0.84(0.74-0.95)	0.03(-1 to 1.07)	13764(12897-15323)	27.53(25.78-30.72)	15215(13537-17300)	24.65(21.94-28.01)	-0.06(-1.12 to 1.01)
Rwanda	55(36-80)	6.5(4.28-9.47)	77(46-122)	4.05(2.46-6.36)	-1.5(-1.63 to -1.36)	1732(1130-2541)	199.04(130.01-291.75)	2390(1404-3831)	119.46(70.84-190.12)	-1.61(-1.77 to -1.45)

location	Incidence					DALYs				
	Num in 1990	ASR in 1990	Num in 2021	ASR in 2021	AAPC	Num in 1990	ASR in 1990	Num in 2021	ASR in 2021	AAPC
Saint Kitts and Nevis	0(0-0)	2.13(1.79-2.52)	0(0-1)	2.29(1.74-2.89)	0.22(-0.21 to 0.65)	5(4-6)	57.33(48.07-68.07)	12(9-16)	58.74(44.44-74.46)	0.07(-0.37 to 0.5)
Saint Lucia	0(0-1)	2.22(1.89-2.58)	2(1-2)	2.23(1.71-2.83)	0.05(-0.17 to 0.26)	13(11-15)	58.19(49.55-67.84)	39(30-50)	57.49(44.09-72.86)	0.05(-0.2 to 0.3)
Saint Vincent and the Grenadines	0(0-0)	1.55(1.29-1.86)	1(1-1)	1.9(1.53-2.31)	0.55(0 to 1.1)	8(6-9)	40.61(33.87-48.78)	20(16-25)	50.13(40.79-61.5)	0.6(0.01 to 1.19)
Samoa	1(1-2)	5.12(3.35-7.63)	2(1-3)	4.54(2.94-6.69)	-0.39(-0.41 to -0.37)	38(25-57)	149.75(97.32-223.93)	57(37-86)	129.67(83.64-193.25)	-0.47(-0.5 to -0.44)
San Marino	0(0-0)	2.84(1.88-4.15)	0(0-0)	1.5(0.79-2.52)	-2.28(-2.73 to -1.83)	5(3-7)	59.11(39.73-84.93)	5(2-8)	26.64(13.99-44.96)	-2.8(-3.02 to -2.59)
Sao Tome and Principe	0(0-0)	0.04(0.03-0.06)	0(0-0)	0.04(0.03-0.06)	0.15(0.01 to 0.3)	0(0-0)	1.16(0.75-1.73)	0(0-1)	1.2(0.75-1.85)	0.1(-0.05 to 0.25)
Saudi Arabia	77(49-116)	4.4(2.8-6.63)	207(132-313)	2.9(1.88-4.34)	-1.34(-1.47 to -1.2)	2421(1524-3677)	129.69(81.82-195.65)	5844(3736-8859)	75.51(48.71-113.08)	-1.77(-1.9 to -1.63)
Senegal	3(2-5)	0.33(0.2-0.52)	8(4-14)	0.34(0.19-0.59)	0.03(-0.05 to 0.12)	103(62-161)	10.57(6.4-16.45)	273(141-483)	10.94(5.74-19.19)	0.08(0 to 0.17)
Serbia	41(28-61)	1.28(0.85-1.9)	46(31-67)	1.13(0.75-1.65)	-0.35(-0.78 to 0.09)	1215(814-1800)	36.2(24.18-53.76)	1158(776-1680)	29.26(19.57-42.44)	-0.65(-1.07 to -0.22)

location	Incidence					DALYs				
	Num in 1990	ASR in 1990	Num in 2021	ASR in 2021	AAPC	Num in 1990	ASR in 1990	Num in 2021	ASR in 2021	AAPC
Seychelles	1(0-1)	4.84(3.19-7.17)	2(1-2)	4.5(2.92-6.71)	-0.28(-0.51 to -0.04)	21(14-31)	138.52(90.94-206.27)	43(27-63)	118.86(76.79-176.31)	-0.52(-0.73 to -0.31)
Sierra Leone	2(1-3)	0.34(0.21-0.52)	4(2-7)	0.35(0.2-0.57)	0.15(-0.07 to 0.38)	62(38-96)	10.75(6.54-16.71)	140(79-234)	11.33(6.44-18.76)	0.19(-0.02 to 0.4)
Singapore	119(98-144)	18.18(14.8-22.05)	185(137-243)	7.83(5.76-10.29)	-2.66(-3.01 to -2.32)	3521(2909-4246)	515.1(424.13-622.73)	4136(3068-5389)	172.78(128.13-225.25)	-3.42(-3.79 to -3.05)
Slovakia	33(23-46)	2.13(1.47-2.93)	33(21-51)	1.36(0.86-2.08)	-1.43(-1.76 to -1.1)	1019(703-1408)	66.35(45.78-91.72)	936(583-1446)	39.32(24.39-60.82)	-1.67(-2.04 to -1.31)
Slovenia	10(7-12)	1.41(1.07-1.82)	5(4-7)	0.48(0.33-0.68)	-3.58(-4.13 to -3.02)	275(210-354)	40.87(31.17-52.68)	120(81-171)	11.52(7.8-16.44)	-4.18(-4.73 to -3.64)
Solomon Islands	2(1-3)	3.8(1.93-6.29)	3(2-6)	3.23(1.92-5.15)	-0.54(-0.71 to -0.37)	50(24-84)	113.12(55.95-188.85)	111(66-177)	96.05(57.31-153.1)	-0.54(-0.72 to -0.36)
Somalia	38(22-60)	4.93(2.95-7.8)	83(48-139)	4.44(2.61-7.3)	-0.33(-0.38 to -0.28)	1255(736-2001)	150.55(89.39-237.77)	2710(1558-4583)	134.32(78.59-224.19)	-0.37(-0.43 to -0.3)
South Africa	93(73-116)	1.63(1.28-2.05)	205(178-234)	1.57(1.37-1.79)	-0.19(-0.77 to 0.38)	2699(2135-3395)	45.85(36.17-57.79)	5822(5052-6688)	42.93(37.3-49.23)	-0.21(-0.92 to 0.5)
South Sudan	30(17-50)	4.1(2.37-6.84)	47(26-80)	3.8(2.16-6.47)	-0.23(-0.32 to -0.14)	907(521-1541)	122.76(70.59-207.85)	1499(838-2637)	112.81(63.76-196.74)	-0.26(-0.35 to -0.17)

location	Incidence					DALYs				
	Num in 1990	ASR in 1990	Num in 2021	ASR in 2021	AAPC	Num in 1990	ASR in 1990	Num in 2021	ASR in 2021	AAPC
Spain	330(270-402)	2.39(1.95-2.91)	336(242-455)	1.46(1.05-1.97)	-1.66(-2.06 to -1.26)	7748(6405-9309)	56.79(46.95-68.23)	6071(4479-8090)	26.8(19.8-35.67)	-2.51(-2.92 to -2.1)
Sri Lanka	92(62-133)	3.13(2.12-4.51)	165(94-265)	2.18(1.24-3.5)	-1.14(-1.61 to -0.66)	2567(1726-3696)	82.58(55.67-118.85)	4041(2281-6513)	52.68(29.81-84.92)	-1.44(-1.91 to -0.96)
Sudan	39(22-65)	1.53(0.88-2.55)	47(29-74)	0.85(0.52-1.32)	-1.87(-1.91 to -1.83)	1179(667-1945)	43.93(24.92-72.73)	1388(828-2194)	23.24(14.03-36.61)	-2.04(-2.09 to -1.99)
Suriname	1(1-2)	1.9(1.38-2.54)	4(2-6)	2.12(1.35-3.14)	0.43(-0.01 to 0.87)	39(28-51)	52.01(37.82-68.88)	108(69-160)	58(37.19-86.17)	0.44(-0.03 to 0.9)
Sweden	27(20-34)	0.73(0.56-0.94)	25(18-33)	0.49(0.36-0.65)	-1.22(-2.23 to -0.2)	430(338-542)	12.07(9.48-15.21)	332(244-434)	6.57(4.87-8.58)	-1.92(-2.69 to -1.14)
Switzerland	49(39-61)	1.92(1.51-2.38)	28(20-38)	0.63(0.45-0.85)	-3.7(-4.17 to -3.22)	1279(1014-1581)	51.18(40.6-63.19)	614(441-833)	14.39(10.38-19.46)	-4.21(-4.72 to -3.69)
Syrian Arab Republic	7(5-9)	0.47(0.34-0.64)	12(8-17)	0.32(0.22-0.43)	-1.33(-1.67 to -0.99)	202(146-271)	13.06(9.41-17.48)	324(225-447)	7.89(5.53-10.82)	-1.66(-1.92 to -1.39)
Taiwan (Province of China)	792(673-921)	17.23(14.63-20.05)	1057(774-1419)	9.67(7.06-13.01)	-1.87(-2.2 to -1.53)	20145(17375-23182)	431.2(371.73-496.54)	20561(15496-26770)	184.82(139.47-240.5)	-2.73(-3.06 to -2.4)
Tajikistan	14(8-23)	1.85(1.08-3.06)	20(12-31)	1.19(0.71-1.88)	-1.41(-1.74 to -1.08)	398(239-642)	51.31(30.59-83.29)	602(356-961)	32.91(19.47-52.37)	-1.43(-1.7 to -1.16)

location	Incidence					DALYs				
	Num in 1990	ASR in 1990	Num in 2021	ASR in 2021	AAPC	Num in 1990	ASR in 1990	Num in 2021	ASR in 2021	AAPC
Thailand	553(396-754)	5.33(3.81-7.26)	1300(864-1875)	4.26(2.84-6.15)	-0.7(-0.88 to -0.52)	16040(11462-21806)	147.11(105.06-199.79)	31585(21084-45517)	102.93(68.74-148.19)	-1.12(-1.32 to -0.92)
Timor-Leste	4(2-6)	4.43(2.58-7.38)	10(6-15)	4.07(2.51-6.38)	-0.3(-0.44 to -0.16)	116(66-199)	126.65(72.76-213.4)	275(168-438)	112.35(68.74-178.44)	-0.43(-0.57 to -0.29)
Togo	1(1-2)	0.35(0.22-0.52)	5(3-9)	0.4(0.24-0.65)	0.5(0.38 to 0.63)	45(28-68)	11.05(7.06-16.74)	184(107-294)	13.02(7.66-20.74)	0.55(0.42 to 0.67)
Tokelau	0(0-0)	3.56(2.13-5.56)	0(0-0)	2.52(1.51-3.95)	-1.11(-1.18 to -1.04)	0(0-1)	102.89(60.89-162.59)	0(0-0)	69.61(41.1-109.78)	-1.25(-1.33 to -1.18)
Tonga	0(0-1)	2.66(1.59-4.28)	1(0-1)	2.28(1.36-3.65)	-0.5(-0.61 to -0.38)	13(8-20)	76.07(44.98-122.34)	15(9-24)	63.19(37.48-101.75)	-0.59(-0.75 to -0.43)
Trinidad and Tobago	2(2-3)	1.08(0.93-1.24)	6(5-8)	1.19(0.85-1.58)	0.49(0.14 to 0.84)	65(56-75)	28.78(24.81-33.09)	169(120-226)	31.78(22.61-42.57)	0.5(0.13 to 0.87)
Tunisia	109(72-162)	7.64(5.02-11.3)	226(136-353)	5.96(3.61-9.3)	-0.81(-0.92 to -0.7)	3147(2081-4667)	213.6(141.25-316.66)	5881(3553-9127)	152.76(92.44-236.84)	-1.1(-1.24 to -0.96)
Turkey	249(163-369)	2.54(1.65-3.78)	403(272-573)	1.53(1.04-2.18)	-1.65(-1.85 to -1.43)	7384(4801-10958)	71.5(46.48-106.37)	10120(6846-14502)	37.79(25.59-54.08)	-2.05(-2.23 to -1.88)
Turkmenistan	6(5-7)	1.16(1-1.34)	15(11-20)	1.25(0.93-1.67)	0.3(-0.28 to 0.88)	189(161-218)	33.6(28.79-38.82)	453(336-619)	35.88(26.69-48.67)	0.28(-0.36 to 0.91)

location	Incidence					DALYs				
	Num in 1990	ASR in 1990	Num in 2021	ASR in 2021	AAPC	Num in 1990	ASR in 1990	Num in 2021	ASR in 2021	AAPC
Tuvalu	0(0-0)	3.77(2.36-5.61)	0(0-0)	2.91(1.9-4.34)	-0.83(-0.87 to -0.78)	2(1-3)	112.4(69.95-168.32)	3(2-4)	83.99(54.45-125.83)	-0.93(-0.98 to -0.89)
Uganda	167(109-247)	8.93(5.82-13.14)	341(214-523)	7.69(4.87-11.69)	-0.5(-0.65 to -0.35)	5207(3368-7685)	266.81(173.08-393.4)	10703(6573-16446)	226.38(140.6-346.04)	-0.55(-0.71 to -0.39)
Ukraine	162(112-241)	0.82(0.57-1.22)	226(143-344)	1.15(0.72-1.74)	1.12(0.27 to 1.97)	4982(3455-7501)	25.36(17.56-38.35)	6768(4287-10367)	35.16(22.2-53.95)	1.08(0.21 to 1.96)
United Arab Emirates	3(2-6)	2.56(1.43-4.33)	20(13-32)	1.58(1.03-2.38)	-1.47(-2.1 to -0.83)	112(59-199)	71.22(38.83-122.64)	615(377-956)	37.31(24.26-56.42)	-2.18(-2.72 to -1.63)
United Kingdom	294(278-310)	1.32(1.25-1.39)	346(321-369)	1.12(1.05-1.2)	-0.54(-0.83 to -0.25)	5826(5558-6086)	26.8(25.59-27.99)	5462(5121-5789)	17.87(16.83-18.91)	-1.32(-1.6 to -1.04)
United Republic of Tanzania	149(92-225)	4.66(2.9-7.04)	283(166-450)	3.69(2.2-5.82)	-0.75(-0.83 to -0.67)	4607(2867-7063)	139.41(86.92-213.27)	8825(5113-14267)	108.99(64.02-174.64)	-0.8(-0.88 to -0.72)
United States of America	1418(1340-1492)	1.82(1.72-1.91)	1880(1741-1999)	1.3(1.21-1.39)	-1.11(-1.32 to -0.9)	23877(22774-24919)	31(29.62-32.34)	26251(24662-27661)	18.19(17.14-19.16)	-1.72(-1.87 to -1.58)
United States Virgin Islands	0(0-1)	2.05(1.41-2.9)	1(0-1)	1.08(0.67-1.66)	-2.13(-2.58 to -1.68)	13(9-19)	53.22(36.23-75.73)	12(8-19)	27.18(16.73-42.15)	-2.22(-2.68 to -1.77)
Uruguay	19(14-24)	1.81(1.38-2.34)	15(11-20)	1.09(0.79-1.46)	-1.61(-2.1 to -1.13)	495(378-641)	48.87(37.28-63.32)	359(263-481)	26.84(19.62-35.94)	-1.92(-2.4 to -1.43)

location	Incidence					DALYs				
	Num in 1990	ASR in 1990	Num in 2021	ASR in 2021	AAPC	Num in 1990	ASR in 1990	Num in 2021	ASR in 2021	AAPC
Uzbekistan	35(23-53)	1.08(0.72-1.67)	92(65-130)	1.2(0.84-1.68)	0.32(-0.02 to 0.66)	1026(693-1518)	31.23(21.05-46.66)	2762(1949-3885)	33.64(23.74-47.29)	0.23(-0.13 to 0.59)
Vanuatu	1(0-1)	3.35(1.98-5.39)	1(1-2)	2.8(1.78-4.22)	-0.57(-0.77 to -0.38)	19(11-32)	98.6(57.67-160.48)	46(29-71)	82.03(52.02-124.64)	-0.61(-0.86 to -0.36)
Venezuela (Bolivarian Republic of)	19(17-22)	0.75(0.64-0.86)	72(51-98)	0.86(0.61-1.17)	0.42(0.11 to 0.73)	533(461-614)	19.86(17.11-22.89)	1920(1351-2669)	22.33(15.74-30.99)	0.32(0 to 0.64)
Viet Nam	930(619-1341)	8.39(5.58-12.1)	2471(1520-3738)	8.28(5.12-12.47)	-0.03(-0.11 to 0.04)	27058(17995-39372)	241.57(160.73-351.75)	66017(40234-101797)	215.47(131.89-331.01)	-0.36(-0.44 to -0.27)
Yemen	22(13-36)	1.57(0.92-2.53)	42(25-64)	1.06(0.64-1.62)	-1.25(-1.35 to -1.15)	677(388-1102)	45.45(26.29-73.54)	1229(739-1880)	29.09(17.62-44.49)	-1.41(-1.52 to -1.29)
Zambia	39(26-57)	4.59(3-6.73)	139(51-274)	6.24(2.42-12.02)	1.05(0.88 to 1.21)	1240(807-1834)	137.89(89.88-203.86)	4534(1609-8997)	191.22(70.77-373.59)	1.12(0.95 to 1.29)
Zimbabwe	19(13-28)	1.67(1.13-2.38)	47(30-70)	2.22(1.43-3.29)	0.93(0.44 to 1.42)	581(392-832)	47.85(32.33-68.46)	1489(939-2227)	66.68(42.4-99.22)	1.06(0.53 to 1.6)

S8 Table: Incidence and DALYs of Lip and oral cavity cancer in middle-aged and older adults, and their average annual percentage changes in 204 countries/territories from 1990 to 2021.

location	Incidence					DALYs				
	Num in 1990	ASR in 1990	Num in 2021	ASR in 2021	AAPC	Num in 1990	ASR in 1990	Num in 2021	ASR in 2021	AAPC
Afghanistan	60(34-107)	3.15(1.81-5.51)	88(49-146)	3.38(1.98-5.36)	0.22(0.16 to 0.29)	1335(738-2444)	66.44(37.36-119.29)	1883(1023-3179)	64.06(36.53-103)	-0.11(-0.15 to -0.07)
Albania	42(30-56)	7.49(5.39-10.12)	104(68-151)	8.8(5.77-12.8)	0.45(0 to 0.91)	799(577-1084)	135.9(98.37-183.93)	1369(895-1982)	117.79(76.91-170.68)	-0.55(-0.97 to -0.12)
Algeria	135(90-195)	4.48(3.03-6.44)	425(272-636)	4.58(2.97-6.81)	0.07(-0.02 to 0.16)	2464(1652-3556)	75.45(50.99-108.59)	5959(3839-8867)	60.5(39.24-89.42)	-0.71(-0.81 to -0.61)
American Samoa	0(0-0)	2.63(1.79-3.7)	0(0-1)	3.39(2.34-4.76)	0.93(0.21 to 1.65)	2(2-3)	39.44(27.29-55.01)	7(5-9)	49.98(35.42-68.97)	0.88(0.2 to 1.55)
Andorra	2(1-3)	11.06(6.25-18.31)	4(2-6)	8.25(4.45-14.09)	-1.01(-1.37 to -0.66)	18(10-29)	113.84(65.33-185.16)	30(17-49)	69.68(39.04-115.02)	-1.71(-1.99 to -1.43)
Angola	72(46-109)	6.82(4.34-10.3)	270(172-403)	8.41(5.36-12.57)	0.63(0.57 to 0.7)	1768(1116-2697)	151.13(95.98-229.41)	5915(3799-8866)	166.49(107.17-248.99)	0.28(0.09 to 0.47)
Antigua and Barbuda	2(1-2)	10.91(9.19-12.79)	3(2-3)	9.53(7.89-11.37)	-0.45(-1.41 to 0.52)	24(20-27)	173.93(149.86-200.48)	40(34-47)	130.89(111.23-152.74)	-0.87(-1.88 to 0.14)
Argentina	895(734-1087)	10.11(8.29-12.28)	1354(1092-1654)	8.97(7.23-10.96)	-0.32(-0.96 to 0.32)	14418(12029-17213)	162.49(135.54-193.98)	17267(14348-20590)	116.26(96.57-138.75)	-0.86(-1.54 to -0.17)

location	Incidence					DALYs				
	Num in 1990	ASR in 1990	Num in 2021	ASR in 2021	AAPC	Num in 1990	ASR in 1990	Num in 2021	ASR in 2021	AAPC
Armenia	37(31-44)	4.87(4.06-5.82)	78(63-94)	6.49(5.26-7.86)	0.99(0.49 to 1.48)	705(591-836)	87.94(73.81-104.27)	1145(941-1352)	96.31(79.04-113.83)	0.33(-0.16 to 0.82)
Australia	1316(1069-1615)	25.05(20.31-30.77)	2817(2213-3507)	23.64(18.6-29.43)	-0.2(-0.91 to 0.52)	8441(7170-9874)	162.38(137.81-190.03)	13306(11010-15881)	113.78(94.34-135.76)	-1.22(-1.81 to -0.63)
Austria	398(326-482)	13.41(10.95-16.31)	632(503-777)	13.53(10.79-16.62)	0.07(-0.36 to 0.51)	5884(4914-6988)	205.55(171.49-244.65)	6453(5299-7699)	143.56(118.13-171.52)	-1.18(-1.73 to -0.62)
Azerbaijan	46(30-67)	3.33(2.21-4.89)	104(63-160)	3.61(2.24-5.51)	0.26(0.05 to 0.47)	944(633-1377)	65.12(43.52-95.28)	1849(1132-2857)	59.8(37.05-91.7)	-0.26(-0.45 to -0.07)
Bahamas	7(6-8)	16.25(13.83-19.06)	17(13-22)	14.69(11.24-18.77)	-0.16(-0.92 to 0.59)	124(107-144)	286.2(247.65-330.8)	276(213-355)	229.94(178.02-293.91)	-0.53(-0.75 to -0.3)
Bahrain	4(2-5)	8.65(6-12.14)	20(11-31)	9.03(5.47-13.82)	0.22(-0.26 to 0.7)	61(42-85)	131.66(91.39-183.07)	250(139-395)	97.02(56.9-149.84)	-0.92(-1.36 to -0.47)
Bangladesh	3235(1997-4842)	25.54(15.74-38.14)	8802(5067-13815)	23.3(13.56-36.36)	-0.27(-0.46 to -0.09)	72780(44932-110249)	551.68(339.9-833.97)	152998(87152-241590)	393.7(225.98-619.49)	-1.06(-1.18 to -0.94)
Barbados	10(8-11)	12.72(10.88-14.78)	16(12-21)	11.36(8.45-14.72)	-0.19(-1.01 to 0.64)	143(126-162)	197.15(172.77-223.72)	215(162-278)	152.53(114.73-196.96)	-0.72(-1.49 to 0.06)
Belarus	756(609-927)	20.97(16.9-25.67)	1033(763-1370)	24.22(17.83-32.18)	0.53(-0.36 to 1.43)	13834(11141-16948)	382.53(308.39-468.31)	13759(10285-18128)	329.62(245.97-434.71)	-0.48(-1.17 to 0.22)
Belgium	635(510-784)	16.16(12.93-19.98)	1044(821-1307)	17.9(14.1-22.46)	0.3(-0.32 to 0.92)	6906(5778-8164)	181.35(151.43-214.69)	8656(7088-10418)	154.21(126.67-185.78)	-0.55(-1.23 to 0.13)

location	Incidence					DALYs				
	Num in 1990	ASR in 1990	Num in 2021	ASR in 2021	AAPC	Num in 1990	ASR in 1990	Num in 2021	ASR in 2021	AAPC
Belize	1(1-2)	5.73(4.95-6.63)	5(4-6)	5.81(4.77-6.99)	0.03(-0.54 to 0.6)	25(22-28)	98.55(86.41-112.08)	80(66-94)	92.81(76.63-109.66)	-0.19(-0.77 to 0.39)
Benin	19(13-26)	3.51(2.4-4.99)	64(40-98)	4.61(2.88-6.96)	0.89(0.74 to 1.05)	405(276-576)	74.1(50.59-105.33)	1337(812-2065)	89.04(54.75-136.43)	0.61(0.43 to 0.79)
Bermuda	4(3-5)	24.64(19.88-30.3)	6(5-8)	17(12.64-22.68)	-1.23(-1.87 to -0.58)	57(47-70)	335.65(273.77-407.76)	59(44-76)	163.11(122.32-212.05)	-2.34(-2.71 to -1.98)
Bhutan	14(9-22)	21.34(12.81-32.45)	36(22-53)	21.82(13.82-32.6)	0.09(-0.02 to 0.21)	332(195-510)	458.32(271.75-703.12)	623(388-950)	372.81(232.79-566.33)	-0.65(-0.72 to -0.57)
Bolivia (Plurinational State of)	46(29-71)	5.52(3.53-8.46)	143(87-220)	5.9(3.62-9.05)	0.21(0.1 to 0.33)	984(627-1504)	111.3(71.12-169.84)	2500(1519-3804)	98.98(60.47-150.02)	-0.37(-0.41 to -0.34)
Bosnia and Herzegovina	100(81-123)	8.44(6.8-10.4)	190(139-254)	11.4(8.28-15.29)	1.01(0.77 to 1.26)	1932(1569-2362)	154.19(125.2-188.52)	2630(1935-3494)	162.54(118.9-217.29)	0.2(0.02 to 0.39)
Botswana	21(13-32)	13.38(8.47-20.27)	54(32-84)	13.03(7.93-20)	-0.13(-0.43 to 0.18)	460(284-707)	280.06(174.53-427.88)	1108(639-1770)	250.43(148.06-393.57)	-0.35(-0.6 to -0.1)
Brazil	2930(2694-3176)	12.08(11.05-13.1)	8752(7906-9563)	12.56(11.33-13.73)	0.13(0.07 to 0.2)	56914(52607-61539)	223.68(206.17-241.84)	138020(126349-149772)	195.81(179.03-212.52)	-0.43(-0.5 to -0.36)
Brunei Darussalam	4(3-6)	15.92(11.15-22.42)	14(10-19)	14.5(10.29-20)	-0.3(-0.53 to -0.07)	66(47-93)	237.49(168.06-333.85)	182(129-249)	177.48(126.52-241.83)	-0.93(-1.11 to -0.74)
Bulgaria	378(304-463)	11.52(9.3-14.09)	699(540-886)	19.98(15.39-25.38)	1.93(0.73 to 3.13)	5068(4207-6041)	154.66(128.38-184.33)	7466(5980-9104)	224.93(179.56-274.7)	1.36(0.08 to 2.65)

location	Incidence					DALYs				
	Num in 1990	ASR in 1990	Num in 2021	ASR in 2021	AAPC	Num in 1990	ASR in 1990	Num in 2021	ASR in 2021	AAPC
Burkina Faso	44(28-64)	3.76(2.45-5.51)	121(77-182)	4.91(3.16-7.37)	0.85(0.65 to 1.05)	968(624-1414)	78.36(50.72-114.39)	2574(1622-3889)	97.53(62.09-146.81)	0.7(0.51 to 0.89)
Burundi	82(55-119)	13.07(8.85-18.76)	134(82-203)	10.04(6.27-15.07)	-0.85(-0.91 to -0.78)	1938(1304-2807)	296.89(200.36-428.6)	3070(1859-4763)	213.59(131.38-327.74)	-1.07(-1.14 to -1)
Cabo Verde	0(0-1)	0.6(0.41-0.84)	22(14-33)	17.36(11.13-26.42)	11.65(11.19 to 12.11)	7(5-9)	11.24(7.69-15.93)	357(227-543)	274.31(174.86-416.36)	11.01(10.66 to 11.37)
Cambodia	109(70-166)	9.34(6.07-14.02)	398(254-591)	12.15(7.86-17.81)	0.86(0.82 to 0.89)	2363(1496-3645)	189.05(120.97-289.28)	7242(4566-10797)	208.07(132.98-307.3)	0.32(0.26 to 0.39)
Cameroon	53(37-74)	4.31(3.02-6.06)	198(125-300)	5.75(3.68-8.65)	0.92(0.84 to 0.99)	1162(814-1635)	88.78(62.42-124.61)	4125(2605-6298)	110.26(70.53-166.68)	0.67(0.55 to 0.79)
Canada	2073(1680-2532)	23.74(19.23-29.03)	3116(2472-3877)	16.03(12.72-19.95)	-1.22(-1.62 to -0.83)	16629(14219-19407)	193.31(165.13-225.71)	20264(16893-24097)	107.02(89.47-127.13)	-1.93(-2.57 to -1.29)
Central African Republic	23(14-38)	7.42(4.75-11.93)	42(26-67)	6.94(4.35-10.76)	-0.21(-0.33 to -0.09)	575(358-975)	168.06(106.45-278.59)	1070(657-1734)	155.59(97.49-245.93)	-0.24(-0.36 to -0.13)
Chad	22(15-32)	2.95(1.95-4.29)	73(47-106)	4.56(2.96-6.66)	1.44(1.38 to 1.51)	488(325-706)	62.99(41.87-91.32)	1658(1062-2426)	96.33(62.33-140.45)	1.39(1.21 to 1.58)
Chile	175(143-213)	6.4(5.25-7.79)	416(334-513)	5.89(4.72-7.25)	-0.11(-0.83 to 0.6)	2650(2209-3161)	95.39(79.5-113.69)	4272(3552-5106)	60.79(50.53-72.68)	-1.33(-2.05 to -0.6)
China	12405(10430-14526)	5.57(4.7-6.49)	51824(40415-65299)	8.68(6.79-10.9)	1.46(1.3 to 1.62)	236662(198073-278431)	100.36(84.2-117.69)	559743(431410-711873)	92.18(71.19-116.83)	-0.27(-0.44 to -0.1)

location	Incidence					DALYs				
	Num in 1990	ASR in 1990	Num in 2021	ASR in 2021	AAPC	Num in 1990	ASR in 1990	Num in 2021	ASR in 2021	AAPC
Colombia	359(305-418)	8.01(6.78-9.31)	1017(779-1283)	6.74(5.17-8.5)	-0.58(-0.89 to -0.28)	6055(5207-6987)	128.82(110.69-148.55)	12510(9693-15699)	82.46(63.94-103.4)	-1.44(-2.11 to -0.77)
Comoros	6(4-10)	11.8(7.69-17.44)	15(10-23)	11.11(7.25-16.6)	-0.22(-0.53 to 0.09)	148(94-222)	254.27(164.1-377.02)	311(198-473)	218.35(140.08-329.44)	-0.52(-0.93 to -0.1)
Congo	25(15-39)	8.67(5.48-13.4)	69(44-102)	9.28(6.09-13.56)	0.25(0.07 to 0.43)	563(340-911)	184.32(113.52-293.32)	1454(923-2164)	177.11(114.89-259.81)	-0.12(-0.36 to 0.11)
Cook Islands	0(0-0)	6.06(3.98-8.93)	1(0-1)	7.98(5.24-11.64)	0.89(0.75 to 1.04)	3(2-5)	98.22(64.31-146.08)	7(5-10)	100.38(65.71-146.59)	0.08(-0.04 to 0.2)
Costa Rica	40(33-48)	8.72(7.17-10.47)	108(85-135)	7.19(5.66-8.96)	-0.69(-0.96 to -0.42)	574(482-678)	122.17(102.44-144.26)	1279(1027-1562)	84.59(67.99-103.21)	-1.17(-1.56 to -0.79)
Coted'Ivoire	73(49-104)	6.59(4.47-9.31)	236(140-365)	7.53(4.61-11.42)	0.42(0.32 to 0.53)	1672(1120-2405)	137.35(93.03-195.71)	5012(2903-7887)	146.11(87.55-226.04)	0.19(0.08 to 0.3)
Croatia	432(351-525)	25.11(20.44-30.48)	524(416-651)	22.66(17.97-28.18)	-0.14(-0.25 to -0.04)	6378(5265-7652)	360.17(297.8-431.29)	4762(3893-5771)	219.95(179.46-266.8)	-1.54(-1.63 to -1.44)
Cuba	472(389-563)	16.94(13.97-20.21)	1050(808-1344)	19.29(14.82-24.71)	0.49(-0.43 to 1.42)	6467(5436-7599)	233.26(196.04-274.12)	12885(10075-16174)	238.34(186.22-299.38)	0.3(0.07 to 0.54)
Cyprus	20(13-28)	9.95(6.78-14.26)	52(35-75)	9.56(6.4-13.7)	-0.15(-0.47 to 0.16)	240(165-336)	116.92(80.34-164.21)	425(294-604)	79.55(54.67-113.4)	-1.23(-1.64 to -0.82)
Czechia	485(408-572)	13.36(11.2-15.77)	841(681-1028)	15.35(12.4-18.79)	0.58(0.39 to 0.77)	9177(7778-10723)	258.83(218.87-302.97)	11066(9119-13365)	213.82(175.77-258.79)	-0.67(-1 to -0.34)

location	Incidence					DALYs				
	Num in 1990	ASR in 1990	Num in 2021	ASR in 2021	AAPC	Num in 1990	ASR in 1990	Num in 2021	ASR in 2021	AAPC
Democratic People's Republic of Korea	253(160-379)	5.48(3.5-8.2)	582(359-884)	6.19(3.83-9.42)	0.4(0.34 to 0.46)	4662(2924-7013)	95(60.01-142.28)	9213(5661-14151)	95.94(59.14-147.27)	0.04(0 to 0.07)
Democratic Republic of the Congo	264(176-385)	6.36(4.19-9.28)	688(435-1008)	6.94(4.35-10.24)	0.29(0.17 to 0.41)	6135(4075-8969)	134.82(89.23-196.89)	15224(9674-22376)	139.21(88.21-205.04)	0.09(0.01 to 0.17)
Denmark	209(177-244)	10.33(8.75-12.06)	465(384-552)	15.16(12.57-18.02)	1.31(0.49 to 2.15)	2370(2119-2645)	121.7(108.83-135.84)	3808(3326-4288)	129.73(113.77-145.92)	0.23(-0.41 to 0.86)
Djibouti	5(3-8)	12.68(8.12-19.23)	22(13-35)	12.52(7.66-19.06)	-0.06(-0.2 to 0.07)	115(71-182)	269.41(169.85-417.16)	482(281-764)	243.94(146.05-378.93)	-0.35(-0.45 to -0.26)
Dominica	2(2-3)	15.04(10.98-20.03)	3(2-5)	14.03(9.45-20.05)	-0.25(-0.5 to 0)	40(29-53)	251.09(184.85-332.87)	55(37-78)	230.84(155.82-328.48)	-0.28(-0.58 to 0.03)
Dominican Republic	97(68-136)	10.26(7.19-14.32)	320(197-500)	11.81(7.26-18.44)	0.53(0.19 to 0.87)	1822(1282-2544)	182.5(128.27-254.58)	5247(3252-8304)	190.49(118.02-301.01)	0.25(-0.13 to 0.63)
Ecuador	52(44-62)	3.77(3.17-4.48)	214(157-284)	4.87(3.58-6.43)	1.21(1.01 to 1.4)	958(816-1131)	66.88(56.93-78.88)	3036(2257-4032)	68.14(50.75-90.29)	0.37(-0.03 to 0.78)
Egypt	102(75-137)	1.54(1.14-2.05)	519(373-710)	3.23(2.34-4.39)	2.37(1.88 to 2.85)	2018(1484-2699)	27.29(20.14-36.41)	8082(5787-10907)	45.74(33.08-61.38)	1.74(1.27 to 2.22)
El Salvador	43(34-52)	5.4(4.33-6.65)	105(78-139)	6.22(4.6-8.26)	0.49(-0.04 to 1.02)	783(630-953)	97.31(78.25-118.37)	1444(1076-1905)	86.42(64.45-114.12)	-0.35(-0.93 to 0.23)
Equatorial Guinea	4(2-6)	7.01(4.41-10.99)	13(8-20)	9.56(5.87-14.78)	1.02(0.69 to 1.35)	90(56-147)	156.8(97.48-251.48)	241(144-375)	163.52(99.32-252.06)	0.16(-0.17 to 0.48)

location	Incidence					DALYs				
	Num in 1990	ASR in 1990	Num in 2021	ASR in 2021	AAPC	Num in 1990	ASR in 1990	Num in 2021	ASR in 2021	AAPC
Eritrea	39(24-62)	11.65(7.36-17.79)	87(55-129)	11.18(7.16-16.31)	-0.14(-0.24 to -0.04)	1019(621-1615)	268.7(168.25-416.72)	2014(1265-3017)	235.54(150.19-347.97)	-0.44(-0.55 to -0.34)
Estonia	81(65-99)	14.31(11.53-17.54)	147(112-186)	21.79(16.66-27.64)	1.48(0.61 to 2.37)	1167(956-1409)	208.18(170.37-251.29)	1329(1039-1645)	208.73(162.6-259.23)	0.42(-0.19 to 1.03)
Eswatini	10(7-15)	12.84(8.48-18.96)	23(13-38)	14.31(8.45-23.51)	0.38(0.14 to 0.61)	232(152-348)	269.78(177.68-401.27)	494(285-829)	292.66(170.9-484.9)	0.27(0.08 to 0.47)
Ethiopia	471(302-752)	9.3(6.13-14.54)	936(714-1204)	8.52(6.5-10.95)	-0.27(-0.36 to -0.19)	11054(7022-17838)	200.86(130.37-319.24)	18070(13895-23036)	155.96(119.89-198.74)	-0.82(-0.91 to -0.74)
Fiji	9(6-12)	9.08(6.2-12.84)	19(12-27)	9.13(5.94-13.31)	-0.04(-0.36 to 0.27)	170(117-239)	162.32(111.13-228.54)	340(219-503)	155.62(101.2-227.71)	-0.17(-0.38 to 0.04)
Finland	189(159-223)	10.14(8.5-11.96)	373(304-449)	11.92(9.78-14.31)	0.59(0.06 to 1.13)	2169(1927-2431)	119.32(105.93-133.83)	2905(2507-3294)	98.01(85.5-110.85)	-0.67(-0.95 to -0.4)
France	7042(5609-8794)	34.46(27.41-43.15)	9072(7233-11195)	26.72(21.4-33)	-0.68(-1.2 to -0.15)	73819(61298-88571)	373.16(309.6-448.33)	53428(44068-63966)	164.35(136.37-196.85)	-2.57(-2.91 to -2.22)
Gabon	15(10-23)	9.76(6.27-14.69)	30(19-45)	10.55(6.88-15.53)	0.27(0.21 to 0.33)	315(202-479)	197.56(126.99-299.92)	580(372-865)	187.51(121.57-277.74)	-0.15(-0.31 to 0.01)
Gambia	4(3-6)	4.28(2.9-6.1)	15(9-22)	5.57(3.58-8.15)	0.83(-0.02 to 1.7)	85(57-123)	85.82(57.74-123.16)	292(185-431)	104.99(66.81-154.31)	0.65(-0.3 to 1.6)
Georgia	134(109-162)	7.59(6.18-9.14)	194(161-230)	12.01(10-14.28)	1.69(0.61 to 2.78)	2492(2051-2963)	139.33(114.78-165.65)	3165(2675-3679)	201.78(170.54-234.57)	1.56(0.41 to 2.73)

location	Incidence					DALYs				
	Num in 1990	ASR in 1990	Num in 2021	ASR in 2021	AAPC	Num in 1990	ASR in 1990	Num in 2021	ASR in 2021	AAPC
Germany	5782(4731-7083)	17.84(14.57-21.9)	8117(6486-9955)	16.76(13.42-20.57)	-0.22(-0.64 to 0.21)	72775(60855-85865)	228.93(191.07-270.43)	65807(54216-77689)	142.47(117.71-168.36)	-1.54(-1.89 to -1.18)
Ghana	25(17-36)	1.46(0.99-2.1)	70(44-105)	1.59(1.01-2.4)	0.25(0.07 to 0.42)	539(361-773)	29.41(19.83-41.98)	1318(837-1959)	27.69(17.63-41.17)	-0.18(-0.31 to -0.04)
Greece	392(330-461)	9.45(7.94-11.12)	752(620-892)	12.62(10.49-14.97)	0.98(0.58 to 1.38)	3749(3337-4176)	91.76(81.64-102.26)	6366(5580-7147)	114.14(101-127.75)	0.66(0.36 to 0.95)
Greenland	3(2-4)	34.56(26.69-43.95)	4(3-6)	20.93(15.27-27.98)	-1.52(-1.71 to -1.32)	50(40-63)	517.55(411.87-651)	56(43-74)	262.61(197.3-344.92)	-2.13(-2.49 to -1.76)
Grenada	3(2-3)	14.34(11.33-17.8)	4(3-5)	12.15(9.35-15.36)	-0.53(-1.78 to 0.73)	47(38-58)	264.06(210.66-326.91)	65(50-81)	193.32(149.8-241.78)	-1.01(-1.35 to -0.67)
Guam	1(1-1)	5.29(4-6.78)	5(4-6)	8.51(6.25-11.23)	1.52(0.73 to 2.32)	16(12-20)	74.57(57.33-95.17)	75(56-97)	130.64(97.38-170.83)	1.71(0.37 to 3.06)
Guatemala	45(40-51)	5.43(4.79-6.12)	124(101-150)	4.27(3.48-5.16)	-0.86(-1.65 to -0.07)	939(836-1049)	101.84(90.63-113.89)	2050(1689-2454)	68.7(56.71-82.07)	-1.34(-2.48 to -0.19)
Guinea	106(74-147)	11.81(8.19-16.36)	221(144-317)	14.32(9.43-20.45)	0.63(0.58 to 0.69)	2377(1643-3291)	256.67(177.36-355.56)	4835(3130-7000)	298.48(194.78-429.65)	0.49(0.41 to 0.58)
Guinea-Bissau	4(3-7)	4.17(2.62-6.49)	12(7-18)	6(3.81-9.03)	1.19(1.1 to 1.27)	108(66-173)	94(58.39-149.34)	272(170-421)	125.61(79.9-191.42)	0.96(0.89 to 1.03)
Guyana	7(6-9)	7.23(5.81-8.84)	12(8-16)	6.56(4.71-8.84)	-0.18(-0.66 to 0.31)	154(123-190)	146.06(116.78-179.55)	239(170-326)	125.08(89.76-169.66)	-0.39(-0.97 to 0.2)

location	Incidence					DALYs				
	Num in 1990	ASR in 1990	Num in 2021	ASR in 2021	AAPC	Num in 1990	ASR in 1990	Num in 2021	ASR in 2021	AAPC
Haiti	86(52-143)	10.02(6.2-16.42)	173(102-279)	9.15(5.47-14.57)	-0.25(-0.28 to -0.22)	2029(1223-3442)	220.89(134.78-369.38)	3888(2278-6214)	189.25(111.71-300.48)	-0.47(-0.5 to -0.43)
Honduras	21(15-29)	3.97(2.86-5.44)	96(61-142)	5.76(3.68-8.54)	1.23(0.9 to 1.56)	421(302-579)	75.46(54.43-103.7)	1690(1094-2498)	97.06(62.76-143.42)	0.84(0.57 to 1.11)
Hungary	1017(839-1221)	26.31(21.66-31.59)	1465(1188-1770)	30.36(24.61-36.74)	0.48(0.15 to 0.81)	18616(15574-22071)	490.67(410.04-582.12)	18816(15707-22172)	409.41(341.56-482.98)	-0.62(-0.92 to -0.32)
Iceland	11(9-13)	14.57(12.17-17.21)	23(19-28)	15.1(12.14-18.42)	0.08(-0.32 to 0.48)	101(89-114)	140.16(123.55-157.19)	163(138-189)	108.88(92.49-125.93)	-0.86(-1.08 to -0.64)
India	28558(24854-32461)	21.98(18.99-25.14)	94499(80205-108696)	28.74(24.38-33.05)	0.87(0.66 to 1.08)	642094(559154-730112)	462.93(401.12-528.58)	1697642(1430849-1961508)	498.71(420.51-576.04)	0.24(0.05 to 0.44)
Indonesia	1867(1392-2366)	7.25(5.4-9.18)	5777(4151-7590)	9.16(6.6-11.98)	0.77(0.69 to 0.84)	37407(27947-47244)	135.13(100.89-170.64)	100686(71840-133072)	147.62(105.81-194.23)	0.29(0.25 to 0.34)
Iran (Islamic Republic of)	120(98-147)	1.93(1.57-2.37)	585(478-707)	2.93(2.38-3.54)	1.29(0.98 to 1.59)	1998(1675-2387)	29.02(24.21-34.77)	6764(5756-7829)	32.37(27.45-37.52)	0.33(0.15 to 0.5)
Iraq	97(64-141)	4.55(3.01-6.59)	362(233-523)	5.5(3.55-7.9)	0.64(0.43 to 0.85)	1791(1190-2547)	81.39(54-115.78)	5243(3357-7516)	74.21(47.77-105.98)	-0.29(-0.36 to -0.22)
Ireland	193(155-238)	17.75(14.26-21.96)	306(239-383)	14.52(11.38-18.19)	-0.72(-1.4 to -0.05)	2093(1737-2494)	197.99(164.21-236.37)	2317(1895-2774)	112.56(92.15-134.83)	-1.91(-2.54 to -1.28)
Israel	87(71-105)	6.83(5.57-8.27)	244(192-303)	7.48(5.88-9.29)	0.52(-0.46 to 1.52)	1029(864-1208)	82.75(69.39-97.28)	2120(1728-2528)	66.8(54.7-79.6)	-0.62(-1.31 to 0.08)

location	Incidence					DALYs				
	Num in 1990	ASR in 1990	Num in 2021	ASR in 2021	AAPC	Num in 1990	ASR in 1990	Num in 2021	ASR in 2021	AAPC
Italy	4015(3473-4605)	17.03(14.72-19.55)	5401(4488-6307)	14.39(12.13-16.78)	-0.55(-0.76 to -0.33)	47830(44545-51040)	207.45(193.19-221.42)	44416(39680-48121)	124.21(112.99-133.8)	-1.72(-2.1 to -1.34)
Jamaica	34(28-40)	7.04(5.76-8.46)	57(41-78)	6.72(4.83-9.23)	0.05(-0.35 to 0.44)	513(426-610)	110.05(91.25-130.99)	816(584-1117)	96.21(68.94-131.53)	-0.28(-0.73 to 0.17)
Japan	4648(4179-5134)	9.93(8.9-10.97)	14465(11501-17218)	14.54(11.9-17.18)	1.17(0.81 to 1.53)	37561(35556-39108)	79.94(75.45-83.33)	83066(71717-90342)	90.03(81.46-96.06)	0.31(-0.11 to 0.73)
Jordan	20(13-28)	5.47(3.77-7.75)	115(74-172)	5.71(3.72-8.45)	0.17(-0.22 to 0.56)	344(236-487)	88.39(60.93-124.76)	1495(958-2249)	68.11(44.07-101.39)	-0.81(-1.07 to -0.55)
Kazakhstan	436(372-508)	12.28(10.47-14.29)	545(442-666)	10.79(8.77-13.17)	-0.4(-1.1 to 0.3)	8872(7656-10203)	240.8(207.66-276.9)	9019(7412-10829)	170.72(140.41-204.68)	-1.04(-1.92 to -0.15)
Kenya	259(174-343)	11.64(7.87-15.35)	1004(756-1293)	15.89(12.08-20.33)	0.98(0.82 to 1.14)	5430(3595-7279)	232.1(154.57-309.66)	20573(15322-26647)	303.21(227.79-390.38)	0.87(0.78 to 0.96)
Kiribati	2(1-2)	16.31(10.24-24.5)	4(2-6)	18.22(10.87-28.71)	0.36(0.33 to 0.4)	41(26-62)	379.19(236.96-574)	90(53-145)	406.88(241.48-652.04)	0.23(0.2 to 0.26)
Kuwait	18(15-23)	10.88(8.69-13.54)	33(24-43)	4.25(3.17-5.55)	-2.79(-5.27 to -0.24)	253(207-308)	137.79(113.13-167.32)	330(253-428)	38.82(29.7-50.23)	-3.85(-6.29 to -1.35)
Kyrgyzstan	133(104-166)	16.11(12.65-20.15)	109(81-142)	8.11(6.08-10.5)	-2.47(-3.19 to -1.73)	2845(2221-3564)	338.14(264.58-422.4)	1970(1485-2551)	136.93(103.96-176.61)	-3.07(-3.82 to -2.32)
Lao People's Democratic Republic	47(29-74)	8.65(5.41-13.39)	107(70-156)	8.8(5.8-12.78)	0.06(0 to 0.12)	1067(652-1699)	183.26(113.29-288.79)	2064(1336-3021)	158.99(104.07-231.27)	-0.45(-0.51 to -0.39)

location	Incidence					DALYs				
	Num in 1990	ASR in 1990	Num in 2021	ASR in 2021	AAPC	Num in 1990	ASR in 1990	Num in 2021	ASR in 2021	AAPC
Latvia	122(99-148)	12.3(10.02-14.94)	185(145-230)	18.56(14.51-23.18)	1.45(0.63 to 2.27)	2437(1970-2967)	247.55(200.09-301.37)	2651(2090-3330)	285.95(224.99-360.31)	0.69(0.01 to 1.37)
Lebanon	39(25-61)	6.53(4.19-10.24)	118(79-169)	7.15(4.79-10.31)	0.38(0.07 to 0.68)	633(400-1011)	101.68(64.96-160.6)	1240(840-1779)	77.16(52.08-111)	-0.83(-1.03 to -0.63)
Lesotho	23(15-34)	9.96(6.49-14.59)	44(26-72)	14.78(8.82-23.61)	1.32(1.08 to 1.56)	503(327-738)	210.44(137.1-308.56)	1017(589-1667)	324.28(189.7-527.23)	1.46(1.27 to 1.64)
Liberia	10(6-15)	3.35(2.12-4.99)	28(17-45)	4.74(2.81-7.46)	1.13(0.95 to 1.32)	225(139-340)	71.52(44.55-107.94)	595(343-939)	90.12(52.94-141.11)	0.75(0.56 to 0.94)
Libya	32(20-48)	6.27(3.93-9.29)	109(70-165)	7.3(4.75-10.91)	0.52(0.23 to 0.8)	551(344-822)	102.5(64.44-152.33)	1634(1068-2471)	101.94(67.47-152.25)	-0.04(-0.16 to 0.09)
Lithuania	171(139-208)	13.68(11.15-16.68)	327(257-411)	23.03(18.02-29)	1.89(1.4 to 2.38)	2752(2266-3307)	220.75(181.85-265.34)	4146(3278-5112)	307.58(242.56-379.65)	1.22(0.61 to 1.83)
Luxembourg	30(25-36)	20.74(17.29-24.83)	46(37-56)	16.06(12.99-19.61)	-0.91(-1.56 to -0.27)	354(311-403)	251.28(220.41-286.23)	375(319-434)	133.68(113.57-154.55)	-2(-2.54 to -1.46)
Madagascar	157(108-221)	11.2(7.75-15.67)	275(180-409)	8.55(5.68-12.53)	-0.86(-1 to -0.72)	3616(2488-5095)	245.37(169.45-344.79)	6231(4026-9249)	176.39(115.35-258.83)	-1.08(-1.26 to -0.9)
Malawi	80(55-112)	7.68(5.28-10.69)	183(116-268)	9.01(5.84-13.08)	0.54(0.36 to 0.73)	1842(1249-2579)	166.06(113.3-231.96)	3944(2504-5823)	182.12(117.41-266.03)	0.32(0.21 to 0.44)
Malaysia	375(270-509)	15.3(10.97-20.77)	1316(930-1807)	17.16(12.16-23.48)	0.28(0.08 to 0.49)	6486(4691-8717)	254.14(183.7-341.63)	17969(12867-24543)	227.77(163.43-310.19)	-0.44(-0.63 to -0.24)

location	Incidence					DALYs				
	Num in 1990	ASR in 1990	Num in 2021	ASR in 2021	AAPC	Num in 1990	ASR in 1990	Num in 2021	ASR in 2021	AAPC
Maldives	3(2-5)	13.21(8.29-20.1)	10(7-14)	11.87(8.06-16.68)	-0.41(-0.83 to 0.01)	55(33-90)	227.99(140.52-358.53)	122(80-175)	137.55(92.56-194.41)	-1.75(-2.03 to -1.46)
Mali	59(42-82)	5.36(3.79-7.4)	142(90-210)	5.79(3.72-8.5)	0.26(0.18 to 0.33)	1362(958-1891)	115.58(81.45-160.16)	3055(1916-4606)	116.71(74.09-174.36)	0.04(-0.03 to 0.1)
Malta	14(11-18)	12.22(9.77-15.18)	31(24-39)	12.85(10-16.12)	0(-0.11 to 0.11)	164(136-196)	142.1(117.75-170.08)	251(205-302)	110.74(90.48-132.9)	-0.87(-1.06 to -0.68)
Marshall Islands	0(0-0)	5.88(3.93-8.76)	1(0-1)	7.75(4.83-11.81)	0.89(0.81 to 0.97)	5(3-8)	118.78(77.21-182.04)	16(9-25)	150.05(92.02-231.37)	0.76(0.66 to 0.85)
Mauritania	11(8-16)	4.13(2.82-5.88)	34(21-51)	5.76(3.63-8.64)	1.08(0.95 to 1.22)	235(160-331)	84.67(57.85-119.39)	609(378-916)	98.83(62.1-147.79)	0.5(0.37 to 0.63)
Mauritius	30(26-34)	15.64(13.41-18)	74(62-87)	14.48(12.09-17.03)	-0.25(-1.8 to 1.32)	485(425-548)	244.79(214.26-276.6)	1063(910-1222)	205.17(175.58-235.66)	-0.52(-1.98 to 0.96)
Mexico	561(537-584)	5.24(4.99-5.46)	1701(1487-1923)	4.96(4.34-5.6)	-0.14(-0.51 to 0.22)	9770(9406-10121)	86.4(82.93-89.62)	24709(21588-28078)	70.17(61.36-79.63)	-0.64(-1.08 to -0.2)
Micronesia (Federated States of)	1(1-1)	6.64(4.47-9.79)	2(1-3)	8.3(5.41-12.22)	0.72(0.67 to 0.77)	18(12-27)	135.19(89.65-203.68)	34(22-52)	154.32(99.76-230.75)	0.43(0.39 to 0.46)
Monaco	1(1-2)	6.99(4.6-10.05)	2(1-3)	8.41(5.7-12.17)	0.6(0.5 to 0.69)	11(7-15)	68.42(46.91-95.95)	16(11-22)	69.12(47.53-98.72)	0.02(-0.06 to 0.1)
Mongolia	32(22-46)	11.2(7.59-16)	51(34-72)	7.93(5.33-11.14)	-1.13(-1.41 to -0.86)	725(493-1038)	245.6(167.28-351.11)	1037(704-1437)	146.05(99.14-202.62)	-1.73(-2.07 to -1.39)

location	Incidence					DALYs				
	Num in 1990	ASR in 1990	Num in 2021	ASR in 2021	AAPC	Num in 1990	ASR in 1990	Num in 2021	ASR in 2021	AAPC
Montenegro	23(17-29)	12.71(9.62-16.59)	41(30-54)	15.37(11.1-20.6)	0.75(0.44 to 1.05)	352(270-450)	194.1(148.92-248.84)	528(385-700)	202.36(146.84-269.25)	0.3(-0.23 to 0.82)
Morocco	119(82-165)	3.1(2.14-4.31)	384(254-542)	4.02(2.67-5.64)	0.84(0.76 to 0.92)	2310(1603-3184)	58.31(40.47-80.42)	6200(4143-8825)	62.51(41.95-88.59)	0.23(0.16 to 0.3)
Mozambique	94(62-137)	5.92(3.96-8.55)	221(138-338)	7.36(4.69-11.05)	0.74(0.67 to 0.81)	2186(1410-3193)	127.13(83.05-184.54)	5061(3154-7847)	154.61(97.64-236.16)	0.65(0.57 to 0.72)
Myanmar	503(308-804)	8.29(5.16-13)	1092(705-1607)	8.35(5.42-12.22)	0.03(-0.02 to 0.08)	10798(6517-17841)	167.5(102.56-272.23)	19443(12510-28867)	141.45(91.52-209.03)	-0.56(-0.6 to -0.52)
Namibia	37(25-53)	20.6(13.87-29.54)	102(64-150)	25.79(16.45-37.66)	0.75(0.59 to 0.9)	821(549-1187)	432.6(290.33-623.85)	2046(1279-3032)	489.58(309.45-720.77)	0.45(0.33 to 0.56)
Nauru	0(0-0)	7.94(4.72-13.06)	0(0-0)	9.61(5.37-16.13)	0.62(0.59 to 0.64)	2(1-4)	155.29(90.94-262.18)	3(2-5)	175.59(98.51-301.54)	0.39(0.32 to 0.45)
Nepal	515(322-783)	20.16(12.55-30.38)	1406(900-2089)	22.18(14.32-32.77)	0.31(0.22 to 0.4)	11847(7327-18065)	431.18(266.67-652.07)	26163(16541-39508)	396.85(252.89-595.93)	-0.26(-0.34 to -0.18)
Netherlands	677(564-811)	12.88(10.71-15.44)	1205(967-1462)	12.68(10.2-15.38)	-0.08(-0.43 to 0.26)	6976(5971-8159)	136.2(116.52-159.49)	9428(7888-11019)	102.63(86.1-119.98)	-0.93(-1.38 to -0.48)
New Zealand	197(158-242)	18.6(14.9-22.78)	395(312-491)	17.21(13.63-21.37)	-0.32(-1.43 to 0.81)	1569(1328-1840)	150.43(127.22-176.37)	2288(1898-2701)	102.22(84.96-120.59)	-1.3(-2.36 to -0.23)
Nicaragua	12(8-16)	2.92(2.06-3.97)	41(27-58)	3.13(2.1-4.42)	0.11(-0.28 to 0.51)	208(148-281)	50.3(35.79-68.08)	614(413-857)	45.71(30.75-63.77)	-0.37(-0.86 to 0.12)

location	Incidence					DALYs				
	Num in 1990	ASR in 1990	Num in 2021	ASR in 2021	AAPC	Num in 1990	ASR in 1990	Num in 2021	ASR in 2021	AAPC
Niger	22(14-32)	2.91(1.91-4.28)	86(51-135)	3.9(2.36-6.11)	0.99(0.77 to 1.2)	510(335-743)	62.83(41.29-91.61)	1891(1116-2983)	78.88(47.13-124.11)	0.78(0.61 to 0.95)
Nigeria	294(220-372)	2.49(1.87-3.14)	822(586-1121)	3.28(2.4-4.38)	0.87(0.81 to 0.93)	6330(4743-8086)	50.74(38.09-64.56)	16337(11685-22227)	60.2(43.95-80.54)	0.54(0.48 to 0.6)
Niue	0(0-0)	6.88(4.67-9.69)	0(0-0)	8.82(5.89-12.61)	0.8(0.72 to 0.89)	1(0-1)	118.04(79.35-167.36)	1(1-1)	136.28(90.05-198.08)	0.45(0.36 to 0.55)
North Macedonia	43(33-55)	8.23(6.34-10.53)	93(66-126)	10.3(7.38-13.98)	0.73(0.41 to 1.05)	823(642-1045)	151.57(117.95-192.87)	1338(965-1823)	146.48(105.86-199.41)	-0.09(-0.37 to 0.18)
Northern Mariana Islands	1(1-1)	16.97(11.46-24.71)	6(4-8)	41.07(28.92-55.85)	2.81(2.54 to 3.08)	14(9-22)	256.7(170.34-380.11)	91(64-126)	572.17(405.66-782.4)	2.59(2.38 to 2.79)
Norway	230(199-264)	13.13(11.36-15.09)	330(275-386)	12.13(10.18-14.2)	-0.12(-1.1 to 0.87)	2263(2120-2392)	136.12(128-143.83)	2330(2111-2494)	88.4(80.9-94.3)	-1.3(-2.23 to -0.35)
Oman	11(7-17)	6.09(3.77-9.29)	35(21-54)	6.24(3.87-9.49)	-0.01(-0.59 to 0.57)	199(119-309)	102.45(62.21-157.23)	466(277-712)	74.77(45.95-112.5)	-1(-1.4 to -0.59)
Pakistan	7971(6061-10333)	52.06(39.53-67.6)	21174(15571-28516)	62.59(46.14-84.03)	0.6(0.53 to 0.66)	171437(130500-221313)	1076.37(818.08-1390.92)	436960(322502-589310)	1204.9(892.71-1620.52)	0.37(0.29 to 0.46)
Palau	2(1-3)	79.35(53.79-115.09)	5(3-7)	76.27(51.4-109.87)	-0.16(-0.22 to -0.1)	34(23-49)	1300.16(881.2-1868.51)	80(52-118)	1162.53(775.81-1698.43)	-0.37(-0.46 to -0.28)
Palestine	6(4-9)	2.72(1.82-3.93)	19(13-27)	3.01(2.12-4.21)	0.29(0.02 to 0.56)	97(64-143)	42.21(28.16-61.73)	266(187-377)	38.37(27.19-54.04)	-0.34(-0.52 to -0.16)

location	Incidence					DALYs				
	Num in 1990	ASR in 1990	Num in 2021	ASR in 2021	AAPC	Num in 1990	ASR in 1990	Num in 2021	ASR in 2021	AAPC
Panama	32(27-37)	8.11(6.95-9.36)	89(67-113)	7.37(5.51-9.35)	-0.28(-0.55 to 0)	491(431-553)	122.72(107.72-138.44)	1116(838-1388)	92.05(69.11-114.46)	-0.78(-0.98 to -0.59)
Papua New Guinea	22(12-35)	4.54(2.5-7.05)	80(44-124)	5.73(3.26-8.76)	0.74(0.61 to 0.87)	481(248-772)	90.72(48.12-143.98)	1738(940-2732)	112.34(62.41-174.03)	0.7(0.58 to 0.82)
Paraguay	39(27-54)	6.46(4.5-8.98)	151(95-230)	9.47(5.99-14.31)	1.21(0.95 to 1.48)	707(489-977)	115.16(79.78-158.97)	2457(1547-3709)	150.02(94.73-225.91)	0.85(0.67 to 1.02)
Peru	136(100-182)	4.31(3.17-5.77)	462(308-665)	5.06(3.38-7.28)	0.58(-0.3 to 1.46)	2520(1867-3349)	77.04(57.09-102.4)	5931(3991-8490)	64.3(43.29-91.95)	-0.56(-1.32 to 0.2)
Philippines	728(563-878)	9.57(7.42-11.53)	1960(1614-2340)	8.88(7.34-10.56)	-0.24(-0.33 to -0.16)	13619(10623-16196)	166.74(130.28-198.21)	34521(28601-41116)	147.95(123.21-175.59)	-0.38(-0.48 to -0.27)
Poland	1282(1193-1374)	11.08(10.29-11.89)	3220(2801-3650)	17.26(15.04-19.57)	1.53(1.43 to 1.62)	26981(25745-28271)	234.93(223.88-246.32)	49530(44575-54331)	279.48(251.69-306.73)	0.59(0.49 to 0.69)
Portugal	662(539-811)	17.77(14.45-21.78)	1388(1085-1751)	23.24(18.2-29.42)	0.99(0.18 to 1.8)	8491(7058-10136)	232.64(193.18-277.83)	10799(8780-13099)	194.83(158.64-236.88)	-0.49(-1.4 to 0.44)
Puerto Rico	169(137-206)	17.18(13.93-20.93)	180(135-233)	9.67(7.19-12.6)	-1.99(-2.43 to -1.55)	2349(1927-2824)	240.73(197.35-289.5)	1870(1428-2373)	106.88(81.24-136.26)	-2.74(-3.2 to -2.28)
Qatar	1(1-2)	5.01(3.27-7.29)	16(9-26)	7.85(4.73-11.98)	1.35(0.75 to 1.95)	20(13-30)	68.76(44.43-101.2)	181(99-290)	70.67(41.77-108.2)	-0.19(-1.04 to 0.68)
Republic of Korea	493(387-627)	6.15(4.81-7.81)	2448(1703-3316)	9.4(6.53-12.74)	1.38(0.76 to 1.99)	7057(5603-8910)	82.48(65.46-103.95)	16051(11300-21219)	61.82(43.5-81.78)	-0.94(-1.12 to -0.76)

location	Incidence					DALYs				
	Num in 1990	ASR in 1990	Num in 2021	ASR in 2021	AAPC	Num in 1990	ASR in 1990	Num in 2021	ASR in 2021	AAPC
Republic of Moldova	177(153-203)	14.18(12.25-16.27)	260(216-311)	15.99(13.27-19.18)	0.34(-0.38 to 1.07)	3639(3192-4142)	285.18(250.08-324.51)	3991(3378-4673)	249.25(210.96-291.85)	-0.51(-1.27 to 0.25)
Romania	824(682-988)	10.6(8.78-12.72)	2034(1594-2544)	22.02(17.24-27.59)	2.48(1.85 to 3.11)	16698(13891-19828)	212.97(177.19-252.71)	30171(24172-37186)	340.87(273.04-420.56)	1.55(0.95 to 2.15)
Russian Federation	7541(7230-7847)	15.06(14.41-15.68)	12600(11297-13799)	19.89(17.83-21.78)	1.26(0.12 to 2.41)	125300(120418-130224)	248.67(238.66-258.63)	153581(137909-168229)	244.26(219.39-267.43)	0.23(-0.91 to 1.39)
Rwanda	113(76-162)	14.48(9.83-20.67)	210(132-322)	12.16(7.72-18.4)	-0.55(-0.66 to -0.43)	2738(1825-3931)	331.59(222.35-474.29)	4426(2718-6828)	238.76(148.75-364.23)	-1.03(-1.2 to -0.86)
Saint Kitts and Nevis	1(1-1)	11.88(10.28-13.56)	2(2-3)	10.9(8.46-13.62)	-0.3(-1.18 to 0.58)	21(18-24)	221.42(194.07-252.06)	34(27-43)	167(130.7-206.04)	-0.77(-1.45 to -0.08)
Saint Lucia	4(3-5)	17.24(14.94-19.82)	9(7-12)	13.77(10.58-17.48)	-0.63(-0.82 to -0.45)	69(60-79)	299.24(260.51-340.24)	144(112-182)	211.78(164.46-267.29)	-1.1(-1.51 to -0.68)
Saint Vincent and the Grenadines	4(3-4)	19.63(16.81-22.83)	7(6-9)	18.12(14.81-21.95)	-0.39(-1.19 to 0.42)	64(56-74)	340.6(295.17-391.09)	121(101-145)	301.33(251.29-360.74)	-0.48(-1.27 to 0.31)
Samoa	1(1-2)	6.31(4.2-9.29)	3(2-4)	6.65(4.28-9.96)	0.16(0.1 to 0.21)	30(19-45)	122.27(79.9-183.19)	50(31-76)	118.27(74.86-178.68)	-0.11(-0.15 to -0.07)
San Marino	1(1-2)	15.95(10.67-22.95)	2(1-3)	9.61(5.23-15.71)	-1.91(-2.33 to -1.49)	13(9-19)	147.17(100.82-209.49)	14(8-23)	78.09(42.68-128.53)	-2.22(-2.63 to -1.8)
Sao Tome and Principe	0(0-0)	0.41(0.27-0.59)	0(0-0)	0.54(0.34-0.83)	0.87(0.74 to 1)	1(1-2)	7.41(4.98-10.54)	3(2-4)	8.63(5.47-13.1)	0.49(0.37 to 0.61)

location	Incidence					DALYs				
	Num in 1990	ASR in 1990	Num in 2021	ASR in 2021	AAPC	Num in 1990	ASR in 1990	Num in 2021	ASR in 2021	AAPC
Saudi Arabia	99(64-143)	6.44(4.24-9.25)	416(281-606)	7.54(5.21-10.68)	0.49(0.39 to 0.59)	1856(1192-2756)	113.02(73.68-165.58)	5683(3856-8262)	91.29(63.51-128.9)	-0.7(-0.83 to -0.58)
Senegal	31(20-45)	3.51(2.31-5.13)	113(70-172)	5.36(3.35-8.12)	1.29(1.15 to 1.43)	665(433-970)	72.81(47.63-106.04)	2272(1398-3509)	101.46(62.94-155.58)	1.04(0.88 to 1.19)
Serbia	424(290-598)	13.62(9.33-19.17)	692(468-972)	16.11(10.83-22.71)	0.61(0.19 to 1.04)	7730(5272-10806)	235.48(160.95-328.96)	8526(5813-11722)	207.57(140.85-286.62)	-0.33(-0.7 to 0.06)
Seychelles	4(3-6)	26.03(17.56-37.39)	11(7-16)	33.72(22.37-48.68)	0.76(0.53 to 0.98)	71(48-103)	472.78(314.73-681.76)	173(114-252)	510.27(337.68-735.16)	0.2(-0.08 to 0.48)
Sierra Leone	17(11-26)	3.21(2.02-4.73)	48(31-71)	4.66(3.02-6.89)	1.21(1.08 to 1.35)	376(234-558)	67.49(42.12-99.94)	1003(636-1497)	91.96(59.15-136.81)	1(0.86 to 1.14)
Singapore	57(46-70)	9.59(7.75-11.67)	240(188-300)	10.19(7.98-12.72)	0.43(-0.48 to 1.35)	665(554-793)	107.16(89.19-127.55)	1614(1330-1934)	67.73(55.73-81.13)	-1.32(-2.37 to -0.26)
Slovakia	462(351-600)	29.05(22.05-37.76)	623(413-895)	24.86(16.48-35.73)	-0.45(-0.61 to -0.29)	7944(6049-10391)	510.19(388.03-668.04)	8557(5593-12223)	354.16(231.09-506.58)	-1.18(-1.39 to -0.97)
Slovenia	128(102-157)	18.79(15.02-23.12)	182(138-234)	16.18(12.18-20.9)	-0.54(-0.66 to -0.42)	1725(1435-2052)	254.38(211.69-302.78)	1494(1187-1857)	137.78(109.35-171.92)	-2.19(-2.31 to -2.06)
Solomon Islands	2(1-3)	5.33(2.78-8.35)	7(4-11)	7.03(4.38-10.72)	0.88(0.74 to 1.03)	44(22-73)	111.13(56.09-178.73)	149(91-233)	140.86(86.47-216.79)	0.77(0.62 to 0.91)
Somalia	70(43-108)	10.42(6.56-15.82)	141(87-216)	8.54(5.4-12.89)	-0.63(-0.74 to -0.52)	1783(1085-2776)	238.85(148.88-366.35)	3458(2141-5379)	192.01(121.14-293.35)	-0.69(-0.78 to -0.6)

location	Incidence					DALYs				
	Num in 1990	ASR in 1990	Num in 2021	ASR in 2021	AAPC	Num in 1990	ASR in 1990	Num in 2021	ASR in 2021	AAPC
South Africa	767(537-983)	13.52(9.43-17.35)	1744(1491-2012)	13.41(11.47-15.47)	0(-0.14 to 0.13)	15524(10921-19835)	264.01(184.95-337.92)	32311(27482-37281)	239.16(203.66-275.64)	-0.33(-0.77 to 0.11)
South Sudan	77(48-119)	11.07(6.97-17.1)	108(68-164)	9.98(6.32-15.01)	-0.33(-0.44 to -0.22)	1723(1063-2713)	241.07(149.55-378.43)	2414(1493-3702)	203.86(127.15-310.38)	-0.53(-0.64 to -0.43)
Spain	4229(3346-5287)	29.46(23.26-36.89)	6320(4845-8073)	25.08(19.27-32.14)	-0.59(-0.94 to -0.24)	33450(27736-40016)	240.89(199.21-288.4)	34504(28086-41780)	142.92(116.86-173.36)	-1.73(-2.12 to -1.34)
Sri Lanka	608(441-825)	22.48(16.35-30.39)	2125(1209-3318)	28.41(16.32-44.16)	0.82(0.49 to 1.15)	10209(7319-13927)	356.6(256.64-484.33)	27209(15400-43085)	359.62(204.69-567.71)	0.08(-0.37 to 0.53)
Sudan	61(40-93)	2.51(1.65-3.8)	133(86-200)	2.6(1.71-3.88)	0.11(0.04 to 0.19)	1251(822-1916)	49.06(32.18-74.9)	2310(1489-3471)	42.15(27.53-62.88)	-0.49(-0.52 to -0.46)
Suriname	5(3-6)	6.66(4.98-8.78)	10(7-15)	5.8(3.82-8.61)	-0.37(-0.74 to -0.01)	89(65-118)	123.12(91.16-163.03)	186(122-280)	101.94(66.84-152.68)	-0.54(-0.85 to -0.23)
Sweden	448(385-514)	11.38(9.82-13.06)	713(585-849)	12.44(10.27-14.8)	0.59(0.1 to 1.08)	4087(3674-4488)	109.65(98.82-120.31)	5045(4269-5815)	92.67(79.02-106.83)	-0.36(-0.82 to 0.1)
Switzerland	320(260-390)	12.03(9.77-14.67)	571(443-715)	12.18(9.49-15.26)	0.04(-0.82 to 0.91)	3498(2937-4115)	135.3(113.41-159.3)	4557(3693-5524)	100.15(81.73-121.49)	-1.08(-1.96 to -0.21)
Syrian Arab Republic	31(21-42)	2.31(1.6-3.15)	97(66-137)	2.81(1.94-3.88)	0.6(0.27 to 0.93)	552(380-758)	38.39(26.53-52.51)	1295(897-1826)	34.58(24.34-48.09)	-0.4(-0.65 to -0.14)
Taiwan (Province of China)	778(640-937)	17.52(14.44-21.06)	5699(4394-7285)	50.62(39.05-64.71)	3.52(3.22 to 3.82)	10498(8819-12410)	231.57(194.57-273.41)	60005(48231-73092)	538.36(433.52-654.61)	2.71(2.36 to 3.07)

location	Incidence					DALYs				
	Num in 1990	ASR in 1990	Num in 2021	ASR in 2021	AAPC	Num in 1990	ASR in 1990	Num in 2021	ASR in 2021	AAPC
Tajikistan	30(18-44)	4.11(2.47-6.19)	44(30-62)	2.81(1.91-3.96)	-1.25(-1.54 to -0.96)	598(371-883)	79.22(48.46-117.66)	895(608-1264)	52.24(35.52-73.58)	-1.36(-1.63 to -1.09)
Thailand	1819(1328-2411)	19.57(14.31-25.9)	6067(4114-8480)	20.02(13.59-27.96)	0.07(-0.08 to 0.21)	30148(22020-39870)	303.87(222.5-401.37)	72747(49250-102007)	238.59(161.75-334.04)	-0.77(-0.95 to -0.59)
Timor-Leste	5(3-7)	6.68(4.25-9.84)	18(11-26)	7.73(5.03-11.44)	0.49(0.36 to 0.61)	104(64-156)	131.43(82.38-195.09)	324(209-482)	137.45(89.14-203.98)	0.14(0.03 to 0.24)
Togo	13(9-19)	3.82(2.59-5.5)	60(38-92)	5.69(3.57-8.65)	1.29(1.2 to 1.37)	284(190-418)	78.64(53.06-115.3)	1276(793-1944)	108.88(68.4-164.76)	1.06(0.96 to 1.17)
Tokelau	0(0-0)	6.73(4.42-9.77)	0(0-0)	8.43(5.45-12.34)	0.73(0.69 to 0.78)	0(0-1)	124.6(80.36-181.51)	1(0-1)	135.39(86.76-199.28)	0.27(0.22 to 0.33)
Tonga	1(1-1)	5.84(3.57-8.74)	2(1-3)	7.64(4.85-11.45)	0.84(0.65 to 1.03)	16(10-24)	100.92(61.2-153.4)	28(17-42)	124.01(77.23-185.48)	0.67(0.54 to 0.8)
Trinidad and Tobago	21(18-24)	9.25(8.04-10.62)	38(28-51)	7.18(5.25-9.47)	-0.62(-1.01 to -0.24)	362(318-409)	159.78(140.48-180.54)	601(443-794)	112.17(82.58-148.19)	-1.01(-1.43 to -0.58)
Tunisia	99(67-142)	7.41(5.02-10.58)	316(195-484)	8.67(5.35-13.19)	0.51(0.41 to 0.61)	1628(1089-2333)	115.97(77.82-165.78)	3930(2444-5945)	105.1(65.5-158.53)	-0.33(-0.4 to -0.26)
Turkey	395(264-566)	4.33(2.91-6.2)	1167(794-1630)	4.61(3.15-6.42)	0.18(-0.2 to 0.57)	7217(4799-10267)	74.55(49.75-105.89)	13782(9458-19074)	53.15(36.62-73.33)	-1.14(-1.34 to -0.94)
Turkmenistan	60(52-68)	11.39(9.81-13.04)	125(93-167)	10.96(8.21-14.53)	-0.08(-0.87 to 0.72)	1280(1110-1447)	232.6(201.53-262.97)	2432(1830-3276)	199.94(151.28-267.51)	-0.45(-1.29 to 0.4)

location	Incidence					DALYs				
	Num in 1990	ASR in 1990	Num in 2021	ASR in 2021	AAPC	Num in 1990	ASR in 1990	Num in 2021	ASR in 2021	AAPC
Tuvalu	0(0-0)	5.77(3.89-8.44)	0(0-0)	7.74(5.29-11.11)	0.95(0.93 to 0.97)	2(1-3)	116.14(76.73-172.07)	4(3-6)	139.18(93.52-202.3)	0.59(0.55 to 0.62)
Uganda	243(160-352)	13.76(9.14-19.84)	626(400-935)	15.4(9.95-22.79)	0.34(0.26 to 0.43)	5457(3596-7997)	293.61(194.1-428.13)	13309(8368-20163)	303.57(193.47-455.69)	0.09(0.02 to 0.16)
Ukraine	2879(2275-3730)	14.41(11.38-18.7)	4251(2808-6039)	20.8(13.67-29.66)	1.34(0.66 to 2.03)	42771(33802-55791)	214.81(169.82-281.12)	54511(35842-78487)	276.58(180.91-399.49)	0.97(0.25 to 1.7)
United Arab Emirates	10(6-18)	9.67(5.89-15.56)	84(54-124)	9.86(6.66-14.19)	-0.09(-1.11 to 0.94)	202(117-345)	160.33(96.76-261.3)	1361(872-2031)	119.89(80.65-173.73)	-1.05(-1.94 to -0.15)
United Kingdom	3266(3070-3438)	13.93(13.12-14.66)	6613(6036-7066)	19.81(18.24-21.11)	1.15(0.89 to 1.41)	30470(29170-31647)	134.71(129.17-139.85)	45010(42054-47335)	138.91(130.84-145.65)	0.11(-0.13 to 0.35)
United Republic of Tanzania	393(254-577)	13.11(8.54-19.09)	789(479-1211)	11.19(6.94-16.98)	-0.51(-0.57 to -0.44)	8768(5539-13067)	277.85(177.09-411.63)	16438(9830-25635)	219.34(133.54-338.04)	-0.77(-0.82 to -0.71)
United States of America	19991(18738-20930)	23.94(22.51-25.04)	31481(28821-33405)	19.84(18.25-21.02)	-0.66(-0.81 to -0.51)	143145(136129-148617)	176.83(168.64-183.36)	176325(164052-185667)	113.59(106.17-119.42)	-1.48(-1.64 to -1.32)
United States Virgin Islands	2(2-3)	9.82(6.58-14.12)	3(2-5)	5.86(3.58-9.21)	-1.71(-2.34 to -1.07)	39(26-56)	157.29(104.77-224.31)	39(24-62)	83.78(51.26-132.73)	-2.07(-2.62 to -1.52)
Uruguay	163(132-200)	15.47(12.54-18.98)	227(181-279)	15.66(12.47-19.28)	0.1(-0.09 to 0.3)	2348(1948-2819)	226.6(187.8-272.45)	2616(2159-3121)	187.88(155.17-224.5)	-0.54(-0.73 to -0.36)
Uzbekistan	189(137-246)	6.04(4.34-7.91)	553(423-711)	7.51(5.75-9.63)	0.69(0.2 to 1.17)	3738(2816-4783)	115.94(86.52-148.94)	10615(8206-13681)	134.25(104.09-172.53)	0.48(-0.02 to 0.98)

location	Incidence					DALYs				
	Num in 1990	ASR in 1990	Num in 2021	ASR in 2021	AAPC	Num in 1990	ASR in 1990	Num in 2021	ASR in 2021	AAPC
Vanuatu	1(1-1)	5.22(3.35-7.88)	3(2-4)	6.36(4.3-9.32)	0.64(0.46 to 0.82)	18(11-29)	103.18(64.65-159.33)	63(42-95)	123.49(82.55-183.12)	0.55(0.37 to 0.74)
Venezuela (Bolivarian Republic of)	183(161-207)	7.31(6.41-8.27)	652(477-862)	7.99(5.87-10.53)	0.24(-0.4 to 0.9)	3191(2856-3542)	122.39(109.31-135.92)	9650(7106-12667)	115.07(84.96-150.67)	-0.18(-0.52 to 0.16)
Viet Nam	1629(1129-2277)	15.01(10.43-20.94)	5442(3574-7953)	19.34(12.83-28.04)	0.84(0.77 to 0.91)	29320(20071-41011)	265.11(182.06-370.32)	78127(51176-114946)	267.6(177.02-390.63)	0.04(-0.02 to 0.11)
Yemen	36(21-59)	2.79(1.63-4.49)	103(64-156)	2.85(1.78-4.31)	0.09(-0.02 to 0.19)	775(456-1268)	55.65(32.7-90.36)	1925(1183-2919)	49.46(30.65-74.62)	-0.37(-0.52 to -0.21)
Zambia	92(64-130)	11.72(8.13-16.44)	385(162-666)	19.15(8.61-32.35)	1.6(1.33 to 1.88)	2157(1485-3063)	256.28(177.14-363)	8649(3406-15185)	399.58(167.13-687.2)	1.46(1.17 to 1.75)
Zimbabwe	87(60-121)	7.9(5.47-10.97)	210(143-298)	10.83(7.43-15.26)	1.13(0.79 to 1.48)	1776(1218-2510)	152.8(105.18-215.09)	4702(3172-6675)	224.78(153.13-316.83)	1.31(1.05 to 1.57)

S9 Table: Absolute and relative cross-country inequality for overall head and neck cancer of age-standardized DALYs rate in middle-aged and older adults from 1990 to 2021.

**location**	**Overall head and neck cancers**					
	**SII_1990**	**SII_2021**	**SII_AbsDiff**	**CI_1990**	**CI_2021**	**CI_AbsDiff**
Global	100.54 (10.65 to 190.43)	-45.97 (-113.84 to 21.91)	-146.51	-0.1223 (-0.0903 to -0.1542)	-0.1991 (-0.1544 to -0.2438)	-0.0768
Andean Latin America	-132.14 (-236.52 to -27.76)	-114.43 (-295.22 to 66.35)	17.71	-0.0812 (0.0118 to -0.1741)	-0.0884 (0.0701 to -0.2468)	-0.0072
Caribbean	90.38 (-96.66 to 277.41)	-67.11 (-292.09 to 157.88)	-157.48	0.0341 (0.1028 to -0.0345)	-0.0289 (0.0787 to -0.1365)	-0.063
Central Asia	197.97 (-35.22 to 431.17)	194.73 (65.94 to 323.51)	-3.25	0.0793 (0.1600 to -0.0014)	0.1292 (0.2099 to 0.0484)	0.0499
Central Europe	184.78 (-75.41 to 444.97)	-127.34 (-426.09 to 171.41)	-312.12	0.0504 (0.1192 to -0.0183)	-0.0635 (0.0018 to -0.1289)	-0.114
Central Latin America	111.90 (15.18 to 208.61)	6.90 (-63.00 to 76.80)	-105	0.0190 (0.0940 to -0.0560)	-0.0828 (0.0188 to -0.1844)	-0.1018
Central Sub-Saharan Africa	38.55 (-29.30 to 106.40)	2.97 (-74.75 to 80.69)	-35.58	-0.0203 (0.0345 to -0.0751)	0.0261 (0.0565 to -0.0043)	0.0464
East Asia	-13.97 (-1420.94 to 1393.00)	480.53 (-753.40 to 1714.46)	494.5	-0.0018 (0.0246 to -0.0281)	0.0270 (0.0772 to -0.0232)	0.0287
Eastern Europe	-133.59 (-249.67 to -17.51)	-102.35 (-240.28 to 35.59)	31.24	-0.0072 (0.0174 to -0.0318)	-0.0308 (-0.0038 to -0.0578)	-0.0236
Eastern Sub-Saharan Africa	6.96 (-225.45 to 239.37)	167.04 (-43.10 to 377.17)	160.08	0.0126 (0.0873 to -0.0621)	0.1010 (0.1738 to 0.0283)	0.0885
High-income Asia Pacific	-943.11 (-1460.90 to -425.32)	-299.86 (-627.74 to 28.03)	643.25	-0.1631 (-0.0426 to -0.2836)	-0.0407 (0.0633 to -0.1446)	0.1224
High-income North America	-1318.20 (-3000.94 to 364.53)	-590.82 (-1242.30 to 60.65)	727.38	0.0089 (0.0350 to -0.0173)	-0.0138 (0.0041 to -0.0317)	-0.0227
North Africa and Middle East	110.59 (-39.57 to 260.74)	-70.78 (-191.56 to 50.00)	-181.36	0.1002 (0.1852 to 0.0152)	-0.0100 (0.0761 to -0.0961)	-0.1102
Oceania	66.21 (-102.54 to 234.95)	50.61 (-180.57 to 281.80)	-15.59	0.0525 (0.1230 to -0.0181)	0.0581 (0.1238 to -0.0076)	0.0057
South Asia	-70.14 (-1408.25 to 1267.96)	238.76 (49.16 to 428.36)	308.9	-0.0693 (0.0050 to -0.1437)	-0.0517 (0.0778 to -0.1812)	0.0176
Southeast Asia	170.23 (-90.69 to 431.16)	227.20 (-21.76 to 476.17)	56.97	0.0192 (0.1219 to -0.0836)	0.0096 (0.1019 to -0.0827)	-0.0096
Southern Latin America	-132.29 (-1632.43 to 1367.84)	-384.75 (-464.34 to -305.16)	-252.46	0.0472 (0.2942 to -0.1999)	-0.1757 (-0.1337 to -0.2177)	-0.2229
Southern Sub-Saharan Africa	61.94 (-580.48 to 704.37)	-347.27 (-959.70 to 265.15)	-409.22	0.0163 (0.0624 to -0.0298)	-0.0413 (0.0017 to -0.0843)	-0.0576
Western Europe	-170.74 (-391.91 to 50.42)	-146.75 (-236.04 to -57.46)	23.99	-0.1191 (-0.0348 to -0.2033)	-0.0784 (-0.0370 to -0.1197)	0.0407

location	Overall head and neck cancers					
	SII_1990	SII_2021	SII_AbsDiff	CI_1990	CI_2021	CI_AbsDiff
Western Sub-Saharan Africa	-63.75 (-159.34 to 31.85)	-60.27 (-161.43 to 40.90)	3.48	0.0203 (0.0872 to -0.0466)	-0.0303 (0.0259 to -0.0864)	-0.0506

SII, slope index of inequality. SII_AbsDiff=SII_1990-SII_2021 (the absolute value of the difference between SII_1990 and SII_2021).

CI, concentration index.CI_AbsDiff=CI_1990-CI_2021 (the absolute value of the difference between CI_1990 and CI_2021).

S10 Table: Frontier analysis on the basis of sociodemographic-index and age-standardized DALYs rate of overall head and neck cancer in middle-aged and older adults from 1990 to 2021.

Location	SDI	Rate of DALYs	Frontier DALYs	Effective difference	Effective difference rank (ASDR)
Afghanistan	0.3372	344.76(177.52 to 558.89)	64.97	279.78	129 (127)
Albania	0.7068	330.24(217.63 to 482.85)	64.84	265.4	122 (120)
Algeria	0.6595	304.64(197.43 to 448.96)	64.8	239.84	111 (109)
American Samoa	0.7237	224.67(151.04 to 322.2)	64.83	159.83	60 (58)
Andorra	0.8694	142.86(79.2 to 235.11)	64.98	77.88	22 (17)
Angola	0.4537	337.07(216.32 to 509.1)	65.06	272.01	125 (123)
Antigua and Barbuda	0.7499	293.65(246.31 to 346.85)	64.67	228.99	104 (102)
Argentina	0.7231	272.18(221.35 to 330.92)	64.75	207.43	93 (92)
Armenia	0.7018	276.55(234.29 to 322.4)	64.91	211.64	96 (96)
Australia	0.8443	178.78(145.16 to 217.33)	64.86	113.92	40 (36)
Austria	0.8538	231.23(187.47 to 279.98)	64.85	166.38	64 (63)
Azerbaijan	0.6949	233.64(162.24 to 347.13)	64.68	168.96	67 (65)
Bahamas	0.8050	486.84(374.94 to 625.62)	64.85	421.99	164 (164)
Bahrain	0.7530	235.55(143.19 to 370.07)	64.86	170.69	71 (70)
Bangladesh	0.4924	662.01(388.98 to 1041.33)	64.86	597.16	188 (188)
Barbados	0.7467	322.36(240.09 to 418.99)	64.88	257.47	118 (116)
Belarus	0.7845	579.99(426.36 to 775.67)	65.03	514.96	180 (180)
Belgium	0.8537	267.65(215.89 to 327.05)	64.87	202.78	90 (89)
Belize	0.6102	247.33(202.85 to 296.84)	65	182.33	84 (83)
Benin	0.3735	187.91(116.35 to 288.14)	64.85	123.06	43 (39)

Location	SDI	Rate of DALYs	Frontier DALYs	Effective difference	Effective difference rank (ASDR)
Bermuda	0.8214	317.53(235.22 to 421.27)	64.99	252.54	116 (114)
Bhutan	0.4731	607.27(374.1 to 941.39)	64.79	542.48	185 (185)
Bolivia (Plurinational State of)	0.5990	199.98(123.97 to 304.87)	64.63	135.35	51 (47)
Bosnia and Herzegovina	0.7231	422.19(300.13 to 558.33)	64.85	357.34	147 (144)
Botswana	0.6427	457.82(271.39 to 740.18)	64.77	393.05	160 (158)
Brazil	0.6530	414.59(378.02 to 451.5)	64.78	349.8	146 (143)
Brunei Darussalam	0.8102	456.36(317.43 to 634.81)	64.88	391.48	159 (156)
Bulgaria	0.7682	568.08(450.42 to 702.76)	64.98	503.1	178 (178)
Burkina Faso	0.2851	212.39(132.27 to 323.97)	143.64	68.74	16 (50)
Burundi	0.2894	462.33(280.9 to 726.53)	143.16	319.17	141 (159)
Cabo Verde	0.5335	410.89(254.64 to 624.88)	64.81	346.08	145 (142)
Cambodia	0.4736	504.14(318 to 771.48)	64.91	439.24	170 (170)
Cameroon	0.4797	233.9(142.04 to 366.52)	64.92	168.98	68 (67)
Canada	0.8732	175.56(144.1 to 211.91)	64.81	110.76	38 (34)
Central African Republic	0.3092	357.26(209.3 to 566.3)	141.83	215.43	98 (134)
Chad	0.2404	227.74(140.56 to 343.13)	144.75	82.99	26 (61)
Chile	0.7715	118.38(96.51 to 143.8)	64.68	53.7	6 (4)
China	0.7216	295.5(228.86 to 375.17)	64.98	230.52	105 (103)
Colombia	0.6554	163.4(124.11 to 209.44)	64.77	98.63	35 (30)
Comoros	0.4760	438.53(271.72 to 683.58)	64.97	373.56	154 (151)
Congo	0.5831	354.64(228.18 to 529.49)	64.81	289.82	134 (132)
Cook Islands	0.7791	141.19(92.23 to 208.64)	65.02	76.17	20 (16)

Location	SDI	Rate of DALYs	Frontier DALYs	Effective difference	Effective difference rank (ASDR)
Costa Rica	0.7003	169.73(132.5 to 213.69)	64.89	104.85	36 (32)
Coted'Ivoire	0.4259	276.47(163.78 to 445.05)	64.65	211.82	97 (95)
Croatia	0.7983	436.57(348.79 to 540.48)	64.99	371.57	152 (149)
Cuba	0.6687	697.25(539.66 to 893.8)	64.67	632.59	191 (191)
Cyprus	0.8356	157.12(107.74 to 226.13)	64.77	92.35	29 (24)
Czechia	0.8285	347.59(280.42 to 428.09)	64.76	282.83	132 (130)
Democratic People's Republic of Korea	0.5699	303.89(186.81 to 461.39)	64.85	239.04	110 (108)
Democratic Republic of the Congo	0.3832	290.07(180.73 to 435.86)	64.88	225.19	102 (100)
Denmark	0.8964	218.4(185.1 to 254.65)	64.95	153.45	56 (54)
Djibouti	0.4880	514.43(298.45 to 833.63)	64.8	449.62	172 (172)
Dominica	0.7470	478.26(320.33 to 685.07)	64.89	413.38	162 (162)
Dominican Republic	0.6194	356.73(224.85 to 549.24)	64.91	291.81	135 (133)
Ecuador	0.6610	118.91(87.01 to 160.15)	64.98	53.92	7 (5)
Egypt	0.6068	142.88(100.52 to 198.57)	65.08	77.8	21 (18)
El Salvador	0.5638	158.26(116.49 to 211.63)	64.71	93.55	30 (25)
Equatorial Guinea	0.6579	299.05(173.84 to 476.82)	64.97	234.08	108 (106)
Eritrea	0.4039	514.74(325.87 to 774.51)	64.62	450.13	173 (173)
Estonia	0.8449	346.78(265.79 to 438.15)	64.98	281.8	131 (129)
Eswatini	0.5855	614.8(350.57 to 993.83)	64.93	549.87	186 (186)
Ethiopia	0.3588	342.77(242.33 to 481.7)	64.9	277.87	127 (125)
Fiji	0.6751	237.34(154.08 to 349.74)	64.87	172.48	72 (71)
Finland	0.8598	143.11(121.08 to 167.52)	64.7	78.41	24 (19)

Location	SDI	Rate of DALYs	Frontier DALYs	Effective difference	Effective difference rank (ASDR)
France	0.8384	318.45(256.26 to 391.26)	64.68	253.77	117 (115)
Gabon	0.6347	364.98(233.25 to 544.46)	64.88	300.1	137 (136)
Gambia	0.4097	161.7(102.84 to 238.89)	64.84	96.86	32 (27)
Georgia	0.7325	529.95(446.68 to 620.44)	64.99	464.96	174 (174)
Germany	0.9030	233.96(190.43 to 281.53)	64.77	169.19	69 (68)
Ghana	0.5649	124.73(76.42 to 188.72)	65.04	59.69	12 (10)
Greece	0.7919	295.98(257.58 to 336.51)	64.83	231.15	106 (104)
Greenland	0.8262	744.87(522.12 to 1044.9)	65.06	679.81	195 (195)
Grenada	0.6690	343.21(263.44 to 436.02)	64.65	278.56	128 (126)
Guam	0.8040	284.83(215.28 to 368.85)	64.94	219.89	101 (99)
Guatemala	0.5400	123.52(100.82 to 148.9)	64.89	58.64	11 (9)
Guinea	0.3364	432.98(277.2 to 637.61)	64.68	368.31	150 (147)
Guinea-Bissau	0.3531	281.65(171.87 to 425.22)	64.97	216.68	99 (97)
Guyana	0.6508	249.49(174.45 to 346.14)	64.96	184.53	85 (84)
Haiti	0.4483	448.12(260.4 to 708.45)	64.9	383.22	158 (155)
Honduras	0.5130	233.74(151.83 to 342.08)	64.85	168.89	66 (66)
Hungary	0.7908	733.05(592.89 to 890.42)	64.79	668.26	194 (194)
Iceland	0.8764	155.69(128.84 to 185.22)	64.89	90.8	28 (23)
India	0.5754	792.4(668.9 to 921.83)	64.84	727.55	201 (201)
Indonesia	0.6569	339.94(239.21 to 456.92)	65.04	274.9	126 (124)
Iran (Islamic Republic of)	0.6972	189.45(163.18 to 217.08)	64.93	124.52	44 (40)
Iraq	0.6626	273.78(174.66 to 397.79)	64.98	208.8	95 (94)

Location	SDI	Rate of DALYs	Frontier DALYs	Effective difference	Effective difference rank (ASDR)
Ireland	0.8738	186.27(150.62 to 227.29)	64.88	121.39	42 (38)
Israel	0.8090	145.69(116.62 to 178.77)	64.96	80.74	25 (21)
Italy	0.8058	240.86(218.07 to 262.18)	64.95	175.91	77 (76)
Jamaica	0.6833	244.94(168 to 345.66)	64.88	180.05	80 (79)
Japan	0.8712	130.67(118.74 to 139.58)	64.84	65.84	15 (13)
Jordan	0.7253	161.13(102.8 to 244.23)	64.75	96.38	31 (26)
Kazakhstan	0.7251	314.17(260.86 to 373.45)	64.82	249.34	115 (113)
Kenya	0.5238	582.83(430.89 to 762.16)	64.88	517.95	181 (181)
Kiribati	0.5272	500.81(296.3 to 804.03)	65.04	435.76	168 (168)
Kuwait	0.8467	85.11(62.86 to 113.09)	64.95	20.16	3 (2)
Kyrgyzstan	0.6040	240.65(178.53 to 314.38)	64.97	175.69	76 (75)
Lao People's Democratic Republic	0.4891	387.43(245.5 to 584.42)	64.86	322.56	142 (139)
Latvia	0.8307	482.48(373.98 to 615.01)	64.73	417.75	163 (163)
Lebanon	0.7447	253.42(172.31 to 362.49)	64.94	188.48	87 (86)
Lesotho	0.5104	712.84(423.4 to 1116.92)	64.85	647.99	192 (192)
Liberia	0.3524	191.73(112.61 to 305.05)	65.39	126.34	47 (43)
Libya	0.7258	572.79(367.94 to 862.37)	64.98	507.81	179 (179)
Lithuania	0.8565	539.39(420.07 to 673.42)	64.84	474.54	175 (175)
Luxembourg	0.8844	244.12(206.26 to 284.93)	64.88	179.23	79 (78)
Madagascar	0.4002	362.19(228.1 to 549.61)	64.96	297.23	136 (135)
Malawi	0.3846	262.39(169.41 to 389.85)	64.88	197.51	88 (87)
Malaysia	0.7425	748.96(510.39 to 1060.06)	64.65	684.31	196 (196)

Location	SDI	Rate of DALYs	Frontier DALYs	Effective difference	Effective difference rank (ASDR)
Maldives	0.6509	190.87(127.3 to 273.19)	64.69	126.18	46 (42)
Mali	0.2686	215.94(137.33 to 326.91)	144.7	71.23	17 (52)
Malta	0.8016	246.02(191.82 to 308.35)	64.77	181.25	82 (81)
Marshall Islands	0.5741	308.19(184.85 to 477.17)	64.87	243.32	113 (111)
Mauritania	0.4989	189.51(116.02 to 292.56)	64.86	124.65	45 (41)
Mauritius	0.7183	392.94(333.96 to 453.79)	64.85	328.09	144 (141)
Mexico	0.6646	143.31(123.37 to 165.2)	65.02	78.29	23 (20)
Micronesia (Federated States of)	0.5875	301.76(191.86 to 456.13)	64.96	236.79	109 (107)
Monaco	0.9083	389.42(263.65 to 567.81)	64.85	324.57	143 (140)
Mongolia	0.6176	252.51(170.44 to 355.17)	64.97	187.54	86 (85)
Montenegro	0.7958	646.01(481.84 to 867.41)	64.87	581.14	187 (187)
Morocco	0.5627	323.85(202.44 to 489.63)	64.89	258.96	119 (117)
Mozambique	0.3265	331.8(211.87 to 498.37)	64.87	266.92	124 (122)
Myanmar	0.5339	323.96(206.59 to 481.91)	64.95	259.01	120 (118)
Namibia	0.6176	764.33(483.96 to 1127.9)	64.98	699.36	198 (198)
Nauru	0.6252	345.59(192.3 to 579.21)	64.94	280.65	130 (128)
Nepal	0.4332	668.46(423.49 to 1013.42)	64.68	603.78	189 (189)
Netherlands	0.8885	177.16(145.67 to 211.82)	64.84	112.32	39 (35)
New Zealand	0.8494	148.28(121.55 to 177.77)	64.84	83.45	27 (22)
Nicaragua	0.5240	122.89(83.16 to 176.19)	64.97	57.92	10 (8)
Niger	0.1681	168.91(98.62 to 270.01)	144.76	24.15	4 (31)
Nigeria	0.5034	233.39(161.26 to 319.36)	64.87	168.52	65 (64)

Location	SDI	Rate of DALYs	Frontier DALYs	Effective difference	Effective difference rank (ASDR)
Niue	0.7262	245.99(158.83 to 365.01)	64.84	181.14	81 (80)
North Macedonia	0.7506	471.12(341.39 to 642.83)	64.98	406.14	161 (160)
Northern Mariana Islands	0.7715	752.94(525.07 to 1044)	64.95	687.99	197 (197)
Norway	0.9161	124.96(113.15 to 135.19)	64.88	60.08	13 (11)
Oman	0.7734	136.28(84.77 to 205.65)	64.94	71.33	18 (15)
Pakistan	0.5040	1714.33(1252.39 to 2330.31)	64.73	1649.61	204 (204)
Palau	0.7540	1199.79(798.87 to 1756.5)	64.65	1135.14	203 (203)
Palestine	0.6310	130.52(89.93 to 186.66)	64.89	65.63	14 (12)
Panama	0.7089	170.07(126.58 to 213.55)	64.89	105.18	37 (33)
Papua New Guinea	0.4178	223.23(122.71 to 366.31)	64.81	158.42	59 (57)
Paraguay	0.6357	313.99(197.87 to 473.78)	64.84	249.15	114 (112)
Peru	0.6621	116.26(76.68 to 169.58)	64.83	51.43	5 (3)
Philippines	0.6512	348.28(285.33 to 419.05)	64.89	283.39	133 (131)
Poland	0.8120	543(486.54 to 598.79)	64.75	478.25	176 (176)
Portugal	0.7442	375.18(299.59 to 464.4)	64.96	310.22	139 (138)
Puerto Rico	0.8255	209.36(156.04 to 273.53)	64.98	144.38	53 (49)
Qatar	0.8469	192.91(116.33 to 303.11)	64.83	128.08	48 (44)
Republic of Korea	0.8867	121.81(85.55 to 164.11)	64.96	56.85	9 (7)
Republic of Moldova	0.7322	561.81(472.71 to 662.97)	65.13	496.68	177 (177)
Romania	0.7685	713.12(563.04 to 889.89)	64.85	648.27	193 (193)
Russian Federation	0.8085	433.2(386.47 to 477.27)	64.75	368.45	151 (148)
Rwanda	0.4356	489.12(300.66 to 763.02)	64.96	424.17	165 (165)

Location	SDI	Rate of DALYs	Frontier DALYs	Effective difference	Effective difference rank (ASDR)
Saint Kitts and Nevis	0.7550	365.07(283.87 to 457.02)	64.83	300.23	138 (137)
Saint Lucia	0.6725	446.13(345.38 to 562.81)	65.02	381.11	156 (153)
Saint Vincent and the Grenadines	0.6372	586.92(487.3 to 704.67)	64.97	521.95	182 (182)
Samoa	0.5934	269.59(173.09 to 404.19)	64.74	204.85	92 (91)
San Marino	0.8880	180.52(96.95 to 298.33)	64.8	115.72	41 (37)
Sao Tome and Principe	0.5054	74.86(47.96 to 111.88)	64.99	9.87	2 (1)
Saudi Arabia	0.8151	219.66(147.35 to 319.25)	64.71	154.95	58 (56)
Senegal	0.4081	212.56(129.17 to 327)	65.05	147.51	54 (51)
Serbia	0.7924	504.26(340.78 to 703.19)	64.82	439.44	171 (171)
Seychelles	0.7302	968.08(642.13 to 1394.26)	64.87	903.21	202 (202)
Sierra Leone	0.3587	199.89(125.97 to 306.45)	64.85	135.04	50 (46)
Singapore	0.8561	272.93(209.38 to 346.85)	65.13	207.8	94 (93)
Slovakia	0.8106	594.43(389.64 to 856.73)	65.02	529.41	183 (183)
Slovenia	0.8424	263.24(206.31 to 331.47)	64.83	198.41	89 (88)
Solomon Islands	0.4294	291.43(177.52 to 453.48)	64.9	226.53	103 (101)
Somalia	0.0777	477.19(287.36 to 756.47)	477.19	0	1 (161)
South Africa	0.6796	431.67(369.14 to 498.65)	64.87	366.8	148 (145)
South Sudan	0.2784	457.02(272.84 to 730.66)	144.14	312.88	140 (157)
Spain	0.7693	306.93(243.95 to 380.04)	64.99	241.94	112 (110)
Sri Lanka	0.7015	491.5(277.92 to 782.07)	64.81	426.68	166 (166)
Sudan	0.5419	218.16(131.97 to 340.3)	64.87	153.29	55 (53)
Suriname	0.6337	231.01(147.88 to 345.46)	64.83	166.17	63 (62)

Location	SDI	Rate of DALYs	Frontier DALYs	Effective difference	Effective difference rank (ASDR)
Sweden	0.8869	121.37(101.5 to 142.56)	64.89	56.49	8 (6)
Switzerland	0.9331	161.81(129.71 to 199.28)	64.82	96.99	33 (28)
Syrian Arab Republic	0.6230	136.24(93.99 to 191.99)	64.88	71.36	19 (14)
Taiwan (Province of China)	0.8747	776.31(614.83 to 961.79)	64.85	711.46	199 (199)
Tajikistan	0.5415	162.09(105.06 to 237.37)	65.08	97.01	34 (29)
Thailand	0.6825	448.07(299.27 to 641.73)	64.89	383.18	157 (154)
Timor-Leste	0.4447	328.86(206.53 to 504.62)	64.71	264.15	121 (119)
Togo	0.4085	235.49(143.94 to 359.51)	64.89	170.6	70 (69)
Tokelau	0.6864	239.78(149.36 to 364.1)	64.81	174.97	74 (73)
Tonga	0.6263	225.71(138.35 to 347.11)	64.8	160.91	62 (60)
Trinidad and Tobago	0.7688	246.23(179.57 to 326.45)	64.85	181.38	83 (82)
Tunisia	0.6824	432.49(262.99 to 667.1)	64.76	367.74	149 (146)
Turkey	0.7127	240.46(161.26 to 344.16)	64.84	175.62	75 (74)
Turkmenistan	0.6822	330.53(250.11 to 441.24)	64.88	265.65	123 (121)
Tuvalu	0.5766	268.03(177.39 to 394.53)	64.76	203.27	91 (90)
Uganda	0.4233	686.57(431.34 to 1044.4)	64.87	621.7	190 (190)
Ukraine	0.7608	495.13(318.06 to 725.94)	64.96	430.17	167 (167)
United Arab Emirates	0.8493	237.55(158.13 to 346.71)	64.8	172.76	73 (72)
United Kingdom	0.8590	218.77(206.01 to 229.92)	65.02	153.76	57 (55)
United Republic of Tanzania	0.4466	440.08(265.7 to 699.81)	64.68	375.4	155 (152)
United States of America	0.8624	203.75(190.93 to 214.4)	64.65	139.09	52 (48)
United States Virgin Islands	0.8218	199.33(122.41 to 308.75)	65	134.33	49 (45)

Location	SDI	Rate of DALYs	Frontier DALYs	Effective difference	Effective difference rank (ASDR)
Uruguay	0.7193	436.88(351.82 to 535.37)	64.97	371.91	153 (150)
Uzbekistan	0.6626	224.88(170.86 to 293.88)	64.69	160.18	61 (59)
Vanuatu	0.4731	242.31(157.51 to 364.37)	65.07	177.24	78 (77)
Venezuela (Bolivarian Republic of)	0.5965	297.1(216.98 to 396.4)	64.93	232.17	107 (105)
Viet Nam	0.6279	607.13(386.03 to 912.51)	64.88	542.25	184 (184)
Yemen	0.4504	281.92(161.52 to 439.16)	64.69	217.23	100 (98)
Zambia	0.5059	782.26(325.89 to 1516.65)	64.89	717.36	200 (200)
Zimbabwe	0.4738	503.4(334.71 to 728.45)	65.06	438.34	169 (169)

S11 Table: Absolute and relative cross-country inequality for larynx cancer of age-standardized DALYs rate in middle-aged and older adults from 1990 to 2021.

location	Larynx cancer					
	**SII_1990**	**SII_2021**	**SII_AbsDiff**	**CI_1990**	**CI_2021**	**CI_AbsDiff**
Global	61.85 (14.92 to 108.78)	-23.12 (-49.19 to 2.95)	-84.97	-0.0338 (0.0062 to -0.0737)	-0.1739 (-0.1336 to -0.2141)	-0.1401
Andean Latin America	-64.75 (-115.08 to -14.42)	-55.55 (-154.30 to 43.20)	9.2	-0.0778 (0.0097 to -0.1653)	-0.1077 (0.1185 to -0.3339)	-0.0299
Caribbean	38.99 (-43.88 to 121.87)	-36.65 (-139.03 to 65.73)	-75.64	0.0464 (0.1508 to -0.0581)	-0.0023 (0.1527 to -0.1573)	-0.0486
Central Asia	249.53 (164.18 to 334.88)	151.68 (48.71 to 254.65)	-97.85	0.1345 (0.1983 to 0.0707)	0.2246 (0.3927 to 0.0565)	0.0901
Central Europe	16.44 (-112.97 to 145.85)	-157.05 (-251.22 to -62.87)	-173.48	-0.0029 (0.0536 to -0.0594)	-0.1042 (-0.0480 to -0.1604)	-0.1013
Central Latin America	52.90 (-15.64 to 121.45)	-7.50 (-33.98 to 18.98)	-60.4	0.0374 (0.1178 to -0.0431)	-0.1145 (0.0358 to -0.2649)	-0.1519
Central Sub-Saharan Africa	-3.39 (-50.47 to 43.68)	-20.30 (-61.81 to 21.22)	-16.9	-0.0376 (0.0234 to -0.0986)	0.0164 (0.0561 to -0.0232)	0.054
East Asia	-76.58 (-132.53 to -20.63)	-23.69 (-86.03 to 38.65)	52.89	-0.0107 (-0.0062 to -0.0152)	-0.0053 (0.0048 to -0.0153)	0.0054
Eastern Europe	-57.47 (-96.96 to -17.99)	-66.35 (-135.04 to 2.34)	-8.88	-0.0100 (0.0002 to -0.0202)	-0.0320 (0.0022 to -0.0662)	-0.022
Eastern Sub-Saharan Africa	-13.50 (-87.36 to 60.36)	-6.08 (-62.07 to 49.90)	7.42	-0.0141 (0.0650 to -0.0933)	0.0241 (0.1162 to -0.0680)	0.0382
High-income Asia Pacific	-190.42 (-402.98 to 22.15)	-10.87 (-69.75 to 48.01)	179.55	-0.2857 (-0.2172 to -0.3541)	0.1475 (0.2605 to 0.0345)	0.4331
High-income North America	-102.91 (-252.82 to 47.00)	-59.92 (-80.29 to -39.54)	42.99	0.0069 (0.0130 to 0.0008)	-0.0299 (-0.0282 to -0.0315)	-0.0368
North Africa and Middle East	1.95 (-88.87 to 92.77)	-88.04 (-150.39 to -25.68)	-89.99	0.0618 (0.1576 to -0.0339)	-0.0459 (0.0377 to -0.1296)	-0.1077
Oceania	-0.96 (-39.77 to 37.86)	-12.74 (-56.54 to 31.06)	-11.79	0.0231 (0.0740 to -0.0279)	0.0269 (0.0671 to -0.0132)	0.0039

location	Larynx cancer					
	SII_1990	SII_2021	SII_AbsDiff	CI_1990	CI_2021	CI_AbsDiff
South Asia	-13.46 (-480.76 to 453.83)	38.63 (32.92 to 44.34)	52.09	-0.0572 (-0.0124 to -0.1020)	-0.0429 (0.0564 to -0.1423)	0.0143
Southeast Asia	17.86 (-46.71 to 82.44)	22.01 (-25.72 to 69.73)	4.14	-0.0204 (0.0723 to -0.1131)	-0.0048 (0.0570 to -0.0665)	0.0157
Southern Latin America	-73.84 (-1081.73 to 934.05)	-207.16 (-230.92 to -183.41)	-133.32	0.0569 (0.3260 to -0.2122)	-0.1962 (-0.1713 to -0.2211)	-0.2532
Southern Sub-Saharan Africa	-41.09 (-204.73 to 122.55)	-171.89 (-517.59 to 173.82)	-130.8	-0.0216 (0.0067 to -0.0499)	-0.0680 (-0.0286 to -0.1073)	-0.0463
Western Europe	-135.43 (-270.77 to -0.09)	-82.13 (-120.62 to -43.64)	53.3	-0.1976 (-0.1139 to -0.2813)	-0.1545 (-0.0990 to -0.2099)	0.0431
Western Sub-Saharan Africa	-10.69 (-42.20 to 20.82)	-5.35 (-38.90 to 28.19)	5.34	0.0506 (0.0985 to 0.0028)	-0.0071 (0.0173 to -0.0314)	-0.0577

SII, slope index of inequality. SII_AbsDiff=SII_1990-SII_2021 (the absolute value of the difference between SII_1990 and SII_2021).

CI, concentration index.CI_AbsDiff=CI_1990-CI_2021 (the absolute value of the difference between CI_1990 and CI_2021).

S12 Table: Frontier analysis on the basis of sociodemographic-index and age-standardized DALYs rate of larynx cancer in middle-aged and older adults from 1990 to 2021.

Location	SDI	Rate of DALYs	Frontier DALYs	Effective difference	Effective difference rank (ASDR)
Afghanistan	0.3372	232.36(118.96 to 377.83)	42.28	190.07	179 (186)
Albania	0.7068	181.95(121.82 to 266)	17.53	164.43	168 (168)
Algeria	0.6595	97.05(64.41 to 141.39)	17.53	79.52	101 (95)
American Samoa	0.7237	40.28(27.04 to 57.62)	17.52	22.76	26 (22)
Andorra	0.8694	61.11(33.92 to 99.14)	17.55	43.57	53 (49)
Angola	0.4537	136.43(88.26 to 205.71)	17.56	118.87	140 (135)
Antigua and Barbuda	0.7499	133.13(110.55 to 158.9)	17.53	115.6	137 (133)
Argentina	0.7231	140.75(113.67 to 171.89)	17.53	123.22	143 (140)
Armenia	0.7018	157.91(137.24 to 181.5)	17.53	140.37	158 (156)
Australia	0.8443	42.52(34.13 to 52.27)	17.53	24.99	29 (26)
Austria	0.8538	72.32(58.1 to 88.1)	17.54	54.77	66 (60)
Azerbaijan	0.6949	162.51(118.5 to 237.37)	17.52	144.98	162 (161)
Bahamas	0.8050	209.63(161.35 to 269.73)	17.53	192.1	181 (180)
Bahrain	0.7530	105.51(67.38 to 165.05)	17.53	87.98	111 (105)
Bangladesh	0.4924	191.1(119.75 to 292.85)	17.53	173.57	171 (171)
Barbados	0.7467	123.59(91.9 to 160.51)	17.53	106.06	130 (125)
Belarus	0.7845	224.13(162.4 to 303.13)	17.52	206.61	185 (184)
Belgium	0.8537	89.21(71.33 to 109.11)	17.55	71.66	93 (85)
Belize	0.6102	128.05(104.7 to 154.77)	17.52	110.52	134 (130)
Benin	0.3735	88.59(55.95 to 134.48)	37.38	51.21	62 (84)
Bermuda	0.8214	117.34(87.38 to 156)	17.53	99.82	126 (120)

Location	SDI	Rate of DALYs	Frontier DALYs	Effective difference	Effective difference rank (ASDR)
Bhutan	0.4731	160.92(99.44 to 248.68)	17.53	143.4	161 (160)
Bolivia (Plurinational State of)	0.5990	84.99(53.5 to 130.29)	17.54	67.44	89 (79)
Bosnia and Herzegovina	0.7231	246.06(171.25 to 323.03)	17.53	228.54	189 (189)
Botswana	0.6427	158.08(95.95 to 265.02)	17.53	140.56	159 (157)
Brazil	0.6530	200.7(182.81 to 218.89)	17.53	183.17	174 (174)
Brunei Darussalam	0.8102	62.41(43.33 to 87.22)	17.54	44.88	57 (53)
Bulgaria	0.7682	307.3(244.74 to 379.09)	17.52	289.77	199 (199)
Burkina Faso	0.2851	102.3(63.02 to 157.17)	44.58	57.72	74 (101)
Burundi	0.2894	127.69(79.65 to 200.51)	44.49	83.21	105 (129)
Cabo Verde	0.5335	121.67(71.01 to 185.07)	17.53	104.13	128 (124)
Cambodia	0.4736	128.49(80.04 to 206.99)	17.53	110.97	135 (131)
Cameroon	0.4797	110.64(63.99 to 178.76)	17.53	93.11	116 (111)
Canada	0.8732	50.36(40.87 to 61.32)	17.53	32.83	34 (31)
Central African Republic	0.3092	160.72(87.04 to 256.44)	43.33	117.4	139 (159)
Chad	0.2404	118.56(70.87 to 182)	53.33	65.23	87 (122)
Chile	0.7715	51.36(41.32 to 62.87)	17.53	33.83	35 (32)
China	0.7216	77.13(58.92 to 98.76)	17.52	59.6	80 (71)
Colombia	0.6554	67.77(50.94 to 87.76)	17.53	50.24	61 (56)
Comoros	0.4760	116.02(74.72 to 176.15)	17.53	98.49	123 (118)
Congo	0.5831	140.75(90.76 to 213.29)	17.53	123.22	144 (141)
Cook Islands	0.7791	25.68(16.97 to 38.97)	17.58	8.09	8 (6)
Costa Rica	0.7003	57.27(44.23 to 73.01)	17.53	39.74	47 (44)

Location	SDI	Rate of DALYs	Frontier DALYs	Effective difference	Effective difference rank (ASDR)
Coted'Ivoire	0.4259	102.54(60.71 to 172.53)	17.53	85.01	108 (102)
Croatia	0.7983	192.32(151 to 241.72)	17.53	174.79	173 (173)
Cuba	0.6687	412.98(320.3 to 531.74)	17.53	395.45	203 (203)
Cyprus	0.8356	65.42(45.09 to 94.5)	17.54	47.88	59 (55)
Czechia	0.8285	113.88(90.68 to 141.92)	17.53	96.35	120 (115)
Democratic People's Republic of Korea	0.5699	76.41(47.3 to 114.68)	17.53	58.88	78 (69)
Democratic Republic of the Congo	0.3832	118.66(73.48 to 177.84)	38.33	80.33	103 (123)
Denmark	0.8964	77.64(63.43 to 93.78)	17.53	60.12	81 (72)
Djibouti	0.4880	155.27(93.43 to 242.32)	17.53	137.74	155 (153)
Dominica	0.7470	205.42(137.09 to 294.27)	17.53	187.88	178 (178)
Dominican Republic	0.6194	134.25(86.79 to 199.38)	17.53	116.73	138 (134)
Ecuador	0.6610	42.21(30.52 to 57.61)	17.53	24.69	28 (25)
Egypt	0.6068	91.39(63.61 to 128.81)	17.53	73.86	94 (87)
El Salvador	0.5638	55.77(40.29 to 76.16)	17.53	38.24	42 (40)
Equatorial Guinea	0.6579	107.55(58.85 to 178.22)	17.53	90.02	114 (108)
Eritrea	0.4039	143.13(91.43 to 213.59)	38.25	104.88	129 (143)
Estonia	0.8449	115.53(87.73 to 147.67)	17.53	98.01	122 (117)
Eswatini	0.5855	250.7(137.65 to 396.3)	17.53	233.17	190 (190)
Ethiopia	0.3588	78.9(54.27 to 111.94)	39.34	39.57	45 (73)
Fiji	0.6751	53.3(34.67 to 78.83)	17.52	35.78	39 (37)
Finland	0.8598	38.17(30.6 to 47.35)	17.54	20.63	21 (17)
France	0.8384	123.92(98.28 to 153.42)	17.53	106.39	131 (126)

Location	SDI	Rate of DALYs	Frontier DALYs	Effective difference	Effective difference rank (ASDR)
Gabon	0.6347	143.13(90.43 to 213.78)	17.53	125.61	146 (142)
Gambia	0.4097	42(26.8 to 62.51)	38.15	3.85	5 (24)
Georgia	0.7325	290.54(249.93 to 334.9)	17.53	273.01	195 (195)
Germany	0.9030	75.93(61.23 to 92.76)	17.53	58.4	76 (67)
Ghana	0.5649	96.17(58.3 to 146.09)	17.53	78.64	100 (93)
Greece	0.7919	147.02(127.13 to 167.84)	17.53	129.5	148 (145)
Greenland	0.8262	107.37(72.93 to 156.41)	17.52	89.85	113 (107)
Grenada	0.6690	101.71(78.14 to 129.16)	17.53	84.18	106 (99)
Guam	0.8040	19.74(15.2 to 25.62)	17.53	2.21	4 (3)
Guatemala	0.5400	39.11(31.68 to 47.49)	17.53	21.58	25 (21)
Guinea	0.3364	109.33(67.24 to 168.61)	42.45	66.88	88 (109)
Guinea-Bissau	0.3531	139.74(81.97 to 208.8)	40.29	99.46	124 (138)
Guyana	0.6508	98.48(68.01 to 137.23)	17.53	80.95	104 (97)
Haiti	0.4483	200.73(116.32 to 316.92)	17.54	183.19	175 (175)
Honduras	0.5130	117.16(76.69 to 169.34)	17.53	99.63	125 (119)
Hungary	0.7908	277.99(216.98 to 347.45)	17.53	260.46	194 (194)
Iceland	0.8764	32.55(25.87 to 40.42)	17.53	15.03	15 (11)
India	0.5754	211.89(178.62 to 250.49)	17.53	194.36	182 (181)
Indonesia	0.6569	86.48(58.73 to 116.82)	17.53	68.94	91 (82)
Iran (Islamic Republic of)	0.6972	146.91(126.81 to 167.86)	17.52	129.39	147 (144)
Iraq	0.6626	173.02(110.41 to 252.04)	17.53	155.49	165 (165)
Ireland	0.8738	59.3(47.82 to 73.19)	17.54	41.76	50 (46)

Location	SDI	Rate of DALYs	Frontier DALYs	Effective difference	Effective difference rank (ASDR)
Israel	0.8090	60.57(48.3 to 74.85)	17.53	43.04	52 (48)
Italy	0.8058	94.98(85.94 to 103.9)	17.53	77.46	99 (91)
Jamaica	0.6833	110(74.2 to 156.34)	17.52	92.47	115 (110)
Japan	0.8712	19.63(17.88 to 21.08)	17.53	2.1	3 (2)
Jordan	0.7253	57.23(36.57 to 86.68)	17.53	39.7	46 (43)
Kazakhstan	0.7251	110.86(94.6 to 127.77)	17.53	93.33	117 (112)
Kenya	0.5238	117.93(86.31 to 154.42)	17.53	100.4	127 (121)
Kiribati	0.5272	17.64(10.99 to 27.47)	17.53	0.11	2 (1)
Kuwait	0.8467	31.05(22.82 to 41.21)	17.53	13.52	11 (8)
Kyrgyzstan	0.6040	69.11(51.18 to 88.96)	17.53	51.59	63 (57)
Lao People's Democratic Republic	0.4891	97.61(59.56 to 153.91)	17.52	80.09	102 (96)
Latvia	0.8307	170.51(130.69 to 218.56)	17.63	152.88	164 (164)
Lebanon	0.7447	151.62(104.34 to 215.1)	17.53	134.09	153 (151)
Lesotho	0.5104	306.8(183.74 to 463.75)	17.53	289.28	198 (198)
Liberia	0.3524	90.3(53.42 to 144.55)	41.39	48.91	60 (86)
Libya	0.7258	264.96(170.29 to 405.25)	17.53	247.43	192 (192)
Lithuania	0.8565	207.85(159.85 to 262.42)	17.53	190.32	180 (179)
Luxembourg	0.8844	85.49(72.1 to 99.75)	17.53	67.97	90 (80)
Madagascar	0.4002	95.39(61.41 to 142.9)	38.31	57.07	71 (92)
Malawi	0.3846	52.78(34.74 to 81.45)	38.11	14.67	13 (33)
Malaysia	0.7425	105.42(70.08 to 150.8)	17.53	87.89	110 (104)
Maldives	0.6509	37.43(24.36 to 55.08)	17.52	19.91	19 (15)

Location	SDI	Rate of DALYs	Frontier DALYs	Effective difference	Effective difference rank (ASDR)
Mali	0.2686	86.19(55.41 to 131.51)	45.38	40.81	49 (81)
Malta	0.8016	72.95(57.32 to 91.58)	17.53	55.42	68 (62)
Marshall Islands	0.5741	57.9(33.62 to 90.61)	17.52	40.38	48 (45)
Mauritania	0.4989	81.79(49.68 to 127.47)	17.53	64.26	86 (78)
Mauritius	0.7183	127.54(108.8 to 146.81)	17.52	110.02	133 (128)
Mexico	0.6646	62.35(52.9 to 73.02)	17.54	44.81	56 (52)
Micronesia (Federated States of)	0.5875	52.81(32.86 to 81.15)	17.57	35.24	36 (34)
Monaco	0.9083	303.13(204.82 to 443.8)	17.54	285.59	197 (197)
Mongolia	0.6176	73.62(50.26 to 105.05)	17.52	56.1	69 (63)
Montenegro	0.7958	434.43(328.06 to 586.1)	17.53	416.9	204 (204)
Morocco	0.5627	155.56(96.95 to 236.42)	17.53	138.03	156 (154)
Mozambique	0.3265	167.71(108.3 to 247.66)	42.58	125.12	145 (163)
Myanmar	0.5339	76.34(47.57 to 115.13)	17.53	58.81	77 (68)
Namibia	0.6176	227.94(145.43 to 337.29)	17.53	210.41	186 (185)
Nauru	0.6252	59.85(34.97 to 92.06)	17.52	42.32	51 (47)
Nepal	0.4332	189.58(119.06 to 290.41)	17.55	172.03	170 (170)
Netherlands	0.8885	54.05(43.91 to 65.58)	17.53	36.52	40 (38)
New Zealand	0.8494	31.58(25.76 to 38.52)	17.53	14.05	12 (9)
Nicaragua	0.5240	62.17(42.48 to 90.35)	17.52	44.65	55 (51)
Niger	0.1681	80.87(47 to 128.34)	68.33	12.54	10 (77)
Nigeria	0.5034	94.45(63.38 to 131.2)	17.52	76.93	97 (89)
Niue	0.7262	38.24(24.7 to 57.21)	17.53	20.71	22 (18)

Location	SDI	Rate of DALYs	Frontier DALYs	Effective difference	Effective difference rank (ASDR)
North Macedonia	0.7506	298.15(216.39 to 406.93)	17.52	280.62	196 (196)
Northern Mariana Islands	0.7715	48.93(32.61 to 70.07)	17.53	31.4	33 (30)
Norway	0.9161	29.9(26.55 to 33.08)	17.53	12.38	9 (7)
Oman	0.7734	38.5(24.7 to 57.98)	17.52	20.98	24 (19)
Pakistan	0.5040	402.24(282.56 to 561.21)	17.53	384.71	202 (202)
Palau	0.7540	34.02(21.16 to 52.85)	17.54	16.48	16 (12)
Palestine	0.6310	74.64(50.94 to 107.56)	17.52	57.11	72 (64)
Panama	0.7089	56.86(41.9 to 71.69)	17.53	39.33	43 (41)
Papua New Guinea	0.4178	38.08(23.05 to 61.8)	17.54	20.54	20 (16)
Paraguay	0.6357	149.57(94.32 to 226.03)	17.53	132.05	151 (148)
Peru	0.6621	43.69(28.1 to 65.13)	17.53	26.16	30 (27)
Philippines	0.6512	72.87(58.21 to 89.21)	17.53	55.34	67 (61)
Poland	0.8120	235.56(210.05 to 261)	17.53	218.04	188 (188)
Portugal	0.7442	147.84(117.11 to 183.5)	17.53	130.31	149 (146)
Puerto Rico	0.8255	75.08(55.67 to 99.46)	17.52	57.56	73 (65)
Qatar	0.8469	103.24(63.63 to 162.33)	17.53	85.71	109 (103)
Republic of Korea	0.8867	41.21(28.74 to 56.5)	17.53	23.68	27 (23)
Republic of Moldova	0.7322	251.09(212.3 to 294.94)	17.54	233.55	191 (191)
Romania	0.7685	311.95(245.89 to 389.18)	17.53	294.42	200 (200)
Russian Federation	0.8085	164.28(145.15 to 181.84)	17.53	146.76	163 (162)
Rwanda	0.4356	130.9(81.07 to 208.67)	17.52	113.38	136 (132)
Saint Kitts and Nevis	0.7550	139.33(108.73 to 176.52)	17.53	121.8	142 (137)

Location	SDI	Rate of DALYs	Frontier DALYs	Effective difference	Effective difference rank (ASDR)
Saint Lucia	0.6725	176.86(136.82 to 222.66)	17.53	159.33	167 (167)
Saint Vincent and the Grenadines	0.6372	235.45(195.22 to 282.43)	17.52	217.93	187 (187)
Samoa	0.5934	21.65(14.59 to 32.26)	17.53	4.13	6 (4)
San Marino	0.8880	75.79(40.28 to 124.84)	17.54	58.25	75 (66)
Sao Tome and Principe	0.5054	65.03(41.74 to 96.92)	17.52	47.51	58 (54)
Saudi Arabia	0.8151	52.85(35.12 to 77.27)	17.53	35.32	37 (35)
Senegal	0.4081	100.16(60.49 to 152.23)	38	62.15	84 (98)
Serbia	0.7924	267.43(180.35 to 374.12)	17.53	249.9	193 (193)
Seychelles	0.7302	338.95(227.66 to 482.8)	17.53	321.42	201 (201)
Sierra Leone	0.3587	96.59(60.38 to 150.88)	39.61	56.98	70 (94)
Singapore	0.8561	32.42(25.52 to 40.48)	17.53	14.89	14 (10)
Slovakia	0.8106	200.95(134.16 to 289.33)	17.53	183.42	176 (176)
Slovenia	0.8424	113.93(89.16 to 143.11)	17.57	96.37	121 (116)
Solomon Islands	0.4294	54.52(33.73 to 83.6)	17.53	36.99	41 (39)
Somalia	0.0777	150.86(87.62 to 238.93)	150.86	0	1 (150)
South Africa	0.6796	149.58(128.18 to 173.78)	17.53	132.05	152 (149)
South Sudan	0.2784	140.35(81.93 to 223.55)	44.56	95.79	119 (139)
Spain	0.7693	137.22(107.29 to 171.01)	17.53	119.69	141 (136)
Sri Lanka	0.7015	79.2(43.41 to 129.44)	17.53	61.67	83 (75)
Sudan	0.5419	152.77(90.41 to 240.8)	17.53	135.25	154 (152)
Suriname	0.6337	71.06(43.85 to 106.61)	17.53	53.54	64 (58)
Sweden	0.8869	22.13(17.61 to 27.15)	17.53	4.6	7 (5)

Location	SDI	Rate of DALYs	Frontier DALYs	Effective difference	Effective difference rank (ASDR)
Switzerland	0.9331	47.27(37.6 to 58.32)	17.54	29.73	32 (29)
Syrian Arab Republic	0.6230	93.77(64.13 to 133.08)	17.53	76.25	96 (88)
Taiwan (Province of China)	0.8747	53.13(41.84 to 66.68)	17.52	35.6	38 (36)
Tajikistan	0.5415	76.94(50.07 to 111.42)	17.52	59.42	79 (70)
Thailand	0.6825	106.55(68.79 to 159.49)	17.53	89.02	112 (106)
Timor-Leste	0.4447	79.06(48.65 to 122.19)	17.53	61.53	82 (74)
Togo	0.4085	113.59(67.89 to 174.01)	38.19	75.39	95 (114)
Tokelau	0.6864	34.78(21.5 to 55.04)	17.52	17.26	17 (13)
Tonga	0.6263	38.5(23.65 to 59.87)	17.53	20.98	23 (20)
Trinidad and Tobago	0.7688	102.28(74.37 to 135.69)	17.53	84.75	107 (100)
Tunisia	0.6824	174.63(105.04 to 271.72)	17.53	157.1	166 (166)
Turkey	0.7127	149.52(99.05 to 216.74)	17.53	131.99	150 (147)
Turkmenistan	0.6822	94.71(72.13 to 125.06)	17.6	77.11	98 (90)
Tuvalu	0.5766	44.86(29.42 to 66.39)	17.53	27.33	31 (28)
Uganda	0.4233	156.61(97.28 to 242.68)	17.53	139.08	157 (155)
Ukraine	0.7608	183.4(114.96 to 272.5)	17.52	165.87	169 (169)
United Arab Emirates	0.8493	80.35(53.22 to 116.57)	17.53	62.82	85 (76)
United Kingdom	0.8590	62(58.34 to 65.36)	17.52	44.48	54 (50)
United Republic of Tanzania	0.4466	111.75(68.14 to 187.14)	17.52	94.23	118 (113)
United States Virgin Islands	0.8218	88.36(54.42 to 133.87)	17.53	70.83	92 (83)
United States of America	0.8624	71.96(67.63 to 75.82)	17.53	54.43	65 (59)
Uruguay	0.7193	222.16(177.03 to 274.93)	17.53	204.63	184 (183)

Location	SDI	Rate of DALYs	Frontier DALYs	Effective difference	Effective difference rank (ASDR)
Uzbekistan	0.6626	56.99(43.04 to 74.06)	17.54	39.45	44 (42)
Vanuatu	0.4731	36.79(22.94 to 56.61)	17.53	19.26	18 (14)
Venezuela (Bolivarian Republic of)	0.5965	159.7(116.28 to 214.74)	17.52	142.18	160 (158)
Viet Nam	0.6279	124.06(77.12 to 190.87)	17.53	106.54	132 (127)
Yemen	0.4504	203.38(113.26 to 320.05)	17.54	185.83	177 (177)
Zambia	0.5059	191.46(87.99 to 455.86)	17.53	173.94	172 (172)
Zimbabwe	0.4738	211.93(139.19 to 312.4)	17.53	194.4	183 (182)

S13 Table: Absolute and relative cross-country inequality for nasopharynx cancer of age-standardized DALYs rate in middle-aged and older adults from 1990 to 2021.

location	Nasopharynx cancer					
	**SII_1990**	**SII_2021**	**SII_AbsDiff**	**CI_1990**	**CI_2021**	**CI_AbsDiff**
Global	-26.64 (-48.99 to -4.29)	-30.89 (-46.62 to -15.16)	-4.25	-0.2165 (-0.1457 to -0.2873)	-0.1061 (-0.0512 to -0.1611)	0.1104
Andean Latin America	-12.01 (-27.70 to 3.68)	-10.60 (-27.19 to 5.99)	1.41	-0.1049 (0.1108 to -0.3206)	-0.1117 (0.0861 to -0.3095)	-0.0068
Caribbean	1.42 (-18.92 to 21.76)	-4.82 (-24.69 to 15.05)	-6.24	-0.0835 (-0.0153 to -0.1516)	-0.0642 (-0.0064 to -0.1220)	0.0193
Central Asia	-22.94 (-46.76 to 0.87)	0.02 (-7.18 to 7.21)	22.96	-0.0934 (0.0052 to -0.1919)	-0.0250 (0.0804 to -0.1305)	0.0683
Central Europe	14.42 (-0.56 to 29.40)	-13.46 (-42.19 to 15.28)	-27.87	0.0905 (0.1698 to 0.0111)	-0.1336 (-0.0238 to -0.2435)	-0.2241
Central Latin America	6.92 (-2.97 to 16.81)	5.64 (-3.52 to 14.81)	-1.28	-0.0231 (0.0920 to -0.1383)	-0.0823 (0.0285 to -0.1930)	-0.0591
Central Sub-Saharan Africa	0.56 (-13.29 to 14.42)	-6.29 (-14.92 to 2.34)	-6.85	-0.0178 (0.0342 to -0.0698)	0.0037 (0.0329 to -0.0256)	0.0214
East Asia	-70.74 (-967.35 to 825.86)	54.20 (-107.21 to 215.60)	124.94	-0.0052 (0.0232 to -0.0337)	0.0073 (0.0230 to -0.0083)	0.0126
Eastern Europe	15.47 (-18.16 to 49.10)	-14.69 (-27.39 to -1.99)	-30.16	0.0024 (0.1167 to -0.1119)	-0.0864 (-0.0323 to -0.1405)	-0.0888
Eastern Sub-Saharan Africa	-21.57 (-98.44 to 55.29)	43.22 (-34.05 to 120.49)	64.8	-0.0280 (0.1039 to -0.1599)	0.1230 (0.2406 to 0.0054)	0.151
High-income Asia Pacific	-546.88 (-1415.54 to 321.79)	-216.59 (-388.67 to -44.51)	330.29	-0.2740 (0.5611 to -1.1090)	-0.1722 (0.4032 to -0.7475)	0.1018
High-income North America	-881.20 (-1970.09 to 207.69)	-369.49 (-819.85 to 80.87)	511.71	0.0225 (0.2025 to -0.1574)	-0.0026 (0.1335 to -0.1388)	-0.0252
North Africa and Middle East	41.75 (-15.56 to 99.06)	0.56 (-34.86 to 35.98)	-41.2	0.1806 (0.3981 to -0.0368)	0.0001 (0.2664 to -0.2661)	-0.1805
Oceania	9.43 (-76.50 to 95.36)	19.94 (-125.22 to 165.09)	10.51	-0.0172 (0.0719 to -0.1064)	-0.0017 (0.0641 to -0.0675)	0.0155
South Asia	-10.31 (-75.00 to 54.38)	6.66 (-22.32 to 35.64)	16.97	-0.0127 (0.0103 to -0.0357)	-0.0144 (0.0228 to -0.0515)	-0.0017
Southeast Asia	-46.50 (-127.38 to 34.38)	-39.78 (-104.54 to 24.98)	6.72	0.0034 (0.1856 to -0.1789)	-0.0214 (0.1499 to -0.1928)	-0.0248
Southern Latin America	-12.05 (-111.33 to 87.23)	-24.77 (-32.70 to -16.85)	-12.72	0.0376 (0.2971 to -0.2219)	-0.1914 (-0.1166 to -0.2663)	-0.2291
Southern Sub-Saharan Africa	-10.30 (-45.04 to 24.44)	-49.44 (-112.35 to 13.48)	-39.13	-0.0172 (0.0072 to -0.0416)	-0.0743 (-0.0404 to -0.1082)	-0.0571
Western Europe	-25.16 (-49.04 to -1.29)	-20.96 (-32.56 to -9.36)	4.21	-0.1169 (-0.0094 to -0.2245)	-0.1353 (-0.0825 to -0.1881)	-0.0183
Western Sub-Saharan Africa	-5.06 (-11.58 to 1.47)	-5.98 (-14.60 to 2.63)	-0.93	0.2338 (0.3906 to 0.0771)	0.2228 (0.3889 to 0.0568)	-0.011

SII, slope index of inequality. SII_AbsDiff=SII_1990-SII_2021 (the absolute value of the difference between SII_1990 and SII_2021).

CI, concentration index.CI_AbsDiff=CI_1990-CI_2021 (the absolute value of the difference between CI_1990 and CI_2021).

S14 Table: Frontier analysis on the basis of sociodemographic-index and age-standardized DALYs rate of nasopharynx cancer in middle-aged and older adults from 1990 to 2021.

Location	SDI	Rate of DALYs	Frontier DALYs	Effective difference	Effective difference rank (ASDR)
Afghanistan	0.3372	48.34(22.03 to 78.06)	1.23	47.11	135 (135)
Albania	0.7068	30.5(18.89 to 46.17)	0.8	29.7	102 (101)
Algeria	0.6595	147.1(93.78 to 218.15)	0.79	146.31	192 (192)
American Samoa	0.7237	134.4(88.59 to 195.61)	0.81	133.6	189 (189)
Andorra	0.8694	12.07(6.23 to 20.95)	0.8	11.27	29 (25)
Angola	0.4537	34.15(20.89 to 54.4)	1.2	32.94	112 (111)
Antigua and Barbuda	0.7499	29.63(24.53 to 35.21)	0.79	28.83	100 (99)
Argentina	0.7231	15.17(11.11 to 20.28)	0.79	14.38	44 (43)
Armenia	0.7018	22.33(18 to 27.07)	0.8	21.54	69 (68)
Australia	0.8443	22.48(16.7 to 29.3)	0.81	21.67	70 (69)
Austria	0.8538	15.36(11.24 to 20.36)	0.81	14.55	46 (45)
Azerbaijan	0.6949	11.33(6.68 to 18.06)	0.81	10.52	27 (22)
Bahamas	0.8050	47.27(35.57 to 61.98)	0.81	46.46	134 (133)
Bahrain	0.7530	33.03(18.91 to 55.18)	0.81	32.21	110 (109)
Bangladesh	0.4924	77.21(43.25 to 128.99)	0.8	76.42	157 (156)
Barbados	0.7467	46.23(33.46 to 61.52)	0.8	45.44	132 (131)
Belarus	0.7845	26.24(17.99 to 37.83)	0.8	25.44	83 (82)
Belgium	0.8537	24.23(17.89 to 32.16)	0.79	23.44	75 (74)
Belize	0.6102	26.47(21.51 to 32.41)	0.81	25.66	84 (83)
Benin	0.3735	10.28(5.65 to 17.24)	1.21	9.07	19 (17)
Bermuda	0.8214	37.08(25.53 to 53.22)	0.79	36.28	122 (120)

Location	SDI	Rate of DALYs	Frontier DALYs	Effective difference	Effective difference rank (ASDR)
Bhutan	0.4731	73.53(41.87 to 126.37)	0.81	72.72	154 (153)
Bolivia (Plurinational State of)	0.5990	16.01(10 to 24.56)	0.8	15.21	51 (49)
Bosnia and Herzegovina	0.7231	13.59(9.98 to 18.01)	0.8	12.79	34 (33)
Botswana	0.6427	49.31(27.38 to 81.59)	0.8	48.52	137 (136)
Brazil	0.6530	18.08(16.18 to 20.09)	0.8	17.28	56 (55)
Brunei Darussalam	0.8102	216.46(147.58 to 305.77)	0.79	215.67	201 (201)
Bulgaria	0.7682	35.85(26.13 to 48.97)	0.81	35.04	118 (117)
Burkina Faso	0.2851	12.56(7.15 to 20)	2.93	9.63	21 (27)
Burundi	0.2894	121.05(69.87 to 198.28)	3.96	117.1	180 (181)
Cabo Verde	0.5335	14.91(8.78 to 23.45)	0.8	14.11	41 (40)
Cambodia	0.4736	167.58(104.99 to 257.2)	1.17	166.41	195 (195)
Cameroon	0.4797	13.01(7.53 to 21.08)	0.8	12.2	32 (29)
Canada	0.8732	18.18(13.77 to 23.45)	0.8	17.38	57 (56)
Central African Republic	0.3092	40.94(24.77 to 63.93)	5.34	35.61	120 (126)
Chad	0.2404	12.84(7.35 to 20.68)	8.19	4.66	6 (28)
Chile	0.7715	6.23(4.66 to 8.25)	0.79	5.44	9 (5)
China	0.7216	126.19(98.74 to 159.58)	0.8	125.39	183 (182)
Colombia	0.6554	13.17(9.23 to 18.27)	0.82	12.35	33 (32)
Comoros	0.4760	104.16(56.92 to 177.98)	0.81	103.36	169 (168)
Congo	0.5831	36.77(22.53 to 56.4)	0.8	35.98	121 (119)
Cook Islands	0.7791	15.14(9.56 to 23.08)	0.8	14.35	43 (42)
Costa Rica	0.7003	27.87(20.28 to 37.47)	0.82	27.05	94 (93)
Coted'Ivoire	0.4259	27.81(15.52 to 46.48)	1.24	26.57	92 (92)

Location	SDI	Rate of DALYs	Frontier DALYs	Effective difference	Effective difference rank (ASDR)
Croatia	0.7983	24.3(18.33 to 31.95)	0.8	23.5	76 (75)
Cuba	0.6687	45.93(33.14 to 62.68)	0.8	45.13	131 (130)
Cyprus	0.8356	12.15(7.98 to 18.23)	0.81	11.34	30 (26)
Czechia	0.8285	19.9(13.97 to 27.38)	0.79	19.1	63 (62)
Democratic People's Republic of Korea	0.5699	131.54(80.37 to 199.45)	0.81	130.74	187 (186)
Democratic Republic of the Congo	0.3832	32.2(19.04 to 52.98)	1.24	30.96	103 (104)
Denmark	0.8964	11.03(7.9 to 14.94)	0.8	10.23	26 (20)
Djibouti	0.4880	115.22(58.97 to 212.38)	0.8	114.43	179 (178)
Dominica	0.7470	42.01(27.43 to 62.32)	0.8	41.21	128 (127)
Dominican Republic	0.6194	31.98(20.04 to 48.85)	0.79	31.19	105 (103)
Ecuador	0.6610	8.55(5.73 to 12.25)	0.78	7.77	15 (11)
Egypt	0.6068	5.75(3.83 to 8.38)	0.79	4.97	8 (4)
El Salvador	0.5638	16.07(11.75 to 21.35)	0.81	15.26	52 (50)
Equatorial Guinea	0.6579	27.98(15.66 to 46.55)	0.79	27.19	96 (95)
Eritrea	0.4039	136.07(84.25 to 212.96)	1.21	134.86	191 (191)
Estonia	0.8449	22.52(15.46 to 31.25)	0.81	21.72	71 (70)
Eswatini	0.5855	71.45(42.02 to 112.63)	0.79	70.66	151 (150)
Ethiopia	0.3588	107.91(68.16 to 171.02)	1.2	106.7	174 (173)
Fiji	0.6751	28.42(18.21 to 43.2)	0.79	27.63	97 (96)
Finland	0.8598	6.93(4.99 to 9.33)	0.8	6.14	12 (8)
France	0.8384	30.18(21.61 to 40.99)	0.81	29.37	101 (100)
Gabon	0.6347	34.33(21.26 to 52.94)	0.82	33.51	113 (112)
Gambia	0.4097	14.71(9.23 to 22.07)	1.21	13.49	36 (39)

Location	SDI	Rate of DALYs	Frontier DALYs	Effective difference	Effective difference rank (ASDR)
Georgia	0.7325	37.63(26.21 to 50.97)	0.82	36.81	124 (122)
Germany	0.9030	15.57(11.49 to 20.41)	0.79	14.77	47 (46)
Ghana	0.5649	0.87(0.48 to 1.46)	0.81	0.06	2 (1)
Greece	0.7919	34.82(29.45 to 40.93)	0.8	34.02	115 (114)
Greenland	0.8262	374.89(251.9 to 543.58)	0.8	374.09	203 (203)
Grenada	0.6690	48.18(35.5 to 65.08)	0.79	47.39	136 (134)
Guam	0.8040	134.45(102.71 to 172.41)	0.8	133.65	190 (190)
Guatemala	0.5400	15.71(12.43 to 19.34)	0.8	14.91	48 (47)
Guinea	0.3364	25.17(15.19 to 39.36)	1.22	23.95	79 (79)
Guinea-Bissau	0.3531	16.3(10 to 25)	1.23	15.07	49 (51)
Guyana	0.6508	25.93(16.69 to 39.25)	0.8	25.13	81 (80)
Haiti	0.4483	58.14(32.37 to 91.05)	1.21	56.93	141 (141)
Honduras	0.5130	19.52(12.37 to 29.32)	0.82	18.7	62 (61)
Hungary	0.7908	45.65(34.35 to 59.99)	0.81	44.84	130 (129)
Iceland	0.8764	14.26(10.48 to 18.87)	0.8	13.46	35 (34)
India	0.5754	81.79(69.77 to 95.3)	0.79	81	162 (159)
Indonesia	0.6569	105.85(74.66 to 145.87)	0.8	105.05	171 (170)
Iran (Islamic Republic of)	0.6972	10.17(8.91 to 11.7)	0.8	9.37	20 (16)
Iraq	0.6626	26.55(16.48 to 39.77)	0.8	25.75	86 (85)
Ireland	0.8738	14.41(10.65 to 19.26)	0.81	13.6	39 (37)
Israel	0.8090	18.32(13.62 to 24.33)	0.82	17.49	59 (58)
Italy	0.8058	21.66(19.13 to 24.48)	0.8	20.86	67 (66)
Jamaica	0.6833	38.73(24.86 to 57.79)	0.8	37.94	126 (124)

Location	SDI	Rate of DALYs	Frontier DALYs	Effective difference	Effective difference rank (ASDR)
Japan	0.8712	21.02(19.39 to 22.44)	0.82	20.2	65 (64)
Jordan	0.7253	35.78(22.15 to 56.16)	0.8	34.99	117 (116)
Kazakhstan	0.7251	32.59(25.84 to 41)	0.8	31.79	107 (106)
Kenya	0.5238	161.7(116.8 to 217.36)	0.8	160.9	194 (194)
Kiribati	0.5272	76.28(43.82 to 124.52)	0.8	75.48	156 (155)
Kuwait	0.8467	15.25(10.35 to 21.66)	0.8	14.45	45 (44)
Kyrgyzstan	0.6040	34.61(23.39 to 48.81)	0.8	33.81	114 (113)
Lao People's Democratic Republic	0.4891	130.83(81.87 to 199.23)	0.81	130.02	186 (185)
Latvia	0.8307	26.02(18.3 to 36.14)	0.8	25.22	82 (81)
Lebanon	0.7447	24.64(15.89 to 36.39)	0.8	23.84	77 (76)
Lesotho	0.5104	81.75(49.97 to 125.94)	0.8	80.96	161 (158)
Liberia	0.3524	11.31(6.25 to 19.39)	1.23	10.08	24 (21)
Libya	0.7258	205.89(130.18 to 304.87)	0.81	205.08	199 (199)
Lithuania	0.8565	23.95(17.67 to 31.34)	0.81	23.14	74 (73)
Luxembourg	0.8844	24.94(20.59 to 30.64)	0.8	24.15	80 (78)
Madagascar	0.4002	90.41(51.35 to 147.88)	1.24	89.16	164 (163)
Malawi	0.3846	27.49(17.26 to 42.37)	1.23	26.25	90 (91)
Malaysia	0.7425	415.76(276.88 to 599.07)	0.79	414.97	204 (204)
Maldives	0.6509	15.89(10.38 to 23.7)	0.81	15.09	50 (48)
Mali	0.2686	13.04(7.83 to 21.03)	8.18	4.85	7 (31)
Malta	0.8016	62.33(44.02 to 83.87)	0.8	61.54	147 (146)
Marshall Islands	0.5741	100.24(59.2 to 155.19)	0.83	99.41	167 (166)
Mauritania	0.4989	8.89(4.24 to 17.3)	0.8	8.09	16 (12)

Location	SDI	Rate of DALYs	Frontier DALYs	Effective difference	Effective difference rank (ASDR)
Mauritius	0.7183	60.23(49.57 to 71.32)	0.81	59.42	144 (143)
Mexico	0.6646	10.79(9.11 to 12.55)	0.8	10	23 (18)
Micronesia (Federated States of)	0.5875	94.63(59.24 to 144.23)	0.79	93.83	165 (164)
Monaco	0.9083	17.18(11.31 to 25.29)	0.8	16.38	53 (52)
Mongolia	0.6176	32.83(21.04 to 47.5)	0.82	32.01	108 (107)
Montenegro	0.7958	9.22(6.95 to 12.06)	0.8	8.43	18 (14)
Morocco	0.5627	105.78(63.54 to 164.62)	0.81	104.97	170 (169)
Mozambique	0.3265	9.48(5.92 to 14.55)	1.19	8.29	17 (15)
Myanmar	0.5339	106.17(67.5 to 157.75)	0.8	105.37	172 (171)
Namibia	0.6176	46.82(29.07 to 69.84)	0.82	46	133 (132)
Nauru	0.6252	110.15(58.82 to 185.6)	0.8	109.35	177 (175)
Nepal	0.4332	82.03(51.54 to 127.08)	1.24	80.79	159 (160)
Netherlands	0.8885	20.48(15.66 to 26.27)	0.79	19.69	64 (63)
New Zealand	0.8494	14.49(10.83 to 18.67)	0.83	13.66	40 (38)
Nicaragua	0.5240	15.01(9.92 to 22.07)	0.82	14.19	42 (41)
Niger	0.1681	9.16(4.49 to 17.56)	8.91	0.25	3 (13)
Nigeria	0.5034	78.75(53.93 to 107.62)	0.79	77.95	158 (157)
Niue	0.7262	71.47(44.07 to 109.73)	0.8	70.67	152 (151)
North Macedonia	0.7506	26.5(19.14 to 36.48)	0.79	25.7	85 (84)
Northern Mariana Islands	0.7715	131.85(86.79 to 191.53)	0.8	131.05	188 (187)
Norway	0.9161	6.66(5.7 to 7.81)	0.8	5.87	11 (7)
Oman	0.7734	23.01(14.11 to 35.17)	0.82	22.19	72 (71)
Pakistan	0.5040	107.2(77.12 to 148.58)	0.81	106.39	173 (172)

Location	SDI	Rate of DALYs	Frontier DALYs	Effective difference	Effective difference rank (ASDR)
Palau	0.7540	3.24(1.9 to 5.23)	0.8	2.44	5 (3)
Palestine	0.6310	17.51(11.8 to 25.05)	0.8	16.72	54 (53)
Panama	0.7089	21.16(15.56 to 27.4)	0.8	20.36	66 (65)
Papua New Guinea	0.4178	72.81(37.25 to 130.49)	1.23	71.58	153 (152)
Paraguay	0.6357	14.39(8.82 to 21.84)	0.8	13.59	37 (36)
Peru	0.6621	8.27(5.29 to 12.5)	0.81	7.46	14 (10)
Philippines	0.6512	127.46(103.9 to 154.25)	0.79	126.67	184 (183)
Poland	0.8120	27.96(24.79 to 31.07)	0.82	27.14	95 (94)
Portugal	0.7442	32.51(23.85 to 44.02)	0.82	31.69	106 (105)
Puerto Rico	0.8255	27.4(19.13 to 37.81)	0.82	26.59	93 (90)
Qatar	0.8469	19(10.93 to 32.58)	0.79	18.2	61 (60)
Republic of Korea	0.8867	18.78(13.31 to 25.82)	0.79	17.98	60 (59)
Republic of Moldova	0.7322	61.47(49.45 to 76.18)	0.79	60.68	146 (145)
Romania	0.7685	60.31(44.11 to 80.15)	0.79	59.51	145 (144)
Russian Federation	0.8085	24.65(21.94 to 28.01)	0.8	23.85	78 (77)
Rwanda	0.4356	119.46(70.84 to 190.12)	1.24	118.22	182 (180)
Saint Kitts and Nevis	0.7550	58.74(44.44 to 74.46)	0.79	57.94	143 (142)
Saint Lucia	0.6725	57.49(44.09 to 72.86)	0.81	56.68	140 (139)
Saint Vincent and the Grenadines	0.6372	50.13(40.79 to 61.5)	0.8	49.34	138 (137)
Samoa	0.5934	129.67(83.64 to 193.25)	0.81	128.86	185 (184)
San Marino	0.8880	26.64(13.99 to 44.96)	0.8	25.84	87 (86)
Sao Tome and Principe	0.5054	1.2(0.75 to 1.85)	0.81	0.39	4 (2)
Saudi Arabia	0.8151	75.51(48.71 to 113.08)	0.81	74.7	155 (154)

Location	SDI	Rate of DALYs	Frontier DALYs	Effective difference	Effective difference rank (ASDR)
Senegal	0.4081	10.94(5.74 to 19.19)	1.22	9.73	22 (19)
Serbia	0.7924	29.26(19.57 to 42.44)	0.79	28.46	99 (98)
Seychelles	0.7302	118.86(76.79 to 176.31)	0.79	118.07	181 (179)
Sierra Leone	0.3587	11.33(6.44 to 18.76)	1.22	10.12	25 (23)
Singapore	0.8561	172.78(128.13 to 225.25)	0.79	171.99	196 (196)
Slovakia	0.8106	39.32(24.39 to 60.82)	0.81	38.52	127 (125)
Slovenia	0.8424	11.52(7.8 to 16.44)	0.82	10.71	28 (24)
Solomon Islands	0.4294	96.05(57.31 to 153.1)	1.23	94.82	166 (165)
Somalia	0.0777	134.32(78.59 to 224.19)	134.32	0	1 (188)
South Africa	0.6796	42.93(37.3 to 49.23)	0.79	42.13	129 (128)
South Sudan	0.2784	112.81(63.76 to 196.74)	5.05	107.77	176 (177)
Spain	0.7693	26.8(19.8 to 35.67)	0.81	25.98	88 (87)
Sri Lanka	0.7015	52.68(29.81 to 84.92)	0.8	51.88	139 (138)
Sudan	0.5419	23.24(14.03 to 36.61)	0.79	22.45	73 (72)
Suriname	0.6337	58(37.19 to 86.17)	0.8	57.2	142 (140)
Sweden	0.8869	6.57(4.87 to 8.58)	0.8	5.77	10 (6)
Switzerland	0.9331	14.39(10.38 to 19.46)	0.79	13.6	38 (35)
Syrian Arab Republic	0.6230	7.89(5.53 to 10.82)	0.82	7.07	13 (9)
Taiwan (Province of China)	0.8747	184.82(139.47 to 240.5)	0.79	184.03	197 (197)
Tajikistan	0.5415	32.91(19.47 to 52.37)	0.8	32.11	109 (108)
Thailand	0.6825	102.93(68.74 to 148.19)	0.8	102.13	168 (167)
Timor-Leste	0.4447	112.35(68.74 to 178.44)	1.23	111.12	178 (176)
Togo	0.4085	13.02(7.66 to 20.74)	1.25	11.77	31 (30)

Location	SDI	Rate of DALYs	Frontier DALYs	Effective difference	Effective difference rank (ASDR)
Tokelau	0.6864	69.61(41.1 to 109.78)	0.79	68.82	150 (149)
Tonga	0.6263	63.19(37.48 to 101.75)	0.8	62.4	148 (147)
Trinidad and Tobago	0.7688	31.78(22.61 to 42.57)	0.81	30.98	104 (102)
Tunisia	0.6824	152.76(92.44 to 236.84)	0.8	151.96	193 (193)
Turkey	0.7127	37.79(25.59 to 54.08)	0.79	37	125 (123)
Turkmenistan	0.6822	35.88(26.69 to 48.67)	0.8	35.08	119 (118)
Tuvalu	0.5766	83.99(54.45 to 125.83)	0.81	83.18	163 (162)
Uganda	0.4233	226.38(140.6 to 346.04)	1.21	225.17	202 (202)
Ukraine	0.7608	35.16(22.2 to 53.95)	0.79	34.37	116 (115)
United Arab Emirates	0.8493	37.31(24.26 to 56.42)	0.8	36.52	123 (121)
United Kingdom	0.8590	17.87(16.83 to 18.91)	0.79	17.07	55 (54)
United Republic of Tanzania	0.4466	108.99(64.02 to 174.64)	1.23	107.76	175 (174)
United States Virgin Islands	0.8218	27.18(16.73 to 42.15)	0.81	26.38	91 (89)
United States of America	0.8624	18.19(17.14 to 19.16)	0.79	17.4	58 (57)
Uruguay	0.7193	26.84(19.62 to 35.94)	0.81	26.03	89 (88)
Uzbekistan	0.6626	33.64(23.74 to 47.29)	0.8	32.84	111 (110)
Vanuatu	0.4731	82.03(52.02 to 124.64)	1.11	80.92	160 (161)
Venezuela (Bolivarian Republic of)	0.5965	22.33(15.74 to 30.99)	0.8	21.53	68 (67)
Viet Nam	0.6279	215.47(131.89 to 331.01)	0.8	214.67	200 (200)
Yemen	0.4504	29.09(17.62 to 44.49)	1.24	27.85	98 (97)
Zambia	0.5059	191.22(70.77 to 373.59)	0.81	190.41	198 (198)
Zimbabwe	0.4738	66.68(42.4 to 99.22)	0.79	65.89	149 (148)

S15 Table: Absolute and relative cross-country inequality for lip and oral cavity cancer of age-standardized DALYs rate in middle-aged and older adults from 1990 to 2021.

**location**	**Lip and oral cavity cancer**					
	**SII_1990**	**SII_2021**	**SII_AbsDiff**	**CI_1990**	**CI_2021**	**CI_AbsDiff**
Global	31.64 (-10.43 to 73.71)	-12.77 (-47.32 to 21.79)	-44.4	-0.1477 (-0.0896 to -0.2058)	-0.2477 (-0.1812 to -0.3141)	-0.1
Andean Latin America	-55.38 (-93.74 to -17.01)	-48.28 (-113.73 to 17.17)	7.1	-0.0816 (-0.0009 to -0.1623)	-0.0716 (0.0339 to -0.1771)	0.01
Caribbean	60.03 (-49.14 to 169.19)	-17.09 (-118.70 to 84.52)	-77.11	0.0400 (0.0858 to -0.0058)	-0.0548 (0.0140 to -0.1235)	-0.0947
Central Asia	-36.61 (-289.32 to 216.11)	71.54 (-47.51 to 190.59)	108.15	0.0186 (0.2197 to -0.1825)	0.0884 (0.2159 to -0.0390)	0.0698
Central Europe	139.50 (4.01 to 274.99)	36.83 (-149.83 to 223.49)	-102.67	0.1201 (0.2297 to 0.0105)	-0.0188 (0.0681 to -0.1057)	-0.1389
Central Latin America	35.78 (-2.50 to 74.06)	3.80 (-36.16 to 43.76)	-31.98	-0.0042 (0.0747 to -0.0831)	-0.0528 (0.0134 to -0.1191)	-0.0487
Central Sub-Saharan Africa	31.92 (-3.64 to 67.48)	29.56 (0.60 to 58.53)	-2.36	-0.0027 (0.0470 to -0.0523)	0.0393 (0.0627 to 0.0159)	0.042
East Asia	133.36 (-321.06 to 587.77)	450.02 (-684.85 to 1584.88)	316.66	0.0214 (0.0694 to -0.0265)	0.0767 (0.2169 to -0.0636)	0.0552
Eastern Europe	-53.84 (-145.08 to 37.40)	-12.55 (-94.98 to 69.89)	41.3	-0.0044 (0.0547 to -0.0636)	-0.0240 (0.0054 to -0.0534)	-0.0196
Eastern Sub-Saharan Africa	48.08 (-52.35 to 148.50)	111.38 (32.14 to 190.63)	63.31	0.0565 (0.1167 to -0.0036)	0.1295 (0.1922 to 0.0668)	0.0729
High-income Asia Pacific	-134.75 (-454.34 to 184.84)	-72.40 (-222.83 to 78.03)	62.35	-0.0092 (0.0193 to -0.0377)	-0.0601 (-0.0089 to -0.1113)	-0.051
High-income North America	-334.09 (-778.03 to 109.85)	-161.42 (-342.16 to 19.32)	172.67	0.0078 (0.0210 to -0.0054)	-0.0058 (0.0031 to -0.0146)	-0.0136
North Africa and Middle East	67.04 (31.58 to 102.51)	36.80 (9.46 to 64.14)	-30.25	0.1432 (0.2440 to 0.0423)	0.0663 (0.1544 to -0.0218)	-0.0769
Oceania	32.85 (-54.82 to 120.52)	38.29 (-78.76 to 155.34)	5.44	0.1206 (0.2809 to -0.0396)	0.1016 (0.2250 to -0.0218)	-0.019
South Asia	-28.18 (-482.09 to 425.72)	192.66 (60.28 to 325.03)	220.84	-0.0892 (0.0232 to -0.2015)	-0.0610 (0.0950 to -0.2170)	0.0282

location	Lip and oral cavity cancer					
	SII_1990	SII_2021	SII_AbsDiff	CI_1990	CI_2021	CI_AbsDiff
Southeast Asia	119.68 (9.26 to 230.10)	121.80 (24.98 to 218.61)	2.12	0.0548 (0.1692 to -0.0595)	0.0387 (0.1382 to -0.0609)	-0.0162
Southern Latin America	-46.40 (-439.37 to 346.57)	-152.82 (-200.73 to -104.91)	-106.41	0.0302 (0.2323 to -0.1719)	-0.1503 (-0.0929 to -0.2077)	-0.1805
Southern Sub-Saharan Africa	93.47 (-254.03 to 440.97)	-78.82 (-400.93 to 243.28)	-172.3	0.0505 (0.1194 to -0.0183)	-0.0173 (0.0475 to -0.0821)	-0.0678
Western Europe	-58.82 (-144.43 to 26.78)	-47.50 (-93.15 to -1.84)	11.33	-0.0361 (0.0496 to -0.1219)	-0.0193 (0.0191 to -0.0577)	0.0168
Western Sub-Saharan Africa	-53.11 (-105.91 to -0.31)	-74.93 (-122.24 to -27.62)	-21.82	-0.1869 (-0.0558 to -0.3179)	-0.1909 (-0.0766 to -0.3052)	-0.004

SII, slope index of inequality. SII_AbsDiff=SII_1990-SII_2021 (the absolute value of the difference between SII_1990 and SII_2021).

CI, concentration index.CI_AbsDiff=CI_1990-CI_2021 (the absolute value of the difference between CI_1990 and CI_2021).

S16 Table: Frontier analysis on the basis of sociodemographic-index and age-standardized DALYs rate of lip and oral cavity cancer in middle-aged and older adults from 1990 to 2021.

Location	SDI	Rate of DALYs	Frontier DALYs	Effective difference	Effective difference rank (ASDR)
Afghanistan	0.337199998	64.06(36.53 to 103)	7.46	56.6	24 (20)
Albania	0.706849791	117.79(76.91 to 170.68)	7.52	110.27	86 (85)
Algeria	0.659500924	60.5(39.24 to 89.42)	7.44	53.05	20 (16)
American Samoa	0.723727533	49.98(35.42 to 68.97)	7.46	42.52	14 (11)
Andorra	0.869444113	69.68(39.04 to 115.02)	7.42	62.27	33 (28)
Angola	0.453721949	166.49(107.17 to 248.99)	7.45	159.03	132 (131)
Antigua and Barbuda	0.749886887	130.89(111.23 to 152.74)	7.44	123.45	96 (95)
Argentina	0.723122973	116.26(96.57 to 138.75)	7.45	108.81	85 (83)
Armenia	0.701833194	96.31(79.04 to 113.83)	7.5	88.81	57 (53)
Australia	0.844252814	113.78(94.34 to 135.76)	7.52	106.26	82 (80)
Austria	0.853837004	143.56(118.13 to 171.52)	7.43	136.13	112 (111)
Azerbaijan	0.694851274	59.8(37.05 to 91.7)	7.53	52.26	18 (14)
Bahamas	0.805020668	229.94(178.02 to 293.91)	7.45	222.49	164 (164)
Bahrain	0.753043204	97.02(56.9 to 149.84)	7.44	89.58	59 (55)
Bangladesh	0.492420885	393.7(225.98 to 619.49)	7.44	386.25	193 (193)
Barbados	0.746748764	152.53(114.73 to 196.96)	7.45	145.08	121 (119)
Belarus	0.784484711	329.62(245.97 to 434.71)	7.44	322.18	188 (188)
Belgium	0.853654016	154.21(126.67 to 185.78)	7.42	146.79	123 (120)
Belize	0.610229002	92.81(76.63 to 109.66)	7.45	85.36	54 (50)
Benin	0.373486574	89.04(54.75 to 136.43)	7.42	81.61	46 (42)

Location	SDI	Rate of DALYs	Frontier DALYs	Effective difference	Effective difference rank (ASDR)
Bermuda	0.821365422	163.11(122.32 to 212.05)	7.45	155.66	129 (128)
Bhutan	0.473062378	372.81(232.79 to 566.33)	7.45	365.37	192 (192)
Bolivia (Plurinational State of)	0.599010799	98.98(60.47 to 150.02)	7.48	91.5	62 (60)
Bosnia and Herzegovina	0.723077893	162.54(118.9 to 217.29)	7.5	155.04	128 (127)
Botswana	0.642721629	250.43(148.06 to 393.57)	7.52	242.9	174 (174)
Brazil	0.653043887	195.81(179.03 to 212.52)	7.43	188.38	147 (146)
Brunei Darussalam	0.810234367	177.48(126.52 to 241.83)	7.49	170	139 (137)
Bulgaria	0.768150939	224.93(179.56 to 274.7)	7.49	217.44	162 (162)
Burkina Faso	0.285118402	97.53(62.09 to 146.81)	45.31	52.23	17 (57)
Burundi	0.289374365	213.59(131.38 to 327.74)	18.93	194.65	150 (156)
Cabo Verde	0.533534539	274.31(174.86 to 416.36)	7.47	266.85	177 (177)
Cambodia	0.473621491	208.07(132.98 to 307.3)	7.51	200.56	154 (153)
Cameroon	0.479691223	110.26(70.53 to 166.68)	7.44	102.82	76 (74)
Canada	0.87317068	107.02(89.47 to 127.13)	7.63	99.39	72 (71)
Central African Republic	0.30916769	155.59(97.49 to 245.93)	15.09	140.51	118 (123)
Chad	0.240436019	96.33(62.33 to 140.45)	61.03	35.3	10 (54)
Chile	0.771514716	60.79(50.53 to 72.68)	7.44	53.35	21 (17)
China	0.72162976	92.18(71.19 to 116.83)	7.44	84.73	52 (48)
Colombia	0.655442913	82.46(63.94 to 103.4)	7.58	74.88	41 (37)
Comoros	0.475978688	218.35(140.08 to 329.44)	7.58	210.77	158 (158)
Congo	0.583075236	177.11(114.89 to 259.81)	7.47	169.64	138 (136)
Cook Islands	0.779109955	100.38(65.71 to 146.59)	7.45	92.93	64 (62)

Location	SDI	Rate of DALYs	Frontier DALYs	Effective difference	Effective difference rank (ASDR)
Costa Rica	0.700340477	84.59(67.99 to 103.21)	7.46	77.14	43 (39)
Coted'Ivoire	0.425941883	146.11(87.55 to 226.04)	7.44	138.68	114 (113)
Croatia	0.798341027	219.95(179.46 to 266.8)	7.47	212.48	160 (160)
Cuba	0.668729864	238.34(186.22 to 299.38)	7.49	230.85	167 (167)
Cyprus	0.835630545	79.55(54.67 to 113.4)	7.44	72.11	40 (36)
Czechia	0.828450433	213.82(175.77 to 258.79)	7.46	206.36	157 (157)
Democratic People's Republic of Korea	0.569854634	95.94(59.14 to 147.27)	7.45	88.48	55 (51)
Democratic Republic of the Congo	0.383179849	139.21(88.21 to 205.04)	7.5	131.71	107 (106)
Denmark	0.896424204	129.73(113.77 to 145.92)	7.51	122.22	94 (93)
Djibouti	0.487958371	243.94(146.05 to 378.93)	7.44	236.5	171 (171)
Dominica	0.746967185	230.84(155.82 to 328.48)	7.54	223.3	165 (165)
Dominican Republic	0.619388201	190.49(118.02 to 301.01)	7.44	183.06	144 (142)
Ecuador	0.661017053	68.14(50.75 to 90.29)	7.46	60.69	29 (25)
Egypt	0.606787094	45.74(33.08 to 61.38)	7.52	38.22	11 (9)
El Salvador	0.563775188	86.42(64.45 to 114.12)	7.49	78.93	44 (40)
Equatorial Guinea	0.657857456	163.52(99.32 to 252.06)	7.49	156.03	130 (129)
Eritrea	0.403863943	235.54(150.19 to 347.97)	7.45	228.09	166 (166)
Estonia	0.844917787	208.73(162.6 to 259.23)	7.45	201.28	155 (154)
Eswatini	0.585459713	292.66(170.9 to 484.9)	7.67	284.99	181 (181)
Ethiopia	0.358823295	155.96(119.89 to 198.74)	7.55	148.41	126 (125)
Fiji	0.675051631	155.62(101.2 to 227.71)	7.45	148.17	125 (124)
Finland	0.859831368	98.01(85.5 to 110.85)	7.54	90.48	60 (58)

Location	SDI	Rate of DALYs	Frontier DALYs	Effective difference	Effective difference rank (ASDR)
France	0.838364875	164.35(136.37 to 196.85)	7.44	156.92	131 (130)
Gabon	0.634691393	187.51(121.57 to 277.74)	7.46	180.05	141 (139)
Gambia	0.40971416	104.99(66.81 to 154.31)	7.48	97.51	70 (68)
Georgia	0.732473604	201.78(170.54 to 234.57)	7.57	194.21	149 (148)
Germany	0.902957091	142.47(117.71 to 168.36)	7.45	135.02	110 (109)
Ghana	0.56493039	27.69(17.63 to 41.17)	7.51	20.18	4 (2)
Greece	0.791854408	114.14(101 to 127.75)	7.42	106.71	83 (81)
Greenland	0.826210336	262.61(197.3 to 344.92)	7.51	255.09	175 (175)
Grenada	0.668993028	193.32(149.8 to 241.78)	7.48	185.84	145 (144)
Guam	0.803982203	130.64(97.38 to 170.83)	7.47	123.17	95 (94)
Guatemala	0.539972424	68.7(56.71 to 82.07)	7.47	61.23	31 (26)
Guinea	0.336401293	298.48(194.78 to 429.65)	7.44	291.04	182 (182)
Guinea-Bissau	0.353109621	125.61(79.9 to 191.42)	7.46	118.14	93 (92)
Guyana	0.650812335	125.08(89.76 to 169.66)	7.5	117.58	92 (91)
Haiti	0.448278285	189.25(111.71 to 300.48)	7.5	181.76	143 (141)
Honduras	0.513037248	97.06(62.76 to 143.42)	7.6	89.46	58 (56)
Hungary	0.790754768	409.41(341.56 to 482.98)	7.48	401.93	197 (197)
Iceland	0.87636168	108.88(92.49 to 125.93)	7.43	101.45	75 (72)
India	0.575401649	498.71(420.51 to 576.04)	7.44	491.27	199 (199)
Indonesia	0.656868336	147.62(105.81 to 194.23)	7.42	140.19	116 (115)
Iran (Islamic Republic of)	0.697207398	32.37(27.45 to 37.52)	7.43	24.94	5 (3)
Iraq	0.662626231	74.21(47.77 to 105.98)	7.56	66.66	36 (31)

Location	SDI	Rate of DALYs	Frontier DALYs	Effective difference	Effective difference rank (ASDR)
Ireland	0.87375385	112.56(92.15 to 134.83)	7.47	105.09	80 (78)
Israel	0.809011652	66.8(54.7 to 79.6)	7.52	59.28	26 (22)
Italy	0.805773534	124.21(112.99 to 133.8)	7.48	116.73	91 (90)
Jamaica	0.683263064	96.21(68.94 to 131.53)	7.43	88.77	56 (52)
Japan	0.871241813	90.03(81.46 to 96.06)	7.45	82.58	47 (43)
Jordan	0.725307227	68.11(44.07 to 101.39)	7.51	60.6	28 (24)
Kazakhstan	0.725144495	170.72(140.41 to 204.68)	7.44	163.28	135 (133)
Kenya	0.523768077	303.21(227.79 to 390.38)	7.46	295.76	184 (184)
Kiribati	0.527186583	406.88(241.48 to 652.04)	7.44	399.44	196 (196)
Kuwait	0.846651055	38.82(29.7 to 50.23)	7.46	31.36	8 (6)
Kyrgyzstan	0.603979328	136.93(103.96 to 176.61)	7.48	129.45	101 (100)
Lao People's Democratic Republic	0.489136091	158.99(104.07 to 231.27)	7.53	151.46	127 (126)
Latvia	0.830663516	285.95(224.99 to 360.31)	7.46	278.49	180 (180)
Lebanon	0.744746351	77.16(52.08 to 111)	7.5	69.66	38 (33)
Lesotho	0.510393066	324.28(189.7 to 527.23)	7.43	316.85	187 (187)
Liberia	0.352442452	90.12(52.94 to 141.11)	7.5	82.62	48 (44)
Libya	0.725771399	101.94(67.47 to 152.25)	7.43	94.51	67 (64)
Lithuania	0.856484049	307.58(242.56 to 379.65)	7.48	300.1	186 (186)
Luxembourg	0.884428955	133.68(113.57 to 154.55)	7.45	126.23	97 (96)
Madagascar	0.400246943	176.39(115.35 to 258.83)	7.45	168.94	137 (135)
Malawi	0.384553634	182.12(117.41 to 266.03)	7.47	174.66	140 (138)
Malaysia	0.742523828	227.77(163.43 to 310.19)	7.5	220.27	163 (163)

Location	SDI	Rate of DALYs	Frontier DALYs	Effective difference	Effective difference rank (ASDR)
Maldives	0.650886627	137.55(92.56 to 194.41)	7.43	130.11	103 (102)
Mali	0.268579941	116.71(74.09 to 174.36)	55.62	61.09	30 (84)
Malta	0.801585034	110.74(90.48 to 132.9)	7.43	103.31	77 (75)
Marshall Islands	0.574091128	150.05(92.02 to 231.37)	7.52	142.53	119 (118)
Mauritania	0.4989451	98.83(62.1 to 147.79)	7.44	91.39	61 (59)
Mauritius	0.718260446	205.17(175.58 to 235.66)	7.5	197.67	152 (151)
Mexico	0.664575304	70.17(61.36 to 79.63)	7.5	62.67	34 (29)
Micronesia (Federated States of)	0.587534967	154.32(99.76 to 230.75)	7.62	146.7	122 (121)
Monaco	0.908262831	69.12(47.53 to 98.72)	7.44	61.68	32 (27)
Mongolia	0.617621565	146.05(99.14 to 202.62)	7.46	138.6	113 (112)
Montenegro	0.795800584	202.36(146.84 to 269.25)	7.43	194.93	151 (149)
Morocco	0.562698301	62.51(41.95 to 88.59)	7.5	55.01	23 (19)
Mozambique	0.326462614	154.61(97.64 to 236.16)	7.42	147.19	124 (122)
Myanmar	0.53390084	141.45(91.52 to 209.03)	7.44	134.01	109 (108)
Namibia	0.617564872	489.58(309.45 to 720.77)	7.45	482.12	198 (198)
Nauru	0.625177834	175.59(98.51 to 301.54)	7.47	168.13	136 (134)
Nepal	0.433174635	396.85(252.89 to 595.93)	7.44	389.41	194 (194)
Netherlands	0.888464256	102.63(86.1 to 119.98)	7.48	95.15	69 (67)
New Zealand	0.849442499	102.22(84.96 to 120.59)	7.42	94.8	68 (66)
Nicaragua	0.523958472	45.71(30.75 to 63.77)	7.44	38.28	12 (8)
Niger	0.168072774	78.88(47.13 to 124.11)	63.28	15.6	3 (35)
Nigeria	0.503390833	60.2(43.95 to 80.54)	7.43	52.77	19 (15)

Location	SDI	Rate of DALYs	Frontier DALYs	Effective difference	Effective difference rank (ASDR)
Niue	0.72622205	136.28(90.05 to 198.08)	7.57	128.71	100 (99)
North Macedonia	0.750629703	146.48(105.86 to 199.41)	7.53	138.95	115 (114)
Northern Mariana Islands	0.771535213	572.17(405.66 to 782.4)	7.51	564.66	202 (202)
Norway	0.91613281	88.4(80.9 to 94.3)	7.49	80.91	45 (41)
Oman	0.773391602	74.77(45.95 to 112.5)	7.52	67.24	37 (32)
Pakistan	0.504028689	1204.9(892.71 to 1620.52)	7.44	1197.46	204 (204)
Palau	0.754046931	1162.53(775.81 to 1698.43)	7.49	1155.04	203 (203)
Palestine	0.631011665	38.37(27.19 to 54.04)	7.55	30.82	7 (5)
Panama	0.708864828	92.05(69.11 to 114.46)	7.44	84.61	51 (47)
Papua New Guinea	0.417797443	112.34(62.41 to 174.03)	7.48	104.86	79 (77)
Paraguay	0.635718099	150.02(94.73 to 225.91)	7.44	142.58	120 (117)
Peru	0.662054037	64.3(43.29 to 91.95)	7.44	56.86	25 (21)
Philippines	0.651219329	147.95(123.21 to 175.59)	7.47	140.48	117 (116)
Poland	0.812042809	279.48(251.69 to 306.73)	7.43	272.05	179 (179)
Portugal	0.744151851	194.83(158.64 to 236.88)	7.58	187.25	146 (145)
Puerto Rico	0.825525847	106.88(81.24 to 136.26)	7.45	99.43	73 (70)
Qatar	0.846860584	70.67(41.77 to 108.2)	7.46	63.21	35 (30)
Republic of Korea	0.886675267	61.82(43.5 to 81.78)	7.44	54.38	22 (18)
Republic of Moldova	0.732214875	249.25(210.96 to 291.85)	7.44	241.81	173 (173)
Romania	0.768453864	340.87(273.04 to 420.56)	7.44	333.43	189 (189)
Russian Federation	0.808536005	244.26(219.39 to 267.43)	7.56	236.7	172 (172)
Rwanda	0.435588706	238.76(148.75 to 364.23)	7.54	231.22	169 (169)

Location	SDI	Rate of DALYs	Frontier DALYs	Effective difference	Effective difference rank (ASDR)
Saint Kitts and Nevis	0.754987055	167(130.7 to 206.04)	7.46	159.54	133 (132)
Saint Lucia	0.672509735	211.78(164.46 to 267.29)	7.52	204.26	156 (155)
Saint Vincent and the Grenadines	0.637195963	301.33(251.29 to 360.74)	7.51	293.82	183 (183)
Samoa	0.593392769	118.27(74.86 to 178.68)	7.44	110.82	87 (86)
San Marino	0.888005474	78.09(42.68 to 128.53)	7.43	70.66	39 (34)
Sao Tome and Principe	0.505413747	8.63(5.47 to 13.1)	7.45	1.18	2 (1)
Saudi Arabia	0.815143493	91.29(63.51 to 128.9)	7.49	83.8	49 (45)
Senegal	0.408054193	101.46(62.94 to 155.58)	7.44	94.02	65 (63)
Serbia	0.792416294	207.57(140.85 to 286.62)	7.51	200.06	153 (152)
Seychelles	0.730150775	510.27(337.68 to 735.16)	7.44	502.83	200 (200)
Sierra Leone	0.358665881	91.96(59.15 to 136.81)	7.49	84.48	50 (46)
Singapore	0.856097766	67.73(55.73 to 81.13)	7.43	60.29	27 (23)
Slovakia	0.81061053	354.16(231.09 to 506.58)	7.51	346.65	190 (190)
Slovenia	0.842430731	137.78(109.35 to 171.92)	7.47	130.31	104 (103)
Solomon Islands	0.429360316	140.86(86.47 to 216.79)	7.43	133.43	108 (107)
Somalia	0.077688109	192.01(121.14 to 293.35)	192.01	0	1 (143)
South Africa	0.679626598	239.16(203.66 to 275.64)	7.43	231.73	170 (170)
South Sudan	0.278371125	203.86(127.15 to 310.38)	43.07	160.79	134 (150)
Spain	0.769283698	142.92(116.86 to 173.36)	7.45	135.46	111 (110)
Sri Lanka	0.701534935	359.62(204.69 to 567.71)	7.57	352.05	191 (191)
Sudan	0.541949735	42.15(27.53 to 62.88)	7.5	34.64	9 (7)
Suriname	0.633665739	101.94(66.84 to 152.68)	7.49	94.45	66 (65)

Location	SDI	Rate of DALYs	Frontier DALYs	Effective difference	Effective difference rank (ASDR)
Sweden	0.886880299	92.67(79.02 to 106.83)	7.51	85.16	53 (49)
Switzerland	0.933059111	100.15(81.73 to 121.49)	7.62	92.53	63 (61)
Syrian Arab Republic	0.623004075	34.58(24.34 to 48.09)	7.44	27.15	6 (4)
Taiwan (Province of China)	0.874747053	538.36(433.52 to 654.61)	7.46	530.9	201 (201)
Tajikistan	0.541511187	52.24(35.52 to 73.58)	7.51	44.73	15 (12)
Thailand	0.682547933	238.59(161.75 to 334.04)	7.48	231.1	168 (168)
Timor-Leste	0.444667619	137.45(89.14 to 203.98)	7.46	129.99	102 (101)
Togo	0.408533695	108.88(68.4 to 164.76)	7.45	101.43	74 (73)
Tokelau	0.686425621	135.39(86.76 to 199.28)	7.43	127.96	99 (98)
Tonga	0.626349936	124.01(77.23 to 185.48)	7.49	116.53	90 (89)
Trinidad and Tobago	0.768763254	112.17(82.58 to 148.19)	7.46	104.71	78 (76)
Tunisia	0.682432216	105.1(65.5 to 158.53)	7.5	97.6	71 (69)
Turkey	0.712692673	53.15(36.62 to 73.33)	7.44	45.71	16 (13)
Turkmenistan	0.682160776	199.94(151.28 to 267.51)	7.41	192.52	148 (147)
Tuvalu	0.576620529	139.18(93.52 to 202.3)	7.52	131.66	106 (105)
Uganda	0.423261181	303.57(193.47 to 455.69)	7.44	296.13	185 (185)
Ukraine	0.760773913	276.58(180.91 to 399.49)	7.47	269.11	178 (178)
United Arab Emirates	0.849317734	119.89(80.65 to 173.73)	7.5	112.4	88 (87)
United Kingdom	0.859000182	138.91(130.84 to 145.65)	7.45	131.46	105 (104)
United Republic of Tanzania	0.446568273	219.34(133.54 to 338.04)	7.44	211.9	159 (159)
United States Virgin Islands	0.821830853	83.78(51.26 to 132.73)	7.44	76.34	42 (38)
United States of America	0.862448354	113.59(106.17 to 119.42)	7.48	106.11	81 (79)

Location	SDI	Rate of DALYs	Frontier DALYs	Effective difference	Effective difference rank (ASDR)
Uruguay	0.719283445	187.88(155.17 to 224.5)	7.43	180.44	142 (140)
Uzbekistan	0.662621694	134.25(104.09 to 172.53)	7.45	126.8	98 (97)
Vanuatu	0.473100706	123.49(82.55 to 183.12)	7.43	116.06	89 (88)
Venezuela (Bolivarian Republic of)	0.596513059	115.07(84.96 to 150.67)	7.47	107.6	84 (82)
Viet Nam	0.627933721	267.6(177.02 to 390.63)	7.45	260.15	176 (176)
Yemen	0.450376375	49.46(30.65 to 74.62)	7.51	41.94	13 (10)
Zambia	0.505948954	399.58(167.13 to 687.2)	7.47	392.11	195 (195)
Zimbabwe	0.473819486	224.78(153.13 to 316.83)	7.46	217.32	161 (161)

S1 Fig. Average annual percentage change of ASIR and ASDR for HNC and its subtypes among middle-aged and older adults at national and GBD regional levels from 1990 to 2021. **A**, AAPC of ASIR for HNC. **B**, AAPC of ASDR for HNC. **C**, AAPC of ASIR for larynx cancer. **D**, AAPC of ASDR for larynx cancer. **E**, AAPC of ASIR for nasopharynx cancer. **F**, AAPC of ASDR for nasopharynx cancer. **G**, AAPC of ASIR for lip and oral cavity cancer. **H**, AAPC of ASDR for lip and oral cavity cancer. The black cross represents the ASR for each country, the blue solid dot represents the ASR for GBD regions, and the red text denotes the countries with the maximum and minimum ASR within each GBD region.

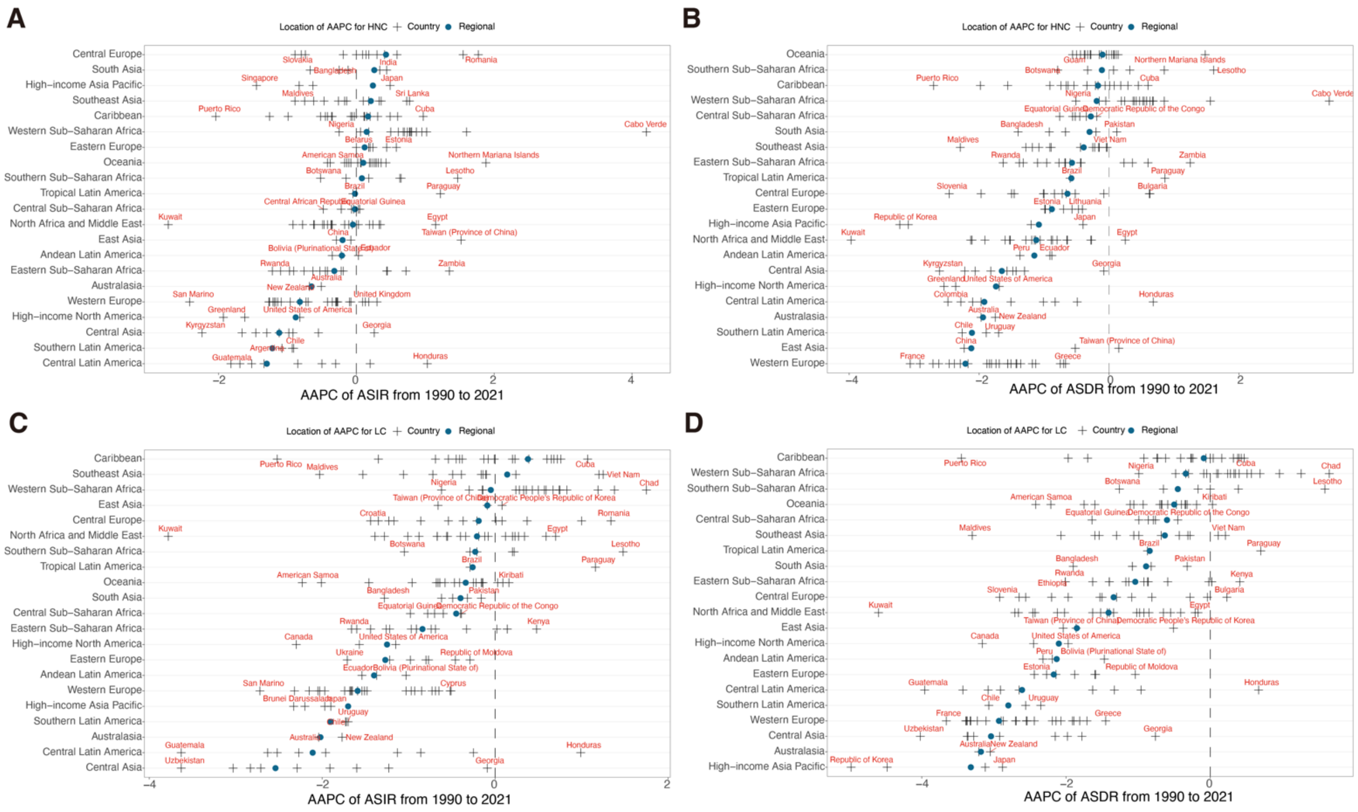

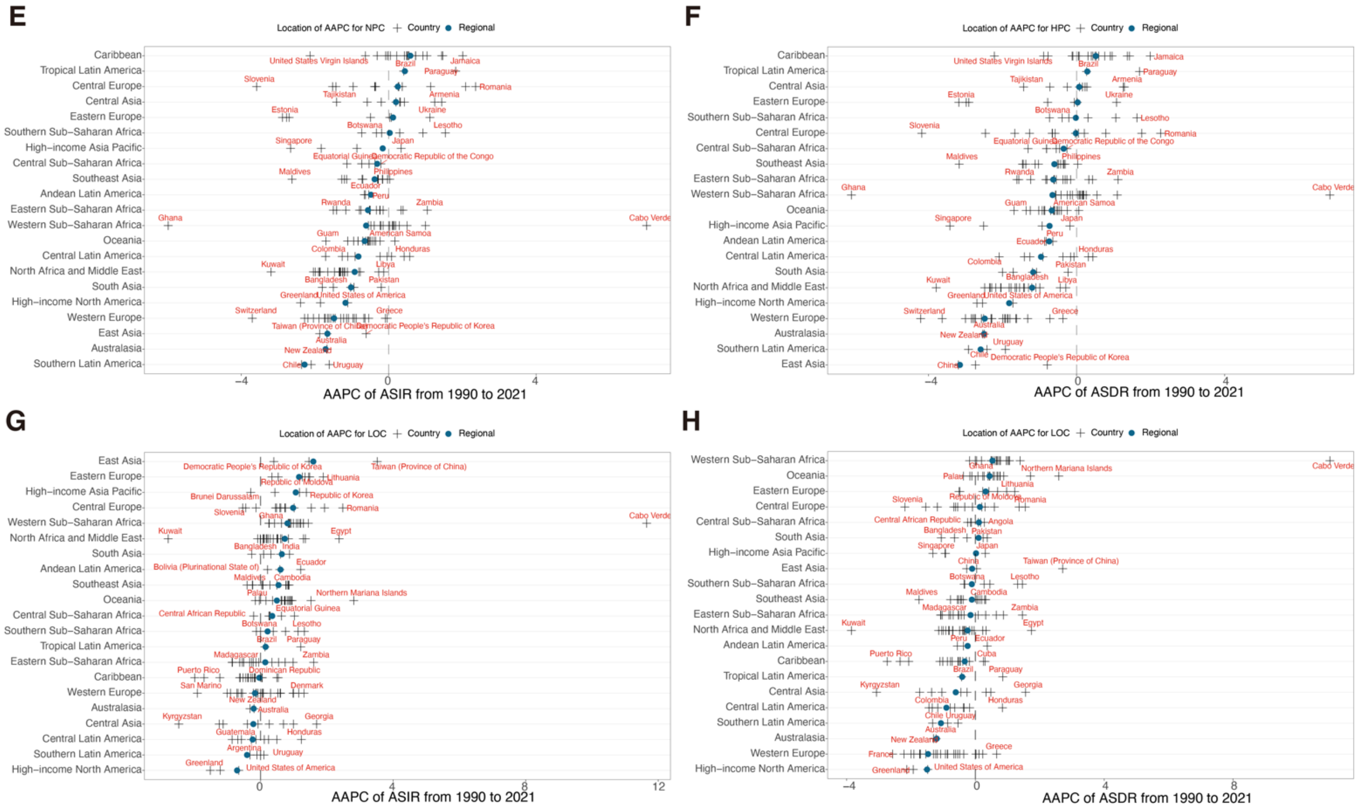
E
Location of AAPC for NPC Country Regional
AAPC of ASIR from 1990 to 2021
F
Location of AAPC for HPC Country Regional
AAPC of ASDR from 1990 to 2021
G
Location of AAPC for LOC Country Regional
AAPC of ASIR from 1990 to 2021
H
Location of AAPC for LOC Country Regional
AAPC of ASDR from 1990 to 2021

S2 Fig. Temporal trend of slope index of inequality and concentration index of head and neck cancer and its subtypes among middle-aged and older adults at global level from 1990 to 2021.

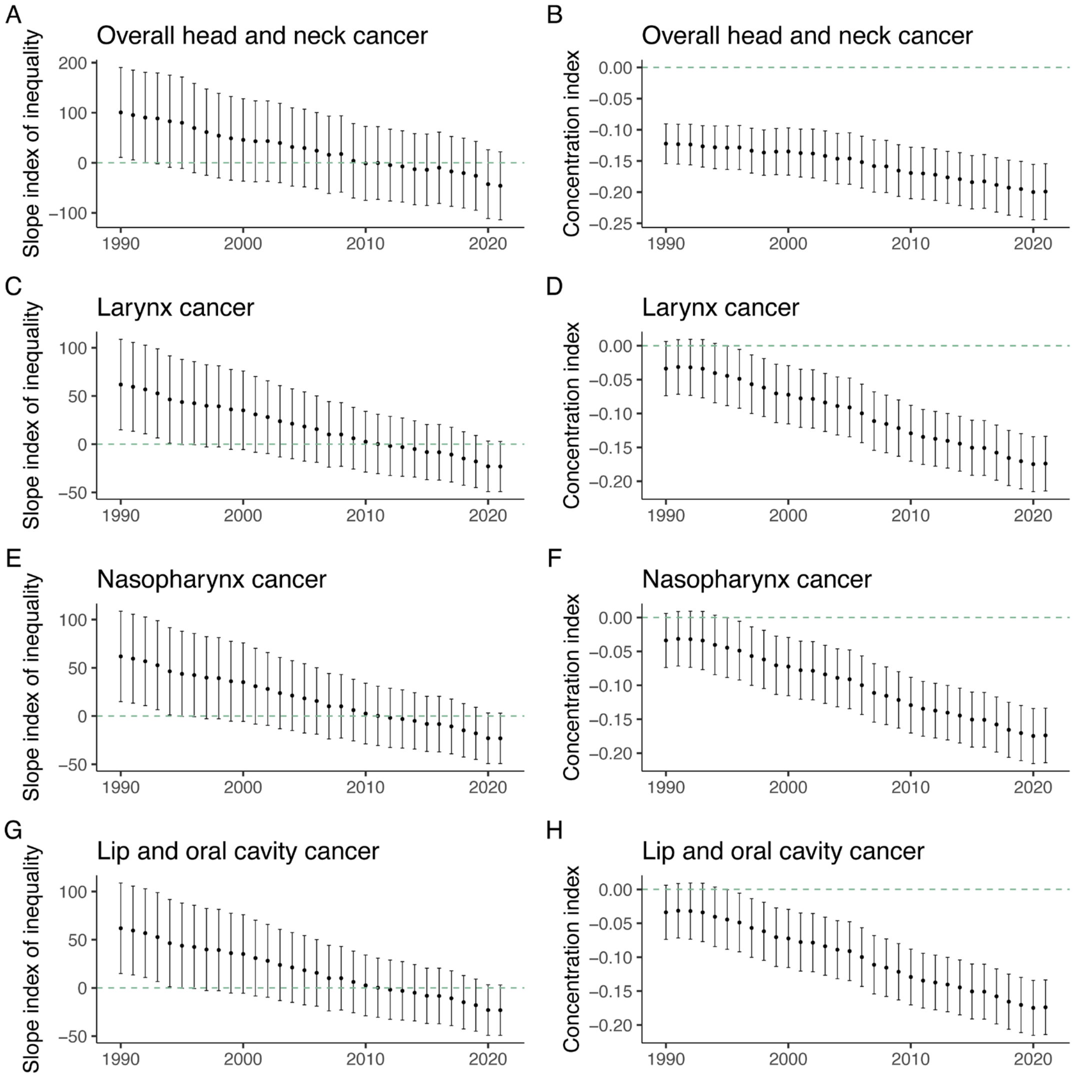

S3 Fig. Temporal trend of slope index of inequality of head and neck cancer and its subtypes among middle-aged and older adults at global and GBD regions from 1990 to 2021.

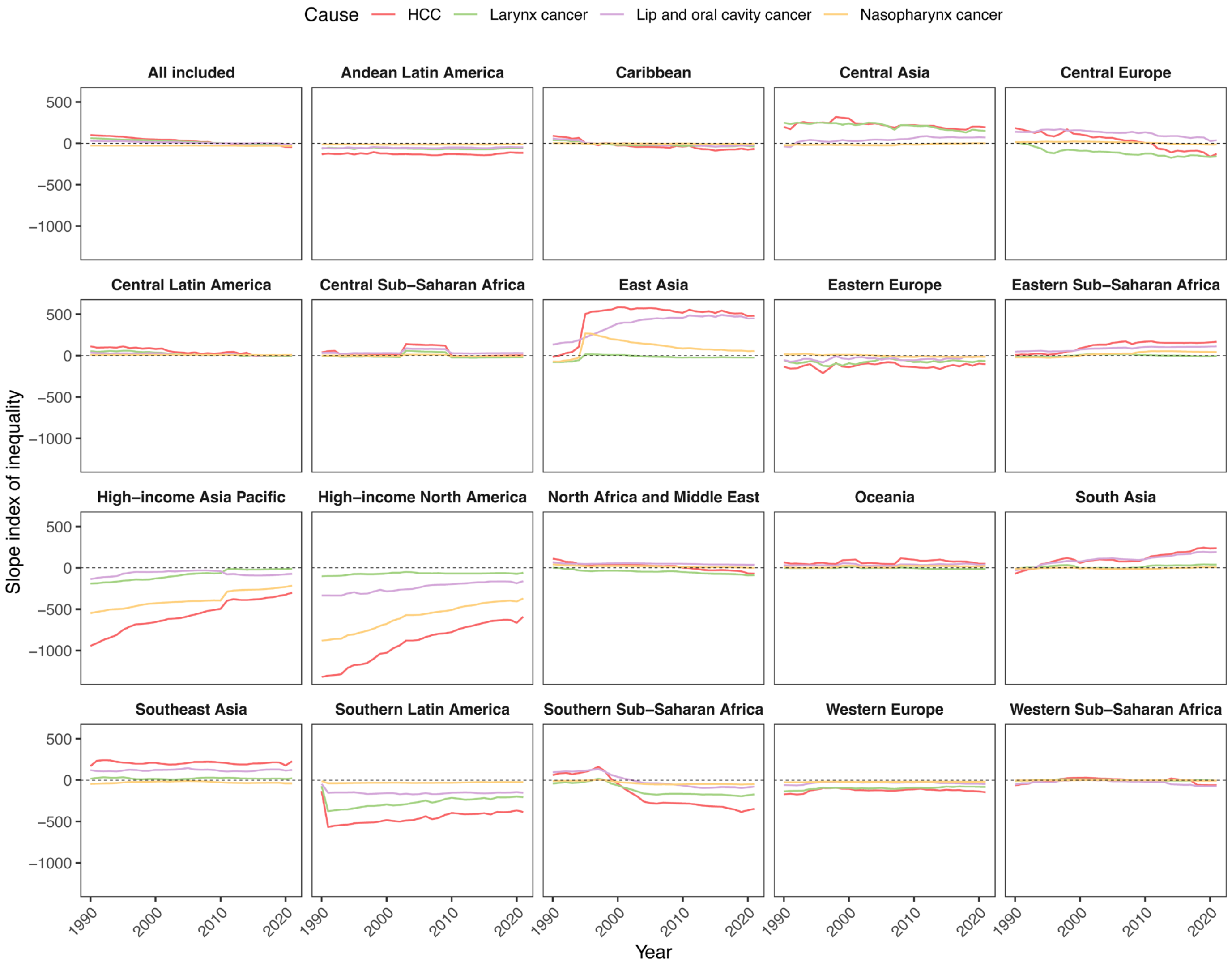

S4 Fig. Temporal trend of concentration index of head and neck cancer and its subtypes among middle-aged and older adults at global and GBD regions from 1990 to 2021.

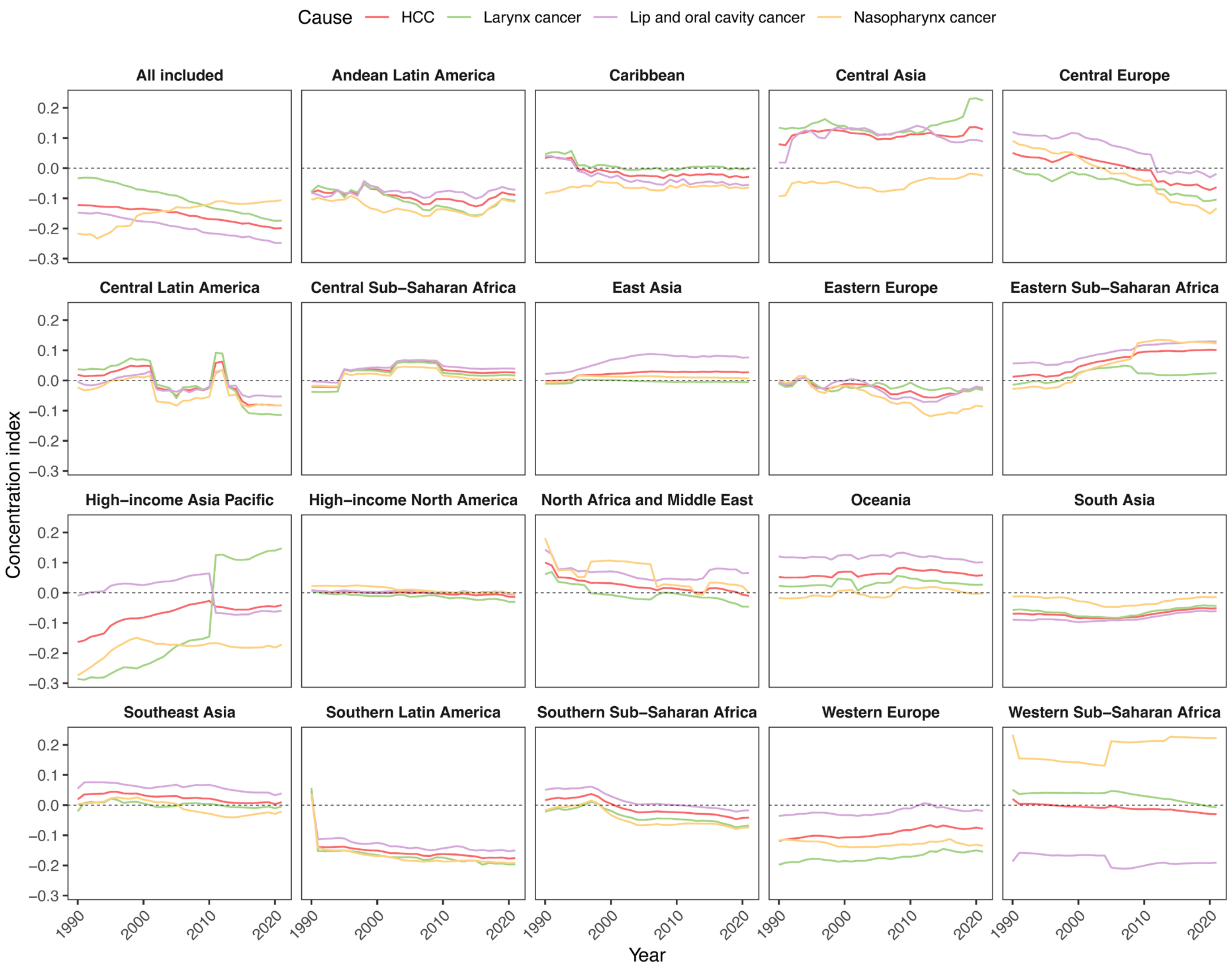

Supplement: S1 File — (PDF) [file pone.0335969.s001.pdf]
